# Three New Prenylated Dihydrobenzofurans and a New Flavonoid Glycoside from the Aerial Parts of *Myrsine seguinii*

**DOI:** 10.3390/molecules30163385

**Published:** 2025-08-14

**Authors:** Youngwoo Jin, Hye Jin Kim, Kye Jung Shin, Khin Myo Htwe, Kee Dong Yoon

**Affiliations:** 1 College of Pharmacy and Integrated Research Institute of Pharmaceutical Science, The Catholic University of Korea, Bucheon 14662, Republic of Korea; wlsduddnpjk@naver.com (Y.J.); kkhj980316@catholic.ac.kr (H.J.K.); kyejung@catholic.ac.kr (K.J.S.); 2 Popa Mountain National Park, Forest Department, Kyaukpadaung 05241, Mandalay, Myanmar; khinmyohtwe007@gmail.com

**Keywords:** computational analysis, dihydrobenzofuran, DP4+ probability, electronic circular dichroism, flavonoid glycoside, *Myrsine seguinii*, Primulaceae

## Abstract

In this study, we aimed to determine the chemical constituents of *M. seguinii*, which led to the isolation and identification of 26 compounds. Three new prenylated dihydrobenzofurans [myrsinoic acids I (**1**), J (**2**), and K (**3**)] and a new flavonoid glycoside, mearnsetin 3-*O*-*α*-L-arabinopyranoside (**4**), were discovered, and the absolute configuration of the known compound, myrsinoic acid B (**5**), was re-established. To ensure the structural accuracy of these compounds, comprehensive spectroscopic analyses were performed, including one- and two-dimensional nuclear magnetic resonance spectroscopy, mass spectrometry, and circular dichroism spectroscopy. In addition, computational analysis methods such as density functional theory (DFT)-based Electronic Circular Dichroism (ECD) simulations and Gauge-Including Atomic Orbitals (GIAOs) ^1^H and ^13^C NMR chemical shift calculations with DP4+ probability analysis were utilised to further support the structural assignments.

## 1. Introduction

*Myrsine seguinii* H. Lév., a plant belonging to the Primulaceae family, is an evergreen tree distributed in tropical and subtropical regions, including Africa, Australia, China, Indochina, Myanmar, Taiwan, and Vietnam. It grows naturally as far north as Chiba Prefecture, Japan [1,2]. Plants belonging to the genus *Myrsine*, which comprises approximately 300 species, have traditionally been regarded as possessing various pharmacological benefits and have been used to treat inflammatory and infectious diseases such as influenza, snake bites, urinary tract infections, toothache, and headache in many countries [2,3,4]. Several studies have provided pharmacological evidence for the traditional use of *M. seguinii* and demonstrated its anti-inflammatory and neuroprotective activities. In terms of the chemical constituents of *M. seguinii*, previous studies have revealed the presence of key bioactive constituents such as flavonoids, terpenes, hydroquinone glycosides, and prenylated dihydrobenzofurans [4]. Among the reported constituents of *Myrsine* species, prenylated dihydrobenzofurans are characteristically found; this class of compounds is termed myrsinoic acids and exhibits structural diversity through two main structural motifs: (i) the attachment of isoprene chains as side chains to *p*-hydroxybenzoic acid (e.g., myrsicorianol and myrsinoic acid A and E), or (ii) the transformation of isoprene and *p*-hydroxybenzoic acid into a dihydrobenzofuran scaffold (e.g., myrsinoic acid B, C, F, G, and H) [4,5]. These dihydrobenzofuran-type myrsinoic acids possess several stereocentres that contribute to their increased structural diversity and complexity. Notably, the absolute configurations of some myrsinoic acids have been reported incorrectly or remain undetermined [5].

This study aimed to elucidate the chemical constituents of *M. seguinii* by employing computational calculation methods to enhance the structural accuracy and confirm the absolute configuration of the isolated compounds. The stereocentres of the isolated compounds were elucidated by quantum chemical calculations, including Electronic Circular Dichroism (ECD) to determine the absolute configuration by comparing calculated and experimental spectra, and nuclear magnetic resonance (NMR) calculations with DP4+ probability analysis to distinguish the most plausible isomer among possible candidates [6,7]. Furthermore, spectroscopy and Nuclear Overhauser Effect spectroscopy (NOESY) correlation analyses were simultaneously conducted to enhance the structural reliability of the compounds.

## 2. Results and Discussion

**Figure 1:**
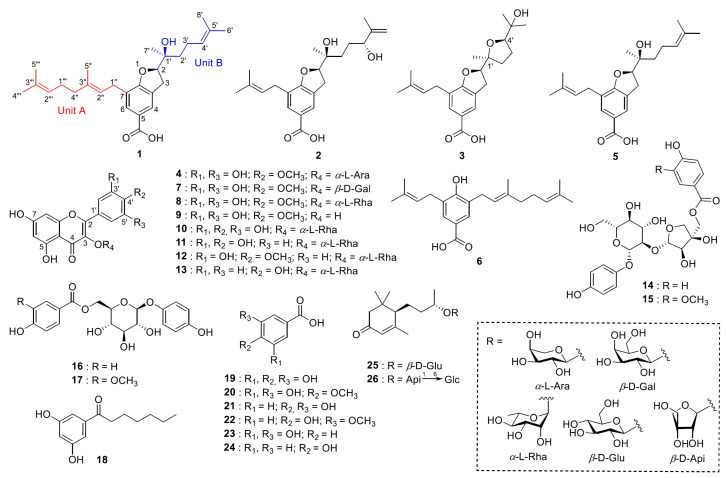
Chemical structures of compounds **1**–**26** isolated from *Myrsine seguinii*.

Chemical analysis of the methanol extract of the aerial parts of *M. seguinii* led to the isolation and structural elucidation of three new terpeno-benzoic acid derivatives (**1**–**3**), a new flavonoid glycoside (**4**), and 22 other known compounds. The known compounds were determined as myrsinoic acid B (**5**), myrsinoic acid A (**6**) [8], myricetin 4′-methyl ether 3-*O*-*b*-D-galactopyranoside (**7**) [9], mearnsitrin (**8**) [10], mearnsetin (**9**) [10], myricitrin (**10**) [10], quercitrin (**11**) [2], tamarixetin 3-*O*-*a*-L-rhamnoside (**12**) [11], kaempferol 3-*O*-*a*-L-rhamnoside (**13**) [12], seguinoside D (**14**) [13], seguinoside E (**15**) [13], breynioside A (**16**) [14], 6′-*O*-vanilloylarbutin (**17**) [15], vanillic acid 4-*O*-*b*-D-glucoside (**18**) [16], gallic acid (**19**) [17], 4-*O*-methylgallic acid (**20**) [18], protocatechuic acid (**21**) [19], vanillic acid (**22**) [19], resorcylic acid (**23**) [20], 4-hydroxy-benzoic acid (**24**) [21], (6*R*,9*S*)-blumenol *C* glucoside (**25**) [22], and byzantionoside B 6′-*O*-*b*-D-apiofuranoside (**26**) [23] (Figure 1; SI Figure S1.1). For myrsinoic acid B (**5**), the previously assigned absolute configuration was incorrect and has been re-established in this paper.

Compound **1** was a yellow amorphous solid, and its molecular formula was determined to be C_27_H_38_O_4_ by Electrospray Ionisation Time-of-Flight Mass Spectrometry (ESI-Q-TOF-MS), with a positive-ion peak at *m*/*z* 427.2818 [M + H]^+^. The ^1^H and ^13^C NMR spectra exhibited signals for a tetrasubstituted benzene group [*δ*_H_ 7.74 (2H, s, H-4, and H-6)/*δ*_C_ 127.37 (C-3a), 125.22 (C-4), 121.94 (C-5), 131.66 (C-6), 123.37 (C-7), and 162.64 (C-7a)], an oxygenated methine [*δ*_H_ 4.72 (1H, t, *J* = 9.0 Hz, H-2)/*δ*_C_ 89.77 (C-2)], a methylene [*δ*_H_ 3.19 (2H, m, H-3)/*δ*_C_ 30.11 (C-3)], and a carboxylic acid [*δ*_C_ 171.96 (COOH)] (Table 1).

**Figure 2:**
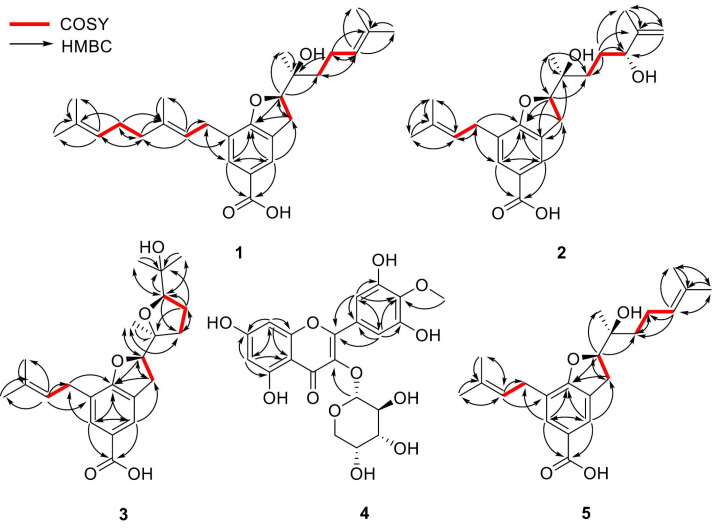
^1^H–^1^H COSY (bold lines) and HMBC (arrows) correlations of **1**–**5.**

The data indicate that **1** possesses a 2,3-dihydrobenzofuran-5-carboxylic acid skeleton, which is corroborated by the heteronuclear multiple-bond correlation (HMBC) (Figure 2) between H-2 and C-7a. Further analysis of the remaining signals of **1** revealed the presence of two distinct side chains: units A and B. The 1D NMR (^1^H and ^13^C NMR spectra) signals of unit A—*δ*_H_ 1.57 (3H, s, H-5‴), 1.65 (3H, s, H-4‴), 1.70 (3H, s, H-5”), 2.01 (2H, m, H-4”), 2.08 (2H, m, H-1‴), 3.28 (2H, m, H-1”), 5.09 (1H, t, *J* = 7.0 Hz, H-2‴), and 5.28 (1H, t, *J* = 7.3 Hz, H-2”)/*δ*_C_ 16.43 (C-5″), 17.89 (C-5‴), 25.87 (C-4‴), 26.87 (C-1‴), 28.38 (C-1″), 39.94 (C-4″), 121.37 (C-2″), 124.34 (C-2‴), 131.75 (C-3‴), and 137.07 (C-3″)—are consistent with a geranyl group. Unit B was identified as a 1′-hydroxy-1′,5′-dimethylhex-4′-enyl moiety, inferred from the following NMR data: *δ*_H_ 1.28 (3H, s, H-7′), 1.51 (2H, m, H-2′), 1.61 (3H, s, H-8′), 1.67 (3H, s, H-6′), 2.11 (2H, m, H-3′), and 5.10 (1H, t, *J* = 7.0 Hz, H-4′)/*δ*_C_ 17.91 (C-8′), 22.18 (C-3′), 22.89 (C-7′), 25.90 (C-6′), 37.32 (C-2′), 73.96 (C-1′), 124.22 (C-4′), and 132.46 (C-5′). These assignments are further substantiated by 2D NMR data, including ^1^H-^1^H correlated spectroscopy (COSY), correlations [unit A—H-1″/H-2″, H-4″/H-1‴, and H-1‴/H-2‴; unit B—H-2′/H-3′ and H-3′/H-4′] and HMBCs [unit A—H-2″/C-1″, C-4″, C-5″, H-4″/C-3″, C-1‴, C-2‴, H-5″/C-3″, H-1‴/C-3‴, H-2‴/C-4‴, C-5‴, H-4‴, H-5‴/C-3‴; unit B—H-3′/C-1′, C-4′, C-5′, H-4′/C-2′, C-6′, C-8′, H-6′, H-8′/C-5′, H-7′/C-1′] (Figure 2).

**Figure 3:**
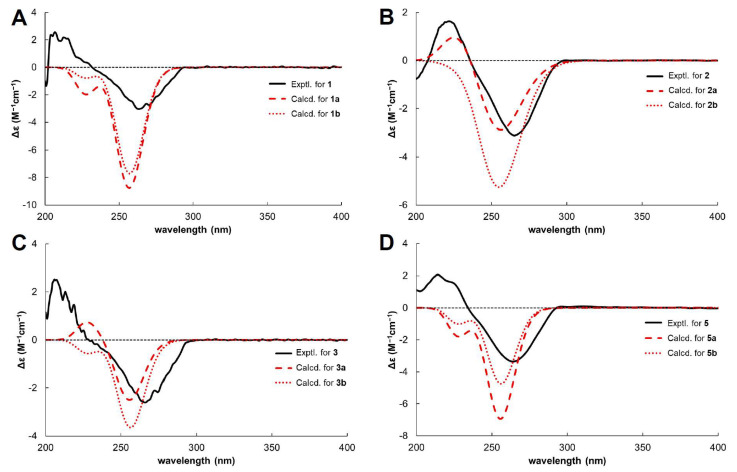
Experimental and calculated ECD spectra of **1** (**A**), **2** (**B**), **3** (**C**) and **5** (**D**).

Additionally, key HMBCs of H-1″ with C-6 and H-2 with C-2′ and C-7′ revealed that A and B units are substituted at C-7 and C-2, respectively, of the 2,3-dihydrobenzofuran-5-carboxylic acid core. A planar structure of **1** was elucidated, as shown in Figure 1. Comparison of the NMR data with those of myrosinoic acid B (**5**) revealed that they possess an identical 2,3-dihydrobenzofuran-5-carboxylic acid scaffold. The only difference is that the 3,3-dimethylallyl moiety in **5** is replaced by a geranyl group corresponding to unit A in **1**. Through HMBC peaks at *δ*_H_ 2.08 (H-1‴)/*δ*_C_ 137.07 (C-3″) and 39.94 (C-4″), attachment of an additional 3,3-dimethylallyl moiety at the C-4″ position was confirmed. Compound **1** has chiral centres at C-2 and C-1′. The absolute configuration of C-2 was determined by comparing its circular dichroism (CD) spectrum (Figure 3) with that of the dihydrobenzofuran derivative [24]. The experimental CD spectrum of **1** exhibited a positive Cotton effect (CE) at 206.6 nm and a negative CE at 263.2 nm, suggesting C-2’s absolute configuration as *R*. The NOESY correlation between H-2 and H-7′ was not suitable for determining the relative configuration due to the free rotation of the chain. Thus, **1** is proposed to have two possible isomers, (2*R*,1′*S*)-**1** (**1a**) and (2*R*,1′*R*)-**1** (**1b**). The C-1′ configuration was assigned based on a comparison of the carbon chemical shifts corresponding to C-2′ and C-7′ of **1** with those of the known compound bonannione B, sharing the identical dihydrobenzofuran with **1** [25]. The experimental chemical values of C-2′ (*δ*_C_ 37.32) and C-7′ (*δ*_C_ 22.89) in **1** closely match the experimental and calculated values of 2*R*,1′*S*-bonannione B (*δ*_C_ 36.6, 22.4 and *δ*_C_ 37.3, 23.1, respectively). In contrast, the calculated shifts for 2*R*,1′*R*-bonannione B are distinct (e.g., *δ*_C_ 40.6 and 19.7), as generated by density functional theory (DFT)–NMR analysis [25]. These results support the assignment of the 2*R*,1′*S* configuration for **1**. To confirm the accurate configurational assignment of **1**, the NMR chemical shifts of **1a** and **1b** were calculated using the Gauge-Including Atomic Orbital (GIAO) method and analysed using DP4+. The results revealed that the calculated ^1^H and ^13^C NMR data for **1a** are in good agreement with the experimental data, with a higher linear correlation coefficient (*R*^2^) and lower Mean Absolute Error (MAE) and Corrected Mean Absolute Error (CMAE) values than those of **1b**. Additionally, the DP4+ analysis indicated that **1a** is the most likely structure with a 100% probability (Table 2). Finally, because of the similar ECD patterns observed for **1a** and **1b**, it was not possible to obtain additional differentiation through ECD experiments (Figure 3). Based on the above evidence (Figures S1.1–S1.6 and Tables S1.1–S1.7), the absolute configuration of **1** was determined to be 2*R*,1′*S* and was named myrsinoic acid I [(*R*)-7-((*E*)-3,7-dimethylocta-2,6-dien-1-yl)-2-((*S*)-2-hydroxy-6-methylhept-5-en-2-yl)-2,3-dihydrobenzofuran-5-carboxylic acid].

Compound **2** was isolated as a white amorphous solid and was confirmed to have the molecular formula C_22_H_30_O_5_ based on the positive-ion peak at 375.2169 [M + H]^+^. Analyses of the ^1^H and ^13^C NMR spectra predict that the overall structure of **2** is similar to that of **5** (Table 1). Notable changes are observed in the ^1^H and ^13^C NMR spectra. The signals corresponding to the methyl protons and olefinic proton of the terminal 3,3-dimethylallyl group in unit B of **1** are replaced by signals for a methylene group [*δ*_H_ 4.96 (1H, s, H-6′α), 4.85 (1H, s, H-6′β)/*δ*_C_ 147.4 (C-5′) and 111.4 (C-6′)] and an oxygenated methine proton [*δ*_H_ 4.10 (1H, dd, *J* = 7.3, 4.6 Hz, H-4′)/*δ*_C_ 76.0 (C-4′)], respectively. A comprehensive interpretation of the above 1D-NMR analysis (Table 1) reveals that the terminal 3,3-dimethylallyl moiety connected to C-2′ in **5** is substituted with a 3-methyl-3-buten-2-ol moiety owing to the transformation, as supported by the COSY correlations [H-2′/H-3′ and H-3′/H-4′] and HMBCs [H-3′/C-2′, C-4′, C-5′, C-8′, H-6′/C-4′, C-5′, C-8′, H-7′/C-1′, C-2′, H-8′/C-4′, C-5′] (Figure 2). Further additional HMBC analysis [H-3/C-1′ and H-2/C-2′] reveals the attachment of the 3-methyl-3-buten-2-ol moiety group to position C-2′ (Figure 2). Based on the above evidence, the planar structure of **2** was established as depicted in Figure 1.

In compound **2**, three stereocentres are present at C-2, C-1′, and C-4′. As previously mentioned for **1**, the stereochemical assignments at C-2 and C-1′ were determined using the same analytical procedure. The CD spectrum indicates that the absolute configuration of C-2 is *R*, as evidenced by the positive CE at 222 nm and the negative CE at 265 nm. The configuration of C-1′ is assigned as *S* according to the experimental carbon chemical shifts at C-2′ and C-7′ at *δ*_C_ 33.13 and 22.83 ppm (Table 1). To determine the stereochemistry of C-4′, NOESY cross peaks were considered; however, the conformational flexibility of the linear side chain makes a definitive assignment difficult [26]. Accordingly, the absolute configuration of C-4′ was reconfirmed via computational analysis. GIAO NMR chemical shift calculations were conducted for two possible isomers, (2*R*,1′*S*,4′*S*)-**2** (**2a**) and (2*R*,1′*S*,4′*R*)-**2** (**2b**), followed by DP4+ probability analysis. The results demonstrate that the parameters from calculated NMR data exhibit a better agreement with the experimental ^1^H NMR spectrum for **2a**, whereas the ^13^C NMR data show higher consistency with **2b** (Table 2). This discrepancy indicates that the NMR chemical shift calculations are inadequate for clearly distinguishing between the two isomers. Therefore, instead of comparing all chemical shifts, we evaluated the ^1^H NMR chemical shifts in H-2 and H-7′, where significant differences are observed between the two stereoisomers. As shown in Table 3, the calculated ^1^H NMR chemical shifts for **2a** exhibit greater consistency with the experimental data. These results are further supported by DP4+ analysis (Table 2). This was further corroborated by comparing the experimental and calculated ECD spectra (Figure 3). Finally, the absolute configuration of **2** was established as 2*R*,1′*S*,4′*S* and named myrsinoic acid J [(*R*)-2-((2*S*,5*R*)-2,5-dihydroxy-6-methylhept-6-en-2-yl)-7-(3-methylbut-2-en-1-yl)-2,3-dihydrobenzofuran-5-carboxylic acid] (Figures S2.1–S2.6 and Tables S2.1–S2.7).

The molecular formula of **3**, a yellow, amorphous solid, was estimated to be C_22_H_30_O_5_ based on the positive-ion ESI-Q-TOF-MS spectrum, which displayed an ion peak at *m*/*z* 375.2176 [M + H]^+^. Compounds **2** and **3** had identical chemical formulas and similar ^1^H and ^13^C NMR spectra (Table 1). A notable difference is the chemical shifts corresponding to unit B, attributable to the presence of two methylene groups [δ_H_ 2.02 (1H, dt, *J* = 12.1, 9.0 Hz, H-2′α), 1.74 (1H, m, H-2′β), and 1.88 (2H, m, H-3′)/δ_C_ 33.76 (C-2′) and 26.61 (C-3′)], an oxygenated methine [δ_H_ 3.91 (2H, t, *J* = 7.6 Hz, H-4′)/δ_C_ 87.45 (C-4′)], three methyl groups [δ_H_ 1.22 (3H, s, H-6′), 1.19 (3H, s, H-7′), 1.13 (3H, s, H-8′)/δ_C_ 27.78 (C-6′), 22.82 (C-7′), 24.23 (C-8′)], and two oxygenated sp^3^ carbons [δ_C_ 84.68 (C-1′) and 71.03 (C-5′)]. Considering the 1D (Table 1) and HSQC data (Figure 2), and the molecular formula confirmed by the ESI-Q-TOF-MS spectrum of **3**, it can be inferred that the terminal 3-methyl-3-buten-2-ol group in **2** is replaced by a tetrahydrofuran structure, as shown in Figure 1. This conclusion was supported by ^1^H–^1^H COSY correlations [H-2′/H-3′ and H-3′/H-4′] and HMBCs [H-2′/C-3′, H-3′/C-1′, C-4′, H-4′/C-2′, C-5′, C-6′, C-8′, H-6′, H-8′/C-5′, H-7′/C-1′] (Figure 2).

**Figure 4:**
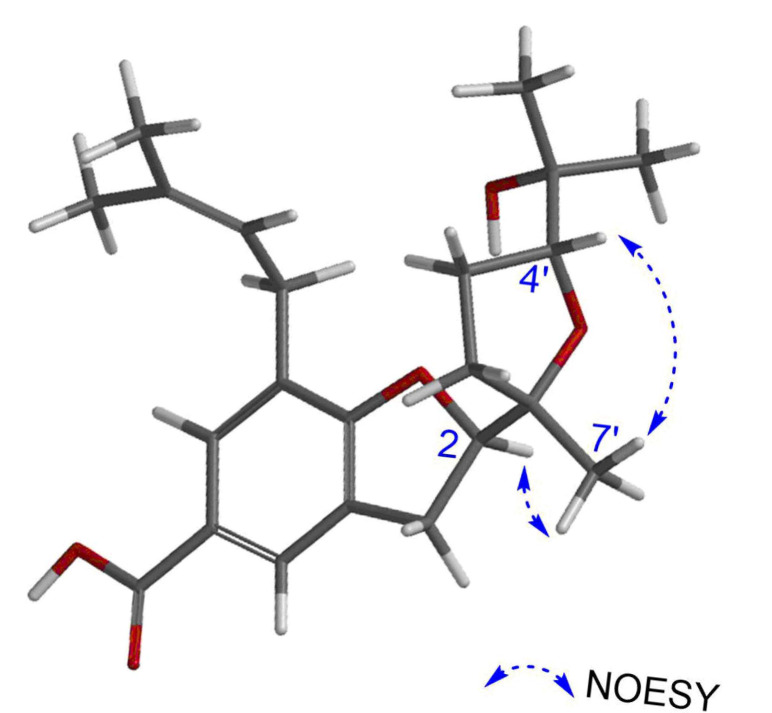
Key NOESY (arrows) correlations of **3**.

Like **2**, compound **3** possesses three stereocentres at C-2, C-1′, and C-4′. The configurations at C-2 and C-1′ were determined to be *R* and *S*, respectively, based on the positive CE at 205.7 nm and the negative CE at 266.2 nm in the CD spectrum (Figure 3), NOESY correlation peaks [*δ*_H_ 4.83 (1H, m, H-2)/*δ*_H_ 1.19 (3H, s, H-7′)] (Figure 4), and the chemical shifts at C-2′ and C-7′ (*δ*_C_ 33.76 and 22.82 ppm, respectively) (Table 1). Additionally, the configuration of C-4′ was provisionally assigned as *R*, supported by the NOESY correlation peak [*δ*_H_ 3.91 (1H, t, *J* = 7.6 Hz, H-4′)/1.19 (3H, s, H-7′)]. To clarify the stereochemistry of **3**, NMR calculations and DP4+ analysis were performed on the two isomers, (2*R*,1′*S*,4′*R*)-**3** (**3a**) and (2*R*,1′*S*,4′*S*)-**3** (**3b**). The results showed that **3a** matches the experimental data more closely than **3b**, with higher *R*^2^ and lower MAE and CMAE values. Moreover, the DP4+ calculations also afford a probability of 100% (Table 2). Based on ECD calculations (Figure 3), the absolute configuration of **3** was identified as 2*R*,1′*S*,4′*R* and called myrsinoic acid K [(*R*)-2-((2*S*,5*R*)-5-(2-hydroxypropan-2-yl)-2-methyltetrahydrofuran-2-yl)-7-(3-methylbut-2-en-1-yl)-2,3-dihydrobenzofuran-5-carboxylic acid] (Figures S3.1–3.6 and Tables S3.1–S3.7).

Compound **4** was isolated as a yellow amorphous powder, and its molecular formula was determined to be C_21_H_20_O_12_ based on the positive ESI-Q-TOF-MS spectrum, which exhibited an ion peak at *m*/*z* 487.0852 [M + Na]^+^. The presence of distinctive peaks at 209.4, 264.2, and 349.8 nm in the UV spectrum indicates that the compound is a flavonoid (Figure S4.4). In the ^1^H-NMR spectrum, the typical signals corresponding to the mearnsetin (myricetin 4′-methyl ether) scaffold are displayed at *δ*_H_ 7.11 (2H, s, H-2′, 6′), 6.38 (1H, d, *J* = 2.0 Hz, H-8), 6.21 (1H, d, *J* = 2.0 Hz, H-6), and 3.75 (3H, s, 4′-OCH_3_) (Table 4) [27]. Additionally, an anomeric proton signal arising from a sugar moiety occurs at *δ*_H_ 5.22 (1H, d, *J* = 5.5 Hz, H-1″), which suggests that **4** is a mearnsetin glycoside.

The ^13^C-NMR spectrum exhibits sixteen carbon signals corresponding to mearnsetin and another five signals for a sugar unit at *δ*_C_ 101.32 (C-1″), 71.46 (C-2″), 70.33 (C-3″), 65.94 (C-4″), and 64.26 (C-5″). From the anomeric proton signal [*δ*_H_ 5.22 (1H, d, *J* = 5.5 Hz, H-1″)] and carbon resonances, the sugar unit was determined to be *α*-arabinopyranoside. The presence of the L-arabinose unit was confirmed by acidic hydrolysis analysis of **4** (Figure S4.4). Finally, the linkage between mearnsetin and the sugar moiety was determined through the HMBC spectrum, showing a correlation peak at *δ*_H_ 5.22 (H-1″)/*δ*_C_ 134.18 (C-3) (Figure 2). Therefore, the chemical structure of **4** was elucidated to be mearnsetin 3-*O*-*α*-L-arabinopyranoside (Figures S4.1–S4.4).

Compound **5** was isolated as a yellow amorphous solid, and its molecular formula was deduced to be C_22_H_30_O_4_ based on the ion peak at *m*/*z* 359.2221 [M + H]^+^ in the ESI-Q-TOF-MS spectrum. The ^1^H and ^13^C NMR spectra show similar patterns to those of **1;** the difference observed in the ^1^H NMR spectrum is the replacement of the geranyl group (unit A) of **1** with a 3,3-dimethylallyl moiety (Table 1). These detailed structural features are supported by 2D NMR and MS data (Figure 2). By comparison with the reported literature data [28], the planar structure **5** was identified as the known compound, myrosinoic acid B (Figure 1).

Although myrsinoic acid B has chiral centres at the C-2 and C-1′ positions, some studies [28,29,30] have presented only a planar structure, lacking stereochemical information. Amaro–Luis et al. assigned the absolute configuration as 2*R*,1′*R*; however, a review of their publication and the related literature provided no supporting evidence for this assignment [4,31]. Compounds **1** and **5** possess an identical 2,3-dihydrobenzofuran-5-carboxylic acid core and side chain unit B; therefore, the stereochemistry of **5** was determined using an approach identical to that of **1**. In the CD spectrum (Figure 3), **5** shows a CE pattern (positive at 214 nm and negative at 264 nm) similar to that of **1**, suggesting that the configuration of **5** could be either (2*R*,1′*S*)-**5** (**5a**) or (2*R*,1′*R*)-**5** (**5b**). Based on the chemical shifts at C-2′ (*δ*_C_ 37.10) and C-7′ (*δ*_C_ 22.84), the absolute configuration of **5** was assigned as **5a**. Similar to **1**, the absolute configuration of **5** could not be clearly assigned because of the similarity between the calculated ECD spectra of **5a** and **5b** (Figure 3). Furthermore, the configuration of **5** was confirmed by GIAO NMR chemical shift calculations and DP4+ probability analyses of the two isomers. As shown in Table 2, the parameters obtained from the ^13^C NMR data were highly consistent with the calculated results for **5a**; however, the ^1^H NMR data indicated that **5b** exhibited higher *R*^2^ and MAE values, leading to ambiguity in the interpretation. These results revealed that the NMR calculation data alone were insufficient to unambiguously determine the structure of **5**. Therefore, a DP4+ probability analysis was performed, and **5a** was identified as the correct configuration with a probability of 100 %. These results support the assignment of the 2*R*,1′*S* configuration for **5** (Figures S5.1–S5.6 and Tables S4.1–S4.7). Therefore, the structure of myrsinoic acid B is re-established as (*R*)-2-((*S*)-2-hydroxy-6-methylhept-5-en-2-yl)-7-(3-methylbut-2-en-1-yl)-2,3-dihydrobenzofuran-5-carboxylic acid.

## 3. Materials and Methods

### 3.1. General

HPLC analyses were carried out on a Waters Alliance HPLC system (2695 separation module, Milford, MA, USA) equipped with a Luna C18 column (4.6 × 250 mm I.D., 5 mm; Phenomenex, Torrance, CA, USA). A Gilson preparative HPLC system (Middleton, WI, USA) consisting of a binary pump, manual injector, and UV/VIS detector was employed to separate the compounds. The preparative HPLC column was a Luna C18(2) column (21.2 × 250 mm, 5 mm; Torrance, CA, USA). The column chromatography (CC) was performed using silica gel 60 F-254 (40–63 mm; Merck, Darmstadt, Germany), ZEOprep 90 C18 (40–63 mm; Zeochem, Uetikon, Switzerland), and Diaion HP-20 (Mitsubishi Chemical, Tokyo, Japan). The structures of the isolated compounds were elucidated using 1D- and 2D-NMR data acquired using an AVANCE 500 spectrometer (Bruker, Karlsruhe, Germany). ESI-Q-TOF-MS spectra were obtained using a 6460 Q-TOF-MS spectrometer (Agilent Technologies, Santa Clara, CA, USA). Optical rotation and CD data were recorded using a P-2000 polarimeter and a J-815 CD spectrophotometer, respectively (Jasco, Tokyo, Japan). Organic solvents for extraction and column chromatography were purchased from Daejung Chemical and Metals Co., Ltd. (Kyunggido, Republic of Korea), and HPLC-grade methanol and acetonitrile were obtained from J.T. Baker Chemical Co. (Phillipsburg, NJ, USA). Deionised water was obtained using a Milli-Q water purification system (Millipore, Burlington, MA, USA).

### 3.2. Plant Materials

Aerial parts of *Myrsine seguinii* were collected from Popa Mountain National Park, Mandalay, Myanmar, in August 2015 and identified by Khin Myo Htwe (staff officer, Popa Mountain National Park, Mandalay, Myanmar). The dried and powdered aerial parts of *M. seguinii* were stored in a freezer at −80 °C before use. A voucher specimen (#M-MS-20150811) was deposited in the herbarium of the College of Pharmacy at the Catholic University of Korea.

### 3.3. Extraction and Isolation

Aerial parts of dried and powdered *M. seguinii* (1.2 kg) were extracted using methanol (MeOH) at room temperature (6 L × 90 min × 3 times) in an ultrasonic bath (Bransonic, Model 5510, 42 kHz, 185 W), and the solvent was evaporated under reduced pressure at 40 °C. The MeOH extract (104.88 g) was suspended in 90% aqueous MeOH (1.5 L) and partitioned with *n*-hexane (*n*-Hex, 1.5 L × 3 times) to give an *n*-Hex-soluble extract (9.26 g). Subsequently, the 90% MeOH layer was evaporated under reduced pressure and resuspended in water (1.5 L), followed by consecutive partitioning using organic solvents to yield ethyl acetate (EtOAc, 32.89 g)- and *n*-butanol (*n*-BuOH, 13.56 g)-soluble extracts.

The EtOAc-soluble extract (32.89 g) was subjected to silica gel column chromatography (CC) [*n*-Hex:EtOAc (5:1→2:1, *v*/*v*), CH_2_Cl_2_:MeOH (10:1→5:1→2:1, *v*/*v*), and MeOH] to afford 20 sub-fractions (E1–E20). Fraction E5 (234.3 mg) was purified by reverse-phase (RP) HPLC and eluted with acetonitrile (MeCN)–water (H_2_O) (85:15, *v*/*v*) to afford **6** (myrsinoic acid A, 10 mg, *t*_R_ = 40.8 min). Fraction E7 (2.5 g) was subjected to RP CC (MeOH-H_2_O step gradient elution from 5:5 to 9:1, *v*/*v*) to yield nine sub-fractions (E7.1–E7.9), and **5** (myrsinoic acid B, 20.9 mg, *t*_R_ = 38.9 min) and **18** (1-(3,5-dihydroxyphenyl)heptan-1-one, 7.0 mg, *t*_R_ = 31.8 min) were obtained from fractions E7.8 (1.17 g) and E7.3 (7.7 mg), respectively. Fraction E7.9 (73 mg) was purified by RP-HPLC using MeCN-H_2_O (90:10, *v*/*v*) to obtain **1** (myrsinoic acid I, 15.8 mg, *t*_R_ = 45.3 min). Compound **2** (myrsinoic acid J, 2.9 mg, *t*_R_ = 31.4 min) was isolated by RP-HPLC [MeCN-H_2_O (90:10, *v*/*v*)] from fraction E7.4 (24.8 mg). Fraction E9 (500 mg) was subjected to RP medium-pressure liquid chromatography (MPLC) (MeOH-H_2_O step gradient elution from 3:7 to 8:2, *v*/*v*) to obtain 16 sub-fractions (E9.1–E9.16). Fraction E9.1 (45.3 mg) was resolved by RP-HPLC eluted with aqueous MeCN (17%) to yield **22** (vanillic acid, 15 mg, *t*_R_ = 16.9 min) and **24** (4-hydroxy-benzoic acid, 5.3 mg, *t*_R_ = 16.3 min). Fraction E9.15 (42.6 mg) was subjected to RP-HPLC using MeCN-H_2_O (55:45, *v*/*v*) to yield **3** (myrsinoic acid K, 20.8 mg, *t*_R_ = 33.8 min). Fraction E11 (1.81 g) was subjected to RP-MPLC (MeOH-H_2_O, 2:8 to 8:2, *v*/*v*) to yield 13 sub-fractions (E11.1–E11.13). Fraction E11.1 (55.3 mg) was subjected to RP-HPLC using 12% aqueous MeCN to afford compounds **20** (4-*O*-methylgallic acid, 10.5 mg, *t*_R_ = 14.1 min), **21** (protocatechuic acid, 6.6 mg, *t*_R_ = 13.0 min), and **23** (resorcylic acid, 1.0 mg, *t*_R_ = 13.1 min). Fraction E11.5 (48 mg) was separated by RP-HPLC using 35% aqueous MeCN to yield compound **9** (mearnsetin, 7.7 mg, *t*_R_ = 22.4 min). Fraction E12 (2.69 g) was separated using silica gel CC with CH_2_Cl_2_-MeOH (12.5:1→10:1→7.5:1, *v*/*v*) to afford six sub-fractions (E12.1–E12.6). Fraction E12.5 (316 mg) was subjected to RP-HPLC with MeCN-H_2_O (21:79, *v*/*v*) to yield **4** (mearnsetin 3-*O*-*a*-L-arabinopyranoside, 5.3 mg, *t*_R_ = 20.3 min), **12** (tamarixetin 3-*O*-*α*-L-rhamnoside, 10.7 mg, *t*_R_ = 22.1 min), **13** (kaempferol 3-*O*-*α*-L-rhamnoside, 1.6 mg, *t*_R_ = 22.0 min), and **19** (gallic acid, 4.9 mg, *t*_R_ = 7.8 min). Fraction E12.6 (38.5 mg) was subjected to RP-HPLC with MeCN (16%) in H_2_O to give **16** (breynioside A, 3.2 mg, *t*_R_ = 18.2 min) and **17** (6′-*O*-vanilloylarbutin, 10.3 mg, *t*_R_ = 18.3 min). Fraction E13 (2 g) underwent RP-MPLC using MeOH (30%) in H_2_O, giving eight sub-fractions (E13.1–E13.8), and **8** (mearnsitrin, 249.8 mg, *t*_R_ = 20.2 min) was obtained from Fraction E13.3. Compound **10** (myricitrin, 10.4 mg, *t*_R_ = 19.1 min) was purified from fraction E17 (147.9 mg) by RP-HPLC using MeCN-H_2_O (30:70, *v*/*v*). Fraction E14 (1.36 g) was subjected to RP-MPLC using MeOH-H_2_O (step gradient elution from 1.5:8.5 to 4:6, *v*/*v*), resulting in the generation of nine sub-fractions (E14.1–E14.9), and **14** (seguinoside D, 4.7 mg, *t*_R_ = 17.3 min) and **15** (seguinoside E, 6.0 mg, *t*_R_ = 17.1 min) were generated from fractions E14.1 and E14.2, respectively. Fraction E14.6 (98.5 mg) was purified by RP-HPLC eluted with 50% aqueous MeOH to yield **7** (myricetin 4′-methyl ether 3-*O*-*β*-D-galactopyranoside, 2.9 mg, *t*_R_ = 19.2 min) and **11** (quercitrin, 13.5 mg, *t*_R_ = 20.9 min).

The *n*-BuOH-soluble extract (6.5 g) was subjected to Diaion HP-20 CC eluted with H_2_O, 50% aqueous MeOH, and MeOH to afford BW (1.5 g), B50M (1.5 g), and BM (1.2 g) fractions, respectively. The BM fraction was separated by silica gel CC with CH_2_Cl_2_-MeOH (step gradient elution from 40:1 to 5:1, *v*/*v*) to yield 14 sub-fractions (BM1-BM14). Fraction BM5 (21.9 mg) was purified using RP-HPLC [MeCN-H_2_O (25:75, *v*/*v*)] and subsequently re-chromatographed using RP-HPLC with MeOH (40%) in H_2_O, resulting in the isolation of **25** [(6*R*,9*S*)-blumenol C glucoside, 0.9 mg, *t*_R_ = 20.0 min]. Fraction BM8 (86 mg) was purified by RP-HPLC eluted with MeCN-H_2_O (20:80, *v*/*v*) to afford **26** (byzantionoside B 6′-*O*-*β*-D-apiofuranoside 4.6 mg, *t*_R_ = 19.1 min).

Myrsinoic acid I (**1**): C_27_H_38_O_4_; white amorphous solid; ${\left[\alpha \right]}_{D}^{25}$ = −62.57° (*c* 0.02, MeOH); ESI-Q-TOF-MS: *m*/*z* 427.2818 [M + H]^+^ (calcd for C_27_H_38_O_4_, 427.2848); UV (*c* 0.0033, MeOH) λ_max_ (log ε) 206.6 (4.42), 264.8 (4.07) nm; ^1^H-NMR (500 MHz, CDCl_3_): Table 1; ^13^C-NMR (125 MHz, CDCl_3_): Table 1.

Myrsinoic acid J (**2**): C_22_H_30_O_5_; white amorphous solid; ${\left[\alpha \right]}_{D}^{25}$ = −44.17° (*c* 0.03, MeOH); ESI-Q-TOF-MS: *m*/*z* 375.2169 [M + H]^+^ (calcd for C_22_H_30_O_5_, 375.2171); UV (*c* 0.0033, MeOH) λ_max_ (log ε) 208.4 (4.41), 264.8 (4.18) nm; ^1^H-NMR (500 MHz, CDCl_3_): Table 1; ^13^C-NMR (125 MHz, CDCl_3_): Table 1.

Myrsinoic acid K (**3**): C_22_H_30_O_5_; yellow amorphous solid; ${\left[\alpha \right]}_{D}^{25}\;$= −56.38° (*c* 0.37, MeOH); ESI-Q-TOF-MS: *m*/*z* 375.2176 [M + H]^+^ (calcd for C_22_H_30_O_5_, 375.2171); UV (*c* 0.0033, MeOH) λ_max_ (log ε) 207.0 (4.36), 266.4 (4.10) nm; ^1^H-NMR (500 MHz, CDCl_3_): Table 1; ^13^C-NMR (125 MHz, CDCl_3_): Table 1.

Mearnsetin 3-*O*-*a*-L-arabinopyranoside (**4**): C_21_H_20_O_12_; yellow amorphous powder; ${\left[\alpha \right]}_{D}^{25}\;$= −77.37° (*c* 0.33, MeOH); ESI-Q-TOF-MS: *m*/*z* 487.0852 [M + Na]^+^ (calcd for C_21_H_20_O_12_, 487.0852); UV (c 0.0022, MeOH) λ_max_ (log ε) 209.4 (4.61), 264.2 (4.23), 349.8 (4.09) nm; ^1^H-NMR (500 MHz, DMSO-*d*_6_): Table 4; ^13^C-NMR (125 MHz, DMSO-*d*_6_): Table 4.

Myrsinoic acid B (**5**): C_22_H_30_O_4_; yellow amorphous solid; ${\left[\alpha \right]}_{D}^{25}$ = −42.81° (*c* 0.524, MeOH); ESI-Q-TOF-MS: *m*/*z* 359.2221 [M + H]^+^ (calcd for C_22_H_30_O_4_, 359.2222); ^1^H-NMR (500 MHz, CDCl_3_): Table 1; ^13^C-NMR (125 MHz, CDCl_3_): Table 1.

### 3.4. Sugar Analysis

Compound **4** (1 mg) was subjected to acid hydrolysis in 1 M HCl (1.0 mL) at 80 °C for 2 h. Subsequently, the aqueous layer, presumed to contain sugar, was analysed by TLC to determine its composition. The TLC plate was developed in chloroform/methanol/water (30:20:4, *v*/*v*/*v*), and subsequently visualised using an aniline hydrogen phthalate reagent. The Rf value was identical to that of the standard L-arabinose, confirming that the sugar moiety of **4** corresponds to L-arabinose.

### 3.5. Computational Analysis

Conformational searches were performed using Merck Molecular Force Field (MMFF) 94 in Spartan’14 software (Spartan Software, San Francisco, CA, USA). All conformers within 21 kJ/mol of the lowest energy minimum were subjected to geometry optimisation and frequency calculations using density functional theory (DFT) at the B3LYP/6-31G(d) level of theory in the gas phase. NMR calculations were performed using the GIAO method at the B3LYP/6-31 + G(d,p) level in chloroform using Gaussian 09 software (Gaussian Inc., Wallingford, CT, USA). The shielding constants of tetramethylsilane (TMS) were obtained using the same level of theory. DP4+ probability analysis of each possible candidate was performed using an Excel spreadsheet provided by Grimblat et al. [6]. The ECD calculations of all the optimised conformers were conducted using the time-dependent DFT (TDDFT) method at the CAM-B3LYP/6-31 + G(d,p) level using the CPCM model.

## 4. Conclusions

The current study successfully identified three novel terpeno-benzoic acid derivatives (myrsinoic acids I, J, and K) and a new flavonoid glycoside (mearnsetin 3-*O*-*α*-L-arabinopyranoside) from the aerial parts of *M. seguinii*. The absolute configuration of the known compound myrsinoic acid B was re-established through advanced spectroscopic and computational analyses. These findings of the current study contribute important data on the chemical diversity of *M. seguinii* and provide a compelling example of how to establish the structures of complex isomers using diverse computational techniques.

## Figures and Tables

**Table 1:** ^1^H (multiplicity, *J* in Hz, 500 MHz) and ^13^C (125 MHz) NMR data for **1**–**3** and **5** (CDCl_3_).

#	1		2		3		5	
	*δ*_H_ (Mult, *J* in Hz)	*δ*_C_	*δ*_H_ (Mult, *J* in Hz)	*δ*_C_	*δ*_H_ (Mult, *J* in Hz)	*δ*_C_	*δ*_H_ (Mult, *J* in Hz)	*δ*_C_
2	4.72 (1H, t, 9.0)	89.77	4.73 (1H, t, 8.9)	89.57	4.83 (1H, m)	89.01	4.72 (1H, t, 8.7)	89.78
3	3.19 (2H, m)	30.11	3.19 (2H, m)	30.08	3.23 (1H, 9.4) 3.04 (1H, dd, 16.1, 7.8)	31.51	3.21 (2H, m) *	30.10
3a		127.37		127.41		127.10		127.38
4	7.74(1H, s)	125.22	7.72 (1H, s)	125.23	7.73 (1H, s)	125.14	7.73 (1H, s) **	125.22
5		121.94		121.50 *		121.80		121.93
6	7.74(1H, s)	131.66	7.72 (1H, s)	131.62	7.74 (1H, s)	131.73	7.73 (1H, s) **	131.62
7		123.37		123.34		123.38		123.34
7a		162.64		162.55		163.05		162.60
1′		73.96		73.53		84.68		73.98
2′	1.51 (2H, m)	37.32	1.60 (2H, m)	33.13	2.02 (1H, dt, 12.1, 9.0) 1.74 (1H, m)	33.76	1.51 (2H, m)	37.10
3′	2.11 (2H, m)	22.18	1.67 (2H, m)	28.45	1.88 (2H, m)	26.61	2.11 (2H, m)	22.16
4′	5.10 (1H, t, 7.0)	124.22	4.10 (1H, dd, 7.3, 4.6)	75.98	3.91 (1H, t, 7.6)	87.45	5.10 (1H, t, 7.0)	124.23
5′		132.46		147.43		71.03		132.43
6′	1.67(3H, s)	25.90 *	4.96 (1H, s) 4.85 (1H, s)	111.37	1.22 (3H, s)	27.78	1.67 (3H, s)	25.90
7′	1.28(3H, s)	22.89	1.26 (3H, s)	22.83	1.19 (3H, s)	22.82	1.28 (3H, s)	22.84
8′	1.61(3H, s)	17.91 **	1.72 (3H, s)	18.23	1.13 (3H, s)	24.23	1.61 (3H, s)	17.88
1″	3.28 (2H, m)	28.38	3.26 (2H, m)	28.45	3.27 (2H, t, 7.5)	28.45	3.21 (2H, m) *	28.49
2″	5.28 (1H, t, 7.3)	121.37	5.25 (1H, t, 7.4)	121.50 *	5.26 (1H, ddt, 7.4, 5.8, 1.5)	121.62	5.26 (1H, t, 7.4)	121.55
3″		137.07		133.45		133.32		133.40
4″	2.01 (2H, m)	39.94	1.72 (3H, s)	26.01	1.71 (3H, s)	26.00	1.72 (3H, s)	25.98
5″	1.70 (3H, s)	16.43	1.70 (3H, s)	18.07	1.70 (3H, s)	18.04	1.71 (3H, s)	18.06
1‴	2.08 (2H, m)	26.87						
2‴	5.09 (1H, t, 7.0)	124.34						
3‴		131.75						
4‴	1.65 (3H, s)	25.87 *						
5‴	1.57 (3H, s)	17.89 **						
COOH		171.96		171.43		172.14		172.05

* and **: these resonances overlapped.

**Table 2:** Analysis of parameters (*R*^2^, MAE, and CMAE) applied to calculated chemical shifts and DP4+ probability results for compounds **1**–**3** and **5**.

Compd.	Conformer	^1^H-NMR			^13^C-NMR			DP4+ (All)
		MAE	CMAE	*R* ^2^	MAE	CMAE	*R* ^2^	
**1**	**1** **a**	0.15	0.06	0.9995	2.44	1.78	0.9992	100%
	**1** **b**	0.19	0.09	0.9987	2.45	2.01	0.9989	0%
**2**	**2** **a**	0.21	0.07	0.9992	3.12	2.56	0.9980	96.89%
	**2** **b**	0.25	0.10	0.9980	2.75	2.29	0.9985	3.11%
**3**	**3** **a**	0.18	0.07	0.9990	2.35	1.68	0.9992	100%
	**3** **b**	0.18	0.08	0.9987	2.76	2.09	0.9986	0%
**5**	**5** **a**	0.19	0.07	0.9991	2.34	1.67	0.9992	100%
	**5** **b**	0.20	0.07	0.9993	2.43	1.94	0.9989	0%

**Table 3:** Comparison of experimental and calculated ^1^H-NMR (*δ* in ppm) for **2**.

No.	*δ*_H_ Exp.	*δ*_H_ Calc.	
	2 (In CDCl_3_)	2a	2b
2	4.73	4.87	5.29
7′	1.26	1.23	1.09

**Table 4:** ^1^H (multiplicity, *J* in Hz, 500 MHz) and ^13^C (125 MHz) NMR spectra of **4** (DMSO-*d*_6_).

#	*δ*_H_ (Mult, *J* in Hz)	*δ*_C_	#	*δ*_H_ (Mult, *J* in Hz)	*δ*_C_
2		155.44	1′		124.66
3		134.18	2′, 6′	7.11 (2H, s)	108.18
4		177.35	3′, 5′		150.11
5		121.9	4′		137.64
6	6.21 (1H, d, 2.0)	98.44	4′-OCH_3_	3.75 (3H, s)	59.49
7		164.032	1″	5.22 (1H, d, 5.5)	101.32
8	6.38 (1H, d, 2.0)	93.15	2″	3.49 (1H, d, 7.5, 2.9)	71.46
9		156.02	3″	3.73 (1H, m)	70.33
10		103.70	4″	3.64 (1H, d, 2.8)	65.94
			5″	3.61 (1H, d, 5.0)	64.26

## Data Availability

All relevant data are available in the article and its Supplementary Materials.

## Supplementary Materials

The following supporting information can be downloaded at https://www.mdpi.com/article/10.3390/molecules30163385/s1: SI 1.1. ^1^H (500 MHz) and ^13^C (125 MHz) NMR and MS data of **6**–**26**; Figure S1.1. ^1^H (500 MHz) and ^13^C NMR (125 MHz) spectra of compound **1** in CDCl_3_; Figure S1.2. ^1^H-^1^H COSY spectrum of compound **1**; Figure S1.3. HSQC spectrum of compound **1**; Figure S1.4. HMBC spectrum of compound **1**; Figure S1.5. NOESY spectrum of compound **1**; Figure S1.6. CD, MS, and UV spectra of compound **1**; Figure S2.1. ^1^H (500 MHz) and ^13^C NMR (125 MHz) spectra of compound **2** in CDCl_3_; Figure S2.2. ^1^H-^1^H COSY spectrum of compound **2**; Figure S2.3. HSQC spectrum of compound **2**; Figure S2.4. HMBC spectrum of compound **2**; Figure S2.5. NOESY spectrum of compound **2**; Figure S2.6. CD, MS, and UV spectra of compound **2**; Figure S3.1. ^1^H (500 MHz) and ^13^C NMR (125 MHz) spectra of compound **3** in CDCl_3_; Figure S3.2. ^1^H-^1^H COSY spectrum of compound **3**; Figure S3.3. HSQC spectrum of compound **3**; Figure S3.4. HMBC spectrum of compound **3**; Figure S3.5. NOESY spectrum of compound **3**; Figure S3.6. CD, MS, and UV spectra of compound **3**; Figure S4.1. ^1^H (500 MHz) and ^13^C NMR (125 MHz) spectra of compound **4** in DMSO-*d*_6_; Figure S4.2. HSQC spectrum of compound **4**; Figure S4.3. HMBC spectrum of compound **4**; Figure S4.4. MS, UV spectra, and sugar analysis by TLC of compound **4**; Figure S5.1. ^1^H (500 MHz) and ^13^C NMR (125 MHz) spectra of compound **5** in CDCl_3_; Figure S5.2. ^1^H-^1^H COSY spectrum of compound **5**; Figure S5.3. HSQC spectrum of compound **5**; Figure S5.4. HMBC spectrum of compound **5**; Figure S5.5. NOESY spectrum of compound **5**; Figure S5.6. CD, MS, and UV spectra of compound **5**; Table S1.1. Detailed DP4+ probability of **1a** (Isomer 1) and **1b** (Isomer 2); Table S1.2. Experimental and calculated ^1^H-NMR chemical shifts (δ in ppm) of **1a** and **1**b; Table S1.3. Experimental and calculated ^13^C-NMR chemical shifts (δ in ppm) of **1a** and **1b**; Table S1.4. Calculated conformational analysis of **1a** at the B3LYP/6-31g(d) level; Table S1.5. Cartesian coordinates of low-energy conformers of **1a**; Table S1.6. Calculated conformational analysis of **1b** at the B3LYP/6-31g(d) level; Table S1.7. Cartesian coordinates of low-energy conformers of **1b**; Table S2.1. Detailed DP4+ probability of **2a** (Isomer 1) and **2b** (Isomer 2); Table S2.2. Experimental and calculated ^1^H-NMR chemical shifts (δ in ppm) of **2a** and **2**b; Table S2.3. Experimental and calculated ^13^C-NMR chemical shifts (δ in ppm) of **2a** and **2b**; Table S1.4. Calculated conformational analysis of **2a** at the B3LYP/6-31g(d) level; Table S2.5. Cartesian coordinates of low-energy conformers of **2a**; Table S2.6. Calculated conformational analysis of **2b** at the B3LYP/6-31g(d) level; Table S2.7. Cartesian coordinates of low-energy conformers of **2b**; Table S3.1. Detailed DP4+ probability of **3a** (Isomer 1) and **3b** (Isomer 2); Table S3.2. Experimental and calculated ^1^H-NMR chemical shifts (δ in ppm) of **3a** and **3**b; Table S3.3. Experimental and calculated ^13^C-NMR chemical shifts (δ in ppm) of **3a** and **3b**; Table S3.4. Calculated conformational analysis of **3a** at the B3LYP/6-31g(d) level; Table S3.5. Cartesian coordinates of low-energy conformers of **3a**; Table S3.6. Calculated conformational analysis of **3b** at the B3LYP/6-31g(d) level; Table S3.7. Cartesian coordinates of low-energy conformers of **3b**; Table S4.1. Detailed DP4+ probability of **5a** (Isomer 1) and **5b** (Isomer 2); Table S4.2. Experimental and calculated ^1^H-NMR chemical shifts (δ in ppm) of **5a** and **5**b; Table S4.3. Experimental and calculated ^13^C-NMR chemical shifts (δ in ppm) of **5a** and **5b**; Table S4.4. Calculated conformational analysis of **5a** at the B3LYP/6-31g(d) level; Table S4.5. Cartesian coordinates of low-energy conformers of **5a**; Table S4.6. Calculated conformational analysis of **5b** at the B3LYP/6-31g(d) level; Table S4.7. Cartesian coordinates of low-energy conformers of **5b**.
