# Supplementary material for: Three New Prenylated Dihydrobenzofurans and a New Flavonoid Glycoside from the Aerial Parts of Myrsine seguinii
Source: Molecules. 2025 Aug 14;30(16):3385. doi: 10.3390/molecules30163385 (PMC12388544; doi:10.3390/molecules30163385)
Supplement: Supplementary file 1 [file molecules-30-03385-s001.zip › molecules-3802622-supplementary.pdf]

Supplementary Information

Three new terpeno-benzoic acid derivatives and a new flavonoid glycoside from the aerial parts of *Myrsine seguinii*

Jin Youngwoo [a, †], Hye Jin Kim[a, †], Kye Jung Shin[a], Khin Myo Htwe[b], Kee Dong Yoon[a,*]

[1] College of Pharmacy and Integrated Research Institute of Pharmaceutical Sciences, The Catholic University of Korea, Bucheon 14662, Republic of Korea; wlsduddnpjk@naver.com (Y.J.); kkhj980316@catholic.ac.kr (H.J.K.); kyejung@catholic.ac.kr (K.J.S.); kdyoon@catholic.ac.kr (K.D.Y.)

[2] Popa Mountain National Park, Forest Department, Kyaukpadaung Township, Mandalay Division, Kyaukpadaung 05241, Myanmar; khinmyohtwe007@gmail.com (K.M.T.)

* Correspondence: kdyoon@catholic.ac.kr (K.D.Y.)

† These authors contributed equally to this work.

Abstract

In this study, we aimed to determine the chemical constituents of *M. seguinii*, which led to the isolation and identification of 26 compounds. Three new prenylated dihydrobenzofurans [myrsinoic acids I (**1**), J (**2**), and K (**3**)] and a new flavonoid glycoside, mearnsetin 3-*O*-*α*-L-arabinopyranoside (**4**), were discovered, and the absolute configuration of known compound, myrsinoic acid B (**5**), was re-established. To ensure the structural accuracy of these compounds, comprehensive spectroscopic analyses were performed, including one- and two-dimensional nuclear magnetic resonance spectroscopy, mass spectrometry, and circular dichroism spectroscopy. In addition, computational analysis methods such as Density Functional Theory (DFT)-based Electronic Circular Dichroism (ECD) simulations and Gauge-Including Atomic Orbitals (GIAO) ^{1}H and ^{13}C NMR chemical shift calculations with DP4+ probability analysis were utilised to further support the structural assignments.

Keywords: computational analysis; dihydrobenzofuran; DP4+ probability; electronic circular dichroism; flavonoid glycoside; *Myrsine seguinii*; Primulaceae

Content

1. Spectroscopic data of compounds 6-26 4

SI 1.1. ^{1}H (500 MHz), ^{13}C (125 MHz) NMR and MS data of **6-26** 4

2. Spectra of physico-chemical properties of 1-5

2.1. Compound 1

Figure S1.1. ^{1}H (500 MHz) and ^{13}C NMR (125 MHz) spectra of compound **1** in $CDCl_3$ 8

Figure S1.2. ^{1}H-^{1}H COSY spectrum of compound **1** 9

Figure S1.3. HSQC spectrum of compound **1** 10

Figure S1.4. HMBC spectrum of compound **1** 11

Figure S1.5. NOESY spectrum of compound **1** 12

Figure S1.6. CD, MS, and UV spectra of compound **1** 13

2.2. Compound 2

Figure S2.1. ^{1}H (500 MHz) and ^{13}C NMR (125 MHz) spectra of compound **2** in $CDCl_3$ 14

Figure S2.2. ^{1}H-^{1}H COSY spectrum of compound **2** 15

Figure S2.3. HSQC spectrum of compound **2** 16

Figure S2.4. HMBC spectrum of compound **2** 17

Figure S2.5. NOESY spectrum of compound **2** 18

Figure S2.6. CD, MS, and UV spectra of compound **2** 19

2.3. Compound 3

Figure S3.1. ^{1}H (500 MHz) and ^{13}C NMR (125 MHz) spectra of compound **3** in $CDCl_3$ 20

Figure S3.2. ^{1}H-^{1}H COSY spectrum of compound **3** 21

Figure S3.3. HSQC spectrum of compound **3** 22

Figure S3.4. HMBC spectrum of compound **3** 23

Figure S3.5. NOESY spectrum of compound **3** 24

Figure S3.6. CD, MS, and UV spectra of compound **3** 25

2.4. Compound 4

Figure S4.1. ^{1}H (500 MHz) and ^{13}C NMR (125 MHz) spectra of compound **4** in DMSO-d_6 26

Figure S4.2. HSQC spectrum of compound **4** 27

Figure S4.3. HMBC spectrum of compound **4** 28

Figure S4.4. MS, UV spectra and sugar analysis by TLC of compound **4** 29

2.5. Compound 5

Figure S.5.1. ^{1}H (500 MHz) and ^{13}C NMR (125 MHz) spectra of compound **5** in $CDCl_3$ 30

Figure S5.2. ^{1}H-^{1}H COSY spectrum of compound **5** 31

Figure S5.3. HSQC spectrum of compound **5** 32

Figure S5.4. HMBC spectrum of compound **5** 33

Figure S5.5. NOESY spectrum of compound **5** 34

Figure S5.6. CD, MS, and HPLC-PDA spectra of compound **5** 35

3. Computational data of compounds 1-3 and 5

3.1. Compound 1

Table S1.1. Detailed DP4+ probability of **1a** (Isomer 1) and **1b** (Isomer 2) 36

Table S1.2. Experimental and calculated ^{1}H-NMR chemical shifts (δ in ppm) of **1a** and **1b** 37

Table S1.3. Experimental and calculated ^{13}C-NMR chemical shifts (δ in ppm) of **1a** and **1b** 38

Table S1.4. Calculated conformational analysis of the **1a** at B3LYP/6-31g(d) level 39

Table S1.5. Cartesian coordinates of low-energy conformers of **1a** 42

Table S1.6. Calculated conformational analysis of the **1b** at B3LYP/6-31g(d) level 47

Table S1.7. Cartesian coordinates of low-energy conformers of **1b** 50

3.2. Compound 2

Table S2.1. Detailed DP4+ probability of **2a** (Isomer 1) and **2b** (Isomer 2) 55

Table S2.2. Experimental and calculated ^{1}H-NMR chemical shifts (δ in ppm) of **2a** and **2b** 56

Table S2.3. Experimental and calculated ^{13}C-NMR chemical shifts (δ in ppm) of **2a** and **2b** 57

Table S2.4. Calculated conformational analysis of the **2a** at B3LYP/6-31g(d) level 58

Table S2.5. Cartesian coordinates of low-energy conformers of **2a** 61

Table S2.6. Calculated conformational analysis of the **2b** at B3LYP/6-31g(d) level 66

Table S2.7. Cartesian coordinates of low-energy conformers of **2b** 68

3.3. Compound 3

Table S3.1. Detailed DP4+ probability of **3a** (Isomer 1) and **3b** (Isomer 2) 72

Table S3.2. Experimental and calculated ^{1}H-NMR chemical shifts (δ in ppm) of **3a** and **3b** 73

Table S3.3. Experimental and calculated ^{13}C-NMR chemical shifts (δ in ppm) of **3a** and **3b** 74

Table S3.4. Calculated conformational analysis of the **3a** at B3LYP/6-31g(d) level 75

Table S3.5. Cartesian coordinates of low-energy conformers of **3a** 78

Table S3.6. Calculated conformational analysis of the **3b** at B3LYP/6-31g(d) level 83

Table S3.7. Cartesian coordinates of low-energy conformers of **3b** 85

3.4. Compound 5

Table S4.1. Detailed DP4+ probability of **5a** (Isomer 1) and **5b** (Isomer 2) 89

Table S4.2. Experimental and calculated ^{1}H-NMR chemical shifts (δ in ppm) of **5a** and **5b** 90

Table S4.3. Experimental and calculated ^{13}C-NMR chemical shifts (δ in ppm) of **5a** and **5b** 91

Table S.4.4. Calculated conformational analysis of the **5a** at B3LYP/6-31g(d) level 92

Table S4.5. Cartesian coordinates of low-energy conformers of **5a** 96

Table S4.6. Calculated conformational analysis of the **5b** at B3LYP/6-31g(d) level 103

Table S4.7. Cartesian coordinates of low-energy conformers of **5b** 106

1. Spectroscopic data of compounds 6-26

SI 1.1. ^{1}H (500 MHz), ^{13}C (125 MHz) NMR and MS data of **6-26**

Myrsinoic acid A (**6**): $C_{22}H_{30}O_3$; colorless oil; ESI-Q-TOF-MS: *m/z* 365.2084 $[M+Na]^+$ (calcd for $C_{22}H_{30}O_3$, 365.2093); ^{1}H-NMR (500 MHz, $CDCl_3$): δ 7.75 (2H, s, H-2, 6), 5.30 (2H, tt, J = 7.2, 1.5 Hz, H-2', 2''), 5.06 (1H, tq, J = 5.3, 1.5 Hz, H-6'), 3.36 (4H, dd, J = 10.3, 7.2 Hz, H-1', 1''), 2.09 (4H, dq, J = 12.1, 6.3 Hz, H-4', 5'), 1.76 (9H, dt, J = 3.1, 1.5 Hz, H-9', 4'', 5''), 1.67 (3H, d, J = 1.4 Hz, H-8''), 1.59 (3H, d, J = 1.3 Hz, H-10'); ^{13}C-NMR (125 MHz, $CDCl_3$): δ 172.09 (C-7), 158.18 (C-4), 139.32 (C-3'), 135.14 (C-3''), 132.23 (C-7'), 130.68 (C-2, 6), 127.62 (C-3 or 5), 127,17 (C-3 or 5), 123.99 (C-1, 6′), 121.56 (C-2''), 121.39 (C-2'), 39.91 (C-4'), 29.89 (C-1' or 1''), 29.56 (C-1′ or 1''), 26.60 (C-5'), 26.03 (C-4''), 25.90 (C-8'), 18.11 (C-5''), 17.93 (C-10'), 16.45 (C-9').

Myricetin 4′-methyl ether 3-*O*-*β*-D-galactopyranoside (**7**): $C_{22}H_{22}O_{13}$; yellow amorphous powder; ESI-Q-TOF-MS: *m/z* 517.0958 $[M+Na]^+$ (calcd for $C_{22}H_{22}O_{13}$, 517.0958); ^{1}H-NMR (500 MHz, DMSO-d_6): δ 7.14 (2H, s, H-2', 6'), 6.38 (1H, t, J = 2.0 Hz, H-8), 6.21 (1H, d, J = 2.0 Hz, H-6), 5.37 (1H, d, J = 7.8 Hz, H-1''), 3.75 (3H, s, 4'-$OC\underline{H_3}$), 3.63 (1H, t, J = 4.3 Hz, H-4''), 3.57 (1H, ddd, J = 9.6, 7.8, 4.7 Hz, H-2''), 3.45 (1H, m, H-6''b), 3.34 – 3.42 (3H, m, H-3'', 5'', 6''a); ^{13}C-NMR (125 MHz, DMSO-d_6): δ 177.63 (C-4), 164.34 (C-7), 161.28 (C-5), 156.36 (C-9), 155.61 (C-2), 150.30 (C-3', 5'), 137.90 (C-4'), 134.33 (C-3), 125.19 (C-1'), 108.64 (C-2', 6'), 104.05 (C-10), 101.87 (C-1''), 98.77 (C-6), 93.47 (C-8), 75.99 (C-5''), 73.24 (C-3''), 71.15 (C-2''), 67.98 (C-4''), 60.05 (C-6''), 59.81 (4'-$O\underline{C}H_3$).

Mearnsitrin (**8**): $C_{22}H_{22}O_{12}$; yellow amorphous powder; ESI-Q-TOF-MS: *m/z* 501.1013 $[M+Na]^+$ (calcd for $C_{22}H_{22}O_{12}$, 501.1009); ^{1}H-NMR (500 MHz, DMSO-d_6): δ 6.81 (2H, s, H-2', 6'), 6.37 (1H, d, J = 2.1 Hz, H-8), 6.21 (1H, d, J = 2.1 Hz, H-6), 5.15 (1H, d, J = 1.6 Hz, H-1''), 3.97 (1H, dd, J = 3.4, 1.6 Hz, H-2''), 3.74 (3H, s, 4'-$OC\underline{H_3}$), 3.50 (1H, q, J = 3.4 Hz, H-3''), 3.16 – 3.13 (2H, m, H-4'', 5''), 0.81 (3H, m, H-6''); ^{13}C-NMR (125 MHz, DMSO-d_6): δ 177.83 (C-4), 164.47 (C-7), 161.32 (C-5), 157.27 (C-2), 156.54 (C-9), 150.69 (C-3', 5'), 137.78 (C-4'), 134.85 (C-3), 124.83 (C-1'), 108.12 (C-2',6'), 104.17 (C-10), 102.18 (C-1''), 98.79 (C-6), 93.65 (C-8), 71.15 (C-4''), 70.54 (C-5''), 70.33 (C-3''), 70.05 (C-2''), 59.77 (4'-$O\underline{C}H_3$), 17.47 (C-6'').

Mearnsetin (**9**): $C_{16}H_{12}O_8$; yellow amorphous powder; ESI-Q-TOF-MS: *m/z* 331.0454 $[M-H]^-$ (calcd for $C_{16}H_{12}O_8$, 331.0454); ^{1}H-NMR (500 MHz, DMSO-d_6): δ 7.21 (2H, s, H-2', 6'), 6.38 (1H, d, J = 2.0 Hz, H-8), 6.19 (1H, d, J = 2.0 Hz, H-6), 3.74 (3H, s, 4'-$OC\underline{H_3}$); ^{13}C-NMR (125 MHz, DMSO-d_6): δ 176.09 (C-4), 164.13 (C-7), 160.79 (C-5), 156.21 (C-9), 150.65 (C-3', 5'), 145.98 (C-2), 137.21 (C-4'), 136.66 (C-3), 125.88 (C-1'), 107.24 (C-2', 6'), 103.10 (C-10), 98.28 (C-6), 93.29 (C-8), 59.79 (4'-$O\underline{C}H_3$).

Myricitrin (**10**): $C_{21}H_{20}O_{12}$; yellow amorphous powder; ESI-Q-TOF-MS: *m/z* 487.0854 $[M+Na]^+$ (calcd for $C_{21}H_{20}O_{12}$, 487.0852); ^{1}H-NMR (500 MHz, DMSO-d_6): δ 6.81 (2H, s, H-2′, 6′), 6.37 (1H, d, J = 2.1 Hz, H-8), 6.20 (1H, d, J = 2.0 Hz, H-6), 5.19 (1H, d, J = 1.6 Hz, H-1''), 3.98 (1H, dd, J = 3.4, 1.6 Hz, H-2''), 3.55 (1H, q, J = 9.3, 3.3 Hz, H-3''), 3.36 (1H, dq, J = 9.7, 6.6 Hz, H-5''), 3.15 (1H, s, H-4''), 0.84 (3H, d, J = 6.2 Hz H-6''); ^{13}C-NMR (125 MHz, DMSO-d_6): δ 177.78 (C-4), 164.17 (C-7), 161.31 (C-5), 157.49 (C-2), 156.4 (C-9), 145.77 (C-3', 5'), 136.45 (C-4'), 134.28 (C-3), 119.6 (C-1'), 107.89 (C-2', 6'), 104.03 (C-10), 101.93 (C-1''), 98.64 (C-6), 93.51 (C-8), 71.26 (C-4''), 70.55 (C-5''), 70.38 (C-3''), 70.01 (C-2''), 17.52 (C-6'').

Quercitrin (**11**): $C_{21}H_{20}O_{11}$; yellow amorphous powder; ESI-Q-TOF-MS: *m/z* 471.0904 $[M+Na]^+$ (calcd for $C_{21}H_{20}O_{12}$, 471.0903); ^{1}H-NMR (500 MHz, DMSO-d_6): δ 7.29 (1H, d, J = 2.2 Hz, H-2′), 7.25 (1H, dd, J = 8.3,

2.2 Hz, H-6′), 6.86 (1H, d, J = 8.4 Hz, H-5′), 6.39 (1H, d, J = 2.0 Hz, H-8), 6.20 (1H, d, J = 2.0 Hz, H-6), 5.25 (1H, d, J = 1.6 Hz, H-1''), 3.97 (1H, dd, J = 3.5, 1.7 Hz, H-2''), 3.50 (1H, dd, J = 9.1, 3.3 Hz, H-3''), 3.19 (1H, d, J = 6.1 Hz, H-5''), 3.13 (1H, d, J = 9.4 Hz, H-4''), 0.81 (3H, d, J = 6.0 Hz H-6''); ^{13}C-NMR (125 MHz, DMSO-d_6): δ 177.78 (C-4), 164.23 (C-7), 161.33 (C-5), 157.35 (C-2), 156.48 (C-9), 148.47 (C-4'), 145.24 (C-3'), 134.23 (C-3), 121.15 (C-6'), 120.74 (C-1'), 115.66 (C-2'), 115.48 (C-5'), 104.11 (C-10), 101.84 (C-1''), 98.72 (C-6), 93.66 (C-8), 71.16 (C-4''), 70.63 (C-5''), 70.33 (C-3''), 70.07 (C-2''), 17.54 (C-6'').

Tamarixetin 3-*O*-α-L-rhamnoside (**12**): $C_{22}H_{22}O_{11}$; yellow amorphous powder; ESI-Q-TOF-MS: *m/z* 485.1059 $[M+Na]^+$ (calcd for $C_{22}H_{22}O_{11}$, 485.1060); ^{1}H-NMR (500 MHz, DMSO-d_6): δ 7.35 (1H, dd, J = 8.5, 2.2 Hz, H-6'), 7.31 (1H, d, J = 2.2 Hz, H-2'), 7.07 (1H, d, J = 8.6 Hz, H-5'), 6.41 (1H, d, J = 2.1 Hz, H-8), 6.21 (1H, d, J = 2.0 Hz, H-6), 5.23 (1H, d, J = 1.7 Hz, H-1''), 3.97 (1H, dd, J = 3.3, 1.7 Hz, H-2''), 3.84 (3H, s, 4'-OC$\underline{H_3}$), 3.49 (1H, dd, J = 8.8, 3.3 Hz, H-3''), 3.14 (2H, dd, J = 6.8, 4.5 Hz, H-4'', 5''), 0.80 (3H, d, J = 5.4 Hz, H-6''); ^{13}C-NMR (125 MHz, DMSO-d_6): δ 177.86 (C-4), 164.33 (C-7), 161.34 (C-5), 157.06 (C-2), 156.55 (C-9), 150.10 (C-4'), 146.37 (C-3'), 134.57 (C-3), 122.32 (C-1'), 120.87 (C-6'), 115.40 (C-2'), 111.76 (C-5'), 104.19 (C-10), 101.98 (C-1''), 98.78 (C-6), 93.75 (C-8), 71.17 (C-5''), 70.64 (C-4''), 70.34 (C-3''), 70.08 (C-2''), 55.73 (4'-O$\underline{C}H_3$), 17.53 (C-6'').

Kaempferol 3-*O*-α-L-rhamnoside (**13**): $C_{21}H_{20}O_{10}$; yellow amorphous powder; ESI-Q-TOF-MS: *m/z* 455.0956 $[M+Na]^+$ (calcd for $C_{21}H_{20}O_{10}$, 455.0954); ^{1}H-NMR (500 MHz, DMSO-d_6): δ 7.75 (2H, d, J = 8.7 Hz H-2', 6'), 6.91 (2H, d, J = 8.8 Hz, H-3', 5'), 6.41 (1H, d, J = 2.0 Hz, H-8), 6.21 (1H, d, J = 2.1 Hz, H-6), 5.28 (1H, d, J = 1.7 Hz, H-1''), 3.97 (1H, dd, J = 4.2, 2.1 Hz, H-2''), 3.46 (3H, dd, J = 9.1, 3.3 Hz, H-3''), 3.12 (1H, d, J = 9.3 Hz, H-4''), 3.06 (1H, dd, J = 9.5, 6.0 Hz, H-5''), 0.78 (3H, d, J = 6.1 Hz, H-6''); ^{13}C-NMR (125 MHz, DMSO-d_6): δ 177.74 (C-4), 164.25 (C-7), 161.30 (C-5), 160.03 (C-4'), 157.29 (C-2), 156.52 (C-9), 134.20 (C-3), 130.65 (C-2', 6'), 120.52 (C-1'), 115.42 (C-3', 5'), 104.15 (C-10), 101.79 (C-1''), 98.75 (C-6), 93.78 (C-8), 71.09 (C-4''), 70.67 (C-5''), 70.31 (C-3''), 70.09 (C-2''), 17.49 (C-6'').

Seguinoside D (**14**): $C_{24}H_{28}O_{13}$; amorphous powder; ESI-Q-TOF-MS: *m/z* 547.1427 $[M+Na]^+$ (calcd for $C_{24}H_{28}O_{13}$, 547.1428); ^{1}H-NMR (500 MHz, CD_3OD): δ 7.83 (2H, d, J = 8.8 Hz, H-2''', 6'''), 6.86 (2H, d, J = 9.0 Hz, H-3, 5), 6.78 (2H, d, J = 8.8 Hz, H-3''', 5'''), 6.56 (1H, d, J = 9.0 Hz, H-2, 6), 5.50 (1H, d, J = 1.4 Hz, H-1''), 4.79 (1H, d, J = 7.6 Hz, H-1'), 4.31 (3H, m, H-4''b, 5''), 4.01 (1H, d, J = 1.4 Hz, H-2''), 3.89-3.40 (7H, m, H-2'~6', 4''); ^{13}C-NMR (125 MHz, CD_3OD): δ 167.99 (C-7'''), 163.75 (C-4'''), 153.73 (C-1), 152.29 (C-4), 133.11 (C-2''', 6'''), 122.10 (C-1′′′), 118.97 (C-3, 5), 116.82 (C-2, 6), 116.31 (C-3''', 5'''), 110.65 (C-1''), 102.00 (C-1'), 79.38 (C-3''), 78.94 (C-2'), 78.82 (C-5'), 78.59 (C-3'), 78.09 (C-2''), 75.58 (C-4''), 71.70 (C-4'), 68.14 (C-5''), 62.69 (C-6').

Seguinoside E (**15**): $C_{25}H_{30}O_{14}$; amorphous powder; ESI-Q-TOF-MS: *m/z* 577.1536 $[M+Na]^+$ (calcd for $C_{25}H_{30}O_{14}$, 577.1533); ^{1}H-NMR (500 MHz, CD_3OD): δ 7.52 (1H, dd, J = 8.2, 2.0 Hz, H-6'''), 7.50 (1H, d, J = 2.0 Hz, H-2'''), 6.84 (2H, d, J = 9.0 Hz, H-2, 6), 6.80 (1H, d, J = 8.2 Hz, H-5'''), 6.54 (2H, d, J = 9.0 Hz, H-3, 5), 5.51 (1H, d, J = 1.3 Hz, H-1''), 4.79 (1H, d, J = 7.5 Hz, H-1'), 4.37 (1H, d, J = 11.3 Hz, H-5''b), 4.32 (1H, d, J = 9.8 Hz, H-4''b), 4.27 (1H, d, J = 11.4 Hz, H-5''a), 4.01 (1H, d, J = 1.3 Hz, H-2''), 3.91 (1H, d, J = 9.7 Hz, H-4''a), 3.88 (3H, s, 3'''-OC$\underline{H_3}$), 3.85 (1H, d, J = 1.9 Hz, H-6'a), 3.70-3.55 (6H, m, H-2'~6'b); ^{13}C-NMR (125 MHz, CD_3OD): δ 167.95 (C-7'''), 153.69 (C-1), 153.09 (C-4'''), 152.22 (C-4), 148.85 (C-3'''), 125.42 (C-6'''), 122.38 (C-1'''), 118.89 (C-2, 6), 116.78 (C-3, 5), 116.06 (C-5'''), 113.84 (C-2'''), 110.59 (C-1''), 101.90 (C-1'), 79.40 (C-3''), 78.95 (C-2'), 78.80 (C-5'), 78.47 (C-3'), 78.07 (C-2''), 75.56 (C-4''), 71.65 (C-4'), 68.40 (C-5''), 62.64 (C-6'), 56.58 (3'''-O$\underline{C}H_3$).

Breynioside A (**16**): $C_{19}H_{20}O_9$; Colorless needles; ESI-Q-TOF-MS: *m/z* 415.1006 $[M+Na]^+$ (calcd for $C_{19}H_{20}O_9$, 415.1005); ^{1}H-NMR (500 MHz, CD_3OD): δ 7.91 (2H, d, J = 8.7 Hz, H-2'', 6''), 6.94 (2H, d, J = 9.0 Hz, H-2, 6),

6.86 (2H, d, J = 8.7 Hz, H-3'', 5''), 6.60 (2H, d, J = 8.9 Hz, H-3, 5), 4.74 (1H, d, J = 7.2 Hz, H-1'), 4.67 (1H, d, J = 11.8, 2.2 Hz, H-6'a), 4.35 (1H, dd, J = 11.8, 7.5 Hz, H-6'b), 3.71 (1H, ddd, J = 9.7, 7.5, 2.2 Hz, H-5'), 3.44 (3H, m, H-2', 3', 4'); ^{13}C-NMR (125 MHz, CD_3OD): δ 168.07 (C-7''), 163.83 (C-4''), 154.05 (C-4), 152.45 (C-1), 133.09 (C-2'', 6''), 122.30 (C-1''), 119.67 (C-2, 6), 116.70 (C-3, 5), 116.37 (C-3'', 5''), 103.79 (C-1'), 78.11 (C-3'), 75.69 (C-5'), 75.11 (C-2'), 72.20 (C-4'), 65.20 (C-6').

6'-*O*-vanilloylarbutin (**17**): $C_{20}H_{22}O_{10}$; yellow amorphous powder; ESI-Q-TOF-MS: *m/z* 445.1110 $[M+Na]^+$ (calcd for $C_{20}H_{22}O_{10}$, 445.1111); ^{1}H-NMR (500 MHz, CD_3OD): δ 7.59 (1H, dd, J = 8.3, 2.0 Hz, H-6''), 7.56 (1H, d, J = 2.0 Hz, H-2''), 6.92 (2H, d, J = 9.0 Hz, H-2, 6), 6.88 (1H, d, J = 8.3 Hz, H-5''), 6.57 (2H, d, J = 9.0 Hz, H-3, 5), 4.74 (1H, d, J = 7.3 Hz, H-1'), 4.69 (1H, d, J = 11.8, 2.2 Hz, H-6'a), 4.36 (1H, dd, J = 11.8, 7.5 Hz, H-6'b), 3.87 (3H, s, 3''-OC$\underline{H_3}$), 3.72 (1H, ddd, J = 9.7, 7.5, 2.2 Hz, H-5'), 3.44 (3H, m, H-2', 3', 4'); ^{13}C-NMR (125 MHz, CD_3OD): δ 168.03 (C-7''), 154.04 (C-4), 153.13 (C-4''), 152.42 (C-1), 148.93 (C-3''), 125.38 (C-6''), 122.60 (C-1''), 119.64 (C-2, 6), 116.69 (C-3, 5), 116.13 (C-5''), 113.81 (C-2''), 103.77 (C-1'), 78.06 (C-3'), 75.68 (C-5'), 75.09 (C-2'), 72.22 (C-4'), 65.31 (C-6'), 56.60 (3''-O$\underline{C}H_3$).

1-(3,5-dihydroxyphenyl)heptan-1-one (**18**): $C_{13}H_{18}O_3$; Colorless needles; ESI-Q-TOF-MS: *m/z* 245.1154 $[M+Na]^+$ (calcd for $C_{13}H_{18}O_3$, 245.1154); ^{1}H-NMR (500 MHz, CD_3OD): δ 6.87 (2H, d, J = 2.3 Hz, H-2, 6), 6.47 (1H, t, J = 2.2 Hz, H-4), 2.90 (2H, t, J = 7.4 Hz, H-2'), 1.66 (2H, m, H-3'), 1.34 (6H, m, H-4', 5', 6'), 0.91 (3H, m, H-7'); ^{13}C-NMR (125 MHz, CD_3OD): δ 203.04 (C-1'), 160.17 (C-3, 5), 140.50 (C-1), 108.39 (C-4), 107.57 (C-2, 6), 39.77 (C-2'), 33.03 (C-5'), 30.24 (C-4'), 25.88 (C-3'), 23.77 (C-6'), 14.54 (C-7').

Gallic acid (**19**): $C_7H_6O_5$; amorphous white powder; ESI-Q-TOF-MS: *m/z* 169.0137 $[M-H]^-$ (calcd for $C_7H_6O_5$, 169.0137); ^{1}H-NMR (500 MHz, CD_3OD): δ 7.05 (2H, s, H-2, 6); ^{13}C-NMR (125 MHz, CD_3OD): δ 170.58 (C-7), 146.55 (C-3, 5), 139.73 (C-4), 122.11 (C-1), 110.43 (C-2, 6).

4-*O*-methylgallic acid (**20**): $C_8H_8O_5$; white amorphous solid; ESI-Q-TOF-MS: *m/z* 185.0450 $[M+H]^+$ (calcd for $C_8H_8O_5$, 185.0450); ^{1}H-NMR (500 MHz, CD_3OD): δ 7.04 (2H, s, H-2, 6), 3.85 (1H, s, 4-OC$\underline{H_3}$); ^{13}C-NMR (125 MHz, CD_3OD): δ 170.08 (C-7), 151.76 (C-3, 5), 141.10 (C-4), 127.58 (C-1), 110.54 (C-2, 6), 60.85 (4-O$\underline{C}H_3$).

Protocatechuic acid (**21**): $C_7H_6O_4$; white powder; ESI-Q-TOF-MS: *m/z* 153.0186 $[M-H]^-$ (calcd for $C_7H_6O_4$, 153.0188); ^{1}H-NMR (500 MHz, CD_3OD): δ 7.43 (1H, s, H-2), 7.42 (1H, s, H-6), 6.79 (1H, d, J = 8.0 Hz H-5); ^{13}C-NMR (125 MHz, CD_3OD): δ 170.57 (C-7), 151.65 (C-4), 146.21 (C-3), 124.03 (C-5), 123.42 (C-1), 117.87 (C-2), 115.87 (C-5).

Vanillic acid (**22**): $C_8H_8O_4$; Colorless needles; ESI-Q-TOF-MS: *m/z* 169.0508 $[M+H]^+$ (calcd for $C_8H_8O_4$, 169.0501); ^{1}H-NMR (500 MHz, $CDCl_3$): δ 7.71 (1H, dd, J = 8.3, 1.9 Hz, H-6), 7.57 (1H, d, J = 1.9 Hz, H-2), 6.96 (1H, d, J = 8.3 Hz, H-5), 3.95 (1H, s, 3-OC$\underline{H_3}$); ^{13}C-NMR (125 MHz, $CDCl_3$): δ 170.45 (C-7), 150.98 (C-4), 146.40 (C-3), 125.39 (C-6), 121.26 (C-1), 114.40 (C-5), 112.28 (C-2), 56.34 (3-O$\underline{C}H_3$).

Resorcylic acid (**23**): $C_7H_6O_4$; white powder; ESI-Q-TOF-MS: *m/z* 153.0187 $[M-H]^-$ (calcd for $C_7H_6O_4$, 153.0188); ^{1}H-NMR (500 MHz, CD_3OD): δ 6.93 (2H, d, J = 2.3 Hz, H-2, 6), 6.45 (1H, t, J = 2.3 Hz, H-5); ^{13}C-NMR (125 MHz, CD_3OD): δ 169.52 (C-7), 159.82 (C-3, 5), 134.14 (C-1), 109.20 (C-2, 6), 108.04 (C-4).

4-hydroxy-benzoic acid (**24**): $C_7H_6O_3$; white needles; ESI-Q-TOF-MS: *m/z* 139.0396 $[M+H]^+$ (calcd for $C_7H_6O_3$, 139.0395); ^{1}H-NMR (500 MHz, CD_3OD): δ 7.88 (2H, d, J = 8.8 Hz, H-2, 6), 6.81 (2H, d, J = 8.8 Hz, H-3, 5); ^{13}C-NMR (125 MHz, CD_3OD): δ 168.70 (C-7), 161.98 (C-4), 131.62 (C-2, 6), 121.35 (C-1), 114.62 (C-3, 5).

(6*R*,9*S*)-blumenol C glucoside (**25**): $C_{19}H_{32}O_7$; white amorphous solid; $[\alpha]^{25}_D$ = 10.67° (*c* 0.03, MeOH); ESI-Q-TOF-MS: *m/z* 395.2046 $[M+Na]^+$ (calcd for $C_{19}H_{32}O_7$, 395.2046); ^{1}H-NMR (500 MHz, CD_3OD): δ 5.80 (1H, d, *J* = 1.6Hz, H-4), 4.32 (1H, d, *J* = 7.8 Hz, H-1'), 3.85 (1H, m, H-6'b), 3.82 (1H, m, H-9), 3.66 (1H, m, H-6a'), 3.34 (1H, m, H-3'), 3.26 (2H, m, H-4', 5'), 3.14 (1H, m, H-2'), 2.49 (1H, d, *J* = 17.4 Hz, H-2b), 2.05 (3H, d, *J* = 1.3 Hz, H-13), 1.98 (2H, m, H-2a, 6), 1.80 (1H, m, H-7b), 1.69 (1H, m, H-7a), 1.64 (2H, m, H-8), 1.25 (3H, d, *J* = 6.3 Hz, H-10), 1.10 (3H, s, H-12), 1.02 (3H, s, H-11); ^{13}C-NMR (125 MHz, CD_3OD): δ 202.56 (C-3), 170.08 (C-5), 125.58 (C-4), 104.18 (C-1'), 78.37 (C-3′), 78.01 (C-5'), 77.80 (C-9), 75.46 (C-2'), 71.84 (C-4'), 62.95 (C-6'), 52.71 (C-6), 48.21 (C-2), 37.58 (C-8), 37.53 (C-1), 29.17 (C-11), 27.62 (C-12), 26.81 (C-7), 25.14 (C-13), 22.10 (C-10).

Byzantionoside B 6′-*O*-*β*-D-apiofuranoside (**26**): $C_{24}H_{40}O_{11}$; white powder; $[\alpha]^{25}_D$ = -13.32° (*c* 0.14, MeOH); ESI-Q-TOF-MS: *m/z* 527.2472 $[M+Na]^+$ (calcd for $C_{24}H_{40}O_{11}$, 527.2468); ^{1}H-NMR (500 MHz, CD_3OD): δ 5.81 (1H, m, H-4), 5.02 (1H, d, *J* = 2.5 Hz, H-1''), 4.30 (1H, d, *J* = 7.8 Hz, H-1'), 3.95 (2H, m, H-6'a, 4''a), 3.88 (1H, d, *J* = 2.4 HZ, H-2''), 3.83 (1H, m, H-9), 3.75 (1H, d, *J* = 9.6 Hz, H-4''b), 3.59 (1H, m, H-6b'), 3.56 (2H, s, H-5''), 3.38 (1H, ddd, *J* = 9.9, 6.4, 1.9 Hz, H-5'), 3.35 (1H, s, H-3'), 3.25 (1H, m, H-4'), 3.13 (1H, dd, *J* = 9.2, 7.8 Hz, H-2'), 2.49 (1H, d, *J* = 17.4 Hz, H-2a), 2.06 (3H, d, J = 1.3 Hz, H-13), 2.00 (3H, m, H-2b, 6, 7a), 1.62 (2H, m, H-8), 1.51 (1H, m, H-7b), 1.19 (3H, d, *J* = 6.2 Hz, H-10), 1.10 (3H, s, H-11), 1.01 (3H, s, H-12); ^{13}C-NMR (125 MHz, CD_3OD): δ 202.76 (C-3), 170.52 (C-5), 125.52 (C-4), 111.10 (C-1''), 102.47 (C-1'), 80.66 (C-3''), 78.25 (C-3'), 78.13 (C-2''), 77.17 (C-5'), 75.94 (C-9), 75.27 (C-2'), 75.10 (C-4''), 71.98 (C-4'), 68.88 (C-6'), 65.76 (C-5''), 52.49 (C-6), 48.24 (C-2), 37.89 (C-8), 37.51 (C-1), 29.21 (C-12), 27.76 (C-11), 27.04 (C-7), 25.23 (C-13), 20.28 (C-10).

2. Spectra of physico-chemical properties of 1-5

2.1. Compound 1

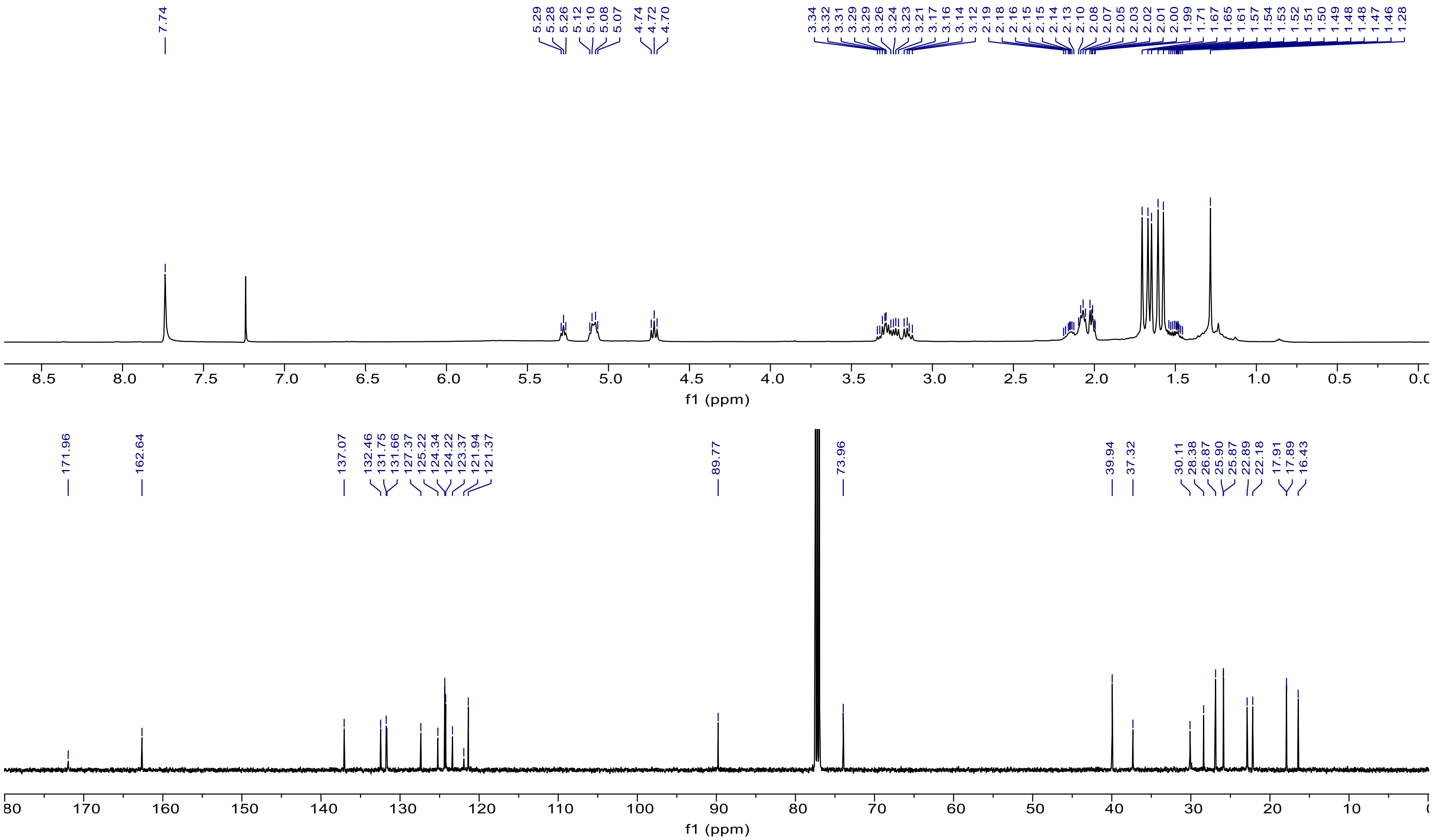

Figure S1.1. ^{1}H (500 MHz) and ^{13}C NMR (125 MHz) spectra of compound **1** in $CDCl_3$

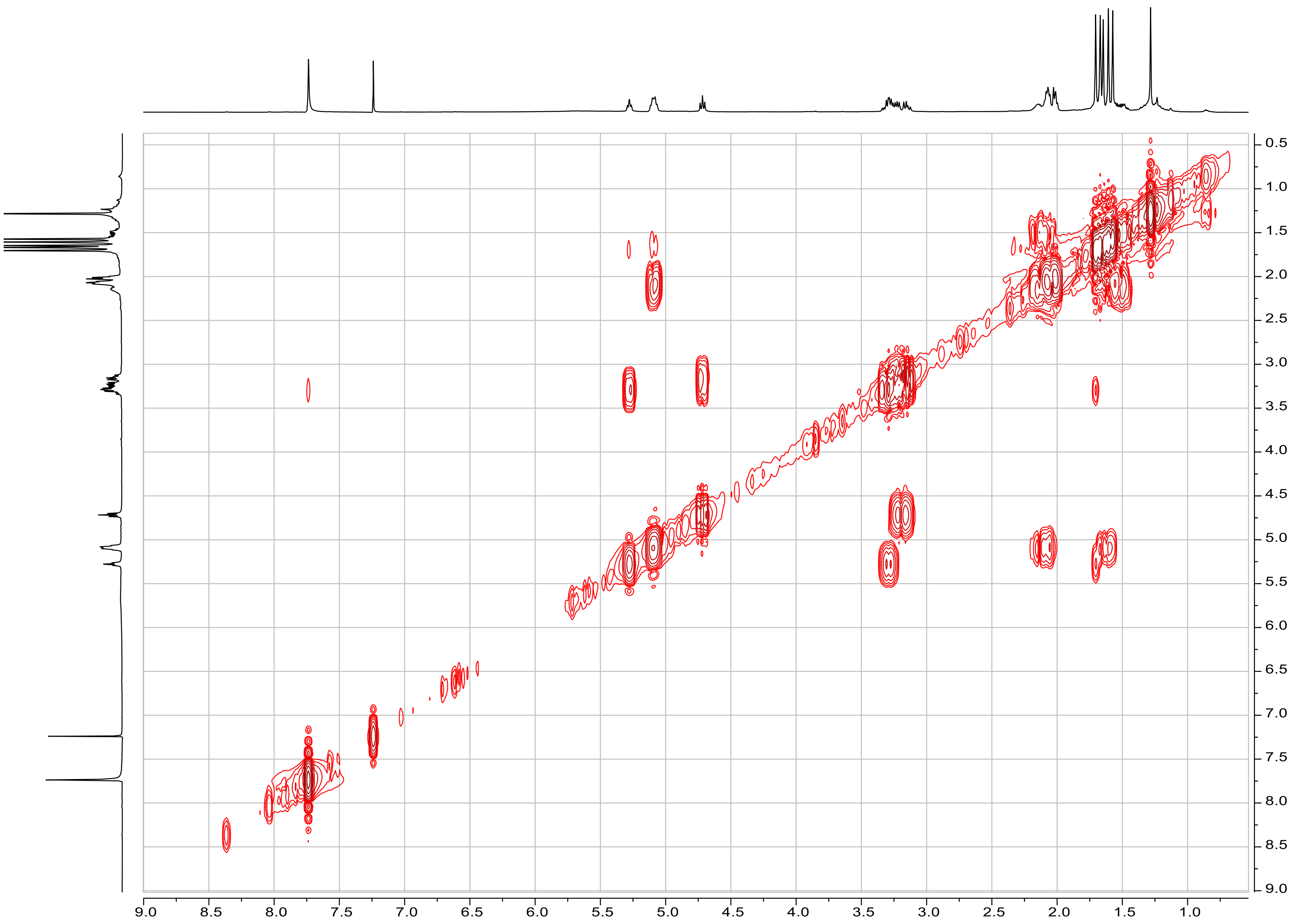

Figure S1.2. ^{1}H-^{1}H COSY spectrum of compound **1**

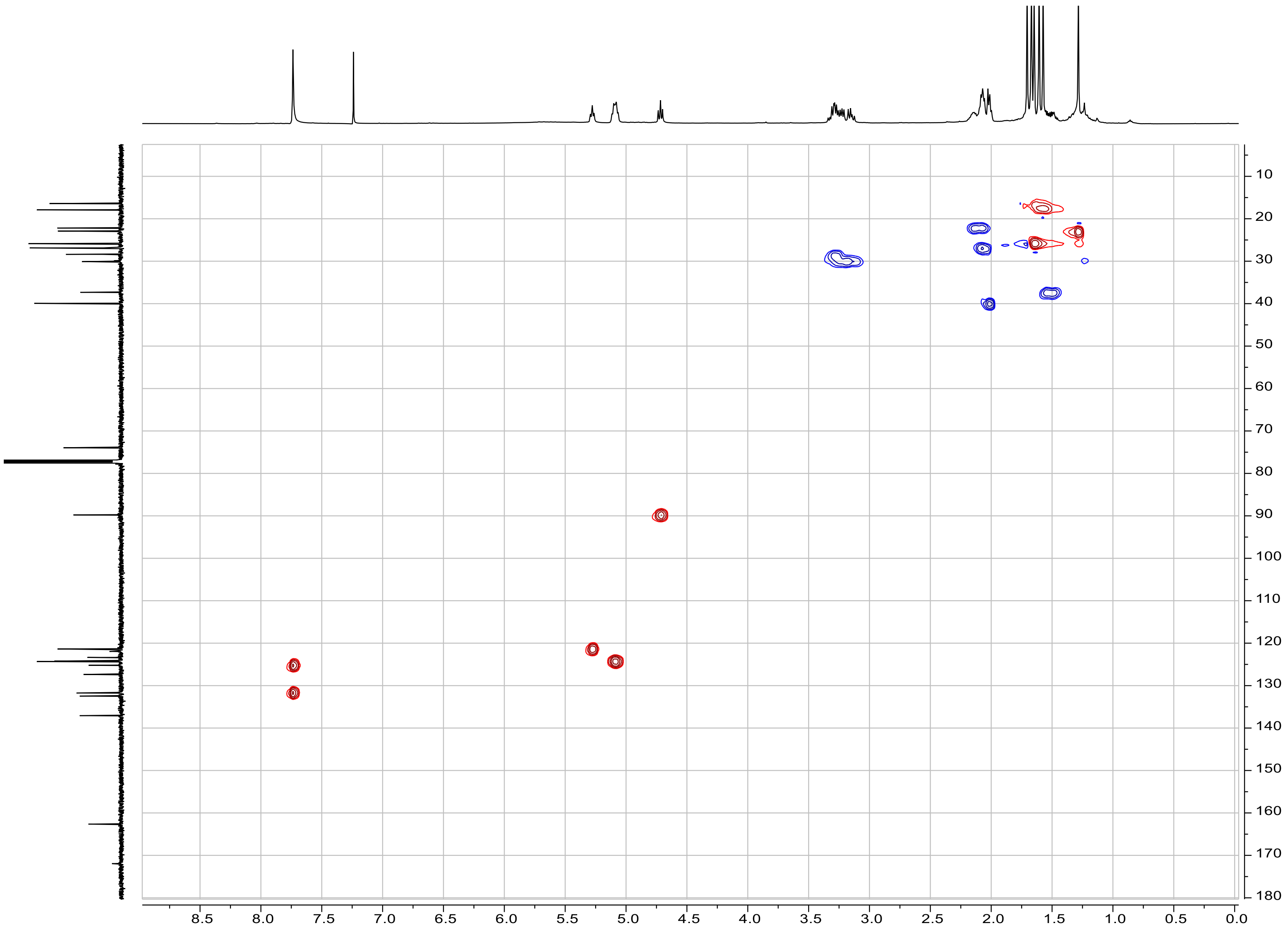

Figure S1.3. HSQC spectrum of compound **1**

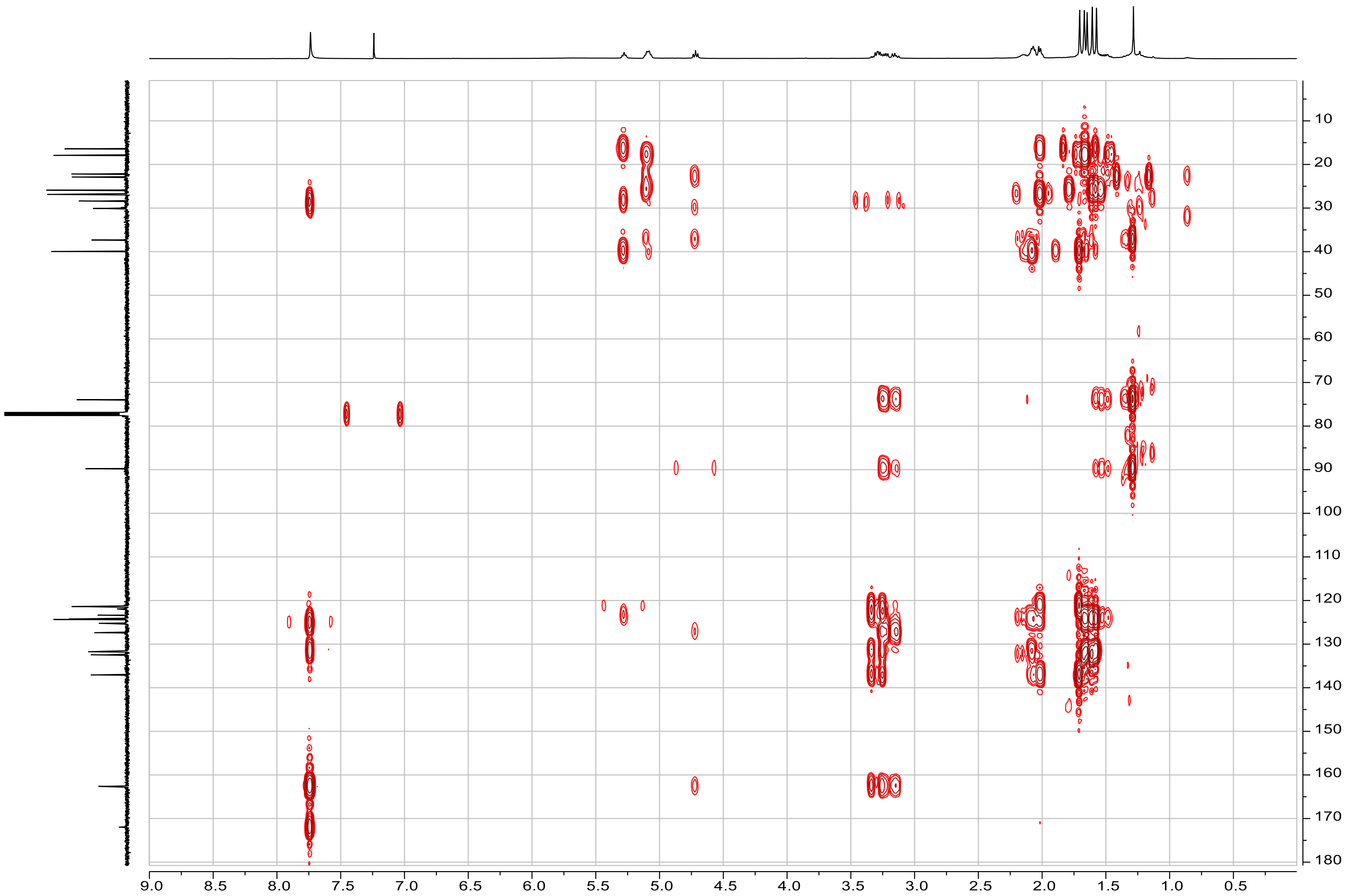

Figure S1.4. HMBC spectrum of compound **1**

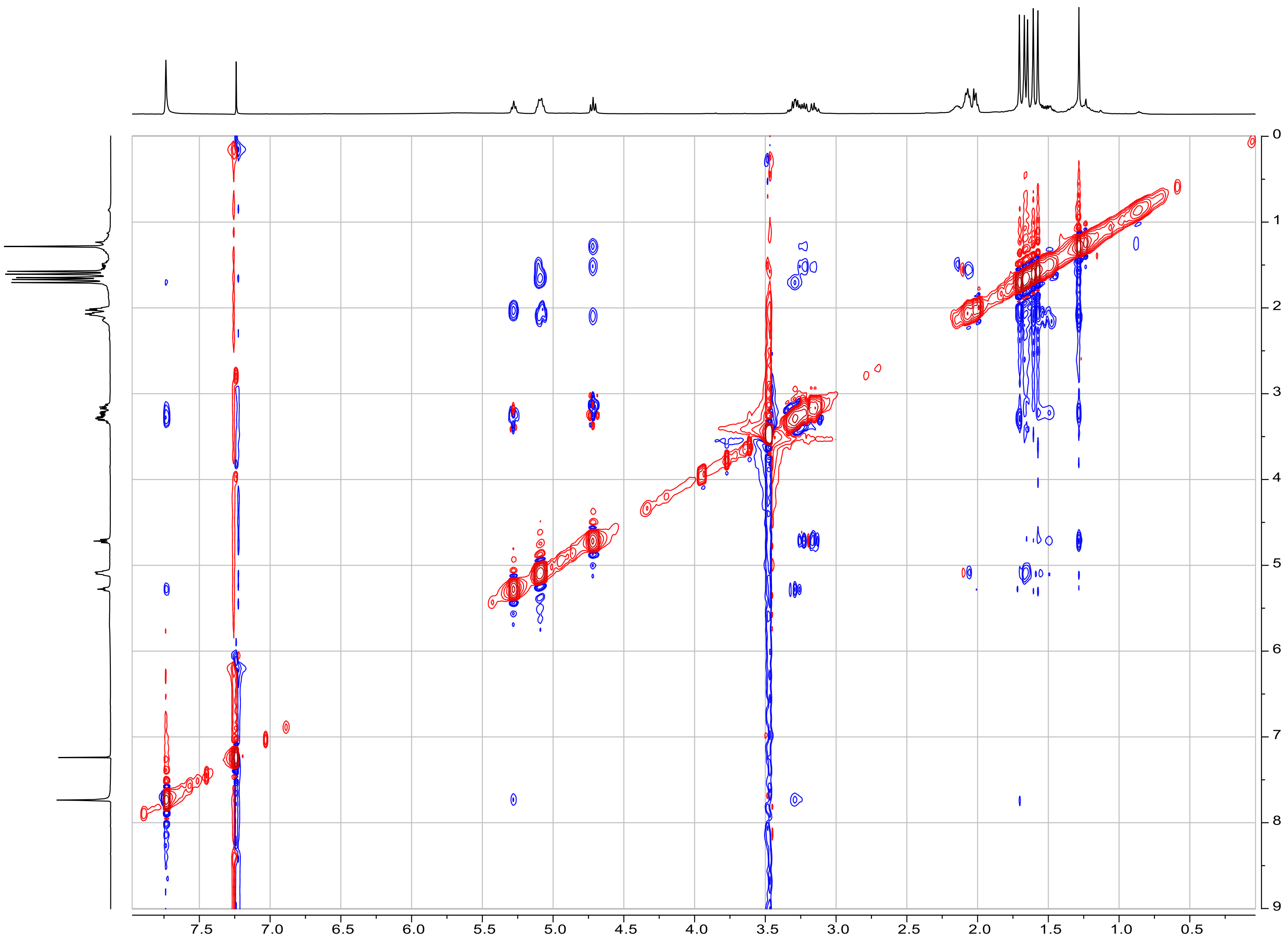

Figure S1.5. NOESY spectrum of compound **1**

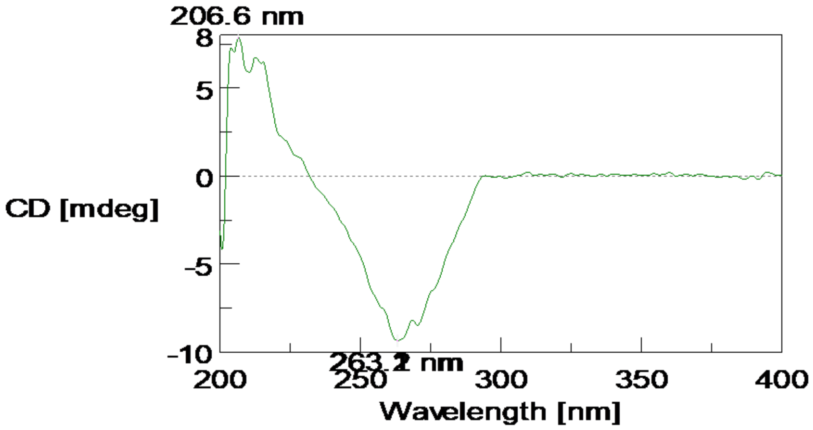

CD

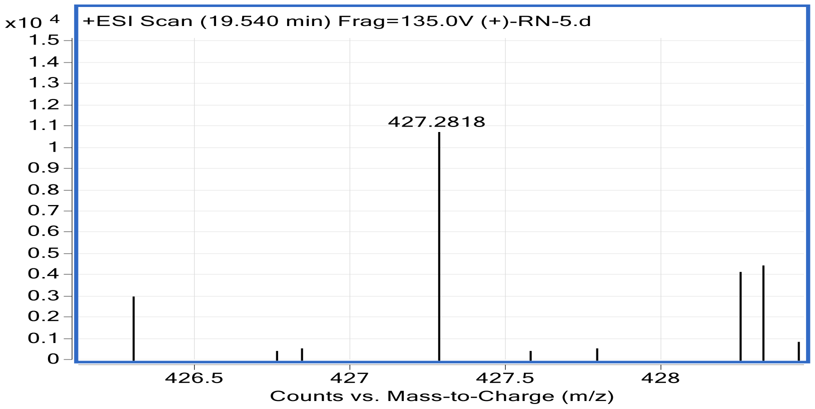

MS

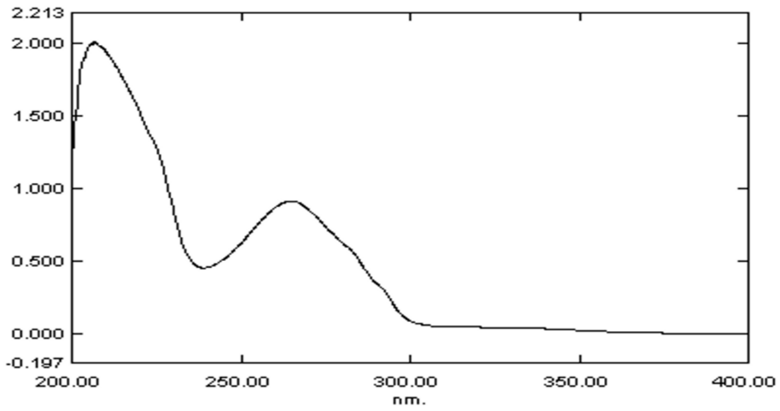

Wavelength nm.	Absorbance
206.60	2.012
264.80	0.916
239.00	0.457

UV

Figure S1.6. CD, MS, and UV spectra of compound **1**

2.2. Compound 2

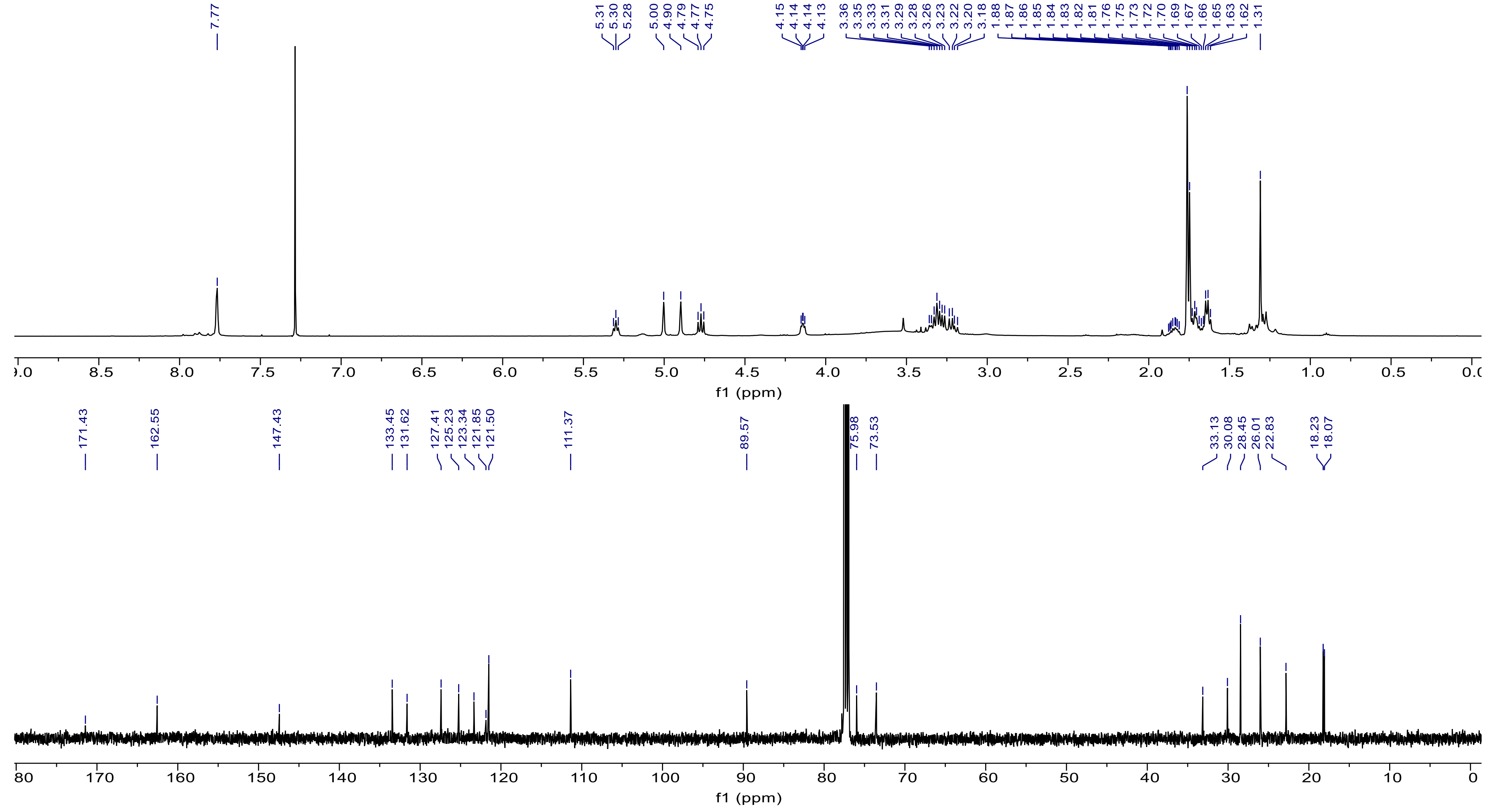

Figure S2.1. ^{1}H (500 MHz) and ^{13}C NMR (125 MHz) spectra of compound **2** in $CDCl_3$

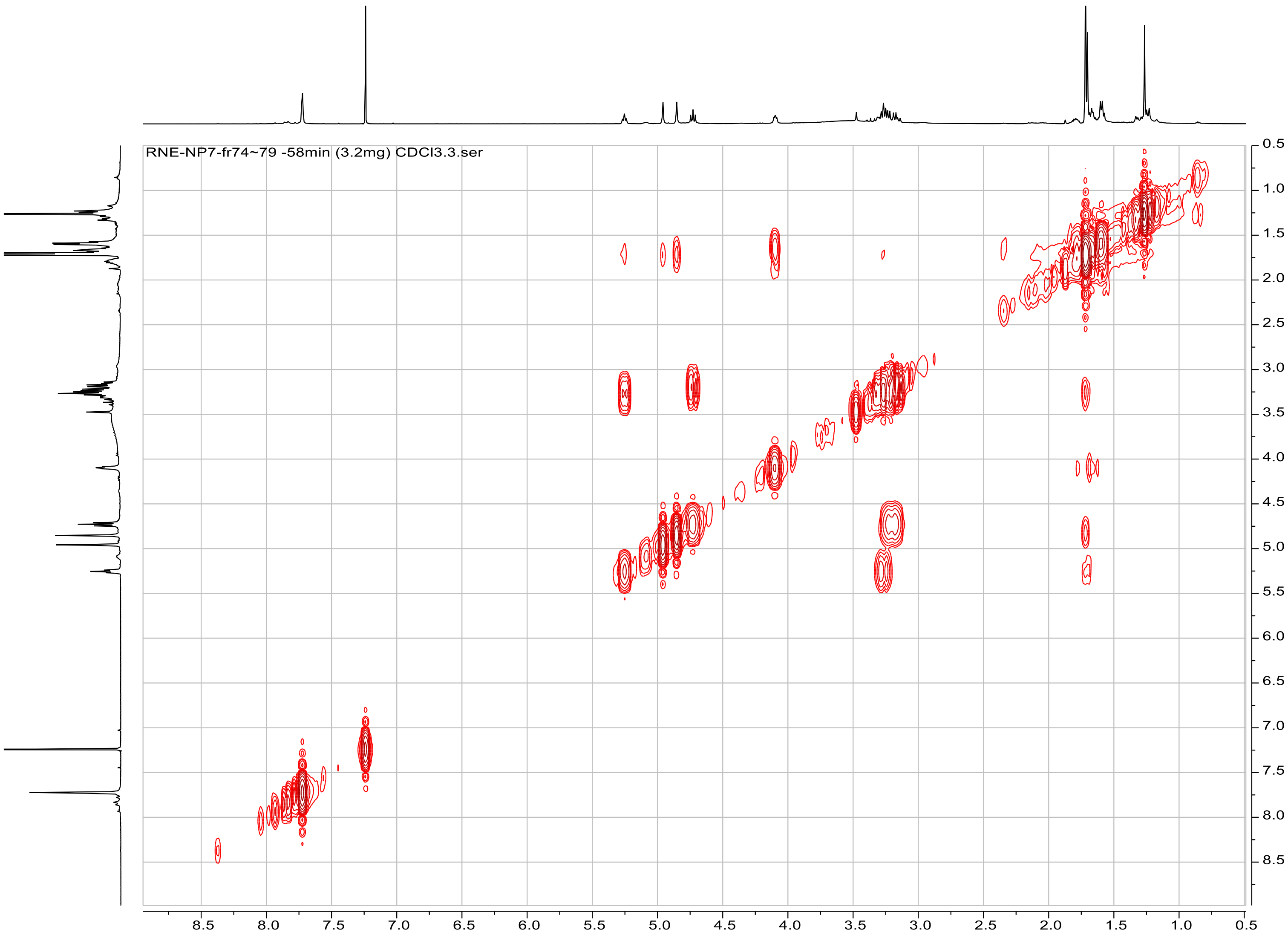

Figure S2.2. ^{1}H-^{1}H COSY spectrum of compound **2**

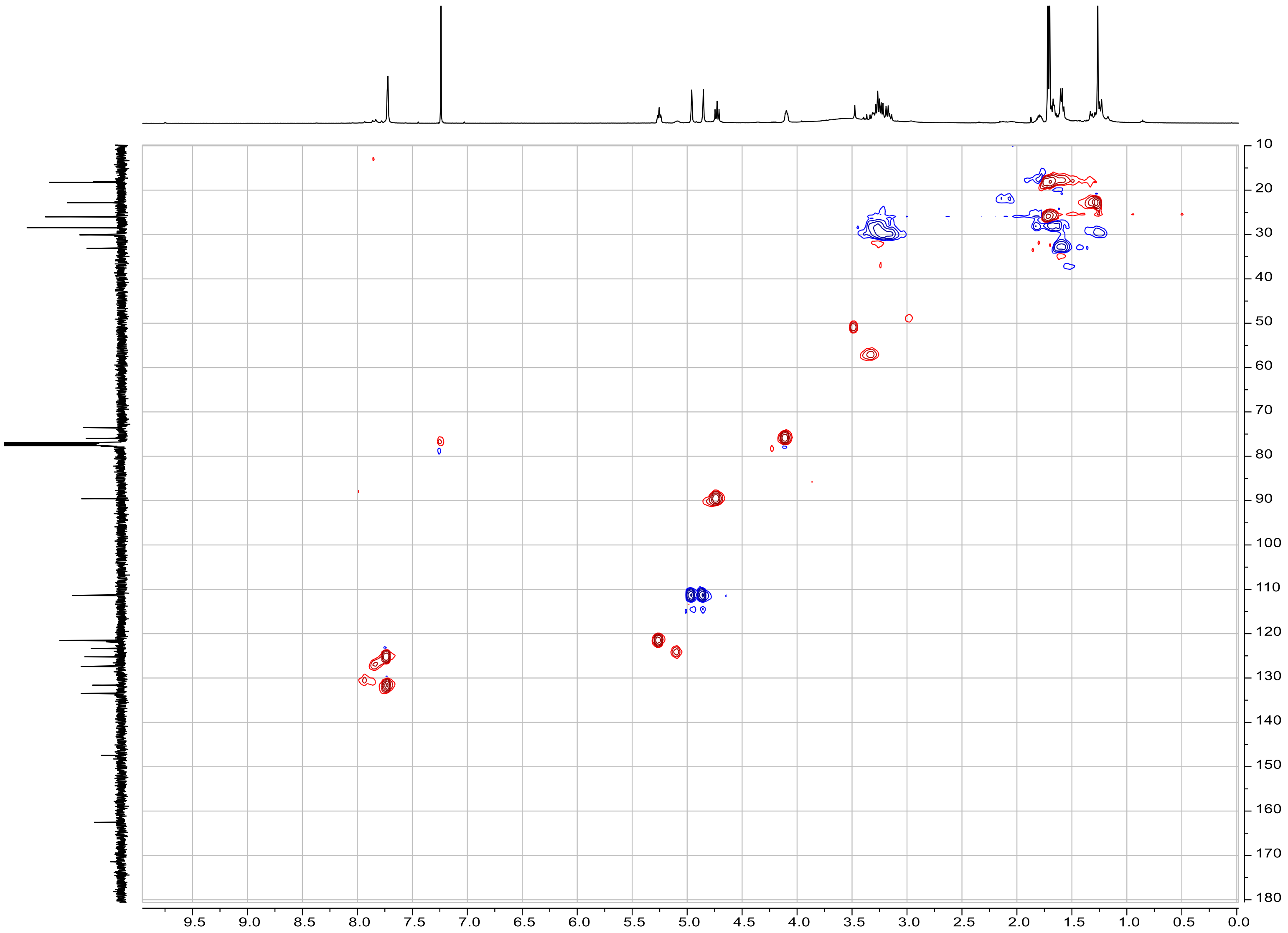

Figure S2.3. HSQC spectrum of compound **2**

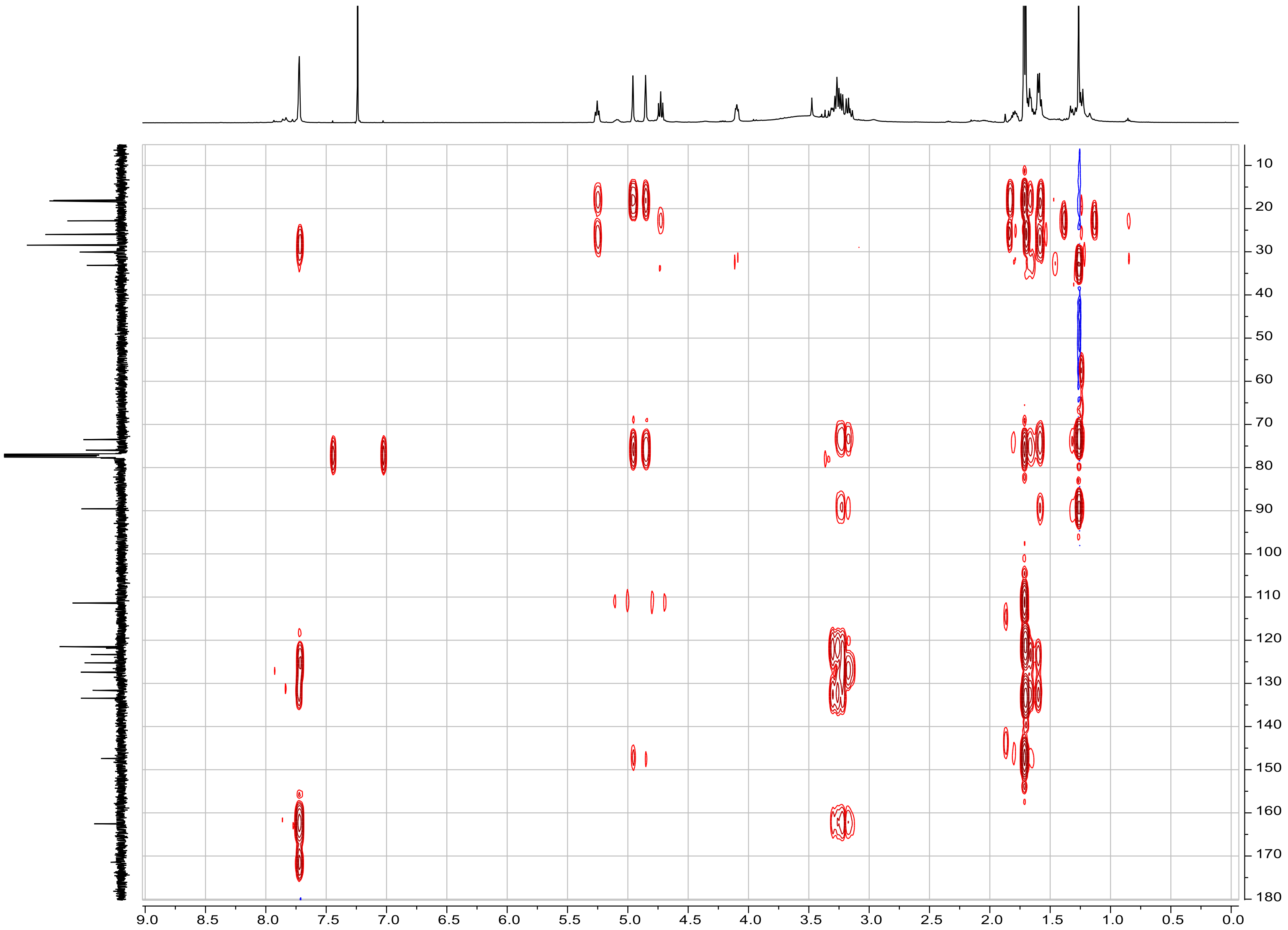

Figure S2.4. HMBC spectrum of compound **2**

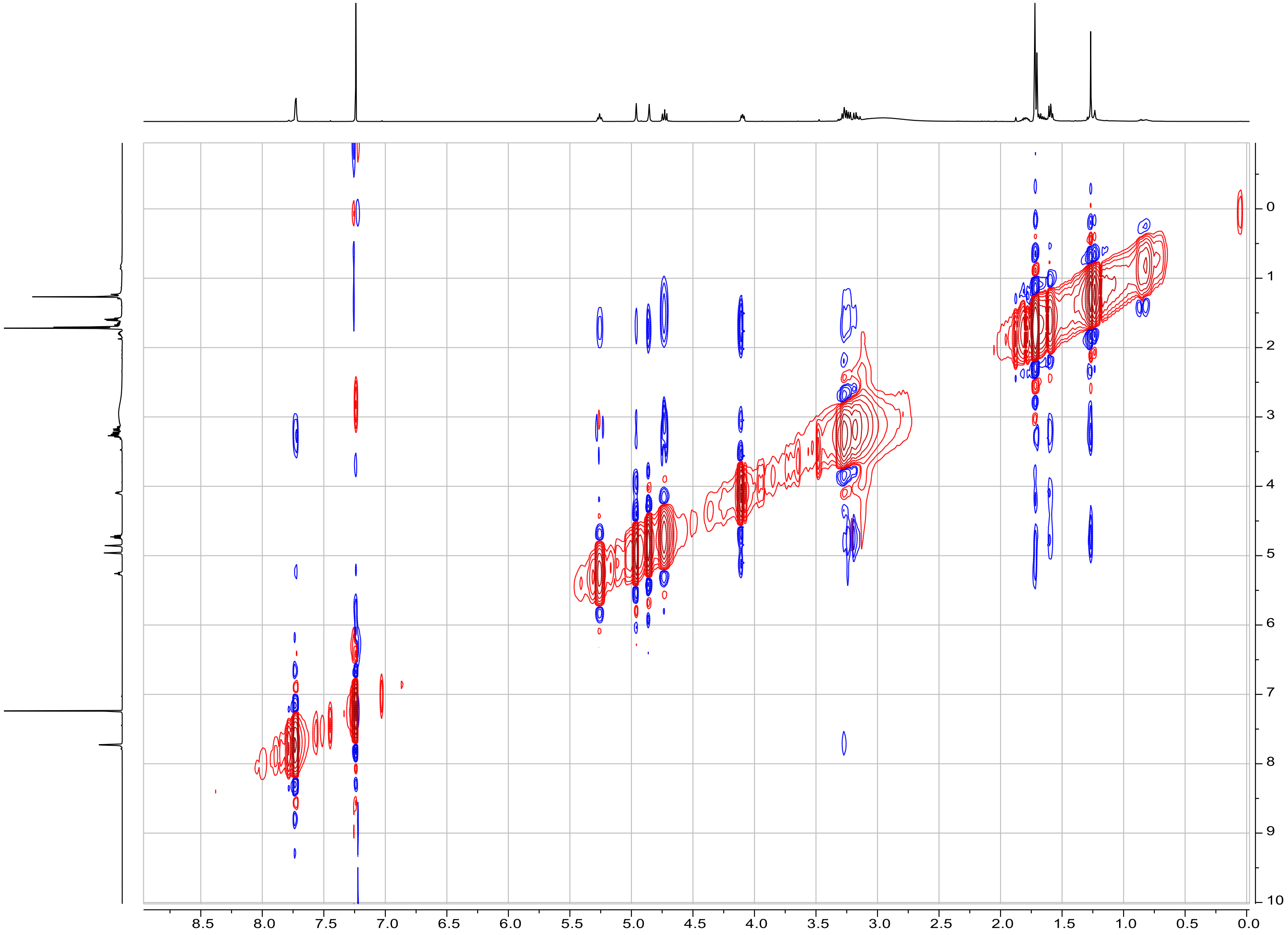

Figure S2.5. NOESY spectrum of compound **2**

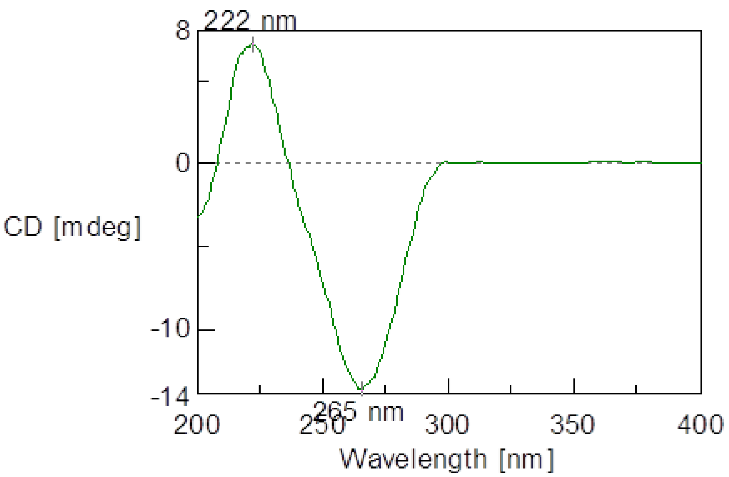

CD

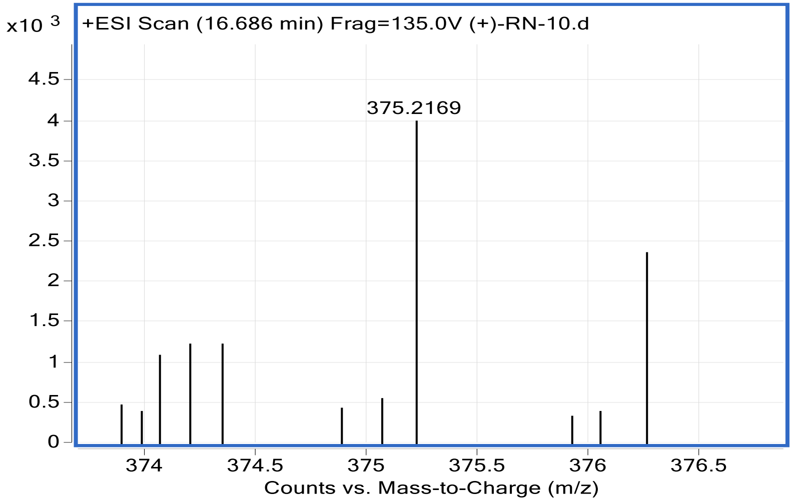

MS

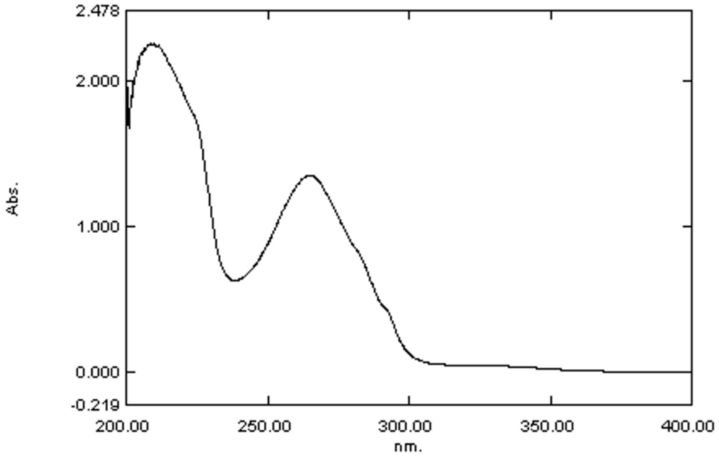

Wavelength nm.	Absorbance
208.40	2.253
264.80	1.348

UV

Figure S2.6. CD, MS, and UV spectra of compound **2**

2.3. Compound 3

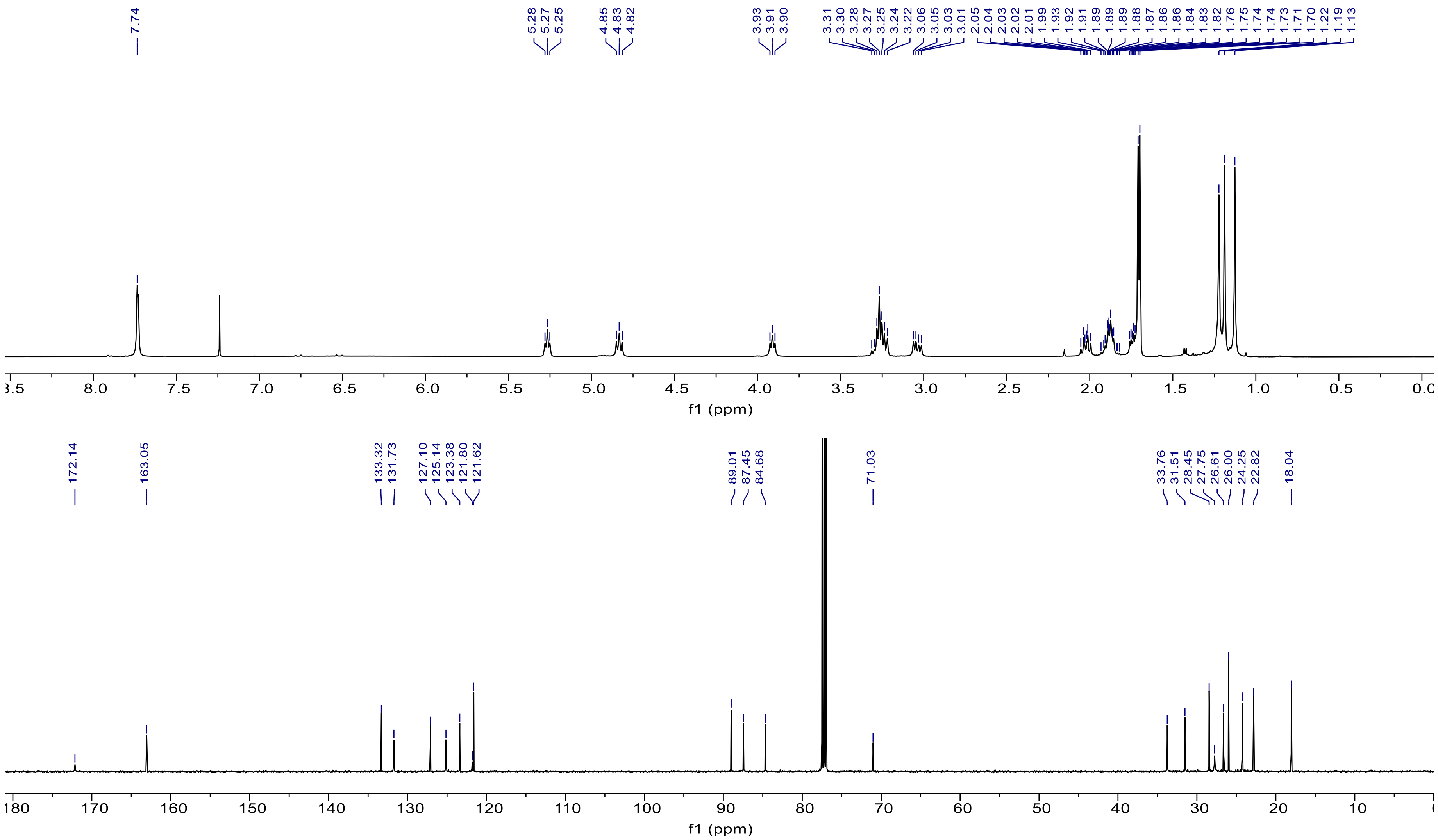

Figure S3.1. ^{1}H (500 MHz) and ^{13}C NMR (125 MHz) spectra of compound **3** in $CDCl_3$

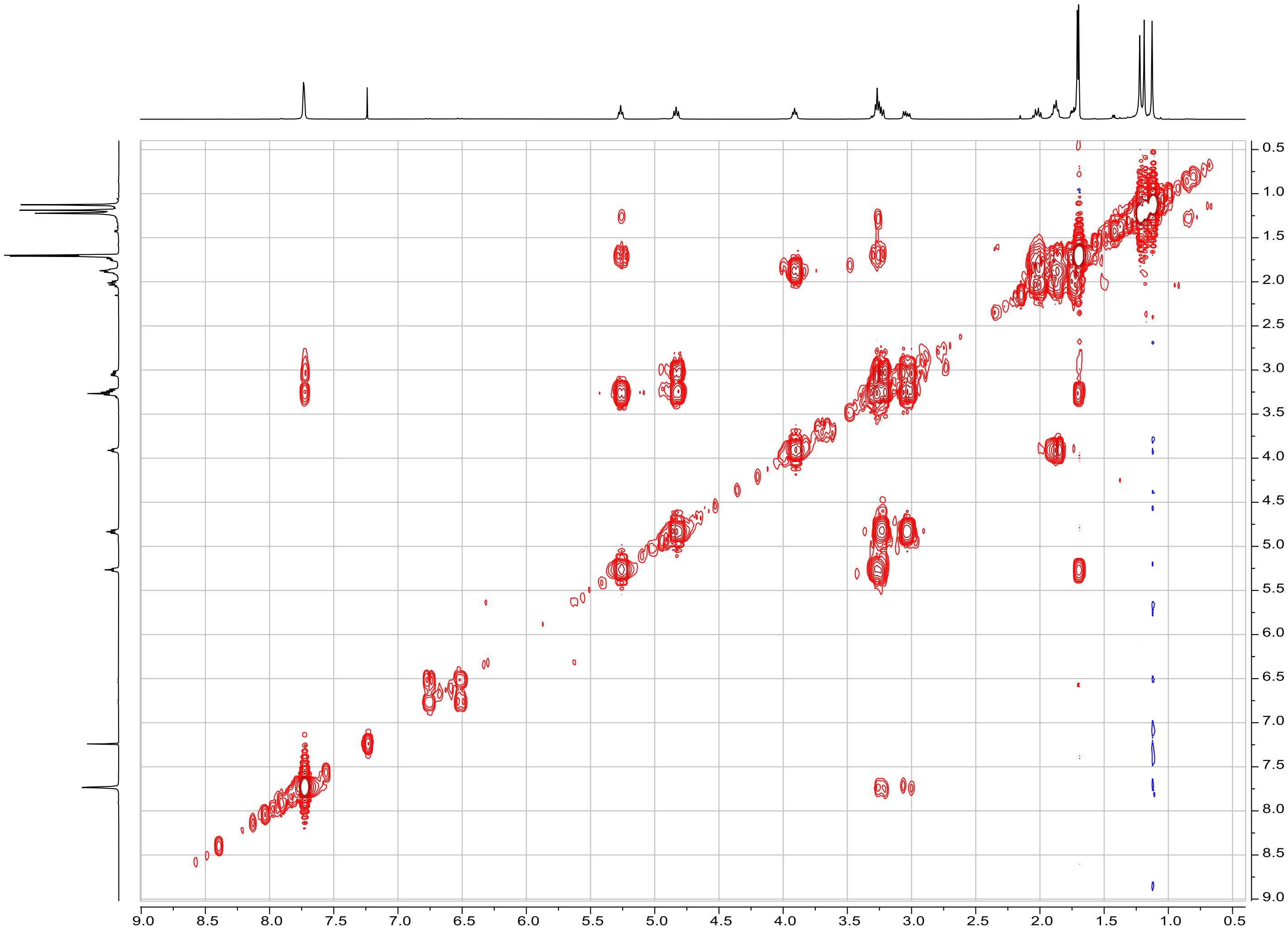

Figure S3.2. ^{1}H-^{1}H COSY spectrum of compound **3**

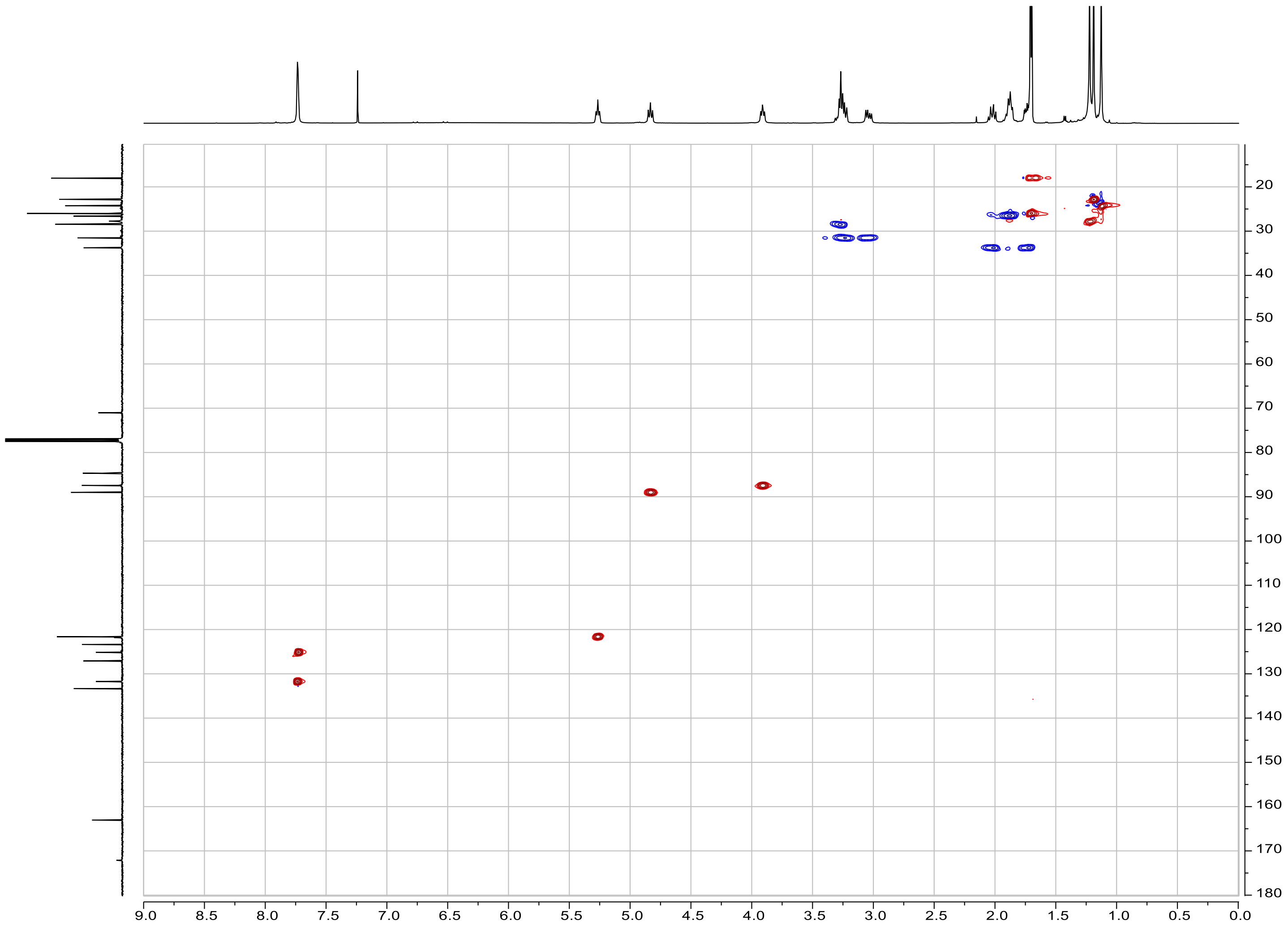

Figure S3.3. HSQC spectrum of compound **3**

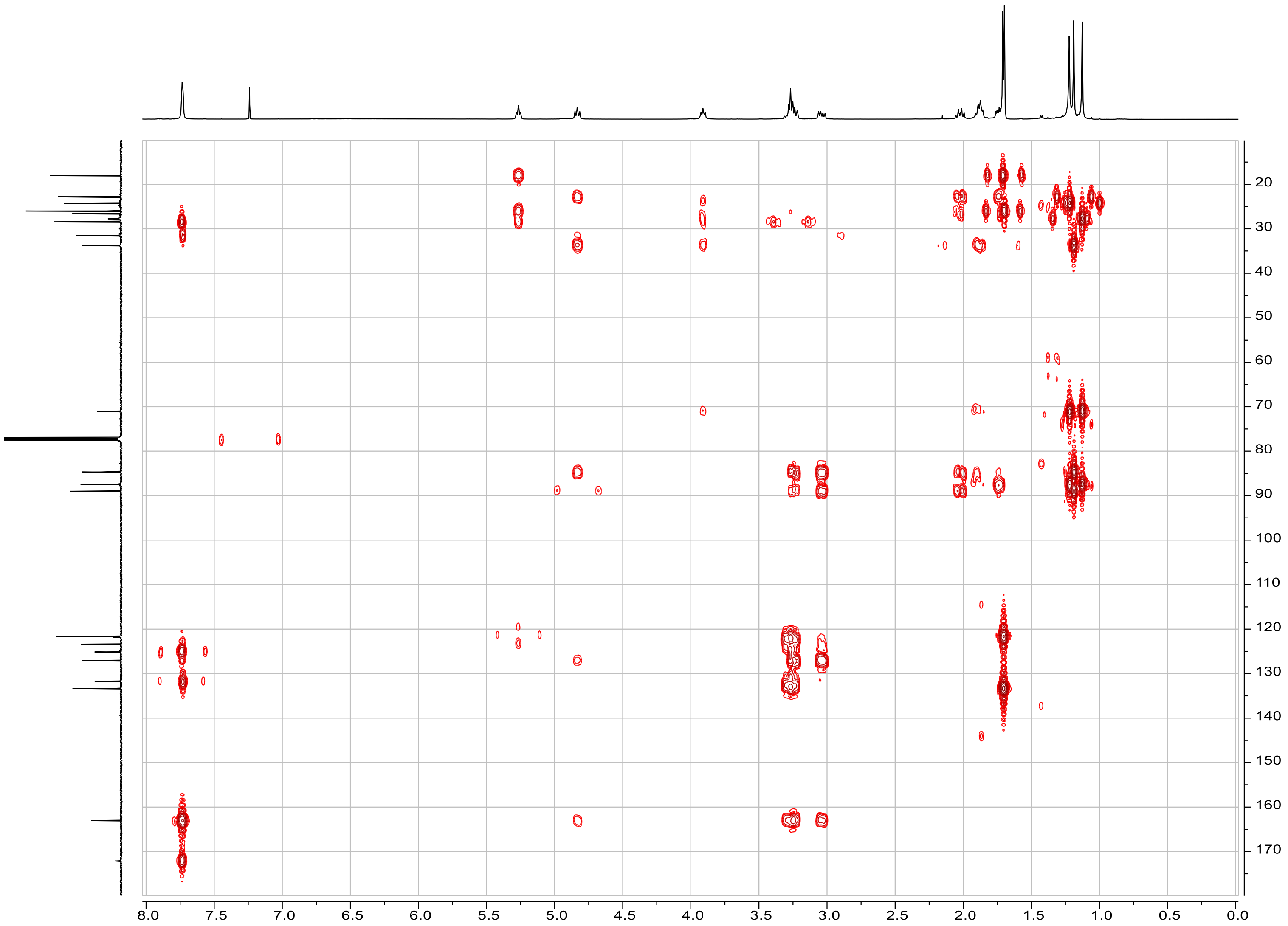

Figure S3.4. HMBC spectrum of compound **3**

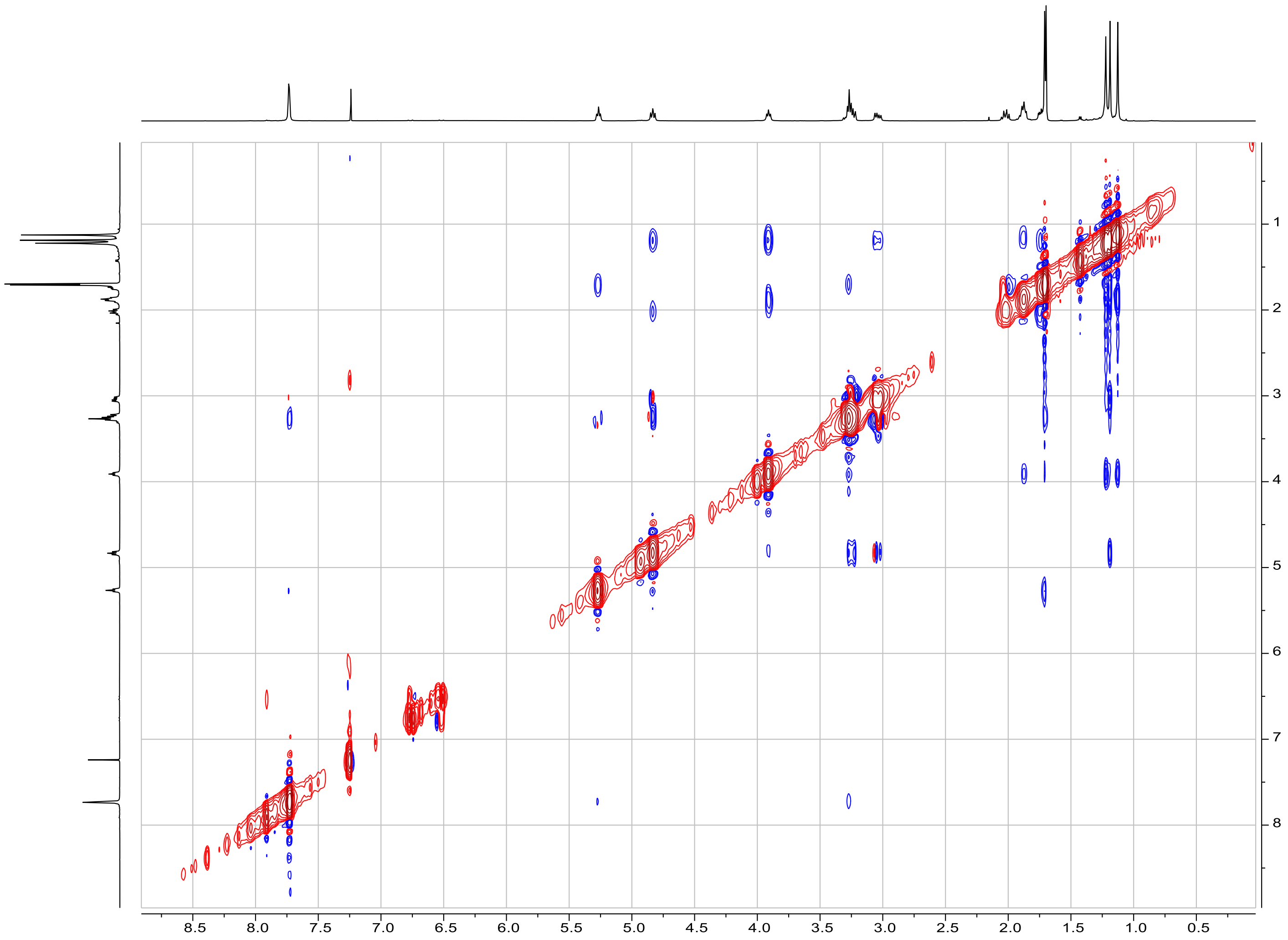

Figure S3.5. NOESY spectrum of compound **3**

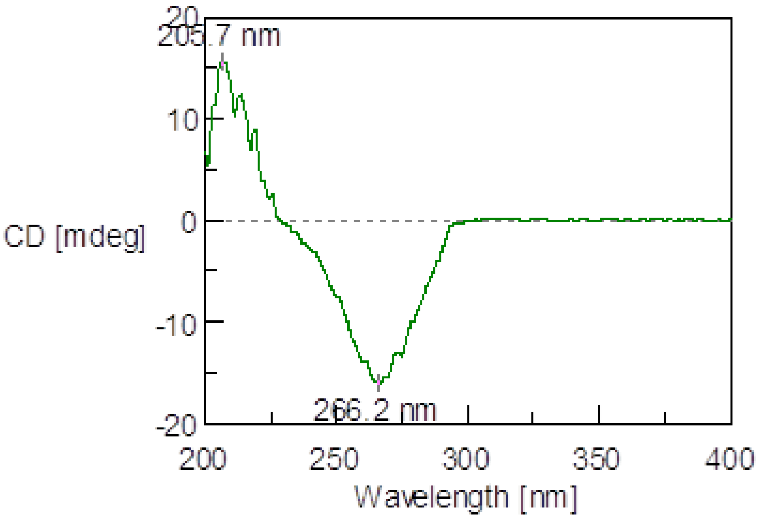

CD

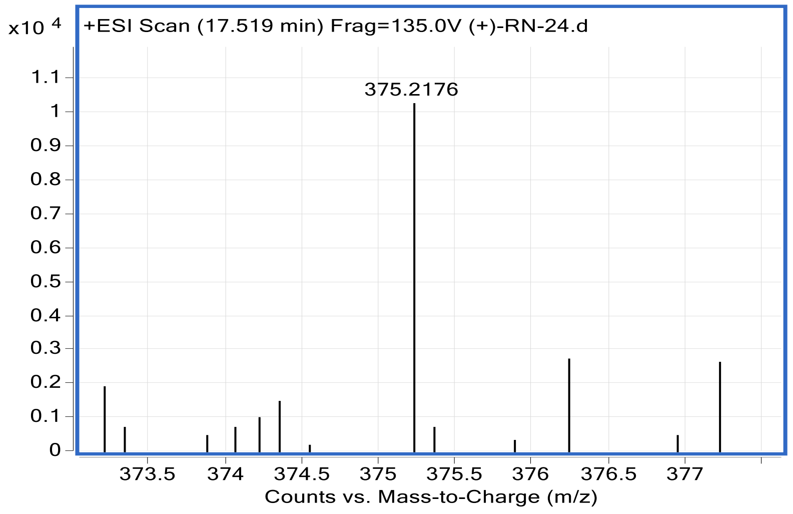

MS

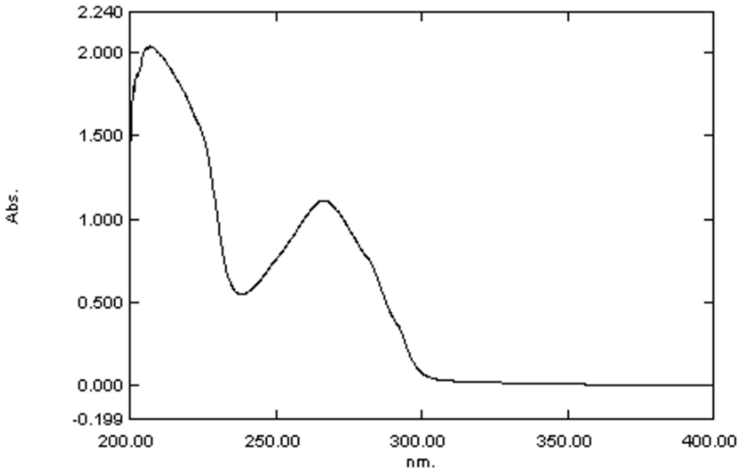

Wavelength nm.	Absorbance
207.00	2.035
266.40	1.109

UV

Figure S3.6. CD, MS, and UV spectra of compound **3**

2.4. Compound 4

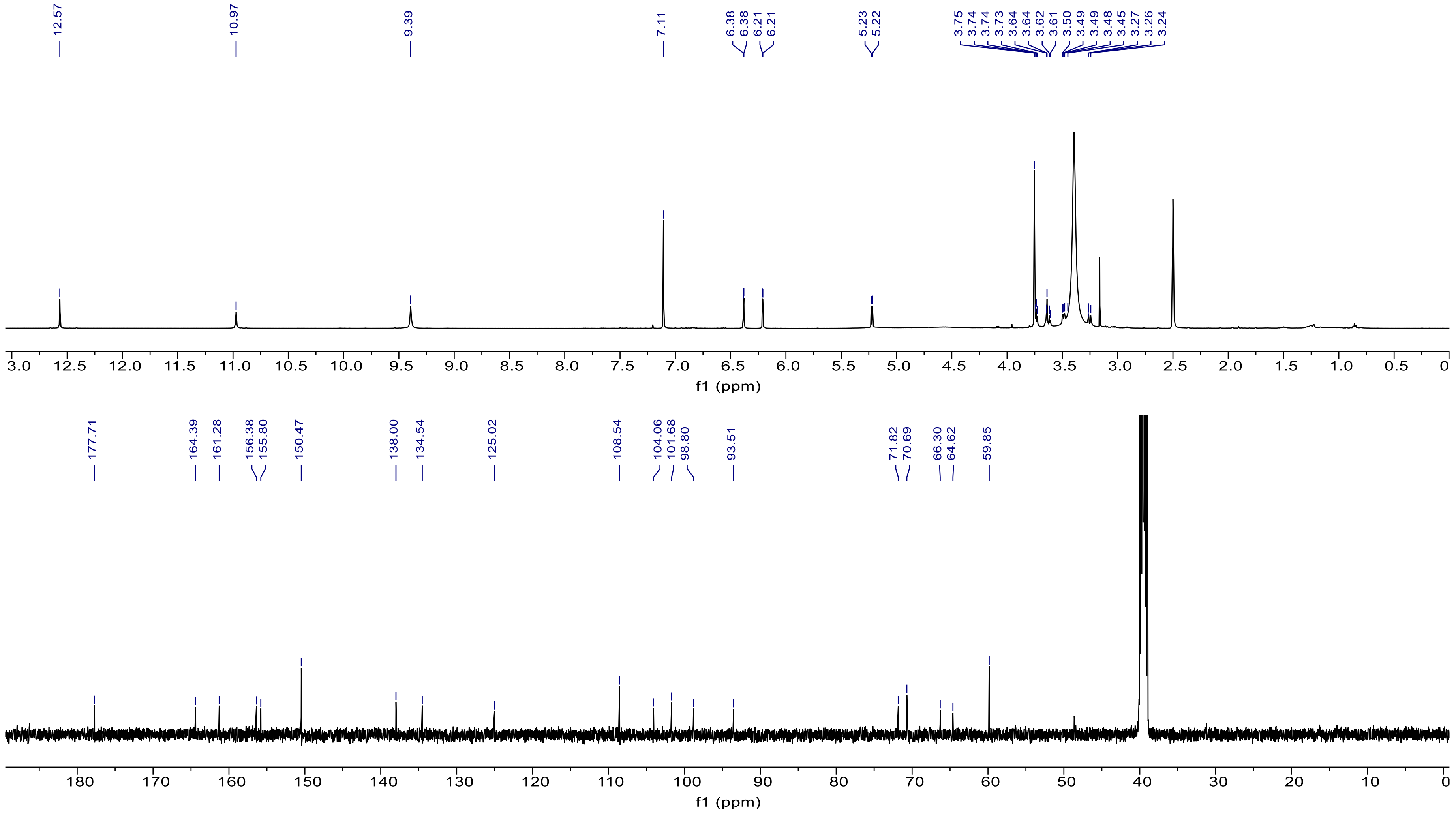

Figure S4.1. ^{1}H (500 MHz) and ^{13}C NMR (125 MHz) spectra of compound **4** in DMSO-d_6

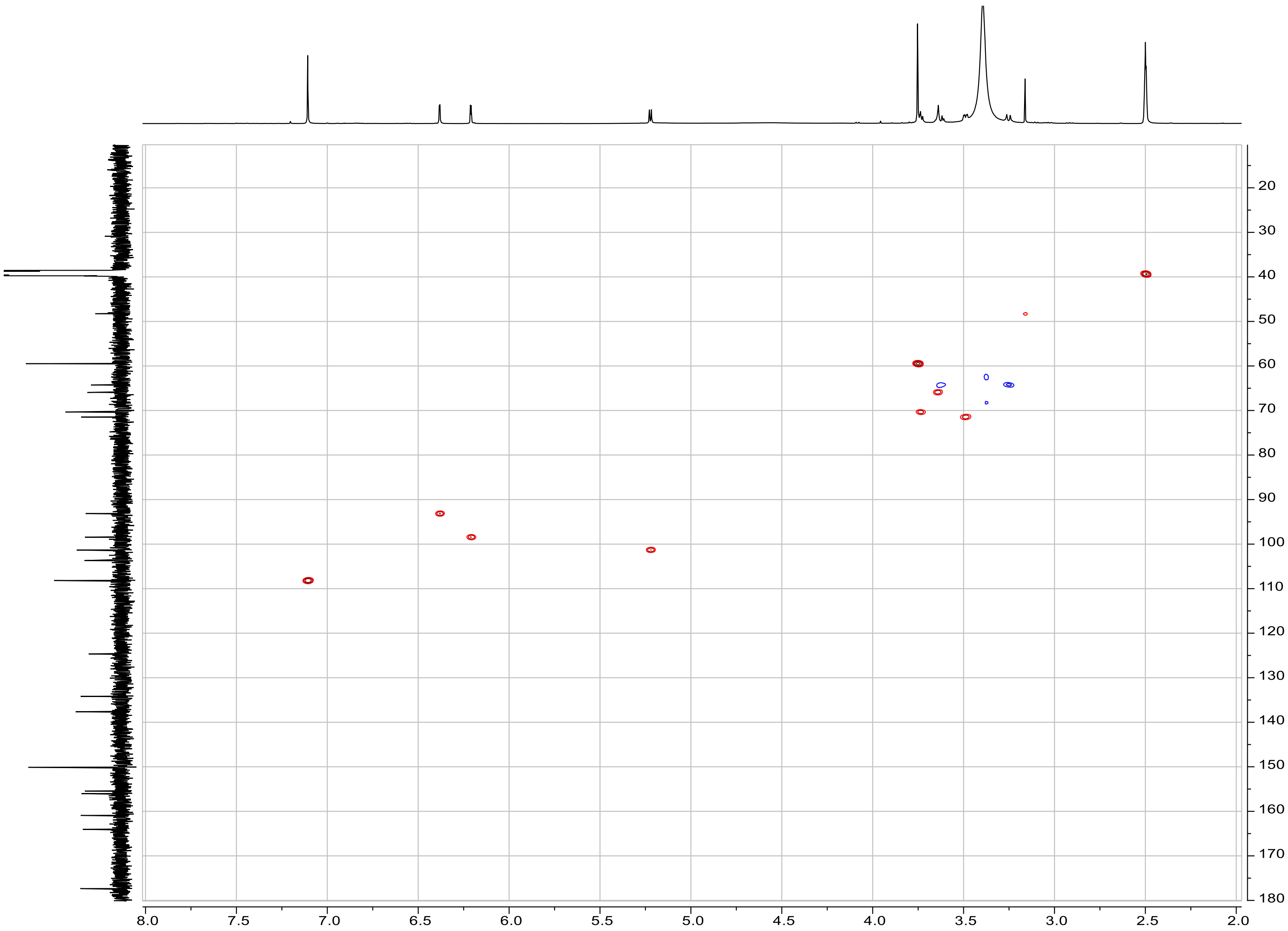

Figure S4.2. HSQC spectrum of compound **4**

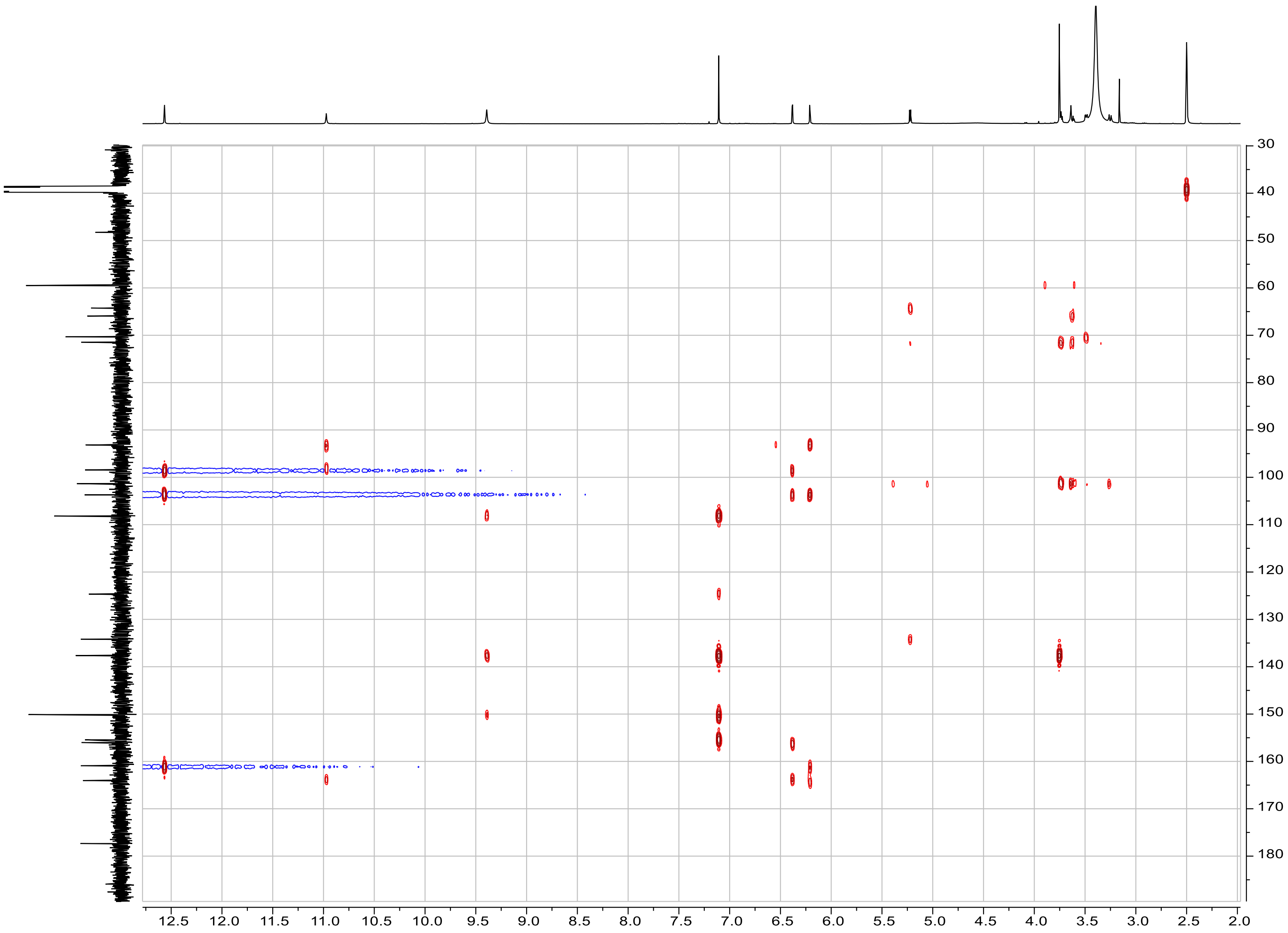

Figure S4.3. HMBC spectrum of compound **4**

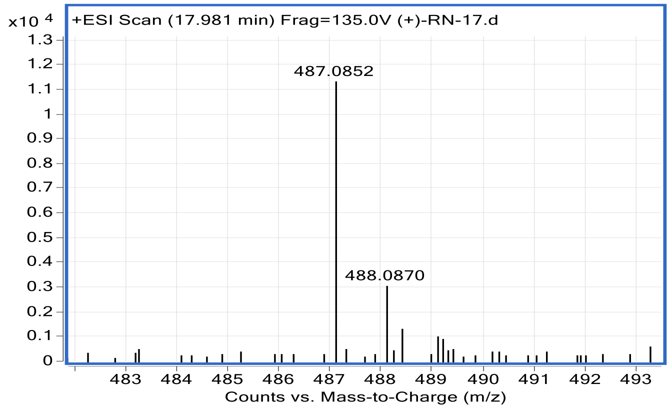

MS

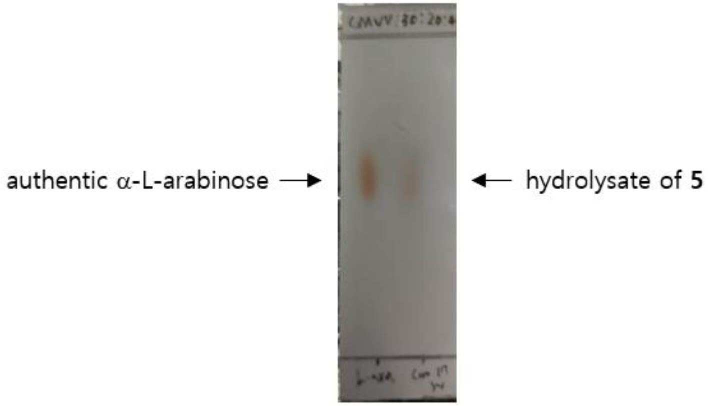

Sugar analysis

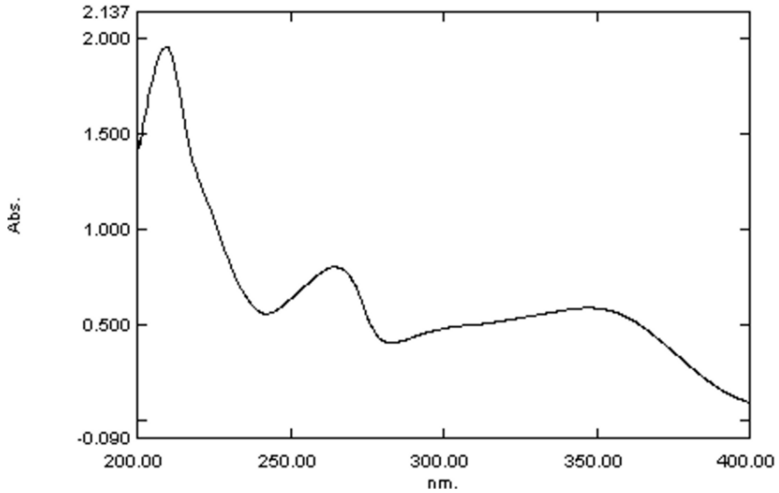

Wavelength nm.	Absorbance
209.40	1.952
264.20	0.804
349.80	0.588

UV

Figure S4.4. MS, UV spectra and sugar analysis by TLC of compound **4**

2.5. Compound 5

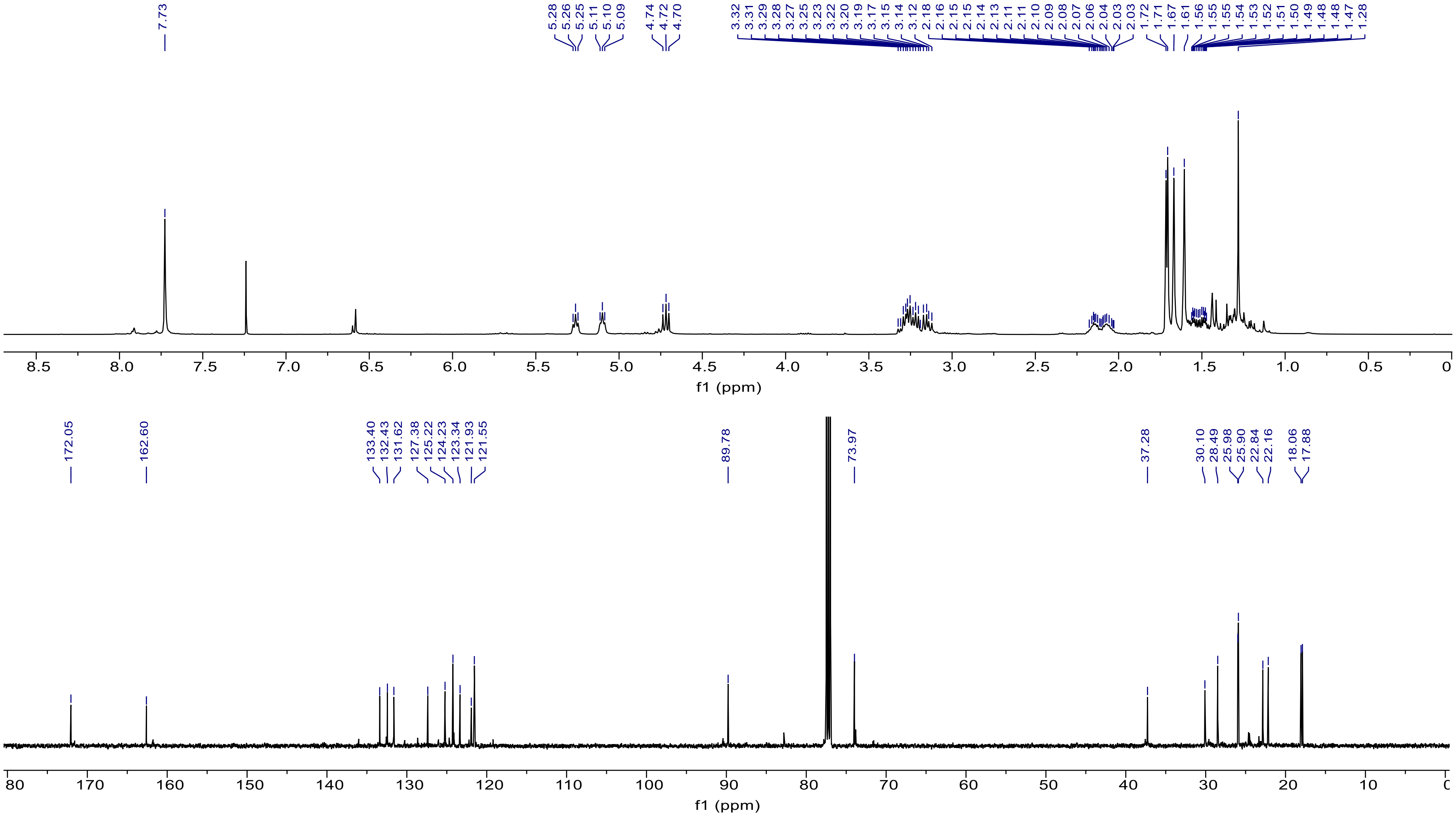

Figure S5.1. ^{1}H (500 MHz) and ^{13}C NMR (125 MHz) spectra of compound 5 in $CDCl_3$

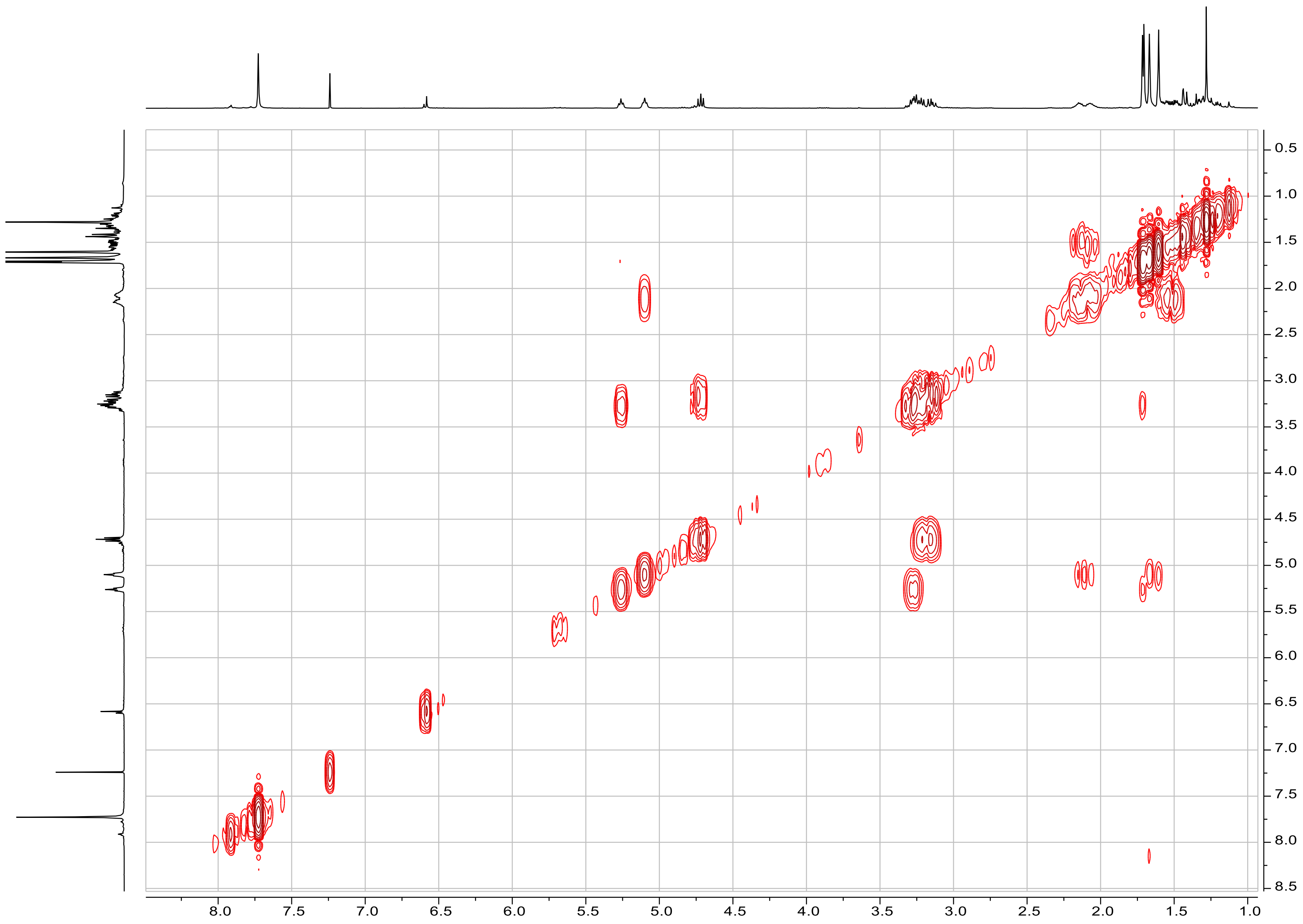

Figure S5.2. ^{1}H-^{1}H COSY spectrum of compound **5**

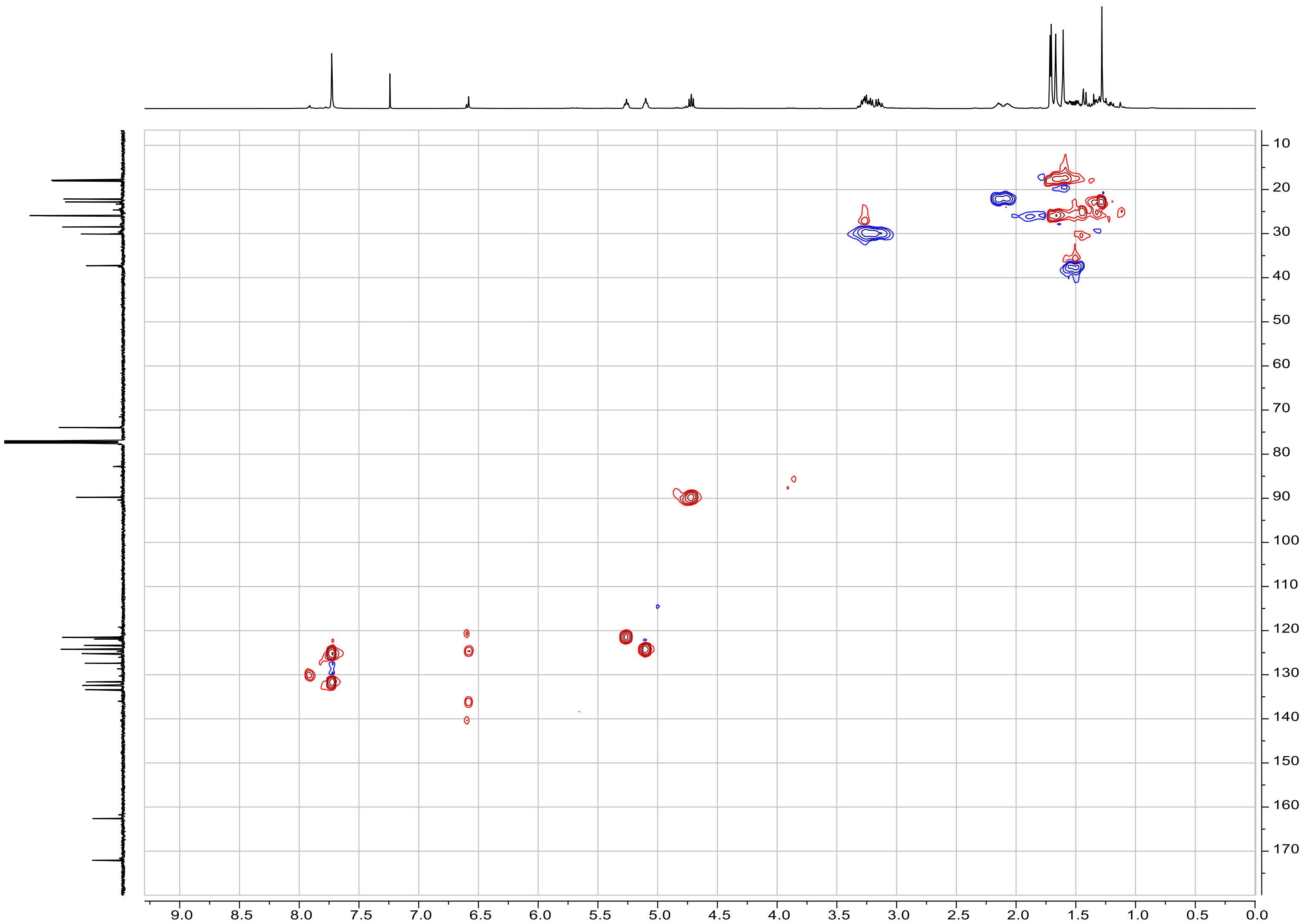

Figure S5.3. HSQC spectrum of compound **5**

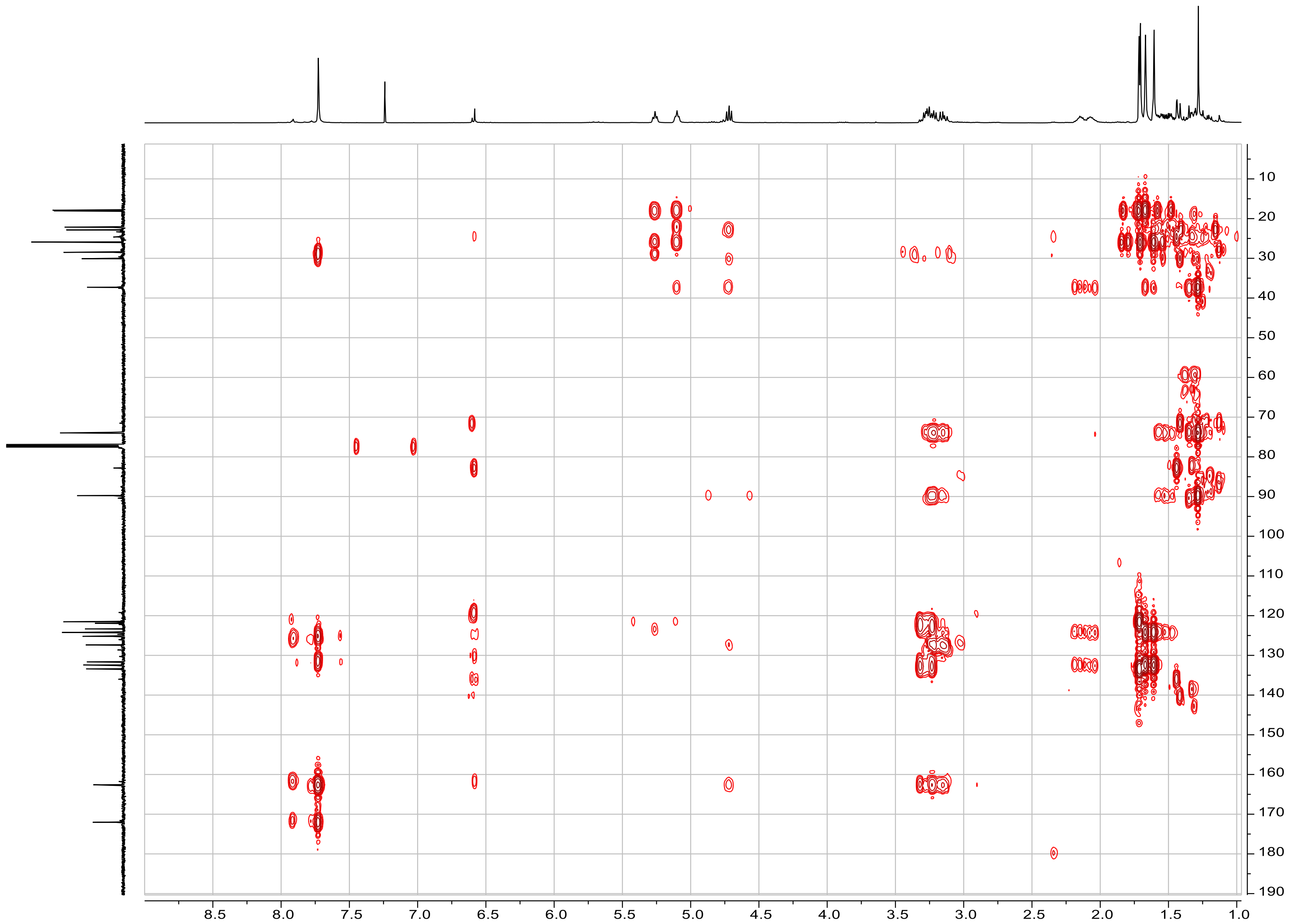

Figure S5.4. HMBC spectrum of compound **5**

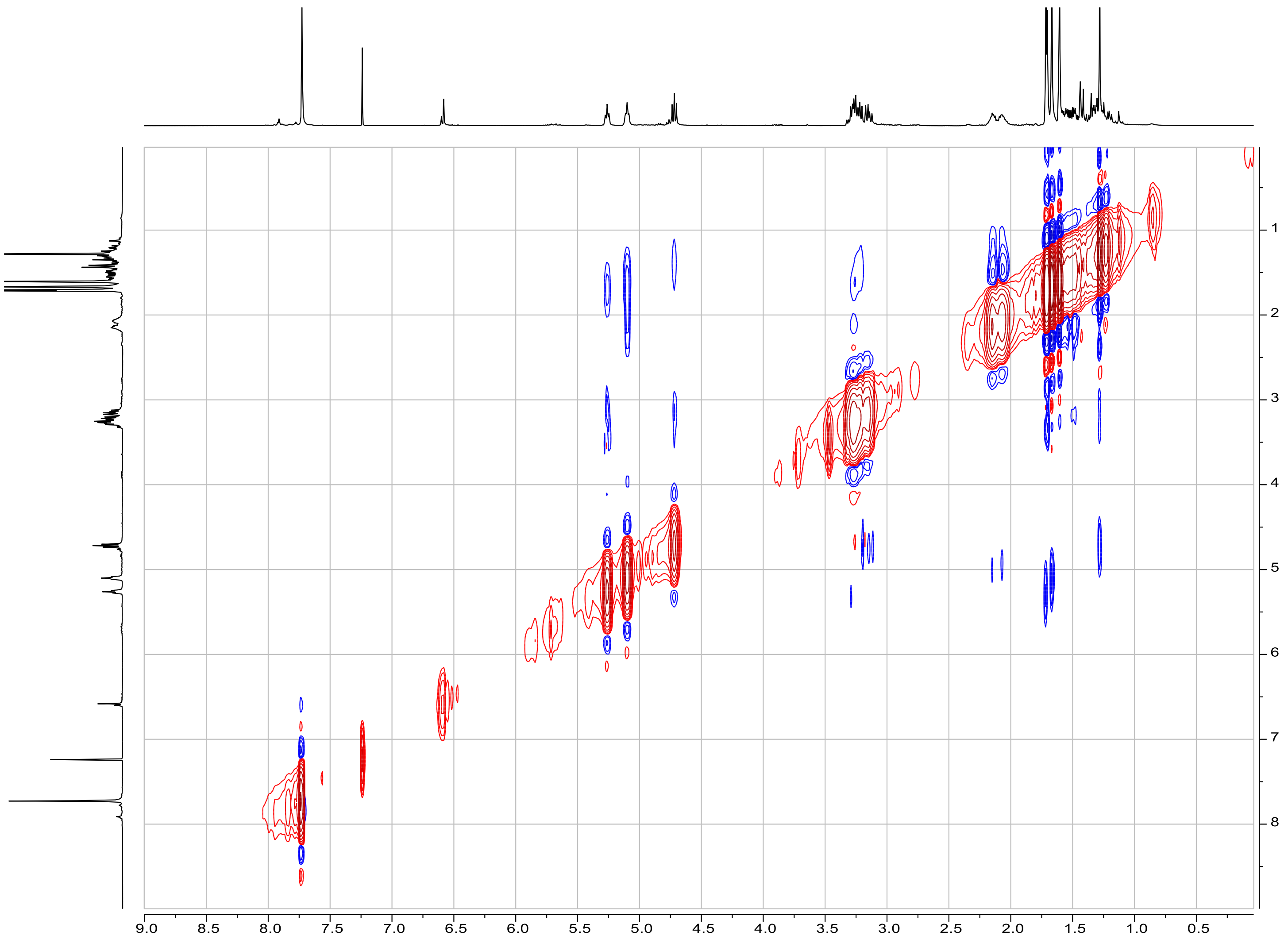

Figure S5.5. NOESY spectrum of compound **5**

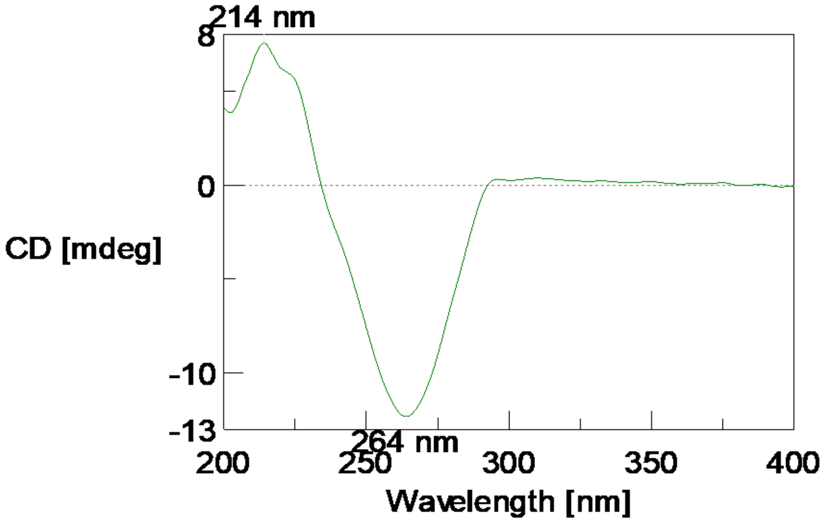

CD

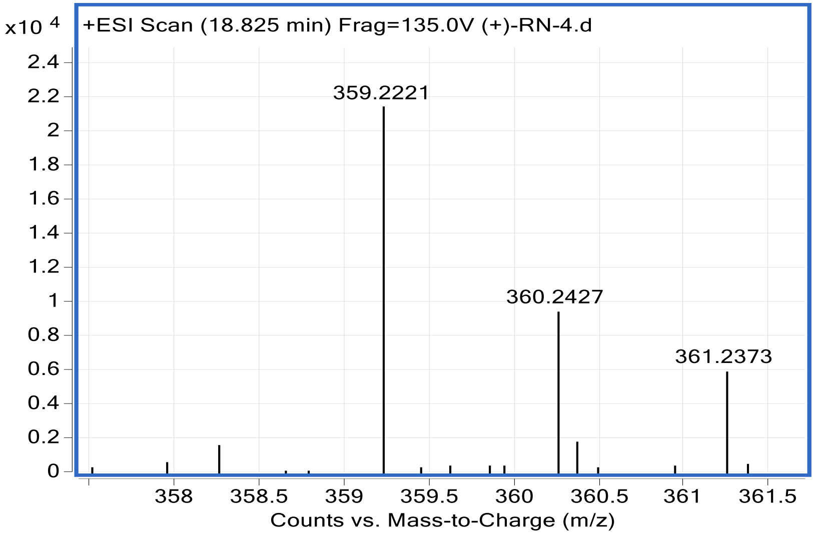

MS

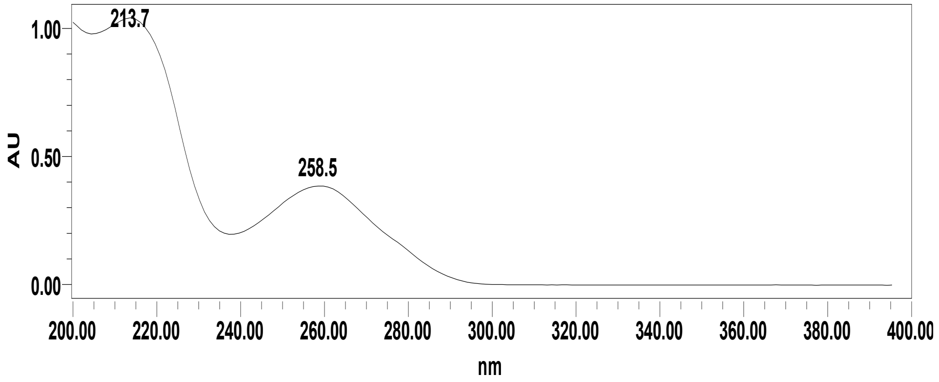

HPLC-PDA

Figure S5.6. CD, MS, and HPLC-PDA spectra of compound **5**

3. Computational data of compounds 1-3,5

3.1. Compound 1

Table S1.1. Detailed DP4+ probability of **1a** (Isomer 1) and **1b** (Isomer 2)

Functional	Solvent?		Basis Set		Type of Data	
B3LYP	PCM		6-31+G(d,p)		Shielding Tensors	
	Isomer 1	Isomer 2	Isomer 3	Isomer 4	Isomer 5	Isomer 6
sDP4+ (H data)	99.62%	0.38%	-	-	-	-
sDP4+ (C data)	98.65%	1.35%	-	-	-	-
sDP4+ (all data)	99.99%	0.01%	-	-	-	-
uDP4+ (H data)	99.90%	0.10%	-	-	-	-
uDP4+ (C data)	99.86%	0.14%	-	-	-	-
uDP4+ (all data)	100.00%	0.00%	-	-	-	-
DP4+ (H data)	100.00%	0.00%	-	-	-	-
DP4+ (C data)	100.00%	0.00%	-	-	-	-
DP4+ (all data)	100.00%	0.00%	-	-	-	-

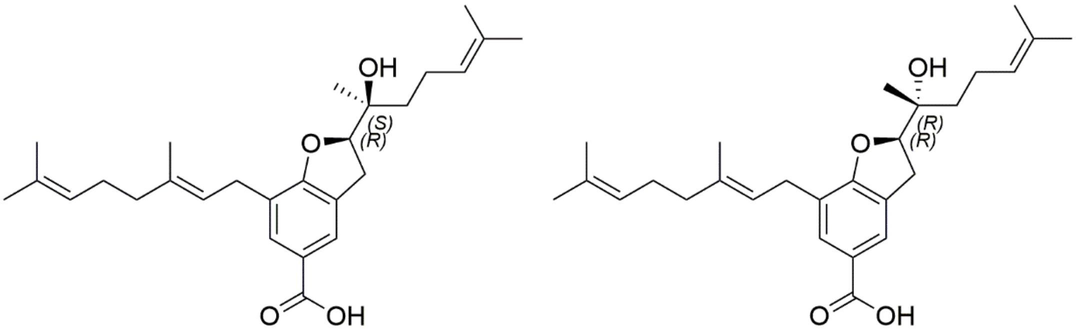

2*R*,1'*S*-**1** (**1a**) 2*R*,1'*R*-**1** (**1b**)

Table S1.2. Experimental and calculated ^{1}H-NMR chemical shifts (δ in ppm) of **1a** and **1b**

**Proton**									
**1**		**1a (2*R*,1'*S*)**				**1b (2*R*,1'*R*)**			
No.	δ_H Exp. (mult., *J* in Hz)	δ_H Calc.	$\|\Delta\delta\|$	δ_H Calc. CORRECTED	$\|\Delta\delta\|$	δ_H Calc.	$\|\Delta\delta\|$	δ_H Calc. CORRECTED	$\|\Delta\delta\|$
2	4.72 (1H, t, 9.0)	4.99	0.27	4.77	0.05	5.09	0.37	4.84	0.12
3	3.19 (2H, m)	3.27	0.08	3.13	0.06	3.32	0.13	3.16	0.03
4	7.74 (1H, s)	8.08	0.34	7.72	0.02	8.10	0.36	7.69	0.05
6	7.74 (1H, s)	8.05	0.31	7.70	0.04	8.03	0.29	7.63	0.11
2'	1.51 (2H, m)	1.47	0.04	1.41	0.10	1.70	0.19	1.61	0.10
3'	2.11(2H, m)	2.19	0.08	2.10	0.01	2.24	0.13	2.12	0.01
4'	5.10 (1H, t, 7.0)	5.43	0.33	5.19	0.09	5.52	0.42	5.24	0.14
6'	1.67 (3H, s)	1.73	0.06	1.66	0.01	1.79	0.12	1.70	0.03
7'	1.28 (3H, s)	1.35	0.07	1.30	0.02	1.14	0.14	1.08	0.20
8'	1.61 (3H, s)	1.71	0.10	1.64	0.03	1.77	0.16	1.68	0.07
1"	3.28 (2H, m)	3.40	0.12	3.26	0.02	3.37	0.09	3.20	0.08
2"	5.28 (1H, t, 7.3)	5.58	0.30	5.34	0.06	5.75	0.47	5.46	0.18
4"	2.01 (2H, m)	2.14	0.13	2.05	0.04	2.14	0.13	2.03	0.02
5"	1.70 (3H, s)	2.26	0.18	2.16	0.08	2.27	0.19	2.15	0.07
1'''	2.08 (2H, m)	5.23	0.14	5.00	0.09	5.15	0.06	4.89	0.20
2'''	5.09 (1H, t, 7.0)	1.64	0.01	1.57	0.08	1.68	0.03	1.60	0.05
4'''	1.65 (3H, s)	1.89	0.19	1.81	0.11	1.81	0.11	1.72	0.02
5'''	1.57 (3H, s)	1.58	0.01	1.51	0.06	1.59	0.02	1.51	0.06
		**MAE**	**0.15**	**CMAE**	**0.05**	**MAE**	**0.19**	**CMAE**	**0.09**

Table S1.3. Experimental and calculated ^{13}C-NMR chemical shifts (δ in ppm) of **1a** and **1b**

**Carbon**									
**1**		**1a (2*R*,1'*S*)**				**1b (2*R*,1'*R*)**			
No.	δC Exp.	δC Calc.	|Δδ|	δC Calc. CORRECTED	|Δδ|	δC Calc.	|Δδ|	δC Calc. CORRECTED	|Δδ|
2	89.77	93.06	3.29	92.52	2.75	90.34	0.57	89.77	0.00
3	30.11	33.88	3.77	30.99	0.88	33.91	3.80	31.06	0.95
3a	127.37	127.40	0.03	128.22	0.85	127.46	0.09	128.40	1.03
4	125.22	123.01	2.21	123.66	1.56	123.20	2.02	123.96	1.26
5	121.94	118.90	3.04	119.38	2.56	118.95	2.99	119.55	2.39
6	131.66	129.78	1.88	130.69	0.97	129.54	2.12	130.57	1.09
7	123.37	123.23	0.14	123.88	0.51	123.44	0.07	124.22	0.85
7a	162.64	159.88	2.76	161.98	0.66	159.94	2.70	162.19	0.45
1'	73.96	76.40	2.44	75.20	1.24	76.11	2.15	74.96	1.00
2'	37.32	40.31	2.99	37.68	0.36	45.02	7.70	42.62	5.30
3'	22.18	27.90	5.72	24.77	2.59	28.72	6.54	25.66	3.48
4'	124.22	123.67	0.55	124.34	0.12	123.36	0.86	124.13	0.09
5'	132.46	133.37	0.91	134.42	1.96	133.27	0.81	134.45	1.99
6'	25.90	27.76	1.86	24.63	1.27	27.76	1.86	24.66	1.24
7'	22.89	25.15	2.26	21.91	0.98	22.16	0.73	18.83	4.06
8'	17.91	18.71	0.80	15.22	2.69	18.90	0.99	15.44	2.47
1"	28.38	33.99	5.61	31.11	2.73	34.10	5.72	31.25	2.87
2"	121.37	123.30	1.93	123.96	2.59	122.68	1.31	123.43	2.06
3"	137.07	137.16	0.09	138.36	1.29	137.06	0.01	138.39	1.32
4"	39.94	44.61	4.67	42.15	2.21	44.76	4.82	42.34	2.40
5"	16.43	31.02	4.15	28.02	1.15	30.38	3.51	27.39	0.52
1'''	26.87	123.41	0.93	124.07	0.27	123.31	1.03	124.08	0.26
2'''	124.34	133.65	1.90	134.72	2.97	133.67	1.92	134.86	3.11
3'''	131.75	27.32	1.45	24.18	1.69	27.23	1.36	24.10	1.77
4'''	25.87	17.17	0.74	13.62	2.81	16.59	0.16	13.03	3.40
5'''	17.89	18.87	0.98	15.39	2.50	19.10	1.21	15.65	2.24
COOH	171.96	163.45	8.51	165.70	6.26	163.43	8.53	165.83	6.13
		**MAE**	**2.43**	**CMAE**	**1.79**	**MAE**	**2.43**	**CMAE**	**1.99**

Table S1.4. Calculated conformational analysis of the **1a** at B3LYP/6-31g(d) level

conformer	3D conformer	G (Hartree)	ΔG (kcal/mol)	Population
1a-1	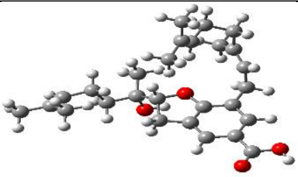	-1352.114208	0	12.995%
1a-2	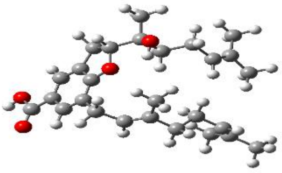	-1352.114121	0.05459337	11.851%
1a-3	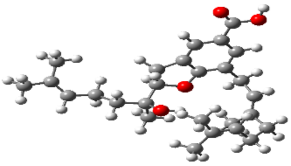	-1352.11404	0.10542168	10.876%
1a-4	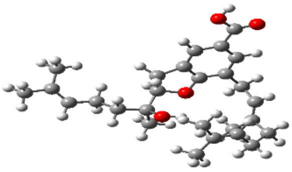	-1352.114027	0.11357931	10.728%
1a-5	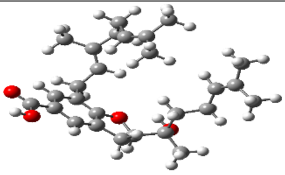	-1352.113973	0.14746485	10.131%
1a-6	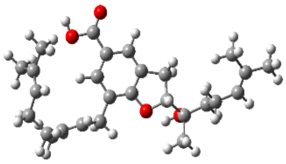	-1352.113724	0.30371484	7.783%
1a-7	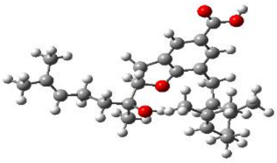	-1352.113228	0.6149598	4.602%
1a-8	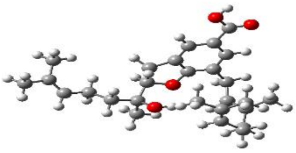	-1352.113228	0.6149598	4.602%

1a-9	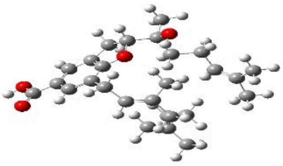	-1352.112965	0.77999493	3.483%
1a-10	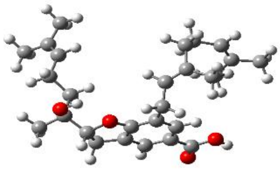	-1352.112856	0.84839352	3.103%
1a-11	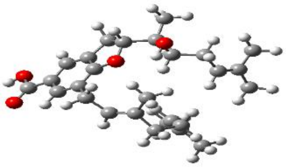	-1352.112839	0.85906119	3.048%
1a-12	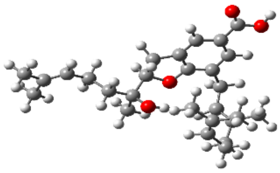	-1352.112543	1.04480415	2.228%
1a-13	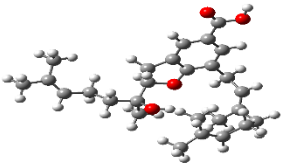	-1352.112346	1.16842362	1.808%
1a-14	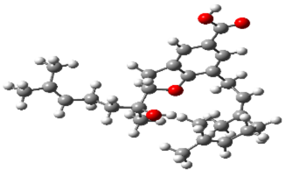	-1352.112272	1.21485936	1.672%
1a-15	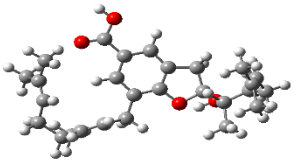	-1352.112231	1.24058727	1.601%
1a-16	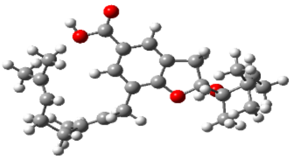	-1352.112099	1.32341859	1.392%
1a-17	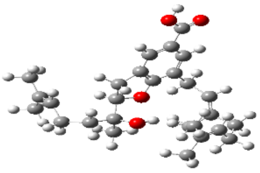	-1352.112038	1.3616967	1.305%

1a-18	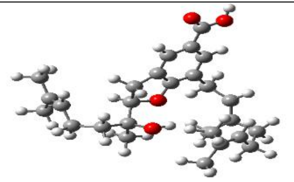	-1352.112032	1.36546176	1.297%
1a-19	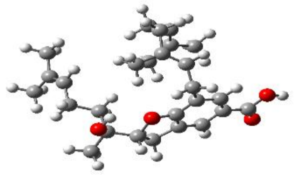	-1352.11191	1.44201798	1.139%
1a-20	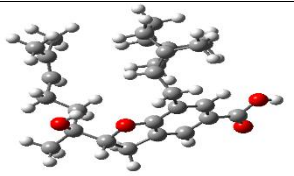	-1352.111805	1.50790653	1.019%

Table S1.5. Cartesian coordinates of low-energy conformers of **1a**

	1a-1			1a-2			1a-3			1a-4		
**Element**	**X**	**Y**	**Z**	**X**	**Y**	**Z**	**X**	**Y**	**Z**	**X**	**Y**	**Z**
C	0.940683	4.339394	0.024905	-1.606688	3.137121	0.416319	0.946834	4.119669	-0.340823	1.019981	4.097051	-0.632868
C	0.397265	3.877541	1.355887	-0.566142	2.729250	1.432321	2.268748	3.619582	0.189218	1.915331	3.815567	0.549362
C	-1.015253	4.332395	1.672479	0.849302	3.194173	1.142733	3.458737	3.834341	-0.726857	3.401670	3.974439	0.290567
C	-2.118593	3.674202	0.798408	1.547164	2.392874	0.012537	3.419332	3.059790	-2.066358	4.013510	2.977962	-0.723533
C	-2.202373	2.182493	0.978879	2.925821	2.911567	-0.298114	3.456932	1.561955	-1.892390	3.953851	1.543018	-0.262351
C	-3.263821	1.440775	1.333119	4.112721	2.333553	-0.051000	2.875307	0.617953	-2.650870	3.813392	0.432212	-1.004730
C	-3.141805	-0.058689	1.476892	5.401466	3.029090	-0.423249	3.081709	-0.850272	-2.354720	3.842193	-0.937057	-0.364137
C	-4.646126	1.978232	1.615299	4.298671	0.985854	0.603813	2.000389	0.896123	-3.850498	3.638786	0.416379	-2.505143
C	1.072309	3.121256	2.235694	-0.816179	2.003602	2.533556	2.443718	3.051771	1.392922	1.480612	3.481944	1.774777
C	2.464108	2.542728	2.111311	-2.132172	1.432228	3.013151	1.418813	2.781442	2.472841	0.058716	3.311938	2.263645
C	2.471462	1.032199	2.256891	-2.131076	-0.084969	3.033148	1.299073	1.303949	2.794194	-0.210161	1.907071	2.767946
C	2.893010	0.394920	3.428201	-1.835541	-0.825351	4.179335	2.024287	0.694310	3.822985	-0.081586	1.548884	4.111877
C	2.871957	-1.002399	3.568909	-1.825321	-2.229858	4.185410	1.925473	-0.680436	4.093026	-0.305342	0.238751	4.565013
C	3.312774	-1.674189	4.811165	-1.503586	-2.910907	5.458710	2.685932	-1.323117	5.187124	-0.148501	-0.025324	6.012175
O	3.736238	-0.807714	5.772758	-1.526674	-4.269578	5.359763	3.477795	-0.455749	5.876905	-0.392708	-1.322719	6.349360
O	3.313136	-2.877176	4.995909	-1.239043	-2.355505	6.508590	2.638467	-2.504827	5.474897	0.164994	0.799354	6.850385
C	2.427820	-1.812943	2.508269	-2.122472	-2.944033	3.009874	1.081367	-1.495525	3.315906	-0.668691	-0.772480	3.655543
C	2.004352	-1.205316	1.340139	-2.424641	-2.231049	1.861797	0.348014	-0.912570	2.298768	-0.811093	-0.438467	2.319561
C	2.026032	0.188474	1.234031	-2.417100	-0.833579	1.886030	0.466369	0.459029	2.055421	-0.582298	0.875130	1.901055
O	1.581294	0.636847	0.022601	-2.738212	-0.276660	0.684558	-0.327057	0.888533	1.030468	-0.773656	1.048063	0.560100
C	1.004054	-0.506003	-0.701101	-3.174730	-1.345996	-0.219746	-1.231208	-0.209858	0.668629	-1.392225	-0.172928	0.031014
C	1.384353	-0.352641	-2.188075	-2.636076	-1.028496	-1.630495	-1.403351	-0.181194	-0.863316	-0.795353	-0.425127	-1.368114
O	2.812793	-0.363737	-2.295881	-3.143204	0.265349	-1.988311	-0.118398	-0.363963	-1.469959	0.620841	-0.596045	-1.235648
C	0.839034	0.970414	-2.749691	-3.255537	-2.006545	-2.632409	-1.995694	1.163820	-1.313057	-1.083517	0.763918	-2.298286
C	0.922806	-1.564513	-3.020749	-1.090328	-1.020114	-1.681574	-2.240765	-1.374479	-1.363701	-1.291249	-1.756371	-1.966076
C	-0.595341	-1.833812	-3.072067	-0.506959	-0.622226	-3.053842	-3.690982	-1.470854	-0.846448	-2.810156	-1.889288	-2.200908
C	-0.919686	-2.978036	-3.997315	0.972451	-0.353399	-2.982416	-4.428523	-2.617893	-1.487509	-3.149993	-3.190542	-2.880473
C	-1.502006	-4.152713	-3.711538	1.978411	-0.994459	-3.597633	-4.963461	-3.703482	-0.907884	-3.911599	-4.200414	-2.432449
C	-1.733160	-5.180781	-4.794387	3.409408	-0.554356	-3.391444	-5.662636	-4.753908	-1.738943	-4.124439	-5.436381	-3.275061
C	-1.980014	-4.574805	-2.343288	1.815461	-2.170269	-4.530190	-4.940377	-3.997931	0.572498	-4.620771	-4.228381	-1.100122
C	1.544229	-1.765836	0.015176	-2.762840	-2.678597	0.457703	-0.592976	-1.486135	1.265650	-1.132771	-1.264308	1.096154
H	0.924788	5.436459	-0.036512	-1.332193	2.792840	-0.590370	0.689532	3.624876	-1.286441	1.253529	3.428299	-1.471615
H	1.965631	4.010628	-0.158953	-2.601388	2.745811	0.641165	0.117407	3.955897	0.350013	-0.041323	3.978280	-0.405760
H	0.324011	3.972519	-0.805858	-1.680908	4.231702	0.355583	0.999534	5.195715	-0.558609	1.175560	5.121054	-1.000737
H	-1.240204	4.129711	2.726120	0.839981	4.258689	0.864430	3.539147	4.906760	-0.961710	3.592137	4.992295	-0.082734
H	-1.083480	5.422124	1.534910	1.460660	3.114260	2.048939	4.379434	3.564410	-0.194230	3.946150	3.885940	1.239178
H	-3.072229	4.158478	1.032573	1.568059	1.336128	0.298293	2.548533	3.371652	-2.653578	3.539167	3.102295	-1.703312
H	-1.916627	3.903830	-0.258783	0.933849	2.455120	-0.897781	4.298109	3.379538	-2.648663	5.066251	3.269531	-0.865934
H	-1.260482	1.659358	0.809102	2.940909	3.898891	-0.764456	4.078082	1.220267	-1.062073	4.087801	1.409376	0.812897
H	-2.121788	-0.405658	1.281593	5.220906	3.999615	-0.896089	3.727115	-1.002992	-1.483491	3.977801	-0.875964	0.720677
H	-3.419823	-0.384036	2.489424	6.031950	3.193805	0.462079	2.122571	-1.349324	-2.167174	2.911986	-1.482753	-0.566985
H	-3.819794	-0.581933	0.787200	5.995998	2.418065	-1.117036	3.543121	-1.361159	-3.211909	4.662146	-1.543742	-0.774423
H	-4.716945	3.064610	1.525728	3.357951	0.500982	0.873957	1.862480	1.962722	-4.046225	3.619095	1.415661	-2.947843
H	-5.381092	1.538037	0.926548	4.849843	0.304336	-0.058877	2.434268	0.446709	-4.754751	4.457711	-0.140884	-2.981326
H	-4.969707	1.702131	2.628651	4.900842	1.082521	1.517994	1.012634	0.438912	-3.710983	2.707060	-0.098737	-2.771125
H	0.568505	2.860331	3.167263	0.027175	1.774926	3.187035	3.453341	2.734326	1.657735	2.232476	3.306980	2.545607
H	2.921268	2.821397	1.156817	-2.321290	1.788290	4.034880	1.722242	3.309010	3.387944	-0.116640	4.013308	3.091122
H	3.103795	2.962707	2.900121	-2.964044	1.785502	2.397062	0.436718	3.172604	2.193196	-0.661972	3.560282	1.479601
H	3.245245	1.000045	4.257473	-1.613383	-0.312996	5.110948	2.676889	1.304197	4.439670	0.190838	2.300156	4.847524
H	3.995165	-1.368717	6.526633	-1.300999	-4.594938	6.250445	3.917084	-0.997141	6.558074	-0.255561	-1.366361	7.313392
H	2.431805	-2.891103	2.632214	-2.117310	-4.028613	3.017325	1.024497	-2.556407	3.537441	-0.834547	-1.785152	4.006670
H	-0.082221	-0.422148	-0.576967	-4.267035	-1.262778	-0.266614	-2.187597	0.009318	1.159166	-2.465293	0.040011	-0.050103
H	3.132417	0.355238	-1.724391	-2.877787	0.871975	-1.276263	0.467426	0.337210	-1.132501	0.973916	0.203115	-0.805003
H	1.126283	1.068216	-3.801496	-3.027850	-1.690069	-3.653457	-2.102911	1.171710	-2.402521	-0.646509	0.573342	-3.283780
H	-0.252453	1.029618	-2.678044	-2.870416	-3.021940	-2.489450	-2.978015	1.350190	-0.865377	-2.158056	0.937724	-2.421807
H	1.252591	1.819739	-2.196830	-4.345441	-2.024356	-2.526278	-1.337577	1.988181	-1.022635	-0.644042	1.680934	-1.894643
H	1.300330	-1.405963	-4.038883	-0.732985	-0.315969	-0.918647	-2.247545	-1.314557	-2.459464	-0.762035	-1.888071	-2.918389
H	1.435703	-2.458188	-2.647410	-0.695027	-2.003887	-1.398912	-1.707783	-2.299940	-1.117637	-0.953073	-2.574473	-1.319918
H	-0.981513	-2.025104	-2.065060	-0.729943	-1.398056	-3.792917	-3.699410	-1.563550	0.244952	-3.350905	-1.791613	-1.253339
H	-1.112759	-0.933181	-3.432584	-1.026949	0.285052	-3.389802	-4.223244	-0.537370	-1.079286	-3.153831	-1.060970	-2.837048
H	-0.619983	-2.809539	-5.033708	1.243453	0.479365	-2.331154	-4.516493	-2.538021	-2.572943	-2.693742	-3.309433	-3.865348
H	-1.374269	-4.836058	-5.769204	3.478929	0.304566	-2.716213	-5.658280	-4.502637	-2.804184	-3.595573	-5.375713	-4.231354
H	-1.223045	-6.125432	-4.558529	4.018545	-1.369246	-2.974178	-5.184303	-5.736234	-1.618958	-3.776885	-6.337606	-2.750470
H	-2.801412	-5.421357	-4.890749	3.879250	-0.275876	-4.345694	-6.708109	-4.877822	-1.422863	-5.191802	-5.593016	-3.485961
H	-1.795185	-3.828440	-1.567068	0.776855	-2.486465	-4.653011	-4.421040	-3.237624	1.160732	-4.446885	-3.335816	-0.494391
H	-3.059169	-4.782022	-2.356730	2.213311	-1.933596	-5.527066	-5.962642	-4.084007	0.966442	-5.705917	-4.329929	-1.240816
H	-1.490415	-5.508800	-2.034394	2.389155	-3.034502	-4.166844	-4.452732	-4.963334	0.766479	-4.303061	-5.100495	-0.511935
H	2.389210	-2.187898	-0.542163	-1.898296	-3.142392	-0.032729	-0.037233	-2.032034	0.493676	-0.274548	-1.882979	0.806973
H	0.776514	-2.540105	0.109103	-3.580732	-3.405475	0.424780	-1.347070	-2.159860	1.684049	-1.999191	-1.920721	1.223819

	1a-5			1a-6			1a-7			1a-8		
**Element**	**X**	**Y**	**Z**	**X**	**Y**	**Z**	**X**	**Y**	**Z**	**X**	**Y**	**Z**
C	-0.507908	3.801099	3.518163	2.658882	4.751206	1.172952	1.442036	3.739293	3.463402	2.039908	3.751925	3.068216
C	-0.308590	3.267873	2.119030	1.262218	4.295255	1.524311	1.021259	3.740520	2.013799	1.623646	3.676553	1.619242
C	0.988230	3.683699	1.449194	0.561437	5.127144	2.582219	2.047787	4.266616	1.030480	2.723127	3.920214	0.604766
C	2.258691	2.984560	2.008825	1.141651	5.015076	4.017017	3.375665	3.465559	0.980228	3.900818	2.911670	0.660342
C	2.271022	1.501102	1.757156	0.863058	3.687766	4.668112	3.202572	2.040025	0.526264	3.495656	1.493769	0.354221
C	3.198828	0.767357	1.122151	1.724555	2.800143	5.187787	3.599903	0.910986	1.135493	3.708281	0.384570	1.081074
C	3.013210	-0.722418	0.947427	1.225674	1.521697	5.819808	3.343855	-0.441916	0.512534	3.234119	-0.966563	0.597844
C	4.481303	1.306508	0.535794	3.226130	2.955276	5.203546	4.332615	0.852063	2.454584	4.429410	0.345048	2.407302
C	-1.179448	2.480672	1.467809	0.626557	3.264619	0.943862	-0.172214	3.311092	1.575722	0.373992	3.412214	1.207940
C	-2.500691	1.931308	1.958419	1.109753	2.370689	-0.172360	-1.305853	2.726709	2.395732	-0.841516	3.115605	2.064555
C	-2.472535	0.421513	2.117719	1.175734	0.896676	0.196784	-1.246298	1.211890	2.498618	-1.039477	1.631932	2.326894
C	-2.309888	-0.201600	3.356779	1.868833	0.432007	1.321308	-1.111609	0.539615	3.714216	-1.026246	1.082602	3.611726
C	-2.279828	-1.598437	3.497696	1.953006	-0.934622	1.630136	-1.028103	-0.861090	3.793746	-1.182858	-0.296137	3.836683
C	-2.112906	-2.149552	4.860693	2.702761	-1.437594	2.800884	-0.893083	-1.466541	5.136542	-1.174972	-0.875242	5.197688
O	-2.096282	-3.511573	4.888757	3.321184	-0.454765	3.519930	-0.823967	-2.827141	5.106440	-1.004412	0.053543	6.179531
O	-1.998248	-1.492307	5.878243	2.795381	-2.605905	3.127417	-0.844078	-0.852725	6.186342	-1.303914	-2.057162	5.458719
C	-2.411900	-2.426812	2.367662	1.323505	-1.889754	0.807439	-1.063552	-1.640701	2.623043	-1.344374	-1.177107	2.752049
C	-2.575463	-1.831506	1.128225	0.622424	-1.452589	-0.299329	-1.204281	-0.995694	1.405055	-1.367873	-0.654873	1.470820
C	-2.598212	-0.438119	1.021615	0.562822	-0.084100	-0.586571	-1.304305	0.395671	1.364129	-1.230088	0.721633	1.282938
O	-2.766338	-0.002500	-0.260223	-0.154682	0.186431	-1.718801	-1.447841	0.885513	0.098031	-1.283133	1.090992	-0.030082
C	-3.057001	-1.163759	-1.108602	-0.845774	-1.045002	-2.123187	-1.589932	-0.244755	-0.827516	-1.611167	-0.091739	-0.835802
C	-2.336969	-0.961909	-2.458242	-0.838877	-1.090303	-3.664966	-0.715222	0.078961	-2.059024	-0.689273	-0.052487	-2.074740
O	-2.819638	0.272643	-3.006900	0.521709	-1.102160	-4.112482	0.636011	0.256917	-1.614145	0.670788	-0.057101	-1.621172
C	-2.791233	-2.051255	-3.433790	-1.556275	0.141226	-4.241555	-1.201279	1.371445	-2.735589	-0.946886	1.223613	-2.893153
C	-0.798905	-0.906999	-2.299681	-1.434869	-2.407206	-4.198780	-0.652762	-1.097997	-3.050846	-0.822814	-1.322162	-2.936669
C	-0.042713	-0.577809	-3.602939	-2.909137	-2.695040	-3.847253	-1.989565	-1.545814	-3.677563	-2.213883	-1.604042	-3.541560
C	1.420446	-0.316695	-3.362707	-3.395360	-3.959289	-4.507392	-1.782310	-2.637966	-4.694740	-2.190217	-2.816889	-4.435357
C	2.482168	-0.977817	-3.851068	-3.849292	-5.085928	-3.937090	-2.254447	-3.894024	-4.689437	-2.865445	-3.969507	-4.308206
C	3.888308	-0.543399	-3.508457	-4.286425	-6.256424	-4.786670	-1.920885	-4.846444	-5.813958	-2.693579	-5.077840	-5.320614
C	2.411575	-2.172265	-4.771821	-3.979103	-5.315683	-2.450771	-3.137295	-4.485345	-3.617176	-3.836274	-4.289255	-3.197340
C	-2.716533	-2.415447	-0.259114	-0.101275	-2.191541	-1.400218	-1.197051	-1.505969	-0.017183	-1.441391	-1.307426	0.110108
H	-0.477059	4.899461	3.518989	3.316704	4.724073	2.051227	1.904811	4.697194	3.736316	2.659358	4.640246	3.250990
H	-1.454565	3.494412	3.968480	3.129378	4.145092	0.395475	0.609160	3.561243	4.148172	1.189254	3.788604	3.753451
H	0.295780	3.467920	4.187797	2.648859	5.793443	0.824551	2.191029	2.960006	3.655428	2.645416	2.880457	3.349456
H	0.929767	3.484129	0.372691	0.606387	6.185442	2.283058	1.609818	4.291155	0.024390	2.295029	3.910727	-0.405786
H	1.120805	4.769641	1.565690	-0.502099	4.858980	2.614104	2.296743	5.307641	1.288261	3.140369	4.927593	0.757633
H	3.136175	3.470358	1.569573	2.212197	5.243579	4.006964	4.042096	3.988970	0.277539	4.643777	3.242237	-0.081705
H	2.319793	3.173710	3.091210	0.671026	5.807884	4.618509	3.870640	3.515433	1.955196	4.398205	2.982325	1.632846
H	1.394573	0.974809	2.136115	-0.198761	3.436434	4.721068	2.698746	1.933858	-0.437669	2.976476	1.371084	-0.599502
H	2.094782	-1.076721	1.425979	0.133672	1.450822	5.791193	2.772956	-0.371496	-0.417527	2.676539	-0.901676	-0.340628
H	2.966590	-0.992253	-0.117565	1.639254	0.645516	5.303437	4.289489	-0.961553	0.300300	4.082670	-1.650257	0.450330
H	3.857497	-1.282091	1.374660	1.542818	1.449372	6.870234	2.785613	-1.088794	1.204553	2.583444	-1.440722	1.346582
H	4.582407	2.390067	0.631638	3.571288	3.888562	4.751931	4.519822	1.833614	2.896268	4.771414	1.324537	2.749857
H	5.352407	0.845915	1.023068	3.609534	2.921478	6.233225	3.767751	0.255107	3.183944	3.781437	-0.079295	3.186699
H	4.557660	1.053932	-0.531061	3.698848	2.123005	4.664669	5.303617	0.351683	2.332651	5.308741	-0.311601	2.346887
H	-0.913844	2.163205	0.459074	-0.382616	3.036674	1.289212	-0.359798	3.346827	0.503808	0.193674	3.369133	0.135119
H	-2.789144	2.380733	2.912398	0.429293	2.474246	-1.026811	-1.317541	3.141129	3.408910	-0.782746	3.631886	3.028317
H	-3.284537	2.192588	1.234068	2.094905	2.690942	-0.530862	-2.257226	3.015655	1.928841	-1.730483	3.509448	1.553441
H	-2.211165	0.400255	4.255230	2.341092	1.155035	1.977859	-1.069527	1.102396	4.641950	-0.892238	1.740026	4.464577
H	-1.981686	-3.746921	5.827689	3.773725	-0.919691	4.247590	-0.736438	-3.099086	6.038342	-1.018115	-0.451040	7.013371
H	-2.388190	-3.505432	2.478821	1.407685	-2.941200	1.062414	-0.979326	-2.720168	2.685816	-1.444371	-2.239947	2.947360
H	-4.133978	-1.118501	-1.308866	-1.873069	-0.953499	-1.749874	-2.648133	-0.274570	-1.111547	-2.657865	0.027129	-1.138453
H	-2.672977	0.952393	-2.327448	0.938931	-0.312267	-3.727936	0.608120	0.911978	-0.894687	0.750489	0.664850	-0.973180
H	-2.433554	-1.823991	-4.441270	-1.526531	0.103854	-5.335151	-0.555213	1.602942	-3.588320	-0.267454	1.251090	-3.750916
H	-2.406947	-3.034560	-3.142155	-2.602715	0.195869	-3.922211	-2.233466	1.286416	-3.092694	-1.977084	1.275333	-3.262171
H	-3.884723	-2.097214	-3.473552	-1.062342	1.060984	-3.912523	-1.161603	2.210138	-2.033860	-0.768743	2.113801	-2.282190
H	-0.561625	-0.145891	-1.544631	-1.317324	-2.384242	-5.289608	0.042640	-0.801933	-3.846495	-0.084675	-1.233183	-3.743854
H	-0.426059	-1.859085	-1.901544	-0.811168	-3.237345	-3.848064	-0.185849	-1.952771	-2.548799	-0.508628	-2.184595	-2.338221
H	-0.180953	-1.385521	-4.328107	-3.037012	-2.748799	-2.760664	-2.685733	-1.868207	-2.895608	-2.956861	-1.721121	-2.745135
H	-0.505946	0.315007	-4.043932	-3.534827	-1.857760	-4.188653	-2.463644	-0.687922	-4.175667	-2.535190	-0.736575	-4.135810
H	1.622348	0.530132	-2.703069	-3.352287	-3.935258	-5.598204	-1.149510	-2.346987	-5.535664	-1.516199	-2.725702	-5.289555
H	3.896349	0.330695	-2.849365	-4.177755	-6.049143	-5.855836	-1.281015	-4.379619	-6.569273	-1.983829	-4.806158	-6.108221
H	4.445270	-1.350707	-3.011983	-3.699494	-7.155922	-4.552998	-1.404551	-5.740033	-5.435664	-2.333828	-5.999244	-4.841274
H	4.455631	-0.288660	-4.415066	-5.337708	-6.514188	-4.595182	-2.832290	-5.203742	-6.313930	-3.651077	-5.330429	-5.797783
H	2.946590	-3.027682	-4.336108	-3.647651	-4.467213	-1.847436	-3.360380	-3.790924	-2.803661	-3.941300	-3.485459	-2.464772
H	1.390622	-2.493176	-4.990778	-5.022956	-5.528991	-2.181453	-4.093881	-4.818774	-4.043096	-4.834513	-4.502341	-3.604474
H	2.906287	-1.954385	-5.728811	-3.395481	-6.194537	-2.143443	-2.667557	-5.376657	-3.178518	-3.522753	-5.194676	-2.659430
H	-1.786626	-2.896750	-0.585710	0.611892	-2.671029	-2.081682	-0.193656	-1.846861	-0.297666	-0.509198	-1.840279	-0.110407
H	-3.510309	-3.165977	-0.329963	-0.791903	-2.957505	-1.034220	-1.895909	-2.330569	-0.190757	-2.269093	-2.016558	0.008590

	1a-9			1a-10			1a-11			1a-12		
**Element**	**X**	**Y**	**Z**	**X**	**Y**	**Z**	**X**	**Y**	**Z**	**X**	**Y**	**Z**

C	-1.245628	2.958253	0.070247	2.844115	2.962439	2.592859	-0.733511	3.478737	1.039843	1.928949	3.517184	3.406607
C	-0.232734	2.695531	1.159706	2.151514	2.578128	1.308088	0.644548	3.012251	1.447259	1.453916	3.487943	1.974191
C	1.174780	3.181452	0.864031	2.979164	2.738864	0.042591	1.777836	3.485469	0.554720	2.496118	3.828812	0.927784
C	1.984854	2.279096	-0.109373	3.800182	1.481548	-0.356544	1.867736	2.779117	-0.825806	3.720840	2.877034	0.889550
C	2.317704	0.931599	0.471361	4.911397	1.122430	0.592927	2.249182	1.327283	-0.716969	3.368575	1.455338	0.538864
C	3.526296	0.388287	0.688368	5.101912	-0.021863	1.270231	3.273476	0.683857	-1.301805	3.663565	0.328255	1.207064
C	3.652383	-0.988453	1.298855	6.308632	-0.195706	2.163295	3.504481	-0.787589	-1.046206	3.230166	-1.022479	0.685854
C	4.846833	1.046022	0.367136	4.187207	-1.221569	1.220224	4.275432	1.315227	-2.238460	4.443553	0.267785	2.498745
C	-0.505282	2.088824	2.325776	0.894854	2.114378	1.219418	0.904384	2.252606	2.523484	0.200895	3.183503	1.602775
C	-1.820936	1.521210	2.813407	-0.081683	1.822166	2.336115	-0.063375	1.715906	3.555486	-0.961586	2.791609	2.494044
C	-1.784678	0.007065	2.913831	-0.446684	0.348824	2.400107	-0.179860	0.202567	3.538618	-1.078427	1.290274	2.698034
C	-1.399910	-0.663599	4.076652	0.384480	-0.594810	3.012902	0.593887	-0.623288	4.356526	-0.987468	0.688448	3.955929
C	-1.338520	-2.064654	4.151388	0.058934	-1.959094	3.058154	0.471106	-2.022241	4.343762	-1.069797	-0.704538	4.125710
C	-0.924300	-2.668665	5.436863	0.935165	-2.956061	3.709799	1.339271	-2.798349	5.256399	-0.979719	-1.339528	5.458564
O	-0.899305	-4.030598	5.404823	2.075907	-2.418364	4.230493	1.137748	-4.143151	5.172021	-0.814525	-0.445760	6.473268
O	-0.627407	-2.052098	6.442982	0.703199	-4.147486	3.794218	2.161683	-2.326415	6.018973	-1.041467	-2.536342	5.671841
C	-1.669791	-2.847866	3.029978	-1.134530	-2.421539	2.470966	-0.463236	-2.642334	3.493495	-1.233276	-1.545805	3.010289
C	-2.062499	-2.205126	1.868093	-1.970079	-1.507603	1.857001	-1.245836	-1.843497	2.676795	-1.333081	-0.971693	1.755191
C	-2.111833	-0.808618	1.825081	-1.617757	-0.153563	1.827136	-1.085936	-0.455187	2.699143	-1.268184	0.416626	1.623079
O	-2.527654	-0.326404	0.619708	-2.536917	0.623306	1.186030	-1.923063	0.192576	1.840830	-1.391856	0.837986	0.330513
C	-2.978304	-1.456880	-0.198382	-3.690837	-0.208910	0.827649	-2.866999	-0.782855	1.283780	-1.699946	-0.324452	-0.512196
C	-2.566752	-1.186826	-1.660355	-4.112457	0.163413	-0.610502	-3.073996	-0.445858	-0.209301	-0.837498	-0.186016	-1.786481
O	-3.201306	0.039625	-2.053138	-4.412601	1.566185	-0.606272	-3.497014	0.922977	-0.271743	0.539461	-0.137055	-1.390405
C	-3.173472	-2.268896	-2.557421	-5.430344	-0.538562	-0.947222	-4.249312	-1.267002	-0.746909	-1.196863	1.107281	-2.536926
C	-1.034858	-1.058436	-1.823436	-2.995075	-0.135283	-1.637325	-1.782960	-0.645338	-1.037713	-0.943361	-1.422327	-2.699119
C	-0.583172	-0.699178	-3.253343	-3.309272	0.330966	-3.073745	-1.880027	-0.155306	-2.496863	-2.341748	-1.744103	-3.265902
C	0.885496	-0.370383	-3.316086	-2.100074	0.244515	-3.966369	-0.554560	-0.248455	-3.205040	-2.328648	-3.010221	-4.082905
C	1.481435	0.687071	-3.890389	-1.917706	-0.497489	-5.069785	-0.252556	-0.901318	-4.338403	-2.603030	-3.170744	-5.386622
C	2.984513	0.837387	-3.856888	-0.612947	-0.441715	-5.829761	1.154136	-0.871834	-4.890137	-2.523245	-4.536743	-6.027290
C	0.760593	1.793075	-4.623251	-2.943692	-1.431032	-5.665514	-1.228063	-1.699603	-5.169264	-3.008531	-2.064845	-6.330653
C	-2.451746	-2.733221	0.505827	-3.280608	-1.676194	1.123253	-2.316481	-2.182726	1.664334	-1.430273	-1.568833	0.370632
H	-0.906434	2.543861	-0.888894	3.126579	4.024336	2.571010	-0.958352	3.184776	0.005236	2.513294	4.425515	3.605965
H	-2.227917	2.533267	0.288614	2.231240	2.801246	3.483291	-1.526078	3.081641	1.677798	1.108306	3.480445	4.127517
H	-1.371487	4.038349	-0.088513	3.773217	2.392360	2.712638	-0.796258	4.575466	1.065687	2.586556	2.664473	3.620217
H	1.119950	4.188899	0.425855	2.316755	2.980069	-0.797911	1.658351	4.564127	0.374353	2.025779	3.842496	-0.063750
H	1.739693	3.272891	1.799013	3.665153	3.590687	0.152752	2.735299	3.355700	1.072524	2.872086	4.847649	1.109167
H	1.407910	2.137163	-1.035377	3.107099	0.645540	-0.493000	0.896335	2.868182	-1.335454	4.414400	3.273912	0.132361
H	2.892057	2.820642	-0.397310	4.239979	1.684682	-1.345587	2.581972	3.330542	-1.445791	4.256390	2.930864	1.842661
H	1.448522	0.340601	0.761224	5.660111	1.907694	0.722885	1.609840	0.743492	-0.053696	2.814093	1.347809	-0.396609
H	2.674896	-1.428645	1.519381	6.945677	0.694621	2.170701	2.759955	-1.205123	-0.361444	2.630169	-0.944763	-0.225092
H	4.191664	-1.673011	0.628254	6.007474	-0.408330	3.198879	3.467217	-1.363611	-1.981596	4.101318	-1.659214	0.473963
H	4.228550	-0.955870	2.234594	6.921392	-1.048687	1.838409	4.500153	-0.961557	-0.613958	2.634347	-1.556948	1.439671
H	4.742728	2.047024	-0.057737	3.310538	-1.071484	0.586693	4.108989	2.382744	-2.399463	4.755414	1.247279	2.868964
H	5.467813	1.127543	1.270314	4.727539	-2.102323	0.844676	5.297011	1.187376	-1.854020	3.850639	-0.218850	3.285532
H	5.420240	0.437774	-0.346691	3.827998	-1.481919	2.225104	4.252272	0.822029	-3.220504	5.348350	-0.343385	2.372745
H	0.319387	1.957539	3.027452	0.511406	1.883878	0.223505	1.944151	1.977980	2.705680	-0.023583	3.180317	0.537444
H	-2.034745	1.928742	3.810530	0.325740	2.130482	3.303496	0.287186	2.018668	4.551459	-0.886001	3.268756	3.476641
H	-2.648096	1.822277	2.164674	-0.999240	2.403556	2.180376	-1.057555	2.153080	3.427053	-1.889083	3.163046	2.037555
H	-1.143530	-0.097060	4.967209	1.309131	-0.259069	3.470709	1.312369	-0.184108	5.042576	-0.850373	1.315027	4.831221
H	-0.614970	-4.302323	6.296688	2.555866	-3.170498	4.623290	1.759823	-4.535867	5.811476	-0.770705	-0.985189	7.283932
H	-1.620424	-3.929731	3.089195	-1.366940	-3.480733	2.515499	-0.563591	-3.722297	3.492579	-1.275217	-2.619542	3.162265
H	-4.074007	-1.428167	-0.163841	-4.501106	0.110024	1.492857	-3.818452	-0.591661	1.793454	-2.763872	-0.244909	-0.762735
H	-2.908580	0.716907	-1.420066	-3.625627	2.014301	-0.252441	-2.824019	1.441399	0.201223	0.608559	0.557518	-0.711885
H	-3.042700	-2.000175	-3.608663	-5.828904	-0.152949	-1.889154	-4.514297	-0.929479	-1.752111	-0.559885	1.205271	-3.421663
H	-2.700119	-3.241614	-2.386314	-5.293982	-1.620803	-1.045195	-4.003477	-2.333448	-0.792687	-2.244401	1.118487	-2.857803
H	-4.248610	-2.362117	-2.370239	-6.175988	-0.345923	-0.168510	-5.130556	-1.134796	-0.110338	-1.036849	1.980212	-1.896780
H	-0.685208	-0.282723	-1.131039	-2.079811	0.364017	-1.292764	-0.967470	-0.108602	-0.534744	-0.244355	-1.267496	-3.529604
H	-0.544010	-1.990036	-1.511682	-2.770523	-1.209263	-1.651691	-1.498841	-1.705093	-1.038993	-0.564122	-2.290856	-2.147294
H	-0.786972	-1.552140	-3.918259	-4.141164	-0.250949	-3.482315	-2.650274	-0.720589	-3.030536	-3.053597	-1.872311	-2.435809
H	-1.198760	0.128290	-3.616367	-3.654818	1.372132	-3.022335	-2.222167	0.888405	-2.481191	-2.710989	-0.900323	-3.856399
H	1.534710	-1.101840	-2.830240	-1.265405	0.873582	-3.648668	0.258751	0.285269	-2.708986	-2.042606	-3.901475	-3.520602
H	3.464594	0.019052	-3.311551	0.101407	0.245981	-5.366156	1.826207	-0.283695	-4.257237	-2.228383	-5.308880	-5.309817
H	3.402956	0.863419	-4.873256	-0.143172	-1.434123	-5.884735	1.567423	-1.887042	-4.977667	-3.490184	-4.827404	-6.461836
H	3.276681	1.781613	-3.376366	-0.772113	-0.116979	-6.867928	1.175575	-0.441783	-5.901860	-1.797757	-4.544508	-6.852983
H	-0.326269	1.684967	-4.610429	-2.543970	-2.452602	-5.732683	-2.234537	-1.733184	-4.745704	-3.048770	-1.080775	-5.858058
H	1.009330	2.772300	-4.190948	-3.875257	-1.473339	-5.096425	-1.306906	-1.281788	-6.182901	-2.308116	-2.002758	-7.175042
H	1.079398	1.830973	-5.674503	-3.192656	-1.129329	-6.692620	-0.878453	-2.734743	-5.289339	-3.997434	-2.269150	-6.764286
H	-1.588253	-3.166216	-0.014550	-3.153874	-2.262802	0.205605	-1.898181	-2.712119	0.800166	-0.481966	-2.042102	0.090501
H	-3.224787	-3.507020	0.547786	-4.041859	-2.183303	1.725024	-3.107539	-2.816518	2.078106	-2.224963	-2.314836	0.269696

		**1a-13**			**1a-14**			**1a-15**			**1a-16**	
**Element**	**X**	**Y**	**Z**	**X**	**Y**	**Z**	**X**	**Y**	**Z**	**X**	**Y**	**Z**
C	1.857742	3.630537	-0.165997	0.413699	4.081648	0.292509	2.193659	4.490130	1.086258	2.337636	4.476281	1.058551
C	2.170289	3.454210	1.299991	0.943701	3.856045	1.687580	0.926246	3.871709	1.627678	1.021311	3.841764	1.441512

C	3.647128	3.412697	1.661178	2.371650	4.317369	1.935334	0.364904	4.539499	2.868855	0.291838	4.516477	2.588167
C	4.291381	2.001261	1.649884	3.467507	3.254449	1.658560	1.200093	4.365612	4.164947	0.956884	4.374267	3.983051
C	4.383999	1.356313	0.291940	3.617080	2.857924	0.213244	1.122303	2.979605	4.745842	0.861631	2.982611	4.546529
C	4.098662	0.090343	-0.057792	3.759900	1.628189	-0.309993	2.116196	2.132788	5.056074	1.842469	2.164584	4.957476
C	4.291880	-0.382003	-1.480852	3.933925	1.435850	-1.799394	1.817430	0.787878	5.676542	1.522392	0.796895	5.514005
C	3.594947	-0.975562	0.887157	3.786949	0.346128	0.489066	3.586053	2.398889	4.838514	3.316560	2.487482	4.915103
C	1.249969	3.344043	2.270570	0.246952	3.298344	2.689897	0.282645	2.836380	1.064483	0.472438	2.786173	0.818891
C	-0.258948	3.341842	2.149625	-1.166281	2.756136	2.674141	0.622489	2.102355	-0.208756	0.988499	2.029470	-0.380907
C	-0.857874	1.996647	2.516958	-1.199877	1.251154	2.865861	0.985816	0.637286	-0.020665	1.235583	0.550838	-0.124078
C	-1.205860	1.662180	3.830058	-1.243664	0.653685	4.128116	1.812302	0.181465	1.010207	2.038833	0.093074	0.927324
C	-1.723541	0.401411	4.167774	-1.234848	-0.739416	4.301289	2.170138	-1.170709	1.138048	2.289654	-1.273037	1.130452
C	-2.099375	0.051761	5.555346	-1.292711	-1.261799	5.684365	3.069514	-1.551295	2.246639	3.155593	-1.765457	2.222380
O	-1.884698	1.061234	6.444035	-1.283935	-2.622988	5.743337	3.320229	-2.888462	2.300384	3.700286	-0.769669	2.982685
O	-2.556734	-1.019075	5.909686	-1.343176	-0.583634	6.693514	3.568014	-0.786657	3.055232	3.396562	-2.934915	2.456388
C	-1.903062	-0.579173	3.174198	-1.174573	-1.591406	3.182173	1.689069	-2.124428	0.218878	1.722030	-2.235277	0.271647
C	-1.575800	-0.267146	1.867586	-1.141621	-1.021842	1.920949	0.858817	-1.697449	-0.801380	0.914797	-1.806915	-0.763977
C	-1.066257	0.998545	1.561294	-1.155354	0.369003	1.782092	0.528902	-0.342686	-0.907691	0.689066	-0.437730	-0.946516
O	-0.808419	1.158764	0.228562	-1.134721	0.772555	0.476241	-0.283651	-0.078307	-1.973275	-0.115915	-0.173791	-2.018235
C	-1.389557	0.013015	-0.482415	-1.352840	-0.411297	-0.363574	-0.752805	-1.362928	-2.519281	-0.688372	-1.448162	-2.484793
C	-0.442709	-0.337908	-1.648042	-0.490780	-0.245632	-1.631593	-0.798719	-1.210136	-4.053137	-0.742068	-1.379255	-4.024488
O	0.848403	-0.646477	-1.106516	0.881232	-0.122522	-1.234393	0.528202	-0.920555	-4.514994	0.596324	-1.211276	-4.512565
C	-0.319121	0.848196	-2.617512	-0.911889	1.011529	-2.408844	-1.736780	-0.057787	-4.448279	-1.601098	-0.187053	-4.477162
C	-0.880434	-1.627150	-2.369573	-0.528261	-1.507487	-2.515170	-1.167592	-2.522709	-4.777215	-1.211186	-2.700506	-4.670634
C	-2.274042	-1.612281	-3.031228	-1.905990	-1.924371	-3.070294	-2.618907	-3.040850	-4.670852	-2.692827	-3.110553	-4.519147
C	-2.538058	-2.889643	-3.785701	-1.790592	-3.107404	-3.996400	-2.994351	-3.631754	-3.335980	-3.084833	-3.608835	-3.151661
C	-3.493158	-3.808663	-3.576348	-2.329713	-4.329857	-3.871547	-4.068725	-3.378068	-2.570431	-4.122714	-3.240873	-2.382168
C	-3.582700	-5.038147	-4.449980	-2.083644	-5.390180	-4.919420	-4.283119	-4.119918	-1.271457	-4.365777	-3.903444	-1.045917
C	-4.548514	-3.734768	-2.499539	-3.209146	-4.778661	-2.729624	-5.153457	-2.382042	-2.903258	-5.137721	-2.183695	-2.745069
C	-1.596016	-1.077964	0.593356	-1.027452	-1.623071	0.539841	0.224363	-2.425241	-1.963108	0.211909	-2.548582	-1.876192
H	2.355491	2.853767	-0.759913	1.077659	3.620110	-0.449152	2.038711	5.553864	0.857755	3.040600	4.468744	1.901541
H	0.788824	3.589008	-0.386324	-0.590002	3.677823	0.142806	3.001167	4.446139	1.828267	2.830133	3.978730	0.220041
H	2.243192	4.594056	-0.528307	0.386810	5.155691	0.060287	2.559794	4.003163	0.179557	2.193139	5.530606	0.784111
H	4.211321	4.063054	0.977262	2.581777	5.207365	1.324764	0.270951	5.618214	2.670529	0.204693	5.590826	2.365159
H	3.784691	3.828545	2.666691	2.473769	4.629443	2.981747	-0.651042	4.169543	3.056448	-0.732794	4.128559	2.649258
H	5.309644	2.112795	2.056329	4.421609	3.682775	2.005987	2.236265	4.671140	3.987440	1.993695	4.723138	3.940932
H	3.748112	1.364763	2.355993	3.274898	2.382544	2.292228	0.800069	5.078179	4.902947	0.434673	5.065772	4.661995
H	4.774708	2.011109	-0.490734	3.651253	3.699877	-0.482407	0.102958	2.645007	4.952968	-0.161666	2.609319	4.629878
H	4.691031	0.409926	-2.123438	3.943944	2.390490	-2.335997	0.741449	0.625142	5.799544	0.444862	0.604668	5.531137
H	4.984761	-1.234364	-1.522731	4.874146	0.911669	-2.022144	2.226909	-0.021588	5.059836	2.000799	0.011568	4.914200
H	3.339091	-0.726440	-1.903266	3.122225	0.817384	-2.203805	2.288489	0.699996	6.666553	1.902765	0.687986	6.540004
H	3.398960	-0.603724	1.895673	3.595804	0.495185	1.554436	3.788375	3.370740	4.380745	3.532246	3.482406	4.517943
H	2.672728	-1.428376	0.501606	3.046345	-0.365299	0.102255	4.131795	2.359344	5.792087	3.756565	2.427447	5.920701
H	4.330525	-1.788392	0.969578	4.767640	-0.141172	0.392150	4.015612	1.618539	4.197703	3.847865	1.752637	4.295270
H	1.610288	3.223361	3.294046	0.747312	3.195806	3.654697	-0.620290	2.476198	1.559127	-0.484785	2.422295	1.194399
H	-0.669764	4.101394	2.828862	-1.733471	3.222008	3.491401	-0.241478	2.148845	-0.884063	0.254589	2.112552	-1.192275
H	-0.574826	3.616064	1.139730	-1.678855	3.015178	1.744051	1.440507	2.602925	-0.741183	1.911252	2.482718	-0.761346
H	-1.081014	2.404159	4.612436	-1.296005	1.274546	5.017878	2.181336	0.886072	1.748783	2.466825	0.819011	1.610459
H	-2.168513	0.707190	7.306698	-1.322358	-2.838005	6.693276	3.923384	-3.005822	3.057060	4.242237	-1.228921	3.650343
H	-2.297423	-1.549723	3.457502	-1.159723	-2.667312	3.318115	1.972410	-3.166481	0.318834	1.935229	-3.285277	0.444115
H	-2.356118	0.355131	-0.872750	-2.417411	-0.405034	-0.628802	-1.760857	-1.519787	-2.122920	-1.700319	-1.504176	-2.071565
H	1.143000	0.137808	-0.611253	0.937951	0.650117	-0.645294	0.820652	-0.138510	-4.016198	0.950773	-0.428183	-4.057549
H	0.365619	0.587678	-3.430915	-0.282759	1.121393	-3.297938	-1.766305	0.037844	-5.538574	-1.634715	-0.149733	-5.570884
H	-1.285838	1.125900	-3.051434	-1.959205	0.965887	-2.726630	-2.757078	-0.213824	-4.081780	-2.626205	-0.250608	-4.096530
H	0.076934	1.727258	-2.100011	-0.792413	1.904582	-1.787912	-1.376759	0.886208	-4.027942	-1.171770	0.751035	-4.112282
H	-0.117585	-1.835032	-3.130548	0.161560	-1.326777	-3.349260	-0.941994	-2.340785	-5.834793	-0.989666	-2.592180	-5.739129
H	-0.827969	-2.458506	-1.657480	-0.100395	-2.341694	-1.947647	-0.472387	-3.308145	-4.455438	-0.566532	-3.513416	-4.313977
H	-3.049950	-1.436803	-2.278264	-2.597540	-2.138659	-2.248313	-3.319966	-2.257247	-4.973440	-3.343463	-2.295696	-4.850535
H	-2.333475	-0.769879	-3.735297	-2.343089	-1.083467	-3.627686	-2.717356	-3.832382	-5.429707	-2.858508	-3.929270	-5.236263
H	-1.836532	-3.076659	-4.601265	-1.169560	-2.924007	-4.875564	-2.299986	-4.395059	-2.976995	-2.441278	-4.403716	-2.767529
H	-2.802006	-5.052940	-5.216907	-1.443842	-5.024897	-5.728789	-3.478695	-4.834220	-1.069343	-3.612202	-4.665216	-0.822473
H	-3.489962	-5.955782	-3.852077	-1.604404	-6.276595	-4.480358	-4.343145	-3.424650	-0.422408	-4.358264	-3.166849	-0.230273
H	-4.557222	-5.094844	-4.955289	-3.028322	-5.736162	-5.362277	-5.232492	-4.673552	-1.285468	-5.353297	-4.385194	-1.018396
H	-4.465965	-2.847362	-1.867463	-3.368566	-4.007396	-1.972272	-5.269716	-1.649389	-2.092756	-4.933071	-1.693008	-3.699404
H	-5.554384	-3.736780	-2.942048	-4.195373	-5.091531	-3.099860	-4.970980	-1.832882	-3.829827	-6.144244	-2.620786	-2.804610
H	-4.496729	-4.617454	-1.847160	-2.774939	-5.655575	-2.229656	-6.123698	-2.888518	-3.002192	-5.184362	-1.407680	-1.968615
H	-0.765999	-1.794778	0.575621	-0.006055	-1.976319	0.352338	0.975938	-2.686890	-2.718157	0.932497	-2.909678	-2.620306
H	-2.527494	-1.630926	0.438150	-1.714319	-2.456744	0.363900	-0.301820	-3.338985	-1.671821	-0.378077	-3.401319	-1.528195

		1a-17			1a-18			1a-19			1a-20	
**Element**	**X**	**Y**	**Z**	**X**	**Y**	**Z**	**X**	**Y**	**Z**	**X**	**Y**	**Z**
C	0.779188	3.572968	-1.145383	1.394478	3.467365	-0.720200	-0.302622	3.277476	0.161807	-0.115667	3.465543	3.118102
C	1.805920	3.462942	-0.044347	1.876819	3.320868	0.702600	0.965159	3.099061	0.962880	-0.074489	2.979021	1.689217
C	3.238248	3.737572	-0.461944	3.369201	3.503227	0.904859	2.229010	3.583271	0.276777	1.134232	3.426212	0.890636
C	3.844288	2.731038	-1.469692	4.269476	2.443591	0.224615	2.856167	2.545908	-0.699141	2.443356	2.669208	1.251402

C	3.963882	1.332488	-0.918001	4.068040	1.051298	0.768130	3.575319	1.435081	0.016931	2.403458	1.215946	0.862283
C	3.842347	0.163873	-1.569186	4.147417	-0.120256	0.115723	3.271400	0.129667	0.079825	2.510988	0.128246	1.641225
C	4.058225	-1.149269	-0.851337	3.971433	-1.429881	0.850619	4.121191	-0.821103	0.889975	2.426720	-1.256590	1.042611
C	3.509239	0.025745	-3.036120	4.428287	-0.266463	-1.361304	2.094671	-0.519457	-0.605476	2.712158	0.146371	3.137250
C	1.524430	3.180553	1.237398	1.082329	3.081516	1.757763	1.023924	2.547751	2.184186	-0.997033	2.187811	1.121184
C	0.179414	2.915163	1.877854	-0.420274	2.906742	1.794931	-0.111411	1.993015	3.019315	-2.217491	1.558305	1.754169
C	0.099766	1.529960	2.490478	-0.826308	1.541368	2.316171	-0.109342	0.474373	3.082970	-2.084596	0.046947	1.827593
C	0.408079	1.277682	3.829141	-1.102658	1.301731	3.665963	0.805662	-0.239421	3.864758	-1.546724	-0.602443	2.942195
C	0.359540	-0.012643	4.381076	-1.450043	0.026837	4.142078	0.816499	-1.642634	3.910701	-1.392990	-1.997566	2.991646
C	0.698317	-0.162252	5.812997	-1.750625	-0.224520	5.568185	1.779233	-2.396186	4.744662	-0.806495	-2.681515	4.164422
O	0.615804	-1.449932	6.251566	-1.645896	0.888147	6.347254	2.642318	-1.596109	5.429254	-0.369192	-1.820919	5.127572
O	1.022999	0.741766	6.560424	-2.063195	-1.301877	6.040858	1.828567	-3.608848	4.835357	-0.694661	-3.886228	4.293095
C	-0.005345	-1.111367	3.580013	-1.525318	-1.063187	3.254789	-0.114438	-2.381974	3.156960	-1.794292	-2.792980	1.901590
C	-0.324204	-0.883830	2.252227	-1.264755	-0.847804	1.913994	-1.033662	-1.698410	2.382961	-2.323005	-2.171045	0.785299
C	-0.266818	0.412668	1.733396	-0.922545	0.432997	1.469804	-1.017015	-0.298858	2.352989	-2.447626	-0.778951	0.760296
O	-0.618769	0.475685	0.416614	-0.706661	0.489947	0.123797	-1.991311	0.226052	1.554101	-2.963556	-0.306805	-0.410050
C	-1.174526	-0.831388	0.032057	-1.139503	-0.790162	-0.459860	-2.875913	-0.863384	1.124969	-3.195961	-1.435182	-1.320077
C	-0.697814	-1.121959	-1.404899	-0.142036	-1.136340	-1.583086	-3.289667	-0.599616	-0.337816	-2.419929	-1.153520	-2.629552
O	0.736639	-1.161662	-1.403762	1.164763	-1.257866	-1.002712	-3.952914	0.672598	-0.363081	-2.868064	0.121051	-3.109620
C	-1.175863	-0.018888	-2.362308	-0.125191	-0.027033	-2.646653	-4.350859	-1.622996	-0.750547	-2.845244	-2.167475	-3.694837
C	-1.108043	-2.525625	-1.901038	-0.401201	-2.523860	-2.209422	-2.073144	-0.581161	-1.292073	-0.889356	-1.128534	-2.402521
C	-2.596169	-2.788989	-2.221414	-1.646904	-2.708166	-3.104331	-2.413515	-0.206979	-2.749756	-0.069929	-0.694746	-3.635404
C	-3.500957	-2.911914	-1.022207	-2.963880	-2.755558	-2.372760	-1.175906	0.071529	-3.560695	1.407152	-0.647131	-3.344752
C	-4.673054	-2.301566	-0.780610	-4.094452	-2.073602	-2.620714	-0.689549	-0.601245	-4.615485	2.240741	0.404722	-3.398422
C	-5.453391	-2.600158	0.478567	-5.332702	-2.302670	-1.785344	0.583378	-0.155101	-5.296187	3.709159	0.244040	-3.079006
C	-5.335062	-1.302824	-1.699200	-4.267873	-1.055386	-3.721958	-1.325981	-1.825084	-5.228780	1.835842	1.808475	-3.778982
C	-0.704440	-1.817343	1.127398	-1.213824	-1.779701	0.726651	-2.132772	-2.180419	1.464085	-2.834140	-2.720852	-0.526153
H	0.991862	2.861626	-1.954151	1.664597	4.454641	-1.120709	-0.181855	2.871022	-0.851692	-0.092337	4.563595	3.151050
H	-0.239832	3.382788	-0.802598	1.870987	2.726425	-1.375368	-1.167029	2.784832	0.612674	-1.003433	3.136189	3.662990
H	0.803508	4.573998	-1.598660	0.314169	3.346329	-0.821823	-0.540675	4.343521	0.039238	0.761768	3.119680	3.680464
H	3.292645	4.739538	-0.914551	3.663902	4.491322	0.519388	2.005534	4.499266	-0.288040	0.939370	3.291310	-0.180989
H	3.876533	3.765996	0.430227	3.590649	3.511692	1.979637	2.983024	3.854591	1.026069	1.305319	4.501338	1.046953
H	3.270294	2.744122	-2.402766	4.126505	2.472581	-0.861153	2.083054	2.159766	-1.372336	3.266190	3.163747	0.713121
H	4.846676	3.103985	-1.733729	5.313424	2.752025	0.393855	3.576512	3.080896	-1.335434	2.662244	2.795164	2.316805
H	4.223203	1.287225	0.141597	3.873097	1.010916	1.841649	4.454739	1.766349	0.574169	2.244307	1.045055	-0.204873
H	4.300994	-1.000028	0.205876	3.774586	-1.275365	1.916570	4.971193	-0.313371	1.357053	2.277047	-1.223012	-0.041271
H	3.163244	-1.781192	-0.915602	3.141174	-2.007834	0.425134	3.528894	-1.294599	1.685505	3.340711	-1.833374	1.244779
H	4.880360	-1.716317	-1.311068	4.871810	-2.054171	0.759280	4.512558	-1.636575	0.265355	1.595908	-1.824688	1.483822
H	3.359283	0.985658	-3.537123	4.554586	0.690758	-1.873872	1.510332	0.171519	-1.217216	2.779511	1.154433	3.553792
H	4.315518	-0.501609	-3.565076	5.344683	-0.851624	-1.521821	2.428772	-1.340653	-1.254912	1.887330	-0.373372	3.644515
H	2.600135	-0.576001	-3.161037	3.611566	-0.814791	-1.847545	1.418066	-0.964293	0.137206	3.632001	-0.389823	3.410700
H	2.359856	3.131641	1.937260	1.557330	2.998149	2.736387	2.006386	2.462809	2.649070	-0.845978	1.909740	0.077593
H	0.016326	3.653523	2.675171	-0.848100	3.670202	2.459757	-0.014202	2.382583	4.042056	-2.389547	1.949092	2.761038
H	-0.633968	3.040838	1.157938	-0.861859	3.063900	0.807054	-1.080401	2.334237	2.647808	-3.106332	1.807113	1.159699
H	0.687903	2.098587	4.483115	-1.057332	2.128934	4.367334	1.525945	0.308473	4.463916	-1.236242	-0.009571	3.796208
H	0.861549	-1.414586	7.194111	-1.865343	0.589795	7.248845	3.213411	-2.205451	5.931948	-0.008980	-2.388222	5.833797
H	-0.036519	-2.108046	4.006653	-1.790089	-2.041016	3.644135	-0.091667	-3.465778	3.208763	-1.674518	-3.869914	1.963458
H	-2.263792	-0.724819	0.052565	-2.135393	-0.619293	-0.880864	-3.780407	-0.766467	1.737180	-4.261771	-1.396153	-1.563736
H	1.050270	-0.316629	-1.034774	1.351541	-0.426001	-0.531898	-3.333291	1.315595	0.020695	-2.758177	0.743011	-2.370067
H	-0.843854	-0.243124	-3.381389	0.576449	-0.289923	-3.445161	-4.779750	-1.346353	-1.717136	-2.436467	-1.883125	-4.667765
H	-2.266901	0.077785	-2.363253	-1.114720	0.131406	-3.088730	-3.925343	-2.628641	-0.834182	-2.493792	-3.176342	-3.453869
H	-0.758745	0.948045	-2.066701	0.193972	0.920304	-2.202749	-5.166635	-1.644524	-0.020098	-3.936305	-2.187216	-3.787560
H	-0.524441	-2.689534	-2.814866	0.490215	-2.738971	-2.810815	-1.346997	0.140604	-0.896473	-0.670920	-0.436178	-1.579899
H	-0.749432	-3.271476	-1.180772	-0.409008	-3.274392	-1.409287	-1.569423	-1.555903	-1.282920	-0.546288	-2.117449	-2.070653
H	-2.966009	-2.039039	-2.927002	-1.655102	-1.952569	-3.895595	-3.013473	-0.998929	-3.208709	-0.240196	-1.410962	-4.452291
H	-2.626598	-3.745242	-2.766104	-1.512695	-3.671787	-3.619525	-3.046627	0.689915	-2.729412	-0.450269	0.267442	-3.987547
H	-3.152561	-3.628206	-0.274486	-2.990677	-3.476720	-1.552585	-0.606531	0.940362	-3.221940	1.840260	-1.608162	-3.057898
H	-4.942966	-3.330683	1.114121	-5.167197	-3.049400	-1.002364	1.011332	0.733820	-4.822022	3.962734	-0.791680	-2.831781
H	-5.616363	-1.688382	1.070155	-5.664733	-1.372013	-1.304184	1.342943	-0.949460	-5.276916	4.334335	0.554192	-3.928290
H	-6.450454	-2.996355	0.240046	-6.171003	-2.644795	-2.408410	0.405591	0.077409	-6.355840	4.001954	0.875776	-2.228719
H	-5.527914	-0.358626	-1.171441	-4.599647	-0.091948	-3.310894	-0.623253	-2.670030	-5.222514	0.770563	1.907594	-3.998691
H	-4.747498	-1.075624	-2.591713	-5.050365	-1.376760	-4.423443	-2.236567	-2.144090	-4.716420	2.081615	2.514241	-2.972990
H	-6.314050	-1.675561	-2.031304	-3.357608	-0.879269	-4.299759	-1.580406	-1.643514	-6.282442	2.393744	2.145698	-4.663783
H	0.166475	-2.388479	0.782834	-0.314741	-2.407149	0.761790	-1.721066	-2.665251	0.570520	-2.083027	-3.327390	-1.043445
H	-1.494752	-2.525049	1.394046	-2.086025	-2.434883	0.647295	-2.805169	-2.900441	1.941037	-3.715722	-3.356994	-0.387137

Table S1.6. Calculated conformational analysis of the **1b** at B3LYP/6-31g(d) level

conformer	3D conformer	G (Hartree)	ΔG (kcal/mol)	Population
1b-1	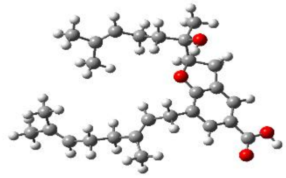	-1352.115836	0	13.966%
1b-2	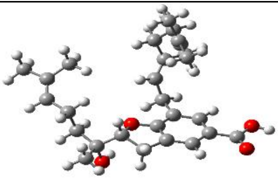	-1352.115793	0.02698293	13.344%
1b-3	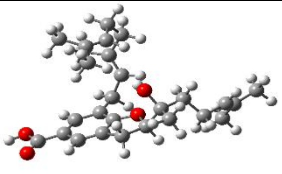	-1352.115167	0.41980419	6.876%
1b-4	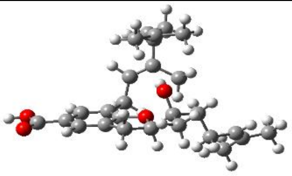	-1352.115118	0.45055218	6.528%
1b-5	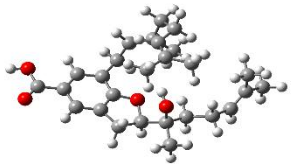	-1352.115105	0.45870981	6.439%
1b-6	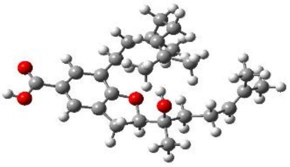	-1352.115032	0.50451804	5.960%
1b-7	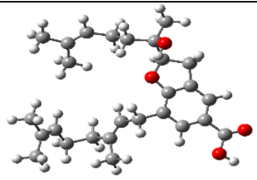	-1352.115004	0.52208832	5.786%
1b-8	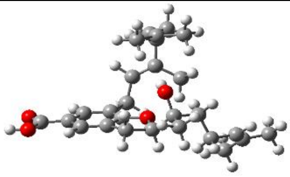	-1352.114981	0.53652105	5.646%

1b-9	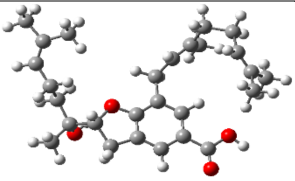	-1352.114738	0.68900598	4.365%
1b-10	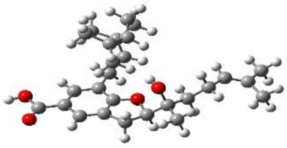	-1352.114593	0.77999493	3.744%
1b-11	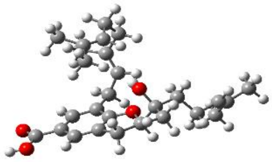	-1352.114491	0.84400095	3.360%
1b-12	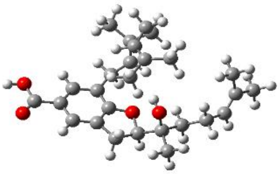	-1352.11442	0.88855416	3.117%
1b-13	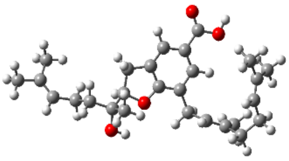	-1352.114219	1.01468367	2.519%
1b-14	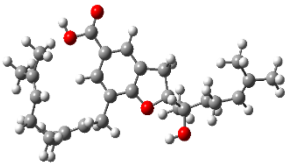	-1352.114108	1.08433728	2.240%
1b-15	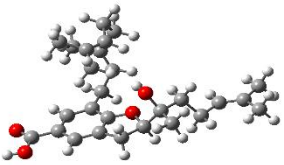	-1352.114031	1.13265555	2.064%
1b-16	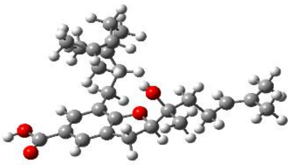	-1352.11392	1.20230916	1.835%
1b-17	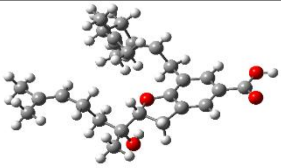	-1352.113847	1.24811739	1.699%

1b-18	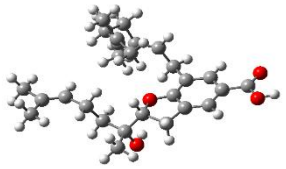	-1352.113818	1.26631518	1.647%
1b-19	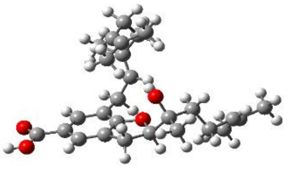	-1352.113531	1.44641055	1.215%

Table S1.7. Cartesian coordinates of low-energy conformers of **1b**

	**1b-1**			**1b-2**			**1b-3**			**1b-4**		
**Element**	**X**	**Y**	**Z**	**X**	**Y**	**Z**	**X**	**Y**	**Z**	**X**	**Y**	**Z**
C	2.056201	2.031484	3.426114	3.796799	2.061856	1.607991	3.294399	1.931497	1.941760	1.173886	2.517863	-0.555135
C	1.058517	1.647085	2.358592	2.463395	1.916678	0.912723	2.239926	2.015679	0.865083	1.802910	2.258227	0.792237
C	-0.144326	2.562645	2.230944	1.617071	3.175854	0.867298	2.740535	2.370479	-0.521099	3.319068	2.205090	0.800341
C	0.196098	3.994805	1.744845	1.050946	3.634553	2.239145	3.774476	1.382433	-1.121609	3.946573	1.003787	0.052898
C	-1.025144	4.870605	1.665219	0.089952	2.647398	2.844664	3.234216	-0.007790	-1.331582	3.624504	-0.328349	0.685124
C	-1.538770	5.506469	0.600695	-1.173613	2.850745	3.250118	3.743011	-1.177582	-0.910995	3.521553	-1.528916	0.090963
C	-2.792546	6.339699	0.730712	-1.984714	1.718534	3.836690	3.063882	-2.486996	-1.239903	3.261481	-2.777230	0.903491
C	-0.953380	5.477570	-0.790418	-1.906676	4.169028	3.185162	5.011755	-1.328795	-0.105976	3.670885	-1.767400	-1.393181
C	1.171933	0.577289	1.555848	2.013278	0.785001	0.348174	0.931335	1.804734	1.074587	1.120998	2.114316	1.939494
C	2.280831	-0.451154	1.514903	2.682988	-0.570007	0.288162	0.248028	1.429552	2.375325	-0.374105	2.163562	2.166947
C	1.805119	-1.838332	1.908830	1.883811	-1.637340	1.014834	0.108382	-0.071161	2.568980	-0.906477	0.870484	2.754176
C	2.033459	-2.378925	3.176376	2.228851	-2.100558	2.288466	0.677782	-0.750308	3.649309	-1.077592	0.681050	4.129191
C	1.579450	-3.656299	3.541183	1.469062	-3.072647	2.959469	0.564169	-2.143301	3.800022	-1.542357	-0.531750	4.663730
C	1.877314	-4.126664	4.912132	1.832612	-3.560319	4.308274	1.162088	-2.859966	4.947398	-1.731516	-0.728661	6.117541
O	1.386568	-5.371242	5.169420	2.961965	-2.983656	4.805141	1.814146	-2.039083	5.817393	-1.405028	0.366827	6.858311
O	2.493160	-3.500953	5.754797	1.212250	-4.392171	4.944225	1.101785	-4.061345	5.133825	-2.135056	-1.750837	6.640827
C	0.873061	-4.448224	2.616204	0.323559	-3.620858	2.352519	-0.124877	-2.905797	2.839631	-1.847623	-1.609567	3.811766
C	0.630555	-3.931338	1.354908	-0.039461	-3.174613	1.094817	-0.702959	-2.253976	1.764526	-1.693982	-1.442180	2.447702
C	1.087807	-2.652302	1.027456	0.733288	-2.200456	0.456703	-0.590934	-0.866694	1.656187	-1.231975	-0.222012	1.945395
O	0.771033	-2.283387	-0.250539	0.251014	-1.859868	-0.775997	-1.219218	-0.362443	0.554478	-1.144432	-0.205320	0.583087
C	-0.149574	-3.287078	-0.798675	-1.070291	-2.480378	-0.933992	-1.924983	-1.453648	-0.125022	-1.784379	-1.422007	0.072746
C	0.226203	-3.508188	-2.277207	-1.191253	-2.958489	-2.394011	-1.631314	-1.306615	-1.632904	-0.957767	-1.919352	-1.128387
O	1.560356	-4.026408	-2.337916	-0.202722	-3.967892	-2.631818	-0.216702	-1.433699	-1.830604	0.355544	-2.266930	-0.673733
C	-0.663941	-4.589119	-2.895173	-2.541161	-3.644917	-2.615476	-2.269277	-2.451497	-2.422310	-1.557075	-3.215242	-1.680857
C	0.178260	-2.188880	-3.085830	-0.958048	-1.803548	-3.398305	-2.070610	0.076825	-2.172950	-0.823173	-0.834748	-2.224410
C	-1.188074	-1.480327	-3.198185	-1.925326	-0.602631	-3.331499	-3.568362	0.435035	-2.067645	-2.127604	-0.301392	-2.854365
C	-1.119644	-0.285686	-4.113837	-1.670245	0.375950	-4.448601	-3.875556	1.738298	-2.759102	-1.845717	0.632468	-4.002972
C	-1.342813	1.006219	-3.827604	-1.254798	1.649632	-4.370947	-4.299158	2.893522	-2.223422	-2.105981	1.945355	-4.100815
C	-1.216291	2.064867	-4.898212	-1.046462	2.469098	-5.623151	-4.543121	4.102907	-3.095621	-1.732672	2.713787	-5.347001
C	-1.734331	1.538760	-2.470615	-0.960415	2.389344	-3.088378	-4.574585	3.119319	-0.756499	-2.773782	2.778396	-3.033595
C	-0.020889	-4.521784	0.126464	-1.146959	-3.581971	0.151315	-1.427235	-2.763522	0.540415	-1.878911	-2.384350	1.281605
H	1.550279	2.160027	4.392714	3.668502	2.341513	2.661906	3.970467	2.795878	1.895115	1.480913	1.760640	-1.287913
H	2.846781	1.290557	3.563887	4.398442	1.150493	1.583459	2.871362	1.889545	2.948609	0.082453	2.520305	-0.528022
H	2.540290	2.990271	3.198472	4.387280	2.864057	1.144069	3.916358	1.036024	1.813664	1.505635	3.486721	-0.954724
H	-0.872325	2.123821	1.538491	2.224081	4.000135	0.462875	1.887152	2.452595	-1.206331	3.711800	3.126968	0.345292
H	-0.648395	2.640442	3.207023	0.778258	3.030656	0.176246	3.212194	3.365048	-0.490321	3.676790	2.195000	1.837745
H	0.911847	4.448714	2.445751	1.890553	3.798754	2.931530	4.088978	1.796159	-2.092136	3.653130	1.025255	-1.002715
H	0.710641	3.927425	0.780540	0.581154	4.614106	2.102500	4.673688	1.369230	-0.497629	5.037831	1.154229	0.058785
H	-1.555825	4.982039	2.613490	0.490100	1.638560	2.951429	2.318285	-0.049083	-1.926289	3.511642	-0.298623	1.770504
H	-3.182940	6.334416	1.753301	-1.419179	0.781939	3.864115	2.127583	-2.346091	-1.786933	3.165871	-2.555175	1.971392
H	-2.606537	7.384438	0.443410	-2.307436	1.950892	4.861708	3.722920	-3.129496	-1.841634	2.342950	-3.276806	0.570414
H	-3.584325	5.971705	0.062582	-2.902606	1.545936	3.256383	2.838993	-3.050057	-0.322802	4.079312	-3.501466	0.779939
H	-0.052499	4.864507	-0.868884	-1.318813	4.972648	2.735635	5.497675	-0.379036	0.129485	3.857974	-0.851724	-1.960119
H	-1.687631	5.092221	-1.511815	-2.835834	4.067678	2.606853	4.809114	-1.849547	0.840139	4.504982	-2.455526	-1.589732
H	-0.696850	6.492858	-1.124361	-2.204655	4.496406	4.191297	5.739191	-1.948715	-0.648748	2.765845	-2.242676	-1.792252
H	0.373131	0.394339	0.836742	1.033178	0.812213	-0.127831	0.263852	1.880056	0.217937	1.697103	1.938517	2.849156
H	2.680962	-0.492466	0.492066	2.788042	-0.864447	-0.765727	0.784042	1.840097	3.237331	-0.599362	2.978585	2.869026
H	3.112785	-0.165983	2.164400	3.692176	-0.537104	0.707720	-0.751572	1.885132	2.384425	-0.907633	2.388873	1.239361
H	2.578208	-1.803672	3.919018	3.109485	-1.694743	2.775671	1.225096	-0.185939	4.397300	-0.853347	1.500234	4.805057
H	1.646803	-5.563865	6.088799	3.088370	-3.384940	5.684401	2.155731	-2.627095	6.515699	-1.571860	0.108686	7.783299
H	0.534812	-5.439903	2.896237	-0.242434	-4.378450	2.884925	-0.183217	-3.982204	2.965063	-2.199013	-2.540149	4.245757
H	-1.155278	-2.856367	-0.723692	-1.806204	-1.692925	-0.732004	-2.992553	-1.301262	0.069475	-2.788007	-1.128752	-0.258638
H	2.128768	-3.362794	-1.910992	0.658823	-3.550302	-2.461632	0.206463	-0.774506	-1.252800	0.755611	-1.466892	-0.287819
H	-0.415683	-4.708331	-3.954556	-2.627540	-3.955534	-3.661451	-2.066910	-2.318533	-3.489777	-0.977823	-3.546562	-2.548475
H	-1.725459	-4.334448	-2.814608	-3.377302	-2.978333	-2.381469	-3.353818	-2.485858	-2.277680	-2.598165	-3.079666	-1.990656
H	-0.499160	-5.551666	-2.402410	-2.625433	-4.539991	-1.992249	-1.848470	-3.414525	-2.119555	-1.519285	-4.009384	-0.929504
H	0.897724	-1.488067	-2.644998	0.064365	-1.430647	-3.261820	-1.492956	0.849222	-1.651621	-0.272777	0.013674	-1.801871
H	0.549845	-2.427853	-4.090325	-0.996326	-2.250140	-4.399936	-1.758365	0.112435	-3.224290	-0.185837	-1.261498	-3.009325
H	-1.931154	-2.185576	-3.598531	-2.961115	-0.963683	-3.412034	-4.168069	-0.358819	-2.536343	-2.725044	-1.145450	-3.228731
H	-1.542178	-1.189122	-2.204094	-1.843077	-0.111092	-2.356889	-3.870875	0.471662	-1.016162	-2.735107	0.193611	-2.090114
H	-0.839380	-0.527539	-5.141192	-1.838887	-0.033321	-5.446841	-3.709135	1.720573	-3.838147	-1.349084	0.158227	-4.851988
H	-0.935414	1.637156	-5.865796	-1.272025	1.897287	-6.528807	-4.334776	3.895922	-4.150036	-1.253295	2.073701	-6.094351
H	-2.161474	2.610669	-5.029387	-1.682109	3.365959	-5.620076	-5.584640	4.445340	-3.015667	-2.618127	3.172261	-5.809776
H	-0.460785	2.815286	-4.625515	-0.009029	2.825289	-5.694975	-3.914424	4.949076	-2.784106	-1.044221	3.538107	-5.112767
H	-1.014592	2.295893	-2.130337	0.075903	2.754630	-3.083163	-4.370633	2.244275	-0.135203	-3.021455	2.213770	-2.131772
H	-2.711009	2.040741	-2.518183	-1.601805	3.277117	-2.996861	-3.965389	3.949931	-0.373887	-2.126359	3.615484	-2.737265
H	-1.794219	0.766876	-1.699802	-1.102672	1.780799	-2.192235	-5.623357	3.407424	-0.598376	-3.701925	3.226732	-3.414965
H	0.628264	-5.276332	-0.333954	-0.942655	-4.565218	-0.289251	-0.733887	-3.293037	-0.123036	-1.069771	-3.123647	1.245319
H	-0.993857	-4.981664	0.324856	-2.133015	-3.616303	0.624356	-2.257122	-3.437977	0.773597	-2.832545	-2.920554	1.299328

	1b-5			1b-6			1b-7			1b-8		
**Element**	**X**	**Y**	**Z**	**X**	**Y**	**Z**	**X**	**Y**	**Z**	**X**	**Y**	**Z**
C	0.269836	2.833799	0.167542	0.268251	2.864340	0.198170	3.395649	1.487772	2.204809	0.946394	2.609404	-0.685212
C	1.212944	2.367605	1.249885	1.198437	2.391442	1.288778	1.924812	1.285085	1.929205	2.059367	2.198843	0.248222
C	2.688535	2.459389	0.909929	2.677971	2.485997	0.967193	0.993601	2.240704	2.650224	3.444803	2.167303	-0.369194
C	3.174333	1.455296	-0.163009	3.177279	1.487069	-0.104358	1.203600	3.739753	2.317690	3.670500	1.073743	-1.440987
C	3.034348	0.014118	0.269206	3.033625	0.043869	0.319980	0.973744	4.058556	0.864542	3.585003	-0.329271	-0.891175
C	2.959865	-1.078294	-0.508894	2.973432	-1.045486	-0.463626	1.732827	4.784897	0.029789	3.206985	-1.451021	-1.526614
C	2.873280	-2.461047	0.094747	2.880812	-2.430924	0.132995	1.315669	4.996042	-1.406961	3.254613	-2.791688	-0.829064
C	2.973511	-1.051118	-2.018555	3.010226	-1.011982	-1.972780	3.030108	5.460167	0.405039	2.732993	-1.507084	-2.959815
C	0.831150	1.913958	2.454279	0.802452	1.930165	2.485664	1.420861	0.336895	1.124572	1.898449	1.908429	1.548867
C	-0.567953	1.732935	3.000499	-0.603074	1.745476	3.013839	2.164902	-0.713157	0.329639	0.627971	1.908957	2.370687
C	-0.857250	0.283403	3.341629	-0.895394	0.293590	3.341685	1.806992	-2.125590	0.756725	0.333468	0.546525	2.968197
C	-0.696845	-0.234852	4.630700	-0.752062	-0.234627	4.626782	2.619024	-2.880352	1.609404	0.727949	0.186527	4.258781
C	-0.937457	-1.586408	4.927628	-0.994078	-1.587616	4.914625	2.271863	-4.179179	2.014598	0.478563	-1.088080	4.792676
C	-0.776372	-2.131635	6.293345	-0.819199	-2.035731	6.313648	3.130315	-4.975883	2.918661	0.933657	-1.356916	6.174346
O	-0.371453	-1.201496	7.202253	-1.083712	-3.360856	6.488512	4.268251	-4.328148	3.293665	0.639536	-2.617644	6.599333
O	-0.974242	-3.288741	6.614943	-0.475776	-1.329641	7.243415	2.881916	-6.100768	3.311452	1.514360	-0.564949	6.893123
C	-1.348521	-2.471577	3.913584	-1.391365	-2.468965	3.891351	1.078330	-4.768817	1.557673	-0.185762	-2.060585	4.021616
C	-1.521610	-1.979320	2.632964	-1.548181	-1.965713	2.611171	0.257154	-4.039560	0.717342	-0.594005	-1.723295	2.742425
C	-1.276073	-0.628656	2.368781	-1.300793	-0.613842	2.357926	0.626359	-2.745456	0.339596	-0.332553	-0.445188	2.241288
O	-1.503025	-0.292407	1.064912	-1.510739	-0.270402	1.052995	-0.283873	-2.151708	-0.490132	-0.805916	-0.262431	0.973752
C	-2.174103	-1.424304	0.417342	-2.171209	-1.398903	0.389921	-1.476433	-3.006671	-0.544459	-1.641099	-1.417261	0.630694
C	-1.655440	-1.518197	-1.033239	-1.632158	-1.484924	-1.053738	-1.993925	-2.995976	-1.996474	-1.392101	-1.754101	-0.851890
O	-0.243206	-1.748285	-1.009704	-0.220627	-1.716524	-1.011681	-0.987458	-3.560061	-2.845614	-0.021136	-2.136335	-1.015214
C	-2.271213	-2.739418	-1.722251	-2.239067	-2.701812	-1.758157	-3.210007	-3.915358	-2.132899	-2.211447	-2.981659	-1.258740
C	-1.983472	-0.201988	-1.780639	-1.948707	-0.164370	-1.798311	-2.297558	-1.560112	-2.489381	-1.674083	-0.543051	-1.773535
C	-1.477010	-0.131673	-3.234775	-1.423082	-0.086992	-3.245257	-3.396817	-0.774854	-1.743214	-3.101119	0.045117	-1.750316
C	-1.742295	1.216224	-3.852484	-1.678505	1.264569	-3.859119	-3.688813	0.541975	-2.414839	-3.275432	1.115175	-2.796969
C	-0.866191	2.090702	-4.371523	-0.794864	2.139120	-4.365080	-3.553643	1.784352	-1.926046	-3.502381	2.426847	-2.626689
C	-1.337895	3.406466	-4.945494	-1.257298	3.458778	-4.937645	-3.898115	2.986921	-2.773426	-3.638899	3.345882	-3.818137
C	0.625991	1.876431	-4.452404	0.697480	1.921239	-4.431952	-3.068978	2.121762	-0.537094	-3.645607	3.115415	-1.290949
C	-1.899929	-2.643162	1.329971	-1.908415	-2.622691	1.299454	-1.030315	-4.383397	0.006181	-1.270977	-2.520152	1.652106
H	0.433304	2.283397	-0.767994	0.444914	2.322057	-0.739735	3.581520	1.540062	3.286327	0.903764	1.952741	-1.563638
H	-0.782371	2.716653	0.433908	-0.787017	2.742850	0.450135	4.023251	0.690782	1.799541	-0.039133	2.588983	-0.215843
H	0.444442	3.894215	-0.063297	0.443371	3.927001	-0.021751	3.751595	2.434854	1.778487	1.119352	3.625512	-1.067382
H	2.916956	3.474818	0.553584	2.909954	3.503156	0.618204	-0.046608	1.971536	2.427159	3.651339	3.142744	-0.835047
H	3.281772	2.308250	1.820610	3.260273	2.331140	1.884240	1.121326	2.118443	3.736997	4.192854	2.037931	0.423192
H	2.649157	1.637974	-1.108225	2.662921	1.673252	-1.054868	0.491707	4.311791	2.933011	2.974500	1.217938	-2.275084
H	4.233090	1.680288	-0.366257	4.238131	1.713679	-0.294381	2.199939	4.056433	2.642977	4.674715	1.237576	-1.863125
H	3.034654	-0.141723	1.349065	3.017696	-0.116326	1.399060	0.054082	3.633598	0.456019	3.923226	-0.431892	0.141642
H	2.882680	-2.429009	1.189115	2.873528	-2.403110	1.227509	0.370652	4.493356	-1.636447	3.608412	-2.700852	0.203152
H	1.954627	-2.967546	-0.228059	1.968333	-2.938197	-0.205810	1.195839	6.065205	-1.633365	2.262796	-3.261129	-0.814935
H	3.714828	-3.087316	-0.233850	3.728761	-3.053963	-0.185196	2.078998	4.616350	-2.100959	3.925778	-3.483694	-1.357589
H	3.015760	-0.038600	-2.428925	3.056372	0.002395	-2.378085	3.334119	5.273016	1.437673	2.706185	-0.527092	-3.443303
H	3.842369	-1.601917	-2.405623	3.886254	-1.559081	-2.348819	3.845396	5.129298	-0.253424	3.390888	-2.153803	-3.557164
H	2.079776	-1.549680	-2.415249	2.123873	-1.510838	-2.385415	2.951315	6.548753	0.274459	1.727722	-1.944509	-3.008460
H	1.615394	1.617610	3.152518	1.578098	1.630381	3.191902	0.337511	0.287277	1.013385	2.787863	1.633519	2.117657
H	-0.674678	2.330825	3.916254	-0.720645	2.336473	3.932503	1.913110	-0.590876	-0.733180	0.738467	2.628419	3.193882
H	-1.318867	2.097908	2.294193	-1.345698	2.114890	2.301067	3.247403	-0.581648	0.410830	-0.228048	2.238097	1.775049
H	-0.386123	0.430275	5.430183	-0.452854	0.412342	5.446345	3.545869	-2.448097	1.972248	1.238763	0.909018	4.888758
H	-0.304590	-1.682284	8.047506	-0.932334	-3.528789	7.436646	4.736353	-4.951964	3.878320	0.987873	-2.668714	7.508242
H	-1.523311	-3.513173	4.163226	-1.571732	-3.514888	4.114563	0.835343	-5.778204	1.873656	-0.371196	-3.046760	4.433193
H	-3.245915	-1.185303	0.408168	-3.243122	-1.161180	0.366914	-2.214954	-2.547963	0.123931	-2.681650	-1.101091	0.773609
H	0.166776	-1.031155	-0.492798	0.183333	-1.001303	-0.487388	-0.196740	-3.004877	-2.734986	0.530579	-1.382925	-0.737561
H	-1.863564	-2.847643	-2.730365	-1.817225	-2.804799	-2.760953	-3.591972	-3.869915	-3.157682	-2.046668	-3.197629	-2.319048
H	-3.359840	-2.640632	-1.797538	-3.326444	-2.601691	-1.848334	-4.015052	-3.626347	-1.449841	-3.282910	-2.823743	-1.099823
H	-2.039303	-3.656428	-1.173046	-2.015637	-3.622080	-1.210823	-2.933473	-4.952898	-1.924491	-1.901414	-3.860358	-0.685635
H	-3.072029	-0.048181	-1.761622	-3.037232	-0.009256	-1.792811	-1.368408	-0.978493	-2.450544	-0.968408	0.255272	-1.516737
H	-1.549086	0.629529	-1.214208	-1.520868	0.663532	-1.221739	-2.567111	-1.648071	-3.549479	-1.426884	-0.862911	-2.793821
H	-0.413153	-0.383336	-3.250353	-0.359420	-0.339961	-3.248228	-4.321194	-1.370718	-1.726164	-3.830152	-0.755239	-1.943949
H	-1.986362	-0.899356	-3.835259	-1.925484	-0.850816	-3.856477	-3.106214	-0.624295	-0.698533	-3.326849	0.435574	-0.752940
H	-2.795960	1.504337	-3.864510	-2.731248	1.555484	-3.880616	-4.048765	0.447220	-3.441460	-3.185613	0.754027	-3.823555
H	-2.423728	3.521035	-4.867801	-2.343573	3.575807	-4.870557	-4.247664	2.698932	-3.769862	-3.529782	2.807620	-4.764929
H	-0.868468	4.255656	-4.428734	-0.791042	4.304271	-4.412061	-4.682727	3.594028	-2.299963	-4.617700	3.846192	-3.822335
H	-1.061428	3.498559	-6.005497	-0.969696	3.555123	-5.994301	-3.027719	3.647187	-2.895822	-2.883598	4.144073	-3.791266
H	0.950858	0.924034	-4.027520	1.015755	0.966599	-4.007110	-2.801119	1.244345	0.056092	-3.528102	2.441939	-0.438865
H	0.965754	1.909681	-5.497136	1.047566	1.957103	-5.473190	-2.186436	2.775181	-0.585545	-2.900789	3.917004	-1.189197
H	1.161848	2.681338	-3.929478	1.230204	2.723003	-3.901108	-3.836277	2.680635	0.016781	-4.629943	3.596765	-1.205736
H	-1.063837	-3.234019	0.935729	-1.066794	-3.210699	0.912626	-0.844575	-5.081765	-0.818617	-0.574084	-3.237859	1.202713
H	-2.773669	-3.296829	1.408115	-2.782929	-3.277215	1.361643	-1.788366	-4.821993	0.662201	-2.154249	-3.068173	1.994129

	1b-9			1b-10			1b-11			1b-12		
**Element**	**X**	**Y**	**Z**	**X**	**Y**	**Z**	**X**	**Y**	**Z**	**X**	**Y**	**Z**

C	0.937173	2.858206	3.200616	3.419852	2.152475	1.958591	2.283907	2.218974	2.891812	1.750364	2.826933	3.127311
C	-0.227428	2.621544	2.268073	2.223089	2.198869	1.039869	1.757075	2.163572	1.478516	1.275145	2.383696	1.764718
C	-1.401860	3.564052	2.454268	2.517742	2.536710	-0.407992	2.785568	2.365018	0.383131	2.330674	2.348260	0.676899
C	-2.204825	3.384060	3.769946	3.447499	1.531687	-1.137959	3.944472	1.334396	0.367939	3.447900	1.288942	0.891128
C	-3.026811	2.124303	3.796380	2.869009	0.145410	-1.246289	3.495386	-0.080862	0.114786	2.938990	-0.112909	1.108559
C	-3.012935	1.120799	4.686841	3.413105	-1.023451	-0.870214	3.760183	-1.185356	0.831834	2.967399	-1.155658	0.261861
C	-3.946633	-0.057237	4.534469	2.676538	-2.327171	-1.074406	3.235330	-2.536837	0.405111	2.445156	-2.510247	0.684303
C	-2.106511	1.042230	5.891523	4.775174	-1.179077	-0.237019	4.593887	-1.219480	2.090550	3.498802	-1.107324	-1.150714
C	-0.254759	1.689890	1.301406	0.962308	1.961153	1.433028	0.468786	1.960330	1.162410	0.018412	2.013586	1.475630
C	0.829428	0.719133	0.900602	0.478569	1.581774	2.819128	-0.693680	1.730266	2.108712	-1.172301	1.902645	2.402577
C	0.425013	-0.743111	1.007893	0.406258	0.079214	3.034118	-0.951083	0.261038	2.398096	-1.481797	0.462139	2.777322
C	-0.100659	-1.295180	2.182448	1.156426	-0.582917	4.009666	-0.892719	-0.279084	3.683576	-1.438511	0.001643	4.096376
C	-0.434705	-2.655015	2.279521	1.100896	-1.977621	4.176233	-1.104970	-1.645368	3.935535	-1.707114	-1.336730	4.430586
C	-0.967138	-3.253193	3.522221	1.890899	-2.676162	5.213586	-1.024829	-2.106108	5.338734	-1.668921	-1.819703	5.828418
O	-1.068407	-2.368116	4.557561	2.654158	-1.837271	5.968070	-1.248115	-3.443218	5.477404	-1.349981	-0.847587	6.727578
O	-1.289789	-4.418163	3.659684	1.889009	-3.877902	5.406937	-0.786693	-1.400398	6.301340	-1.892848	-2.962485	6.182372
C	-0.255213	-3.511992	1.175523	0.285039	-2.759495	3.338606	-1.372054	-2.528473	2.873340	-2.018365	-2.269392	3.424682
C	0.248993	-2.985780	0.002229	-0.472587	-2.124677	2.370086	-1.441737	-2.013905	1.588921	-2.071403	-1.835938	2.111827
C	0.580881	-1.627623	-0.062024	-0.410343	-0.735900	2.244726	-1.247632	-0.648298	1.377275	-1.814630	-0.495965	1.814214
O	1.079199	-1.263286	-1.281967	-1.218154	-0.251191	1.256231	-1.357248	-0.287936	0.065408	-1.911761	-0.218248	0.480321
C	0.871139	-2.379653	-2.210742	-2.016187	-1.358109	0.717683	-1.761081	-1.462583	-0.713159	-2.428776	-1.405742	-0.206782
C	2.102128	-2.454489	-3.134741	-2.020695	-1.201583	-0.820176	-0.871718	-1.491798	-1.974095	-1.639909	-1.556724	-1.527218
O	3.256173	-2.760177	-2.342651	-0.663470	-1.232243	-1.278241	0.494389	-1.623176	-1.558460	-0.250939	-1.723654	-1.217522
C	1.956303	-3.620314	-4.115567	-2.726501	-2.390727	-1.475740	-1.173164	-2.730586	-2.819955	-2.072172	-2.834644	-2.250722
C	2.355818	-1.113064	-3.864981	-2.689053	0.143912	-1.198885	-1.000821	-0.188252	-2.800216	-1.853022	-0.295569	-2.401173
C	1.228941	-0.579526	-4.774747	-2.670030	0.473924	-2.704709	-2.397565	0.162647	-3.355787	-1.038697	-0.267905	-3.710310
C	1.663730	0.647732	-5.533423	-3.088187	1.895845	-2.970167	-2.346992	1.367867	-4.259085	-1.215678	1.030944	-4.451865
C	1.202461	1.903904	-5.432364	-4.139116	2.348800	-3.671322	-2.902316	2.576152	-4.078544	-0.296567	1.968432	-4.730762
C	1.783327	3.008275	-6.284304	-4.387194	3.832080	-3.817616	-2.724929	3.666735	-5.109083	-0.676684	3.221697	-5.484215
C	0.098944	2.349897	-4.503878	-5.153125	1.477715	-4.372781	-3.734631	2.978076	-2.885069	1.161624	1.891088	-4.347874
C	0.611959	-3.618897	-1.320496	-1.371375	-2.652532	1.276484	-1.624196	-2.676392	0.243227	-2.298805	-2.567991	0.809914
H	0.632065	2.751359	4.249565	4.064725	3.028029	1.804338	2.947247	3.083781	3.027903	2.271251	3.791892	3.060709
H	1.771731	2.173576	3.033320	3.143593	2.120680	3.015542	1.489710	2.283370	3.639701	0.937841	2.934407	3.849980
H	1.317966	3.883269	3.090911	4.036036	1.266359	1.757764	2.877129	1.325397	3.125580	2.470826	2.113385	3.549420
H	-1.028710	4.599207	2.427058	1.574285	2.614962	-0.963285	2.284749	2.351995	-0.593405	1.848129	2.151270	-0.287810
H	-2.092159	3.461080	1.607526	2.993545	3.528239	-0.459504	3.233887	3.365122	0.489877	2.813398	3.334524	0.597181
H	-1.531456	3.445336	4.630912	3.621904	1.929328	-2.149534	4.636503	1.640031	-0.431901	4.125431	1.337013	0.031662
H	-2.881719	4.247896	3.856005	4.427007	1.517183	-0.649628	4.513746	1.409819	1.299749	4.049506	1.580275	1.763944
H	-3.733258	2.040673	2.967302	1.881150	0.106654	-1.712027	2.894689	-0.207158	-0.789414	2.498879	-0.288535	2.090807
H	-4.595850	-0.164303	5.415519	1.677932	-2.180128	-1.495305	2.600932	-2.477132	-0.483557	2.074135	-2.499512	1.714357
H	-4.587706	0.040855	3.652597	3.238981	-2.995165	-1.742691	4.063061	-3.229216	0.193515	1.630413	-2.840275	0.027278
H	-3.380049	-0.993237	4.443492	2.567297	-2.865360	-0.121888	2.646774	-2.998117	1.211086	3.236114	-3.270977	0.617204
H	-1.443006	1.904539	5.993032	5.302727	-0.233058	-0.094191	4.970006	-0.239772	2.394167	2.705127	-1.382689	-1.858938
H	-2.695355	0.963973	6.816474	4.692120	-1.671915	0.741542	4.013983	-1.633406	2.927050	3.881546	-0.124407	-1.437245
H	-1.483115	0.139421	5.838493	5.414108	-1.826103	-0.854390	5.460858	-1.882191	1.959006	4.307115	-1.838686	-1.290029
H	-1.158641	1.620601	0.694856	0.179827	2.009646	0.677575	0.206584	1.927574	0.106317	-0.190059	1.711314	0.451022
H	1.111261	0.915589	-0.141446	1.120986	2.011376	3.594696	-0.537632	2.246519	3.061435	-1.022843	2.477475	3.321396
H	1.737150	0.880555	1.493552	-0.520937	2.012334	2.967809	-1.595818	2.168905	1.660815	-2.048891	2.335333	1.899547
H	-0.265492	-0.645406	3.035373	1.802117	-0.003406	4.661389	-0.677618	0.364278	4.531536	-1.193481	0.700018	4.889910
H	-1.423011	-2.888392	5.301872	3.116438	-2.414360	6.603174	-1.167323	-3.617850	6.432927	-1.358517	-1.290042	7.596062
H	-0.514818	-4.560876	1.276002	0.272076	-3.836416	3.472170	-1.515023	-3.585774	3.068205	-2.206994	-3.301164	3.703259
H	-0.024760	-2.134627	-2.794107	-3.033204	-1.226413	1.107180	-2.808914	-1.304913	-0.992355	-3.483170	-1.199821	-0.430643
H	3.336022	-2.042932	-1.690849	-0.176433	-0.577840	-0.746536	0.669932	-0.892858	-0.939310	0.010989	-0.973054	-0.655350
H	2.825488	-3.651245	-4.780036	-2.668773	-2.307077	-2.563944	-0.546294	-2.723483	-3.717214	-1.474857	-2.975512	-3.154928
H	1.054506	-3.522967	-4.728384	-3.782943	-2.425511	-1.186946	-2.222198	-2.760769	-3.131322	-3.128228	-2.783360	-2.538162
H	1.910704	-4.572643	-3.579136	-2.254317	-3.335654	-1.192943	-0.950208	-3.645448	-2.263569	-1.923537	-3.714024	-1.617645
H	2.579764	-0.348684	-3.110962	-3.725686	0.145388	-0.835071	-0.655503	0.647617	-2.180475	-2.924517	-0.199670	-2.627909
H	3.271757	-1.247136	-4.454252	-2.174350	0.943688	-0.651919	-0.287595	-0.274939	-3.629607	-1.588817	0.585549	-1.804973
H	0.945564	-1.356533	-5.499710	-1.645604	0.319006	-3.070177	-2.786854	-0.689456	-3.931929	0.010904	-0.461284	-3.473250
H	0.335539	-0.371964	-4.177362	-3.303819	-0.229561	-3.253061	-3.098160	0.324264	-2.530267	-1.365950	-1.091002	-4.361496
H	2.473988	0.469674	-6.243446	-2.438102	2.644608	-2.511909	-1.761436	1.219157	-5.168672	-2.237457	1.222120	-4.787332
H	2.576949	2.642467	-6.943402	-3.630586	4.426818	-3.296091	-2.118996	3.333582	-5.957552	-1.740560	3.237842	-5.741356
H	1.009269	3.474381	-6.910408	-4.385410	4.132199	-4.875211	-3.695630	4.006694	-5.497126	-0.455908	4.121838	-4.893027
H	2.201684	3.811041	-5.660714	-5.373153	4.110541	-3.418893	-2.240193	4.550577	-4.670758	-0.100333	3.314504	-6.415732
H	0.454896	3.148792	-3.838681	-4.971020	0.408341	-4.243517	-3.833613	2.188194	-2.137002	1.420025	0.979038	-3.805099
H	-0.740095	2.773595	-5.073490	-6.166810	1.692748	-4.006553	-3.297060	3.855218	-2.388544	1.800272	1.942103	-5.240869
H	-0.294072	1.546442	-3.876758	-5.164295	1.687091	-5.451679	-4.745723	3.273422	-3.198741	1.441970	2.748188	-3.719379
H	1.525268	-4.219166	-1.229592	-0.778067	-3.152249	0.501606	-0.740809	-3.271695	-0.014931	-1.434833	-3.197534	0.565450
H	-0.177541	-4.255442	-1.731245	-2.127588	-3.358649	1.632975	-2.501237	-3.329483	0.193186	-3.190543	-3.202131	0.814526

		**1b-13**			**1b-14**			**1b-15**			**1b-16**	
**Element**	**X**	**Y**	**Z**	**X**	**Y**	**Z**	**X**	**Y**	**Z**	**X**	**Y**	**Z**
C	3.385630	4.338897	0.528798	2.398796	4.399301	2.279928	2.424284	2.476817	2.989972	3.481085	2.140319	2.007392
C	3.376951	3.237594	-0.505088	1.016534	4.272616	1.683846	1.838660	2.376725	1.602425	2.361466	2.201044	0.996870

C	4.613471	3.168498	-1.381547	0.087248	5.439399	1.961845	2.824778	2.515969	0.459531	2.772271	2.545278	-0.421119
C	5.913799	2.713799	-0.666640	-0.381468	5.588806	3.433644	3.954141	1.452920	0.431835	3.771079	1.555186	-1.075619
C	5.904028	1.258308	-0.285988	-1.359641	4.526755	3.856151	3.455468	0.044303	0.243028	3.222966	0.161966	-1.240299
C	6.068876	0.696350	0.921151	-1.283299	3.661208	4.878477	3.707977	-1.042198	0.991216	3.756808	-1.002562	-0.836589
C	6.041176	-0.805602	1.082618	-2.400580	2.678514	5.140810	3.125910	-2.389513	0.630738	3.061523	-2.315986	-1.111205
C	6.288407	1.453860	2.208425	-0.128970	3.561282	5.846280	4.579392	-1.059295	2.224434	5.069497	-1.143737	-0.103454
C	2.360782	2.382275	-0.702129	0.605034	3.247599	0.920165	0.535712	2.181946	1.347060	1.071015	1.976366	1.288403
C	1.030765	2.322950	0.009952	1.391894	2.044941	0.458867	-0.589386	2.002488	2.347713	0.473756	1.604360	2.631626
C	0.759625	0.990473	0.691146	0.806053	0.711825	0.898106	-0.854513	0.547264	2.695093	0.364815	0.103601	2.843719
C	1.621941	0.454953	1.654429	0.499026	0.428620	2.233651	-0.738957	0.044980	3.991990	1.013189	-0.561573	3.887629
C	1.345666	-0.754460	2.311247	-0.002870	-0.820406	2.633031	-0.958922	-1.309390	4.296207	0.926018	-1.954636	4.054341
C	2.233900	-1.311633	3.353130	-0.302685	-1.140719	4.044593	-0.814431	-1.729169	5.707112	1.607860	-2.656533	5.163432
O	3.329796	-0.538172	3.609687	-0.020194	-0.122662	4.909839	-1.051415	-3.057434	5.898076	2.307607	-1.822391	5.982238
O	2.045312	-2.352214	3.954715	-0.750668	-2.198561	4.445399	-0.516159	-0.999146	6.634162	1.573908	-3.857143	5.361116
C	0.181525	-1.481989	1.996352	-0.227055	-1.832421	1.679694	-1.293580	-2.219259	3.276580	0.182622	-2.731451	3.147429
C	-0.679247	-0.977326	1.040919	0.057279	-1.570632	0.353325	-1.420790	-1.742218	1.982107	-0.473838	-2.093598	2.109590
C	-0.385135	0.238216	0.412145	0.567991	-0.320565	-0.014295	-1.216114	-0.387199	1.718961	-0.384737	-0.706065	1.984894
O	-1.332414	0.611601	-0.494208	0.811645	-0.217595	-1.351667	-1.386914	-0.064615	0.403708	-1.091411	-0.216694	0.924649
C	-2.439408	-0.353145	-0.448301	0.333162	-1.437845	-2.015147	-1.854408	-1.253863	-0.315279	-1.833466	-1.319798	0.305191
C	-2.822208	-0.686497	-1.905074	1.412409	-1.863132	-3.031908	-1.038692	-1.335363	-1.623272	-1.651848	-1.179424	-1.221237
O	-3.179049	0.544785	-2.547088	1.599856	-0.770147	-3.940779	0.347659	-1.459460	-1.278569	-0.251740	-1.275648	-1.515722
C	-1.660997	-1.337529	-2.674368	2.752475	-2.188222	-2.351848	-1.392570	-2.601549	-2.405658	-2.317451	-2.344398	-1.956355
C	-4.074136	-1.582169	-1.972575	0.946843	-3.056230	-3.888523	-1.209271	-0.062356	-2.488656	-2.161148	0.188648	-1.738548
C	-5.347649	-1.012591	-1.312767	-0.347211	-2.839092	-4.700921	-2.616929	0.225442	-3.052927	-3.672231	0.475637	-1.608268
C	-6.563342	-1.831702	-1.657041	-0.571097	-3.949288	-5.693227	-2.654191	1.530247	-3.805795	-4.012802	1.866950	-2.076187
C	-7.367948	-2.531440	-0.842059	-1.579983	-4.831937	-5.758717	-2.896450	1.742044	-5.108606	-4.783939	2.241031	-3.108793
C	-8.547665	-3.297056	-1.394496	-1.616690	-5.881630	-6.844948	-2.876940	3.141541	-5.677775	-4.994088	3.704044	-3.422428
C	-7.209019	-2.632791	0.655820	-2.751370	-4.882150	-4.807667	-3.206442	0.664669	-6.119498	-5.505354	1.296443	-4.039399
C	-1.969710	-1.514394	0.466375	-0.037597	-2.432585	-0.884463	-1.682826	-2.441138	0.668343	-1.273640	-2.619399	0.940581
H	3.499099	5.319712	0.046249	2.351525	4.492486	3.372553	3.109880	3.331715	3.063743	4.143928	3.010529	1.908831
H	2.477916	4.370570	1.135627	3.047484	3.550030	2.053701	1.663143	2.588459	3.766166	3.121454	2.106933	3.038915
H	4.235028	4.231142	1.215587	2.896228	5.307645	1.912186	3.008546	1.580581	3.235967	4.103303	1.249355	1.851798
H	4.426288	2.494412	-2.226905	0.599070	6.370862	1.675728	2.283463	2.484666	-0.494732	1.877374	2.616936	-1.052445
H	4.796905	4.164557	-1.812354	-0.799129	5.363039	1.319603	3.303120	3.506234	0.514298	3.240163	3.542016	-0.429285
H	6.745427	2.889818	-1.366012	0.485548	5.628311	4.100828	4.621239	1.713206	-0.404344	4.024320	1.961454	-2.066968
H	6.109735	3.356069	0.197961	-0.870668	6.571256	3.518569	4.562278	1.541305	1.337593	4.706659	1.549816	-0.507474
H	5.744874	0.582288	-1.129239	-2.254886	4.477965	3.231996	2.822139	-0.094166	-0.636775	2.274459	0.113107	-1.780881
H	5.878948	-1.316476	0.128175	-3.218430	2.788132	4.421504	2.466159	-2.339881	-0.239918	2.094872	-2.181468	-1.604389
H	6.984977	-1.175253	1.509033	-2.031536	1.646006	5.084826	3.923119	-3.117140	0.420243	3.685375	-2.964854	-1.742880
H	5.243068	-1.107918	1.773379	-2.816389	2.812126	6.149994	2.547714	-2.800685	1.470671	2.890748	-2.868754	-0.176316
H	6.311773	2.538299	2.076045	0.309001	2.554565	5.811386	4.999829	-0.083337	2.477890	5.567834	-0.191171	0.091265
H	5.492814	1.217136	2.927915	0.667808	4.282383	5.648105	4.011680	-1.419219	3.093662	4.921235	-1.651757	0.859528
H	7.236042	1.154863	2.678534	-0.472441	3.719199	6.878506	5.417412	-1.758227	2.092841	5.764930	-1.771175	-0.678564
H	2.479144	1.632248	-1.485210	-0.420336	3.275694	0.549496	0.230045	2.113423	0.304386	0.351561	2.034900	0.473461
H	0.949038	3.123578	0.753608	1.433270	2.051078	-0.637492	-0.385621	2.546608	3.275544	1.057199	2.027882	3.455687
H	0.228670	2.503651	-0.716320	2.432286	2.104154	0.799012	-1.503293	2.440260	1.923570	-0.528848	2.048156	2.699435
H	2.532396	0.993407	1.895355	0.644909	1.204832	2.977341	-0.471403	0.709785	4.807861	1.602675	0.014099	4.593776
H	3.818742	-1.013571	4.306371	-0.258246	-0.464999	5.791077	-0.924652	-3.204699	6.853217	2.701984	-2.401593	6.659780
H	-0.009995	-2.417208	2.512604	-0.614297	-2.790562	2.011097	-1.443164	-3.267593	3.510929	0.144858	-3.807475	3.283548
H	-3.272799	0.189605	0.006686	-0.557018	-1.131376	-2.571873	-2.913935	-1.084794	-0.537823	-2.885129	-1.176950	0.577770
H	-2.431928	1.151665	-2.413847	1.788045	0.012231	-3.396081	0.557581	-0.711940	-0.691470	0.195476	-0.606875	-0.967791
H	-1.952103	-1.455102	-3.722369	3.503904	-2.380508	-3.123370	-0.830549	-2.622374	-3.344801	-2.208170	-2.207572	-3.036930
H	-1.397969	-2.325521	-2.280837	2.692441	-3.071712	-1.706843	-2.460968	-2.643476	-2.640981	-3.384912	-2.410363	-1.722305
H	-0.767046	-0.706611	-2.635218	3.093551	-1.344651	-1.742949	-1.126878	-3.497076	-1.836877	-1.844222	-3.293591	-1.689717
H	-4.277044	-1.753707	-3.037427	1.765617	-3.277700	-4.584997	-0.884140	0.799693	-1.892713	-1.605023	0.977693	-1.216855
H	-3.841075	-2.564158	-1.541632	0.838920	-3.941438	-3.248914	-0.500280	-0.151067	-3.320218	-1.873487	0.251406	-2.794649
H	-5.217930	-0.947352	-0.227136	-1.206726	-2.739083	-4.029408	-2.942394	-0.603854	-3.688724	-4.245102	-0.275453	-2.161021
H	-5.484656	0.014448	-1.674949	-0.253601	-1.884937	-5.235319	-3.338616	0.282842	-2.224411	-3.973148	0.381299	-0.554173
H	-6.797566	-1.851399	-2.723562	0.212968	-4.033782	-6.448766	-2.438057	2.405766	-3.190199	-3.546504	2.660752	-1.489084
H	-8.627259	-3.196807	-2.481535	-0.751872	-5.810013	-7.512031	-2.651276	3.891224	-4.912912	-4.456751	4.353496	-2.724240
H	-8.472814	-4.367591	-1.155840	-1.634155	-6.894382	-6.417600	-2.127229	3.233760	-6.476385	-4.654529	3.943569	-4.440112
H	-9.490650	-2.946593	-0.951227	-2.525644	-5.788205	-7.456186	-3.844970	3.398637	-6.130784	-6.060176	3.968960	-3.381464
H	-6.343017	-2.088022	1.039258	-2.702758	-4.129210	-4.017434	-3.202898	-0.342909	-5.697276	-5.333204	0.242388	-3.809588
H	-8.100824	-2.245162	1.167974	-3.697097	-4.737757	-5.348621	-4.191698	0.833432	-6.576260	-6.589507	1.473818	-4.007963
H	-7.108628	-3.682927	0.964015	-2.818190	-5.868690	-4.327977	-2.477582	0.685893	-6.941618	-5.194811	1.467554	-5.079523
H	-1.804371	-2.441155	-0.095887	0.655604	-3.280909	-0.837288	-0.822143	-3.056669	0.382449	-0.620283	-3.143505	0.233673
H	-2.716933	-1.736783	1.235588	-1.039622	-2.847124	-1.037898	-2.569686	-3.082570	0.683939	-2.076393	-3.303402	1.233270

	**1b-17**			**1b-18**			**1b-19**		
**Element**	**X**	**Y**	**Z**	**X**	**Y**	**Z**	**X**	**Y**	**Z**
C	1.986994	2.317746	-1.380648	2.050471	2.468573	-1.061695	3.394762	1.509403	2.750154
C	2.430567	1.944376	0.013350	2.141967	2.258245	0.430565	2.528940	1.536144	1.514000
C	2.054346	2.935179	1.099364	1.388559	3.269936	1.274112	3.258211	1.734203	0.199496
C	0.541552	2.955548	1.454943	-0.157851	3.122499	1.226222	4.222724	0.580099	-0.191975

C	0.056702	1.654568	2.036647	-0.641950	1.816880	1.798032	3.592748	-0.789441	-0.192718
C	-0.614067	1.448033	3.181517	-1.570054	1.611403	2.746550	3.230240	-1.544361	-1.243539
C	-1.002771	0.050404	3.605315	-1.909161	0.210798	3.201981	2.667944	-2.933418	-1.040652
C	-1.046951	2.528572	4.142726	-2.356060	2.695604	3.443364	3.334922	-1.120011	-2.689114
C	3.114758	0.832318	0.324592	2.830451	1.268970	1.021311	1.195834	1.385531	1.509309
C	3.556366	-0.290316	-0.587690	3.615164	0.148981	0.375269	0.268645	1.114101	2.673621
C	3.094103	-1.647173	-0.090364	3.181541	-1.215836	0.876280	-0.187836	-0.335072	2.724525
C	3.960276	-2.543923	0.543372	3.934207	-1.946886	1.798246	0.074070	-1.168810	3.812950
C	3.525659	-3.787542	1.029987	3.521168	-3.197315	2.285443	-0.330700	-2.513937	3.845373
C	4.442762	-4.732086	1.704863	4.397403	-3.879244	3.263260	-0.004773	-3.301003	5.054738
O	5.722223	-4.273565	1.791993	3.904540	-5.079962	3.677285	-0.442050	-4.589773	4.989057
O	4.126203	-5.819153	2.151370	5.456178	-3.449600	3.681854	0.590345	-2.882427	6.030340
C	2.181600	-4.174286	0.878840	2.313543	-3.765147	1.839314	-1.013870	-3.074356	2.750563
C	1.305463	-3.303505	0.256685	1.548598	-3.058345	0.926761	-1.289706	-2.265320	1.660531
C	1.764991	-2.067715	-0.208427	1.982574	-1.811620	0.467965	-0.887846	-0.928278	1.667553
O	0.783851	-1.323479	-0.800360	1.121411	-1.245100	-0.429031	-1.237122	-0.259630	0.529287
C	-0.503433	-1.990055	-0.559118	-0.106011	-2.051829	-0.456246	-2.065660	-1.142269	-0.296270
C	-1.346179	-1.867615	-1.844124	-0.584373	-2.134289	-1.919430	-1.590714	-0.991104	-1.756464
O	-0.661944	-2.545772	-2.904784	0.423941	-2.796931	-2.692323	-0.236541	-1.451783	-1.855410
C	-2.678107	-2.603225	-1.675862	-1.829357	-3.019330	-2.020437	-2.403926	-1.906030	-2.675393
C	-1.556359	-0.392712	-2.264574	-0.826821	-0.733871	-2.532802	-1.636206	0.482639	-2.228470
C	-2.350826	0.511918	-1.298667	-1.938508	0.131741	-1.902406	-3.002653	1.198819	-2.178200
C	-2.478801	1.913221	-1.837714	-2.062406	1.461293	-2.600651	-2.937021	2.563318	-2.814498
C	-3.590725	2.604953	-2.131208	-3.121449	1.982256	-3.239414	-3.071074	3.765936	-2.234266
C	-3.501903	4.011911	-2.674961	-3.036904	3.344706	-3.887216	-2.961619	5.031826	-3.051736
C	-5.000750	2.093887	-1.959933	-4.465722	1.310275	-3.381123	-3.336830	3.994787	-0.766026
C	-0.151434	-3.431917	-0.119061	0.265338	-3.400215	0.207705	-1.926635	-2.557384	0.321726
H	2.458389	3.260232	-1.692929	2.489519	3.437120	-1.339502	4.035131	2.401055	2.793677
H	2.230534	1.560584	-2.128963	2.560734	1.694774	-1.639156	2.817056	1.466432	3.676587
H	0.903029	2.485458	-1.421513	1.005434	2.495146	-1.396396	4.069105	0.642514	2.745558
H	2.622473	2.717438	2.011399	1.712436	3.194608	2.318795	2.523103	1.858600	-0.604482
H	2.339249	3.948782	0.780032	1.640949	4.285407	0.934068	3.847137	2.663652	0.239628
H	0.363996	3.789491	2.141917	-0.598292	3.975407	1.753000	4.651039	0.819655	-1.171511
H	-0.034529	3.187255	0.546361	-0.491583	3.210228	0.181223	5.067998	0.571549	0.510724
H	0.307677	0.778049	1.438249	-0.148746	0.938007	1.381264	3.428951	-1.214485	0.798258
H	-0.577197	-0.198395	4.587870	-2.970642	-0.018764	3.029891	2.600581	-3.193194	0.020857
H	-2.093228	-0.046675	3.707656	-1.308273	-0.543734	2.683730	1.667962	-3.023698	-1.483195
H	-0.658523	-0.703863	2.890131	-1.739010	0.093270	4.281664	3.302243	-3.684413	-1.533286
H	-0.738194	3.530914	3.837463	-2.083448	3.703764	3.123249	3.768388	-0.124908	-2.818206
H	-2.140869	2.535165	4.250310	-3.433145	2.568895	3.264074	3.943899	-1.832020	-3.263345
H	-0.639706	2.340809	5.146084	-2.215800	2.637605	4.531851	2.337690	-1.118272	-3.150030
H	3.391389	0.686279	1.369522	2.832492	1.236926	2.111549	0.692783	1.422370	0.544997
H	3.195983	-0.130582	-1.608686	3.526185	0.185767	-0.714968	0.736594	1.365306	3.630009
H	4.654125	-0.305477	-0.641913	4.682487	0.270830	0.607184	-0.614491	1.761955	2.575774
H	5.002809	-2.268310	0.666247	4.874943	-1.546021	2.164188	0.606612	-0.779911	4.675686
H	6.216052	-4.978567	2.249377	4.563988	-5.423696	4.307416	-0.167714	-4.996023	5.831531
H	1.867847	-5.144475	1.250483	2.001138	-4.736726	2.206450	-1.310318	-4.117424	2.770677
H	-0.981841	-1.443103	0.261864	-0.840511	-1.512219	0.153175	-3.095554	-0.779667	-0.199496
H	0.204891	-2.113022	-2.988116	1.231541	-2.263458	-2.598318	0.298430	-0.919086	-1.240827
H	-3.286494	-2.471496	-2.576220	-2.185952	-3.034479	-3.055196	-2.066180	-1.780947	-3.708918
H	-3.242625	-2.224972	-0.817587	-2.638233	-2.652762	-1.380329	-3.473200	-1.676217	-2.630127
H	-2.512205	-3.675420	-1.537570	-1.596314	-4.048141	-1.731371	-2.263001	-2.955788	-2.401822
H	-0.569267	0.055782	-2.436673	0.118570	-0.177286	-2.493781	-0.923694	1.059697	-1.627097
H	-2.063510	-0.414927	-3.236519	-1.052412	-0.893135	-3.593993	-1.253434	0.492738	-3.256863
H	-3.336722	0.078220	-1.104531	-2.890322	-0.408158	-1.921817	-3.751156	0.598418	-2.715676
H	-1.834080	0.557703	-0.328623	-1.705769	0.315094	-0.843017	-3.347181	1.269073	-1.141685
H	-1.523112	2.410412	-2.018054	-1.149760	2.061079	-2.582149	-2.736642	2.546673	-3.887738
H	-3.987833	4.087989	-3.658052	-2.048278	3.797349	-3.761311	-2.772284	4.821379	-4.109059
H	-4.020198	4.724413	-2.017588	-3.249008	3.285037	-4.964172	-3.882508	5.628218	-2.983344
H	-2.464644	4.344332	-2.783255	-3.782046	4.032413	-3.462663	-2.149377	5.673167	-2.681377
H	-5.049872	1.069237	-1.584486	-4.506809	0.318285	-2.925519	-3.385463	3.072238	-0.183042
H	-5.562217	2.732644	-1.263785	-5.254820	1.922277	-2.922144	-2.551608	4.625700	-0.327060
H	-5.542544	2.127843	-2.915392	-4.734649	1.204345	-4.441337	-4.283187	4.534856	-0.622215
H	-0.276233	-4.127642	-0.957400	0.440548	-4.166057	-0.557365	-1.268234	-3.182329	-0.292820
H	-0.786938	-3.770730	0.704764	-0.527525	-3.755670	0.872991	-2.896097	-3.059321	0.400720

3.2. Compound 2

Table S2.1. Detailed DP4+ probability of **2a** (Isomer 1) and **2b** (Isomer 2)

Functional	Solvent?	Basis Set	Type of Data
B3LYP	PCM	6-31+G(d,p)	Shielding Tensors

	Isomer 1	Isomer 2	Isomer 3	Isomer 4	Isomer 5	Isomer 6
sDP4+ (H data)	98.70%	1.30%	-	-	-	-
sDP4+ (C data)	0.80%	99.20%	-	-	-	-
sDP4+ (all data)	37.91%	62.09%	-	-	-	-
uDP4+ (H data)	99.18%	0.82%	-	-	-	-
uDP4+ (C data)	29.59%	70.41%	-	-	-	-
uDP4+ (all data)	98.08%	1.92%	-	-	-	-
DP4+ (H data)	99.99%	0.01%	-	-	-	-
DP4+ (C data)	0.34%	99.66%	-	-	-	-
DP4+ (all data)	96.89%	3.11%	-	-	-	-

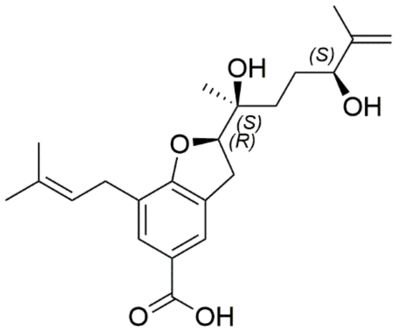

2*R*,1'*S*,4'*S*-**2** **(2a)**

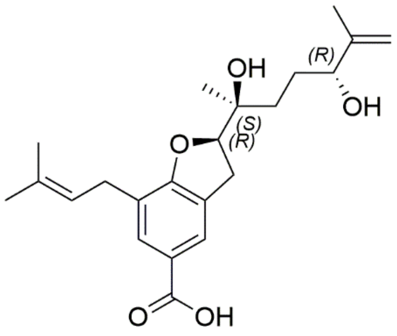

2*R*,1'*S*,4'*R*-**2** (**2b**)

Table S2.2. Experimental and calculated ^{1}H-NMR chemical shifts (δ in ppm) of **2a** and **2b**

Proton									
**2**		**2a (2*R*,1'*S*,4'*S*)**				**2b (2*R*,1'*S*,4'*R*)**			
No.	δ_H Exp. (mult., *J* in Hz)	δ_H Calc.	|Δ*δ*|	δ_H Calc. CORRECTED	|Δδ|	δ_H Calc.	|Δδ|	δ_H Calc. CORRECTED	|Δδ|
2	4.73 (1H, t, 8.9)	4.87	0.14	4.64	0.09	5.29	0.56	5.01	0.28
3	3.19 (2H, m)	3.44	0.25	3.25	0.06	3.44	0.25	3.23	0.04
4	7.72 (1H, s)	8.09	0.37	7.77	0.05	8.13	0.41	7.72	0.00
6	7.72 (1H, s)	8.02	0.30	7.70	0.02	8.02	0.30	7.61	0.11
2'	1.60 (2H, m)	1.92	0.32	1.77	0.17	1.95	0.35	1.80	0.20
3'	1.67 (2H, m)	1.90	0.23	1.76	0.09	1.93	0.26	1.78	0.11
4'	4.10 (1H, dd, 7.3, 4.6)	4.39	0.29	4.18	0.08	4.41	0.31	4.16	0.06
6'α	4.96 (1H, s)	5.16	0.20	4.92	0.04	5.18	0.22	4.89	0.07
6'β	4.85 (1H, s)	5.02	0.17	4.79	0.06	5.01	0.16	4.74	0.11
7'	1.26 (3H, s)	1.23	0.03	1.10	0.16	1.09	0.17	0.98	0.28
8'	1.72 (3H, s)	1.92	0.20	1.77	0.05	1.93	0.21	1.79	0.07
1"	3.26 (2H, m)	3.37	0.11	3.18	0.08	3.37	0.11	3.17	0.09
2"	5.25 (1H, t, 7.4)	5.53	0.28	5.28	0.03	5.53	0.28	5.24	0.01
4"	1.72 (3H, s)	1.88	0.16	1.74	0.02	1.88	0.16	1.73	0.01
5"	1.70 (3H, s)	1.74	0.04	1.60	0.10	1.73	0.03	1.60	0.10
		**MAE**	**0.21**	**CMAE**	**0.07**	**MAE**	**0.252**	**CMAE**	**0.10**

Table S2.3. Experimental and calculated ^{13}C-NMR chemical shifts (δ in ppm) of **2a** and **2b**

Carbon									
	**2**	**2a (2*R*,1'*S*,4'*S*)**				**2b (2*R*,1'*S*,4'*R*)**			
No.	δ_C Exp.	δ_C Calc.	$\|\Delta\delta\|$	δ_C Calc. CORRECTED	$\|\Delta\delta\|$	δ_C Calc.	$\|\Delta\delta\|$	δ_C Calc. CORRECTED	$\|\Delta\delta\|$
2	89.57	92.43	2.86	91.70	2.13	88.41	1.16	87.71	1.86
3	30.08	33.95	3.87	30.79	0.71	33.92	3.84	30.86	0.78
3α	127.41	127.13	0.28	127.83	0.42	126.78	0.63	127.74	0.33
4	125.23	123.36	1.87	123.91	1.32	123.32	1.91	124.13	1.10
5	121.50	118.76	2.74	119.12	2.38	118.62	2.88	119.23	2.27
6	131.62	129.76	1.86	130.57	1.05	129.84	1.78	130.94	0.68
7	123.34	122.82	0.52	123.34	0.00	122.82	0.52	123.61	0.27
7α	162.55	160.36	2.19	162.44	0.11	160.59	1.96	163.02	0.47
1'	73.53	76.47	2.94	75.08	1.55	75.97	2.44	74.74	1.21
2'	33.13	41.74	8.61	38.89	5.76	40.95	7.82	38.20	5.07
3'	28.45	34.35	5.90	31.20	2.75	33.62	5.17	30.55	2.10
4'	75.98	81.91	5.93	80.74	4.76	81.60	5.62	80.61	4.63
5'	147.43	151.44	4.01	153.15	5.72	151.10	3.67	153.12	5.69
6'	111.37	107.80	3.57	107.71	3.66	108.64	2.73	108.82	2.55
7'	22.83	20.34	2.49	16.61	6.22	23.11	0.28	19.58	3.25
8'	18.23	18.96	0.73	15.17	3.06	18.27	0.04	14.54	3.69
1''	28.45	34.68	6.23	31.55	3.10	34.53	6.08	31.50	3.05
2''	121.50	121.99	0.49	122.48	0.98	121.80	0.30	122.55	1.05
3''	133.45	132.76	0.69	133.69	0.24	132.78	0.67	134.00	0.55
4''	26.01	27.66	1.65	24.24	1.77	27.73	1.72	24.40	1.61
5''	18.07	19.21	1.14	15.44	2.63	19.22	1.15	15.53	2.54
COOH	171.43	163.32	8.11	165.53	5.90	163.25	8.18	165.79	5.64
		**MAE**	**3.12**	**CMAE**	**2.56**	**MAE**	**2.75**	**CMAE**	**2.29**

Table S2.4. Calculated conformational analysis of the **2a** at B3LYP/6-31g(d) level

conformer	3D conformer	G (Hartree)	ΔG (kcal/mol)	Population
2a-1	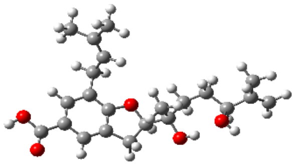	-1232.090032	0	13.984%
2a-2	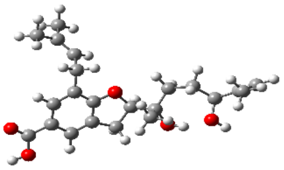	-1232.089537	0.31061745	8.278%
2a-3	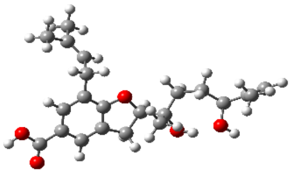	-1232.089483	0.34450299	7.818%
2a-4	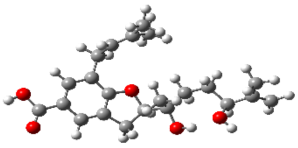	-1232.089394	0.40035138	7.115%
2a-5	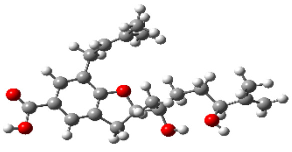	-1232.089373	0.41352909	6.958%
2a-6	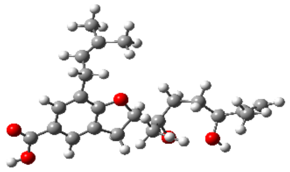	-1232.089181	0.53401101	5.678%
2a-7	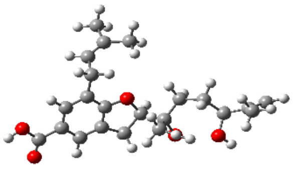	-1232.089141	0.55911141	5.442%
2a-8	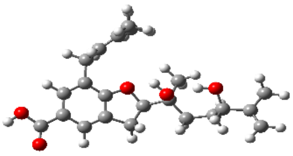	-1232.089045	0.61935237	4.916%

2a-9	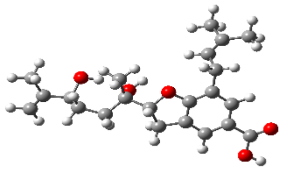	-1232.089037	0.62437245	4.874%
2a-10	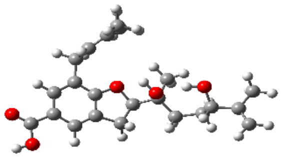	-1232.089034	0.62625498	4.859%
2a-11	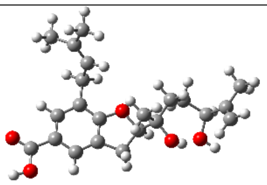	-1232.088971	0.66578811	4.545%
2a-12	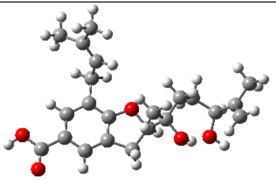	-1232.088828	0.75552204	3.906%
2a-13	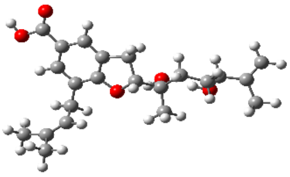	-1232.088626	0.88227906	3.154%
2a-14	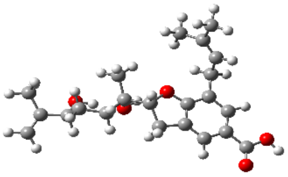	-1232.088453	0.99083829	2.626%
2a-15	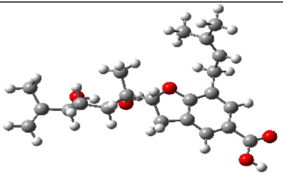	-1232.088406	1.02033126	2.498%
2a-16	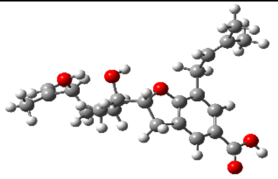	-1232.087856	1.36546176	1.395%
2a-17	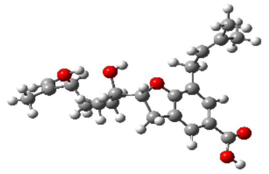	-1232.087805	1.39746477	1.322%

2a-18	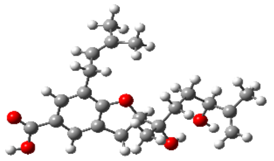	-1232.087686	1.47213846	1.165%
2a-19	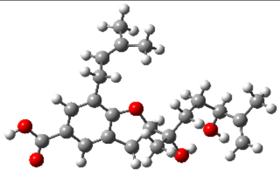	-1232.087663	1.48657119	1.137%

Table S2.5. Cartesian coordinates of low-energy conformers of **2a**

	**2a-1**			**2a-2**			**2a-3**			**2a-4**		
**Element**	**X**	**Y**	**Z**	**X**	**Y**	**Z**	**X**	**Y**	**Z**	**X**	**Y**	**Z**
O	6.337981	0.580920	2.824489	6.079341	-1.713101	2.405164	6.639596	0.363307	1.386538	6.376140	1.808487	2.980958
C	5.458060	-0.434218	3.055620	5.809526	-0.547360	1.751553	5.788322	-0.618912	1.797373	5.476247	0.915162	3.481699
C	4.086066	-0.139769	2.591642	4.370854	-0.356196	1.471799	4.372948	-0.343367	1.473118	4.141769	1.011764	2.854549
C	3.100806	-1.125495	2.790326	3.402611	-1.306502	1.848310	3.414709	-1.302513	1.851624	3.139021	0.132786	3.304963
C	1.807967	-0.871381	2.369869	2.074331	-1.055334	1.547686	2.083535	-1.068292	1.557493	1.880030	0.208129	2.737266
C	0.544413	-1.695704	2.431724	0.814799	-1.848194	1.803176	0.833084	-1.873820	1.817246	0.608521	-0.563156	2.996087
C	-0.513487	-0.742004	1.812271	-0.285519	-0.954665	1.167429	-0.278574	-0.991395	1.185871	-0.375737	0.046235	1.960855
C	-1.311371	-1.312522	0.616506	-1.103750	-1.614851	0.031684	-1.094506	-1.659337	0.053020	-0.938868	-0.951260	0.919208
O	-1.879938	-2.505204	1.155860	-1.631987	-2.783534	0.657846	-1.609304	-2.832796	0.681214	-1.528409	-1.965183	1.732888
C	-0.398885	-1.632933	-0.577888	-0.216295	-1.989058	-1.166073	-0.207851	-2.025100	-1.147949	0.175038	-1.533525	0.034718
C	-2.412148	-0.287373	0.243529	-2.235809	-0.636522	-0.371487	-2.237576	-0.692224	-0.346127	-2.015321	-0.213793	0.084012
C	-3.322768	-0.581243	-0.966157	-3.169529	-1.019963	-1.537690	-3.172544	-1.085850	-1.507949	-2.699674	-0.965549	-1.075983
C	-4.313074	-1.745104	-0.818890	-4.130835	-2.192289	-1.296368	-4.120300	-2.268268	-1.261986	-3.637627	-2.119573	-0.693948
O	-3.565518	-2.981006	-0.966138	-3.360457	-3.419780	-1.386216	-3.337687	-3.487553	-1.357072	-2.811582	-3.256630	-0.330103
C	-5.437171	-1.717389	-1.838811	-5.278163	-2.249678	-2.288745	-5.272482	-2.336874	-2.248015	-4.583328	-2.526533	-1.809597
C	-5.049524	-1.735474	-3.297136	-4.923567	-2.348110	-3.752290	-4.924963	-2.431261	-3.713522	-3.966099	-2.961560	-3.116167
C	-6.710192	-1.705013	-1.429379	-6.541512	-2.238857	-1.850312	-6.533438	-2.338958	-1.802635	-5.904498	-2.515057	-1.602841
O	0.201169	0.462815	1.391114	0.387112	0.237833	0.653358	0.380114	0.208373	0.669372	0.348849	1.112692	1.269997
C	1.508273	0.348339	1.755107	1.724550	0.123726	0.884168	1.719347	0.107734	0.895250	1.628547	1.144489	1.729258
C	2.453185	1.357363	1.541564	2.653419	1.093205	0.489412	2.636547	1.086244	0.497768	2.595145	2.036466	1.250554
C	2.080820	2.665454	0.866832	2.222620	2.364361	-0.219646	2.188697	2.352619	-0.209478	2.287363	3.017780	0.135992
C	2.011098	2.539089	-0.638436	1.588276	3.363512	0.722143	1.551814	3.346817	0.735786	2.352166	2.376476	-1.232496
C	2.730124	3.190414	-1.564735	1.998293	4.606314	1.016227	1.945007	4.597885	1.017530	1.408828	2.344501	-2.186316
C	2.514151	2.916948	-3.035210	1.223859	5.456230	1.996882	1.170378	5.440475	2.004321	1.679297	1.668905	-3.510846
C	3.789750	4.225766	-1.275636	3.218688	5.281226	0.438967	3.145354	5.290061	0.418887	0.037717	2.964299	-2.069978
C	3.751894	1.077322	1.974899	3.984628	0.818562	0.805359	3.974241	0.828366	0.808137	3.857375	1.944713	1.843192
O	5.812544	-1.466668	3.595082	6.698739	0.227356	1.449125	6.198835	-1.610634	2.372342	5.786533	0.131359	4.360359
H	7.190527	0.261175	3.172106	7.046268	-1.717518	2.527315	7.525269	0.061183	1.658957	7.200474	1.640578	3.473078
H	3.379839	-2.057663	3.271196	3.703463	-2.213429	2.361660	3.744541	-2.201105	2.363252	3.377608	-0.578784	4.089083
H	0.617311	-2.632249	1.870343	0.828745	-2.843647	1.349559	0.856124	-2.868942	1.363487	0.716376	-1.641980	2.851501
H	0.271287	-1.967285	3.456619	0.627997	-1.994259	2.872744	0.652290	-2.022047	2.887448	0.240321	-0.418706	4.017834
H	-1.233664	-0.422767	2.573284	-0.990862	-0.606076	1.928469	-0.984426	-0.649786	1.949580	-1.223442	0.524446	2.461613
H	-2.411266	-2.907072	0.437843	-2.174289	-3.238190	-0.019370	-2.148044	-3.293839	0.005521	-1.907887	-2.626274	1.117986
H	-0.965923	-2.154801	-1.354693	-0.792171	-2.568020	-1.894366	-0.780866	-2.609309	-1.874305	-0.227935	-2.321574	-0.608808
H	0.027463	-0.722892	-1.012184	0.177876	-1.098203	-1.665595	0.176088	-1.130483	-1.648776	0.626263	-0.762194	-0.597767
H	0.423604	-2.289117	-0.277706	0.627802	-2.609252	-0.849543	0.643102	-2.637418	-0.834541	0.963312	-1.983258	0.646245
H	-3.043358	-0.152039	1.132812	-2.847409	-0.461534	0.524611	-2.847039	-0.522444	0.552421	-2.789871	0.129548	0.783966
H	-1.922039	0.676354	0.059442	-1.771336	0.324130	-0.624854	-1.783602	0.272722	-0.602275	-1.551811	0.686962	-0.335973
H	-3.923064	0.320448	-1.142735	-3.793203	-0.142589	-1.751723	-3.806362	-0.215127	-1.719376	-3.310509	-0.232360	-1.618325
H	-2.729236	-0.737388	-1.874463	-2.595143	-1.218036	-2.450136	-2.600299	-1.278242	-2.422965	-1.958385	-1.338113	-1.792539
H	-4.750063	-1.712539	0.189582	-4.545286	-2.107067	-0.281464	-4.529921	-2.187986	-0.244736	-4.231881	-1.818014	0.180648
H	-4.199358	-3.712831	-0.885128	-3.976904	-4.157752	-1.247413	-3.945398	-4.232068	-1.214693	-3.410501	-3.986397	-0.100713
H	-4.351171	-2.554484	-3.504335	-4.210711	-3.162299	-3.926429	-4.205018	-3.238271	-3.891847	-3.202332	-3.729795	-2.949313
H	-5.926564	-1.852638	-3.940245	-5.812028	-2.524047	-4.365633	-5.814977	-2.615801	-4.322070	-4.721256	-3.361622	-3.798974
H	-4.541462	-0.806488	-3.586221	-4.444696	-1.427306	-4.109397	-4.457190	-1.505626	-4.072866	-3.466569	-2.123912	-3.619727
H	-7.538752	-1.693876	-2.133327	-7.385888	-2.287669	-2.533555	-7.381034	-2.396090	-2.481236	-6.608643	-2.808780	-2.377505
H	-6.972312	-1.693890	-0.373729	-6.779766	-2.169019	-0.791260	-6.766537	-2.272040	-0.742250	-6.333030	-2.200421	-0.653842
H	1.096777	2.982323	1.239913	3.087051	2.793561	-0.733631	3.044835	2.789848	-0.730873	1.307989	3.473243	0.309603
H	2.795896	3.436377	1.167570	1.490567	2.100584	-0.996617	1.453439	2.080973	-0.980671	3.025702	3.830923	0.182751
H	1.277736	1.811520	-0.987313	0.689424	2.994972	1.216434	0.665851	2.966488	1.244147	3.304076	1.888698	-1.447362
H	1.739464	2.161658	-3.201043	0.348299	4.930604	2.390769	0.309551	4.902467	2.413555	2.686178	1.242115	-3.554582
H	2.218632	3.830918	-3.569960	1.854300	5.751921	2.847522	1.807567	5.751727	2.844342	0.958390	0.860423	-3.698234
H	3.439825	2.564484	-3.512012	0.878816	6.388856	1.528026	0.802934	6.364378	1.535219	1.573896	2.377037	-4.345159
H	3.939921	4.410702	-0.209542	3.772410	4.655384	-0.264563	3.700093	4.668940	-0.288145	-0.173707	3.364836	-1.076459
H	4.755338	3.918832	-1.701200	2.939133	6.206524	-0.084155	2.842382	6.206340	-0.107053	-0.083474	3.779503	-2.797523
H	3.534237	5.184416	-1.748516	3.911201	5.575790	1.239714	3.843322	5.603137	1.207894	-0.739938	2.224055	-2.303899
H	4.526149	1.824882	1.835625	4.762296	1.525193	0.531140	4.730028	1.553704	0.524688	4.643157	2.617455	1.514208

	**2a-5**			**2a-6**			**2a-7**			**2a-8**		
**Element**	**X**	**Y**	**Z**	**X**	**Y**	**Z**	**X**	**Y**	**Z**	**X**	**Y**	**Z**
O	5.550741	-1.102483	4.407549	6.421269	-1.540877	2.478668	6.977717	0.521218	1.504917	6.146280	1.524136	3.550786
C	5.546274	-0.088868	3.495660	6.156996	-0.376791	1.819781	6.118390	-0.458188	1.905898	5.284058	0.477635	3.671387
C	4.207771	0.179109	2.929656	4.719892	-0.181705	1.535990	4.707609	-0.175799	1.568912	4.000529	0.691175	2.965609
C	3.070069	-0.554160	3.316691	3.746441	-1.124567	1.916760	3.740664	-1.129313	1.938379	3.043822	-0.338664	3.023372
C	1.849617	-0.238256	2.743892	2.420165	-0.871020	1.609441	2.413846	-0.889782	1.629259	1.831009	-0.154189	2.383895
C	0.466350	-0.808942	2.943561	1.159175	-1.660299	1.866748	1.159561	-1.692706	1.876165	0.637719	-1.059692	2.187728
C	-0.388392	0.012424	1.940151	0.064085	-0.774936	1.211401	0.058636	-0.813831	1.222053	-0.413445	-0.091082	1.590915
C	-1.079809	-0.816568	0.830146	-0.739133	-1.450592	0.073486	-0.733043	-1.489506	0.076091	-1.227626	-0.600201	0.381637
O	-1.837849	-1.773576	1.570039	-1.263633	-2.618786	0.703920	-1.249295	-2.666490	0.696669	-0.283536	-0.881321	-0.673864
C	-0.053379	-1.511245	-0.078741	0.163149	-1.827022	-1.112545	0.178026	-1.849677	-1.108285	-2.219371	0.479457	-0.077838

C	-2.009378	0.132871	0.033525	-1.876688	-0.486740	-0.347884	-1.878039	-0.534023	-0.344235	-1.922107	-1.927249	0.749277
C	-2.777671	-0.426116	-1.181503	-2.796218	-0.888369	-1.519289	-2.789221	-0.937853	-1.521404	-3.003560	-2.492432	-0.195557
C	-3.894257	-1.438060	-0.887444	-3.749444	-2.067125	-1.277001	-3.730839	-2.128143	-1.289953	-2.523255	-2.992728	-1.578609
O	-3.266428	-2.711975	-0.586155	-2.967606	-3.288344	-1.349809	-2.936191	-3.340544	-1.369591	-2.128887	-1.923266	-2.432768
C	-4.865569	-1.620638	-2.039768	-4.887106	-2.142575	-2.279328	-4.865128	-2.208080	-2.295764	-3.619884	-3.768897	-2.290228
C	-4.293760	-2.066537	-3.363234	-4.518131	-2.249957	-3.738653	-4.491297	-2.301598	-3.754806	-4.731968	-2.961285	-2.910451
C	-6.172999	-1.412700	-1.850642	-6.154471	-2.138993	-1.852524	-6.133567	-2.220335	-1.872339	-3.583469	-5.103169	-2.347727
O	0.508938	0.991441	1.328072	0.739579	0.414255	0.694042	0.724625	0.385856	0.715497	0.342015	1.078331	1.129649
C	1.769145	0.787502	1.797362	2.073717	0.304754	0.937499	2.058660	0.287599	0.963634	1.584965	1.036032	1.696251
C	2.874929	1.540576	1.383820	3.009141	1.268800	0.541581	2.985980	1.262835	0.578438	2.512224	2.076519	1.593702
C	2.749089	2.626891	0.333236	2.585399	2.546650	-0.157792	2.551505	2.541055	-0.113858	2.216634	3.323819	0.784216
C	2.732119	2.070225	-1.073189	2.082945	3.590054	0.815219	2.033427	3.572560	0.863635	2.311821	3.072646	-0.705144
C	1.811755	2.251146	-2.032820	0.861898	4.139439	0.894857	0.806222	4.108354	0.941249	1.395808	3.319156	-1.654966
C	1.992412	1.626272	-3.396964	0.554941	5.176058	1.950437	0.482999	5.133703	2.002946	1.695370	3.018830	-3.105701
C	0.556390	3.075683	-1.884911	-0.298682	3.809548	-0.010980	-0.346407	3.772506	0.027522	0.033432	3.921300	-1.411077
C	4.092457	1.206826	1.978944	4.338889	0.989407	0.860013	4.319790	0.997015	0.899194	3.728162	1.870764	2.253078
O	6.568707	0.506579	3.208301	7.049595	0.393755	1.516460	6.519274	-1.452634	2.483031	5.577191	-0.518744	4.305346
H	6.477625	-1.177068	4.699455	7.387891	-1.548092	2.603071	7.859643	0.214395	1.784134	6.940463	1.261976	4.051540
H	3.159488	-1.345749	4.052916	4.041802	-2.029369	2.437060	4.060548	-2.029595	2.453397	3.281081	-1.247544	3.566860
H	0.401574	-1.880292	2.732459	1.175494	-2.661867	1.427025	1.187975	-2.691211	1.430342	0.876852	-1.859039	1.476143
H	0.107084	-0.668645	3.969193	0.965228	-1.791348	2.937036	0.963145	-1.832494	2.944803	0.280759	-1.524321	3.111619
H	-1.160586	0.586263	2.462101	-0.650409	-0.420782	1.961208	-0.662496	-0.471552	1.970998	-1.099051	0.266803	2.369389
H	-2.305610	-2.324963	0.909446	-1.795081	-3.083757	0.025147	-1.771529	-3.133276	0.012050	0.213899	-0.056295	-0.821016
H	-0.561951	-2.184525	-0.775574	-0.398992	-2.419740	-1.840526	-0.375363	-2.442500	-1.842858	-2.683914	0.175634	-1.018604
H	0.525797	-0.783147	-0.655793	0.552552	-0.936876	-1.617055	0.561474	-0.952568	-1.605023	-3.006630	0.634599	0.669382
H	0.642277	-2.116615	0.510514	1.011086	-2.434297	-0.781633	1.029937	-2.451444	-0.777581	-1.705485	1.433781	-0.228620
H	-2.735509	0.548900	0.745855	-2.497882	-0.310678	0.541365	-2.504321	-0.369061	0.543571	-1.142970	-2.686963	0.888805
H	-1.401445	0.974011	-0.320629	-1.419936	0.476698	-0.604673	-1.429642	0.435243	-0.593796	-2.391063	-1.780891	1.732336
H	-3.253016	0.427421	-1.681833	-3.425730	-0.018492	-1.746537	-3.427009	-0.073180	-1.745456	-3.457082	-3.346711	0.323653
H	-2.088556	-0.865727	-1.911970	-2.211698	-1.089042	-2.424678	-2.199482	-1.126784	-2.425951	-3.804547	-1.758533	-0.341470
H	-4.454183	-1.100332	-0.003445	-4.173908	-1.977193	-0.266650	-4.158729	-2.049839	-0.280090	-1.674730	-3.677037	-1.410797
H	-3.978152	-3.350263	-0.413218	-3.578549	-4.030604	-1.209539	-3.539496	-4.090172	-1.235547	-1.337021	-1.518720	-2.024418
H	-3.664072	-2.954614	-3.236021	-3.796447	-3.059104	-3.899340	-3.761243	-3.102457	-3.919142	-4.324795	-2.260738	-3.647187
H	-5.086111	-2.299414	-4.080501	-5.399227	-2.438981	-4.358695	-5.368849	-2.494967	-4.378529	-5.464034	-3.608669	-3.402960
H	-3.657358	-1.288975	-3.804964	-4.044185	-1.327895	-4.099092	-4.025505	-1.372419	-4.107536	-5.261776	-2.357251	-2.161793
H	-6.896089	-1.542791	-2.652146	-6.992117	-2.200721	-2.542965	-6.968744	-2.285777	-2.565430	-4.373255	-5.676648	-2.827110
H	-6.568978	-1.093102	-0.889239	-6.403010	-2.062588	-0.796290	-6.385612	-2.153940	-0.816261	-2.762257	-5.674149	-1.920182
H	1.853779	3.224090	0.529685	3.455513	2.948644	-0.694847	3.419310	2.956359	-0.644880	1.229021	3.710703	1.053802
H	3.608954	3.303589	0.437186	1.827590	2.316564	-0.912530	1.799600	2.308161	-0.873601	2.945543	4.096465	1.066279
H	3.597094	1.450338	-1.313350	2.829329	3.909911	1.543715	2.772525	3.894915	1.598518	3.263383	2.642493	-1.020753
H	2.918197	1.046159	-3.461990	1.423985	5.387556	2.581265	1.346696	5.350135	2.639447	2.692352	2.587075	-3.237538
H	1.154115	0.958518	-3.641724	0.227234	6.121320	1.494725	0.146807	6.078594	1.552689	0.960953	2.318001	-3.527061
H	2.016550	2.393770	-4.183737	-0.266233	4.845142	2.602147	-0.337345	4.788811	2.648366	1.636662	3.930463	-3.716839
H	-0.326131	2.487615	-2.172714	-0.069206	3.031764	-0.741908	-0.105241	3.002299	-0.707625	-0.196340	4.063276	-0.352852
H	0.395033	3.440307	-0.868426	-1.159062	3.466326	0.579953	-1.205727	3.415917	0.611980	-0.050776	4.898176	-1.907583
H	0.580585	3.944924	-2.557523	-0.632363	4.703597	-0.556529	-0.687098	4.666779	-0.513297	-0.752881	3.288098	-1.844391
H	4.992335	1.752917	1.710815	5.120160	1.689881	0.579283	5.081875	1.717427	0.619049	4.483705	2.649093	2.218460

	**2a-9**			**2a-10**			**2a-11**			**2a-12**		
**Element**	**X**	**Y**	**Z**	**X**	**Y**	**Z**	**X**	**Y**	**Z**	**X**	**Y**	**Z**
O	5.093200	-0.784031	4.181741	4.757667	-0.325412	5.109927	5.818723	-2.185069	2.557196	6.451525	-0.006865	1.990013
C	4.908970	0.424632	3.582107	4.825366	0.727236	4.248568	5.599530	-0.915624	2.110741	5.564920	-1.030304	2.146910
C	3.602356	0.538893	2.894942	3.633497	0.843196	3.378292	4.187461	-0.658201	1.758062	4.182767	-0.674387	1.763087
C	2.669706	-0.515398	2.884631	2.561777	-0.065472	3.454411	3.191470	-1.644674	1.889184	3.190613	-1.664530	1.892423
C	1.464970	-0.322011	2.230115	1.476638	0.122683	2.614448	1.889424	-1.323454	1.544452	1.888110	-1.354168	1.545920
C	0.286326	-1.234775	1.982559	0.220605	-0.685168	2.385234	0.613336	-2.130996	1.570724	0.616527	-2.168685	1.568861
C	-0.775345	-0.248153	1.438099	-0.616857	0.259722	1.487730	-0.444836	-1.108491	1.071732	-0.446959	-1.150255	1.073109
C	-1.626932	-0.728857	0.245032	-1.315186	-0.373898	0.264859	-1.276911	-1.541954	-0.160448	-1.283226	-1.586808	-0.155007
O	-0.716783	-1.043158	-0.831807	-0.277573	-0.925120	-0.574137	-1.948962	-2.748066	0.200381	-1.949546	-2.794978	0.209155
C	-2.594748	0.379124	-0.196553	-2.098607	0.697876	-0.508316	-0.390620	-1.894285	-1.360023	-0.401388	-1.936622	-1.358596
C	-2.349515	-2.035730	0.631891	-2.207452	-1.543128	0.728866	-2.262258	-0.425093	-0.603485	-2.274401	-0.473368	-0.593784
C	-3.469285	-2.572510	-0.284372	-3.210810	-2.160003	-0.268320	-3.194192	0.242124	0.430615	-3.204231	0.190591	0.444310
C	-3.037819	-3.093260	-1.675846	-2.610935	-2.939834	-1.462429	-4.414720	-0.548285	0.942042	-4.419336	-0.604359	0.961679
O	-2.633198	-2.039974	-2.545007	-1.978952	-2.080374	-2.406080	-3.929794	-1.639273	1.744489	-3.926421	-1.692756	1.762641
C	-4.173988	-3.839853	-2.356502	-3.686106	-3.721358	-2.200462	-5.370612	-0.997917	-0.153828	-5.378379	-1.058425	-0.129618
C	-5.275750	-3.002436	-2.955186	-4.598624	-2.933677	-3.106264	-6.345185	0.050895	-0.631110	-6.360187	-0.014153	-0.602030
C	-4.178899	-5.174919	-2.406380	-3.802717	-5.042131	-2.036501	-5.351173	-2.238805	-0.652632	-5.355475	-2.299132	-0.628717
O	-0.023416	0.929349	0.993031	0.328171	1.252615	0.967548	0.276230	0.131650	0.769293	0.268941	0.092379	0.766342
C	1.207127	0.894533	1.594525	1.469641	1.191626	1.716798	1.592263	-0.041633	1.072293	1.585122	-0.073721	1.072148
C	2.103770	1.965719	1.576175	2.520821	2.104990	1.591969	2.550189	0.968837	0.929178	2.537677	0.941098	0.930323
C	1.782054	3.268512	0.866456	2.489383	3.213874	0.558798	2.182199	2.345631	0.405850	2.161839	2.315186	0.405240
C	1.988408	3.178740	-0.629169	2.752199	2.697192	-0.839156	2.024140	2.368172	-1.098022	2.014992	2.337604	-1.099717
C	2.846457	3.868893	-1.395081	2.008009	2.859968	-1.944458	2.725871	3.071193	-1.999488	2.706529	3.058553	-1.994874
C	2.904943	3.631492	-2.886135	2.461559	2.283623	-3.266092	2.417670	2.950648	-3.473990	2.411658	2.934304	-3.471796

C	3.811344	4.915973	-0.894807	0.707413	3.623065	-2.010202	3.853216	4.021582	-1.677029	3.808578	4.034937	-1.662453
C	3.311204	1.748426	2.244832	3.600681	1.899794	2.453547	3.854338	0.621302	1.283992	3.845820	0.603723	1.287194
O	5.758034	1.294502	3.630081	5.786888	1.472301	4.229004	6.508208	-0.109531	2.028029	5.921704	-2.116378	2.566117
H	5.976656	-0.736169	4.590915	5.588908	-0.295585	5.618202	6.772946	-2.224502	2.751615	7.310452	-0.370118	2.273224
H	2.900358	-1.450514	3.383112	2.596805	-0.889571	4.158577	3.450706	-2.631539	2.257251	3.472236	-2.644769	2.263729
H	0.532503	-1.991255	1.227395	0.455154	-1.616149	1.855246	0.654769	-3.016387	0.929229	0.664338	-3.051361	0.924161
H	-0.065194	-1.752174	2.880087	-0.311245	-0.944185	3.305643	0.369601	-2.485976	2.578071	0.374650	-2.529011	2.574596
H	-1.437584	0.091387	2.245026	-1.353670	0.811718	2.084897	-1.137430	-0.864551	1.883247	-1.136565	-0.907533	1.887586
H	-0.204425	-0.233185	-1.002313	0.329998	-0.189877	-0.774367	-2.630631	-2.507074	0.862107	-2.628201	-2.556430	0.874841
H	-3.091789	0.085725	-1.123852	-2.464700	0.279166	-1.448429	-1.022041	-2.150400	-2.216823	-1.035955	-2.195340	-2.212282
H	-3.357473	0.563516	0.569094	-2.955327	1.056482	0.074448	0.253992	-1.055227	-1.637616	0.238949	-1.095400	-1.639599
H	-2.055228	1.316257	-0.365266	-1.458109	1.557260	-0.729053	0.236243	-2.763744	-1.143946	0.229516	-2.803741	-1.145057
H	-1.587422	-2.815721	0.754359	-1.551418	-2.337335	1.106507	-1.657575	0.378810	-1.038755	-1.674443	0.332642	-1.031740
H	-2.790182	-1.874920	1.625593	-2.784332	-1.181916	1.591714	-2.866688	-0.841034	-1.418082	-2.880941	-0.891471	-1.405699
H	-3.935484	-3.410458	0.249973	-3.830149	-2.862037	0.304950	-2.628184	0.581756	1.305722	-2.635561	0.532674	1.316746
H	-4.251236	-1.815541	-0.414790	-3.890301	-1.390131	-0.651779	-3.590999	1.155529	-0.030683	-3.606714	1.102272	-0.015516
H	-2.206045	-3.801744	-1.525029	-1.881370	-3.661529	-1.058189	-4.970067	0.133519	1.606916	-4.974400	0.075718	1.628577
H	-1.820979	-1.656196	-2.157910	-1.207191	-1.687045	-1.950946	-4.688042	-2.211369	1.948351	-4.681276	-2.267992	1.970230
H	-4.865335	-2.317954	-3.705073	-4.014072	-2.416214	-3.874283	-5.823570	0.925661	-1.042130	-5.844693	0.863302	-1.015066
H	-6.038068	-3.630105	-3.426847	-5.329495	-3.585071	-3.595237	-7.002354	-0.342258	-1.411998	-7.019009	-0.410175	-1.380077
H	-5.769662	-2.379173	-2.197811	-5.148672	-2.159278	-2.555097	-6.972053	0.419236	0.192386	-6.985087	0.350832	0.224457
H	-4.996683	-5.727066	-2.863304	-4.583227	-5.613361	-2.533431	-6.052463	-2.545218	-1.424410	-6.059070	-2.608729	-1.397126
H	-3.365112	-5.767926	-1.994891	-3.121596	-5.604266	-1.401444	-4.615690	-2.974302	-0.342466	-4.615008	-3.031184	-0.322291
H	0.731227	3.522996	1.063608	1.528636	3.735559	0.609593	1.230964	2.650692	0.864459	1.204463	2.611307	0.856852
H	2.387863	4.067288	1.302513	3.262316	3.949189	0.822057	2.937481	3.063814	0.736743	2.907747	3.040469	0.742383
H	1.348395	2.446661	-1.123187	3.679354	2.130153	-0.933366	1.236600	1.712235	-1.469978	1.245136	1.664917	-1.478631
H	2.189916	2.867999	-3.208408	3.408107	1.742073	-3.173809	1.596079	2.252909	-3.664442	1.608058	2.217936	-3.669447
H	2.690270	4.554649	-3.442607	1.711628	1.593681	-3.678074	2.143559	3.924909	-3.903276	2.117664	3.902820	-3.900930
H	3.909223	3.309270	-3.195225	2.595600	3.075963	-4.015920	3.296748	2.601532	-4.034076	3.302477	2.607123	-4.026594
H	3.762144	5.076889	0.184468	0.356264	3.968911	-1.035575	4.067324	4.098744	-0.608694	4.014876	4.112686	-0.592538
H	4.844436	4.637143	-1.143972	0.808648	4.501873	-2.662387	4.777937	3.707483	-2.180719	4.743316	3.746507	-2.163172
H	3.623796	5.880691	-1.386286	-0.083866	3.002327	-2.452500	3.626839	5.031358	-2.047185	3.559168	5.040592	-2.028999
H	4.057913	2.535950	2.281249	4.450829	2.575010	2.424475	4.650873	1.354772	1.201045	4.625812	1.353032	1.198507

	**2a-13**			**2a-14**			**2a-15**			**2a-16**		
**Element**	**X**	**Y**	**Z**	**X**	**Y**	**Z**	**X**	**Y**	**Z**	**X**	**Y**	**Z**
O	6.220480	1.136304	2.458328	6.155980	0.729495	3.597384	5.976211	-0.810463	3.301733	4.862623	-1.013597	3.802070
C	5.381107	0.074618	2.603521	5.288507	-0.313141	3.482006	5.740926	0.390765	2.704990	3.827209	-1.874465	3.604094
C	4.068199	0.270697	1.947775	4.079444	0.014574	2.693113	4.395976	0.481593	2.092874	2.681324	-1.260439	2.896116
C	3.134549	-0.778899	2.029338	3.125444	-1.002247	2.506220	3.483319	-0.588630	2.132115	1.562201	-2.072412	2.638796
C	1.898771	-0.614082	1.430258	1.985719	-0.715983	1.776601	2.243416	-0.419395	1.538939	0.479843	-1.521092	1.976239
C	0.713080	-1.537383	1.272506	0.820508	-1.571474	1.337953	1.078963	-1.361618	1.342118	-0.845977	-2.109708	1.552101
C	-0.376042	-0.575241	0.738780	-0.170850	-0.512056	0.798683	-0.030193	-0.402283	0.846416	-1.542277	-0.912684	0.848969
C	-1.274497	-1.100201	-0.400349	-0.913939	-0.855321	-0.509032	-0.917481	-0.903353	-0.311977	-1.940603	-1.076000	-0.635614
O	-0.408736	-1.440739	-1.505385	0.088571	-1.071595	-1.526378	-0.045442	-1.177908	-1.430454	-2.534640	0.188497	-1.017052
C	-2.270167	-0.013913	-0.833818	-1.831557	0.305965	-0.919433	-1.941703	0.173052	-0.700896	-3.061121	-2.109534	-0.759345
C	-1.968259	-2.399454	0.057946	-1.677301	-2.183317	-0.326108	-1.578131	-2.236907	0.094121	-0.715802	-1.385709	-1.528096
C	-3.121552	-2.973340	-0.791831	-2.712321	-2.605083	-1.390236	-2.718126	-2.804265	-0.777675	-0.951982	-1.771237	-3.003919
C	-2.745727	-3.534038	-2.183941	-2.158934	-2.984663	-2.784249	-2.330301	-3.297527	-2.191856	-1.520794	-0.674798	-3.935891
O	-2.388588	-2.505258	-3.102008	-1.672334	-1.852575	-3.498892	-2.002217	-2.223170	-3.067612	-2.894805	-0.403184	-3.680651
C	-3.903406	-4.310294	-2.791200	-3.231917	-3.640841	-3.638407	-3.467918	-4.079083	-2.829415	-1.398487	-1.081095	-5.396013
C	-5.036827	-3.500430	-3.368291	-4.270668	-2.730680	-4.243544	-4.623867	-3.277195	-3.371926	-2.382545	-2.106311	-5.900013
C	-3.899019	-5.646270	-2.798301	-3.238571	-4.963427	-3.827190	-3.427579	-5.413042	-2.891466	-0.455206	-0.546586	-6.176596
O	0.349044	0.590234	0.221123	0.635872	0.686043	0.543177	0.670145	0.796807	0.375574	-0.617684	0.222880	0.941082
C	1.608443	0.576741	0.760471	1.805070	0.563081	1.244007	1.925792	0.791991	0.920544	0.519303	-0.182428	1.580400
C	2.506408	1.639734	0.643239	2.728725	1.600517	1.396613	2.804208	1.877355	0.850516	1.609997	0.660642	1.811845
C	2.139115	2.921128	-0.083686	2.479526	2.985887	0.830239	2.409557	3.189102	0.198289	1.600688	2.114284	1.374811
C	1.232006	3.809767	0.738210	1.567489	3.808391	1.713262	1.580117	4.056871	1.118664	0.763897	2.984973	2.285855
C	1.461990	5.057720	1.172813	0.392792	4.376217	1.401873	0.345306	4.542119	0.921030	1.152974	4.044421	3.010718
C	0.422272	5.786730	1.991911	-0.370833	5.172316	2.434363	-0.315437	5.405544	1.970256	0.158943	4.790103	3.870482
C	2.717695	5.853853	0.913335	-0.277392	4.301990	0.052251	-0.494046	4.306401	-0.310280	2.554945	4.601330	3.058126
C	3.747560	1.452476	1.260525	3.873528	1.287632	2.137249	4.048554	1.683363	1.455388	2.690414	0.082417	2.484361
O	5.714342	-0.920752	3.218877	5.520146	-1.393425	3.991759	6.578649	1.272868	2.698458	3.876573	-3.028705	3.986156
H	7.036885	0.884612	2.927751	6.898110	0.388951	4.129823	6.880660	-0.747070	3.659730	5.542319	-1.531906	4.270695
H	3.407982	-1.687144	2.556566	3.309391	-1.982236	2.934592	3.757805	-1.520199	2.614864	1.577319	-3.107576	2.964429
H	0.928658	-2.322620	0.537739	1.124045	-2.259043	0.539129	1.311987	-2.111366	0.576142	-0.723343	-2.968639	0.882772
H	0.404949	-2.019875	2.204662	0.380876	-2.162969	2.146351	0.780462	-1.888386	2.253317	-1.435872	-2.456774	2.407567
H	-1.005596	-0.209949	1.560327	-0.900344	-0.236308	1.570977	-0.666769	-0.082716	1.681481	-2.442645	-0.601569	1.387294
H	0.085404	-0.631040	-1.724368	0.623055	-0.258591	-1.559241	0.434850	-0.350537	-1.610885	-1.884019	0.878242	-0.797764
H	-2.804014	-0.340094	-1.729147	-2.243279	0.116161	-1.913165	-2.468159	-0.130687	-1.608441	-3.481918	-2.074298	-1.766964
H	-3.000905	0.188756	-0.042044	-2.660153	0.421199	-0.210749	-2.676267	0.323606	0.098995	-2.689401	-3.122473	-0.572884
H	-1.746664	0.921450	-1.055024	-1.274527	1.247722	-0.940406	-1.443602	1.130207	-0.883481	-3.863261	-1.892603	-0.045933
H	-1.194873	-3.169496	0.171752	-0.934904	-2.983938	-0.217374	-0.785936	-2.991732	0.176012	-0.046745	-0.515497	-1.487219
H	-2.367174	-2.210642	1.064289	-2.205457	-2.118434	0.635327	-1.979930	-2.098752	1.107536	-0.156251	-2.210135	-1.068054

H	-3.556427	-3.797799	-0.212052	-3.229463	-3.487113	-0.990744	-3.131176	-3.662623	-0.232112	0.024080	-2.070388	-3.407457
H	-3.915529	-2.227139	-0.911592	-3.475299	-1.827274	-1.510135	-3.530776	-2.074024	-0.866106	-1.596506	-2.655953	-3.067826
H	-1.901834	-4.230982	-2.047634	-1.347379	-3.716277	-2.634228	-1.467871	-3.976616	-2.084502	-0.916834	0.237169	-3.786799
H	-1.563400	-2.104034	-2.763046	-0.894933	-1.524296	-3.004194	-1.186432	-1.815151	-2.714242	-2.940371	-0.017846	-2.782362
H	-4.664708	-2.837880	-4.156867	-3.790581	-1.979452	-4.879558	-4.271424	-2.573934	-4.133855	-3.407413	-1.745913	-5.762185
H	-5.813520	-4.148340	-3.786052	-4.990747	-3.295750	-4.843339	-5.384056	-3.928300	-3.814348	-2.221237	-2.321973	-6.960684
H	-5.502777	-2.856245	-2.610704	-4.827444	-2.180648	-3.473166	-5.104970	-2.676530	-2.588503	-2.304106	-3.050937	-5.345434
H	-4.730859	-6.218693	-3.201779	-4.014063	-5.453868	-4.410717	-4.244485	-5.990659	-3.317422	-0.335291	-0.845612	-7.215155
H	-3.063388	-6.219873	-2.403444	-2.468416	-5.607180	-3.408083	-2.575960	-5.979609	-2.521366	0.232813	0.211610	-5.809101
H	3.057451	3.442410	-0.367301	3.448281	3.496765	0.739615	3.329580	3.726687	-0.069716	2.631847	2.473161	1.317344
H	1.624091	2.660594	-1.019503	2.074405	2.902706	-0.182527	1.879077	2.990522	-0.737621	1.191275	2.171924	0.355688
H	0.280135	3.348100	1.000694	1.936672	3.936267	2.731743	2.074935	4.300922	2.059636	-0.282589	2.685852	2.346231
H	-0.471080	5.176128	2.156040	0.150902	5.203039	3.395930	0.327598	5.553117	2.843445	-0.843542	4.355542	3.807667
H	0.823517	6.074057	2.974073	-0.528191	6.207256	2.099337	-0.571332	6.394734	1.565125	0.467138	4.784253	4.925523
H	0.111666	6.718801	1.499031	-1.369952	4.745840	2.602238	-1.258950	4.957892	2.313432	0.091605	5.846036	3.572852
H	3.464220	5.314684	0.325629	0.270716	3.696438	-0.672656	-0.015521	3.654614	-1.044320	3.266278	4.050969	2.438003
H	2.481932	6.785477	0.380414	-1.286736	3.878410	0.147291	-1.457342	3.853473	-0.037840	2.565896	5.649573	2.728683
H	3.188408	6.150395	1.860905	-0.402468	5.307041	-0.374238	-0.729079	5.257976	-0.807204	2.936228	4.597233	4.088645
H	4.485209	2.246699	1.210517	4.626078	2.054594	2.290343	4.784398	2.482152	1.443769	3.565529	0.688962	2.693403

	2a-17			2a-18			2a-19		
**Element**	**X**	**Y**	**Z**	**X**	**Y**	**Z**	**X**	**Y**	**Z**
O	5.448147	-1.989686	2.375749	6.506666	-1.954455	1.526556	7.037157	0.145083	0.644499
C	5.209569	-0.754845	1.853225	6.220859	-0.762265	0.930025	6.189023	-0.850677	1.028516
C	3.769381	-0.478111	1.651468	4.773961	-0.549927	0.717023	4.767812	-0.547455	0.760338
C	2.776031	-1.421328	1.971452	3.812482	-1.504554	1.098737	3.811100	-1.515924	1.117120
C	1.450502	-1.085258	1.749597	2.475678	-1.233219	0.860321	2.474612	-1.257215	0.872162
C	0.169357	-1.854624	1.976176	1.223099	-2.026731	1.145337	1.227533	-2.067510	1.132209
C	-0.918151	-0.858895	1.488073	0.107849	-1.125718	0.547660	0.108468	-1.166367	0.541978
C	-1.838843	-1.302466	0.328370	-0.716639	-1.761036	-0.601359	-0.704221	-1.789902	-0.621726
O	-2.717702	-0.178227	0.081846	-1.307692	-2.949596	-0.077459	-1.287469	-2.991981	-0.120702
C	-2.748566	-2.439264	0.796556	0.172168	-2.210149	-1.766473	0.194813	-2.212028	-1.789079
C	-1.032346	-1.644248	-0.946531	-1.775898	-0.771137	-1.159404	-1.769465	-0.800546	-1.169360
C	-1.768825	-2.290888	-2.138960	-2.745593	-0.049173	-0.199286	-2.750858	-0.103159	-0.202972
C	-2.817344	-1.422255	-2.874060	-3.909408	-0.850508	0.417129	-3.910015	-0.925094	0.394870
O	-4.003637	-1.238087	-2.108722	-3.350601	-1.797562	1.344536	-3.346755	-1.880957	1.310341
C	-3.220046	-2.054367	-4.197034	-4.833435	-1.496245	-0.605588	-4.822722	-1.563520	-0.642522
C	-4.156648	-3.234014	-4.128475	-5.881175	-0.584306	-1.195682	-5.876331	-0.652469	-1.223494
C	-2.752230	-1.575058	-5.352970	-4.726531	-2.784030	-0.950826	-4.701759	-2.844631	-1.007365
O	-0.213470	0.354213	1.060148	0.763938	0.096100	0.072940	0.757218	0.068513	0.090179
C	1.130007	0.166203	1.220576	2.106482	-0.028179	0.254949	2.099086	-0.045815	0.282391
C	2.084215	1.132305	0.885944	3.030027	0.948047	-0.139489	3.016344	0.944822	-0.087880
C	1.687501	2.486288	0.326279	2.584168	2.255909	-0.765749	2.562250	2.257750	-0.697627
C	1.148446	3.415005	1.391800	2.148338	3.266966	0.271470	2.104501	3.247615	0.350522
C	1.631908	4.605100	1.778042	0.940654	3.826291	0.438063	0.889339	3.791932	0.512394
C	0.948087	5.389431	2.873744	0.705800	4.829223	1.543520	0.631605	4.773880	1.631493
C	2.852209	5.279837	1.200720	-0.271642	3.541555	-0.413893	-0.310258	3.508933	-0.357881
C	3.412400	0.771837	1.119984	4.370690	0.650475	0.108898	4.360207	0.659237	0.167058
O	6.117217	0.012067	1.592885	7.103659	0.017599	0.621966	6.606507	-1.873948	1.539647
H	6.417389	-2.050501	2.459929	7.477713	-1.970999	1.608359	7.927665	-0.178027	0.873639
H	3.054572	-2.386489	2.380326	4.124943	-2.431966	1.566608	4.146231	-2.442442	1.572460
H	0.147023	-2.797527	1.418069	1.222487	-3.020714	0.688941	1.241154	-3.052823	0.657736
H	0.020532	-2.107954	3.031690	1.071884	-2.175155	2.220854	1.069488	-2.237384	2.203439
H	-1.569897	-0.545204	2.309139	-0.584552	-0.809584	1.333058	-0.590866	-0.868012	1.328205
H	-2.145128	0.594532	-0.067094	-1.995548	-2.669436	0.562223	-1.980073	-2.728585	0.520935
H	-3.531818	-2.610489	0.054402	-0.453628	-2.637719	-2.556358	-0.422849	-2.632642	-2.589037
H	-2.185837	-3.369199	0.929216	0.736436	-1.370479	-2.182261	0.753321	-1.360530	-2.188307
H	-3.228145	-2.180028	1.746430	0.875983	-2.984811	-1.449802	0.904179	-2.985084	-1.481070
H	-0.540805	-0.722283	-1.285457	-1.227382	0.013408	-1.693271	-1.225382	-0.002422	-1.687466
H	-0.219395	-2.325829	-0.665385	-2.355847	-1.317779	-1.912226	-2.340397	-1.340092	-1.934100
H	-0.997270	-2.562713	-2.870976	-2.199921	0.427150	0.623600	-2.213864	0.365193	0.630152
H	-2.244529	-3.228996	-1.829246	-3.203828	0.775661	-0.759930	-3.214223	0.726030	-0.752912
H	-2.349015	-0.447060	-3.093680	-4.509186	-0.130222	0.997454	-4.519601	-0.219615	0.983145
H	-3.749694	-0.712404	-1.323466	-4.066277	-2.394200	1.619570	-4.057944	-2.488542	1.572757
H	-5.086193	-2.949546	-3.624233	-5.423879	0.268544	-1.715154	-5.424778	0.212841	-1.727164
H	-4.395154	-3.608765	-5.128590	-6.510348	-1.114548	-1.916236	-6.496799	-1.177294	-1.955476
H	-3.725302	-4.061499	-3.549454	-6.530896	-0.163591	-0.416167	-6.533932	-0.250371	-0.440761
H	-3.007296	-2.031553	-6.306362	-5.406148	-3.230634	-1.671944	-5.373576	-3.286285	-1.738773
H	-2.097722	-0.707099	-5.392091	-3.939077	-3.422335	-0.562756	-3.910142	-3.481461	-0.625410
H	2.549494	2.923444	-0.184507	3.428554	2.669988	-1.333790	3.407389	2.690393	-1.250900
H	0.911602	2.336632	-0.438676	1.784127	2.062543	-1.486298	1.771818	2.067264	-1.429442
H	0.258531	3.040439	1.897839	2.937610	3.552675	0.968273	2.882598	3.529645	1.061343
H	0.069570	4.866536	3.264435	1.611479	5.010736	2.130607	1.528684	4.955108	2.231755
H	1.634019	5.576359	3.711982	0.364090	5.792678	1.139018	0.284043	5.740254	1.239133

H	0.624490	6.375584	2.511940	-0.081070	4.484598	2.229638	-0.159031	4.409589	2.302911
H	3.341402	4.701949	0.413321	-0.099450	2.769608	-1.166276	-0.121980	2.750548	-1.120116
H	2.589467	6.260980	0.781430	-1.110614	3.211252	0.214346	-1.152938	3.160669	0.255449
H	3.595774	5.468586	1.987283	-0.608627	4.452733	-0.928328	-0.650351	4.424800	-0.861833
H	4.210660	1.469791	0.886073	5.143073	1.359223	-0.175687	5.114477	1.390724	-0.105488

Table S2.6. Calculated conformational analysis of the **2b** at B3LYP/6-31g(d) level

conformer	3D conformer	G (Hartree)	ΔG (kcal/mol)	Population
2b-1	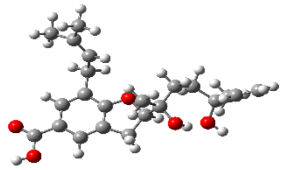	-1232.090091	0	23.822%
2b-2	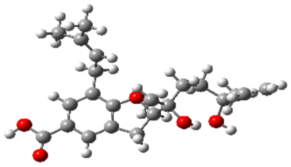	-1232.090022	0.04329819	22.143%
2b-3	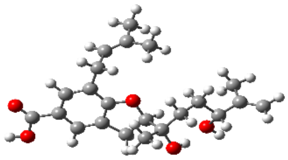	-1232.089058	0.64821783	7.976%
2b-4	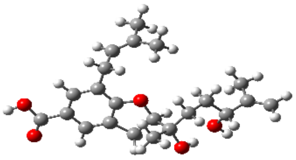	-1232.088994	0.68837847	7.453%
2b-5	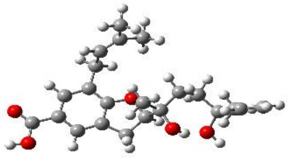	-1232.088824	0.79505517	6.225%
2b-6	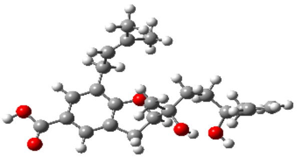	-1232.088783	0.82078308	5.960%
2b-7	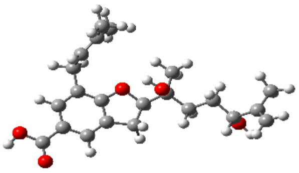	-1232.088275	1.13955816	3.480%
2b-8	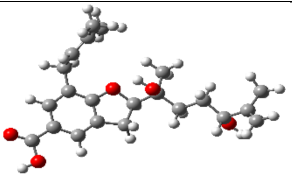	-1232.088255	1.15210836	3.407%

2b-9	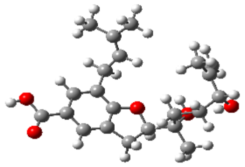	-1232.08803	1.29329811	2.685%
2b-10	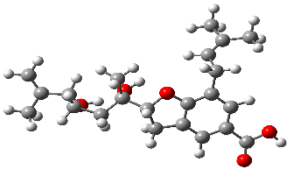	-1232.087809	1.43197782	2.124%
2b-11	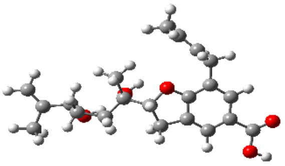	-1232.0878	1.43762541	2.104%
2b-12	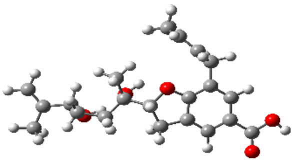	-1232.08779	1.44390051	2.082%
2b-13	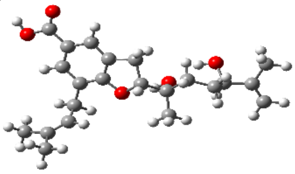	-1232.0875	1.62587841	1.081%
2b-14	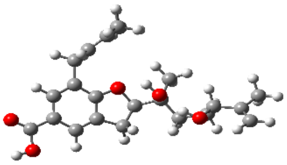	-1232.087171	1.8323292	1.065%
2b-15	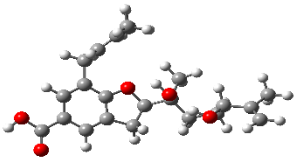	-1232.087157	1.84111434	1.058%
2b-16	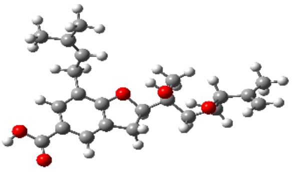	-1232.087151	1.8448794	1.081%

Table S2.7. Cartesian coordinates of low-energy conformers of **2b**

	**2b-1**			**2b-2**			**2b-3**			**2b-4**		
**Element**	**X**	**Y**	**Z**	**X**	**Y**	**Z**	**X**	**Y**	**Z**	**X**	**Y**	**Z**
O	4.330044	-0.874118	4.940349	5.676100	-1.415288	3.116595	4.707779	-2.005391	4.696021	5.836302	-2.777327	2.764720
C	4.597333	-1.048278	3.614804	4.526970	-0.992284	3.715403	4.830935	-2.266378	3.363536	4.803121	-2.198312	3.439591
C	3.458210	-0.698349	2.740384	3.445772	-0.679946	2.757344	3.669747	-1.810325	2.571223	3.686927	-1.786191	2.563166
C	2.235990	-0.226844	3.256260	2.217661	-0.230494	3.278092	2.560946	-1.176955	3.163517	2.567927	-1.181652	3.165544
C	1.218301	0.085918	2.370956	1.192098	0.074180	2.401605	1.509302	-0.783601	2.353050	1.508329	-0.791251	2.366801
C	-0.179479	0.613930	2.591043	-0.213469	0.577416	2.629377	0.204228	-0.091402	2.665677	0.192123	-0.127115	2.692720
C	-0.733234	0.721676	1.143313	-0.768843	0.698419	1.183377	-0.492038	-0.038800	1.278204	-0.505582	-0.059364	1.306675
C	-2.028669	-0.078454	0.857534	-2.061040	-0.103235	0.888374	-1.836977	-0.803999	1.181241	-1.839494	-0.841715	1.195873
O	-3.028878	0.396681	1.756770	-3.062861	0.356983	1.793384	-2.715978	-0.247668	2.157475	-2.725447	-0.317419	2.183371
C	-1.864030	-1.571194	1.162028	-1.890428	-1.598871	1.174742	-1.684426	-2.284068	1.547931	-1.665210	-2.326280	1.533558
C	-2.470430	0.066793	-0.622965	-2.503926	0.058192	-0.590173	-2.438580	-0.718952	-0.246232	-2.444165	-0.737320	-0.229034
C	-2.618464	1.468682	-1.251450	-2.658456	1.467058	-1.201254	-2.625531	0.654514	-0.925150	-2.653327	0.646543	-0.879795
C	-3.760943	2.350002	-0.729750	-3.803835	2.337296	-0.667319	-3.675532	1.595756	-0.320089	-3.715400	1.559936	-0.253320
O	-3.360836	2.862772	0.569055	-3.403531	2.836511	0.636594	-3.106466	2.167278	0.887980	-3.150494	2.117057	0.963317
C	-4.097221	3.513873	-1.644282	-4.146638	3.510306	-1.567667	-4.095954	2.716863	-1.253001	-4.156258	2.691915	-1.163397
C	-2.987666	4.484403	-1.967223	-3.041929	4.489636	-1.880510	-3.013197	3.634766	-1.765290	-3.089543	3.636198	-1.661203
C	-5.343291	3.660227	-2.107220	-5.393954	3.656456	-2.027290	-5.386017	2.874296	-1.568197	-5.449608	2.835213	-1.471768
O	0.334325	0.265523	0.247886	0.300214	0.256644	0.281770	0.460401	-0.588216	0.308829	0.454171	-0.577636	0.326763
C	1.418750	-0.076141	0.996929	1.391476	-0.074423	1.025346	1.566598	-1.015873	0.975488	1.567870	-0.998643	0.984851
C	2.618037	-0.537280	0.441797	2.595444	-0.513373	0.464063	2.650148	-1.641475	0.346208	2.660352	-1.595163	0.344252
C	2.789821	-0.701540	-1.057673	2.763987	-0.663172	-1.037349	2.685069	-1.846291	-1.156564	2.695475	-1.773100	-1.162027
C	2.145625	-1.966941	-1.578005	2.146773	-1.939513	-1.563506	3.140914	-0.605662	-1.892165	3.127572	-0.512384	-1.877518
C	2.730078	-3.011323	-2.183818	2.748545	-2.958467	-2.195113	2.464667	0.133193	-2.784307	2.436516	0.228991	-2.756119
C	1.907284	-4.197935	-2.629451	1.951270	-4.161742	-2.642427	3.104969	1.346153	-3.418179	3.053340	1.464247	-3.369871
C	4.204610	-3.131569	-2.482815	4.219757	-3.032097	-2.524449	1.054471	-0.140764	-3.245578	1.030770	-0.062808	-3.219948
C	3.628520	-0.844755	1.353899	3.616345	-0.814319	1.369419	3.696087	-2.030795	1.184090	3.716820	-1.983364	1.172311
O	5.681288	-1.453993	3.237043	4.444083	-0.896011	4.926366	5.818147	-2.821841	2.917183	4.839527	-2.054470	4.648137
H	5.147983	-1.136481	5.400829	6.295993	-1.585134	3.849265	5.525425	-2.350394	5.098928	6.494061	-2.998394	3.449121
H	2.107677	-0.111601	4.327158	2.108023	-0.131124	4.353336	2.540768	-1.008803	4.234935	2.563228	-1.039999	4.241643
H	-0.795673	-0.044239	3.210853	-0.817950	-0.100276	3.239549	-0.407534	-0.623137	3.400230	-0.409604	-0.683389	3.417217
H	-0.175740	1.593484	3.082257	-0.225100	1.548946	3.135770	0.364772	0.918211	3.061640	0.336239	0.876921	3.108377
H	-0.913701	1.769604	0.887303	-0.953208	1.748605	0.939390	-0.662999	0.998063	0.976832	-0.691108	0.980278	1.024046
H	-3.188895	1.337986	1.536249	-3.225613	1.300644	1.585379	-2.865733	0.688370	1.909017	-2.887143	0.621686	1.954882
H	-2.794554	-2.095907	0.922643	-2.818534	-2.124570	0.928253	-2.656465	-2.780642	1.464066	-2.630352	-2.834818	1.442072
H	-1.052821	-2.009762	0.573864	-1.076911	-2.026684	0.581818	-0.975659	-2.786480	0.883582	-0.950947	-2.805657	0.858145
H	-1.659204	-1.736450	2.223312	-1.685699	-1.776169	2.234066	-1.343957	-2.401879	2.580333	-1.320728	-2.459144	2.562772
H	-1.747401	-0.480746	-1.238832	-1.778769	-0.478611	-1.212996	-1.801566	-1.315779	-0.909533	-1.799028	-1.310803	-0.905009
H	-3.427421	-0.463924	-0.715595	-3.458661	-0.475468	-0.688919	-3.411475	-1.226742	-0.205130	-3.409093	-1.260720	-0.197098
H	-1.678431	2.028773	-1.185934	-1.720672	2.030207	-1.129887	-1.670772	1.188285	-0.998549	-1.706815	1.195917	-0.944681
H	-2.805899	1.316501	-2.322116	-2.846578	1.327277	-2.273498	-2.941628	0.455784	-1.957242	-2.969254	0.463856	-1.914913
H	-4.659312	1.728605	-0.603756	-4.699319	1.710616	-0.547118	-4.564935	1.008652	-0.049489	-4.594732	0.954272	0.009113
H	-4.080490	3.430990	0.890328	-4.125052	3.397591	0.966133	-3.768196	2.772838	1.261386	-3.819994	2.704924	1.350895
H	-2.495540	4.832560	-1.051773	-2.550446	4.829442	-0.961587	-2.405448	4.020489	-0.938652	-2.485413	4.015722	-0.829071
H	-2.211447	4.012414	-2.583098	-2.264185	4.028400	-2.502592	-2.328383	3.105801	-2.440531	-2.398580	3.130857	-2.348181
H	-3.366366	5.353745	-2.512470	-3.425231	5.363473	-2.415263	-3.436469	4.481982	-2.312579	-3.527662	4.486844	-2.191218
H	-5.620001	4.486947	-2.756909	-5.675251	4.489531	-2.666818	-5.722109	3.671688	-2.226481	-5.800149	3.639685	-2.113703
H	-6.131919	2.951770	-1.863523	-6.179083	2.941679	-1.790864	-6.154318	2.204163	-1.188920	-6.206216	2.146195	-1.102973
H	2.327377	0.160649	-1.558585	2.279085	0.191557	-1.529716	3.382835	-2.666275	-1.375327	3.406672	-2.577177	-1.396955
H	3.856364	-0.671586	-1.297116	3.828321	-0.606516	-1.282486	1.699734	-2.169551	-1.504747	1.715191	-2.107638	-1.513761
H	1.067864	-2.012596	-1.419235	1.073860	-2.017760	-1.385876	4.152807	-0.287878	-1.636821	4.133738	-0.179971	-1.617946
H	0.845712	-4.071136	-2.394669	0.891375	-4.068632	-2.385542	4.127486	1.502956	-3.060776	4.073538	1.633597	-3.011513
H	1.999381	-4.360437	-3.712820	2.026697	-4.306154	-3.729639	3.138427	1.250439	-4.512863	3.086255	1.388249	-4.466112
H	2.254003	-5.122694	-2.146801	2.333214	-5.083065	-2.180174	2.526444	2.256254	-3.204265	2.458742	2.359704	-3.139071
H	4.788866	-2.269223	-2.153902	4.785949	-2.159056	-2.191905	0.586289	-0.980849	-2.729216	0.578620	-0.919531	-2.716838
H	4.624559	-4.023594	-1.997648	4.673979	-3.920504	-2.063960	0.421345	0.742799	-3.083364	0.382165	0.806374	-3.042214
H	4.372295	-3.258127	-3.561592	4.369656	-3.134938	-3.608353	1.030521	-0.345492	-4.325344	1.008726	-0.250046	-4.302921
H	4.587658	-1.207279	0.996176	4.572678	-1.158750	0.989273	4.567078	-2.527232	0.765980	4.587041	-2.456435	0.728240

	**2b-5**			**2b-6**			**2b-7**			**2b-8**		
**Element**	**X**	**Y**	**Z**	**X**	**Y**	**Z**	**X**	**Y**	**Z**	**X**	**Y**	**Z**
O	5.052492	-0.051462	4.784943	6.058375	-1.814034	3.087331	7.044123	0.628614	1.773763	5.776809	1.896270	3.282592
C	5.211747	-0.558321	3.529332	4.969021	-1.257020	3.688477	5.904350	1.231429	2.213073	5.966999	1.107549	2.187907
C	4.034501	-0.345119	2.661857	3.892327	-0.902675	2.740440	4.725114	0.943855	1.367673	4.732706	0.899720	1.399515
C	2.880775	0.322115	3.114931	2.722948	-0.319197	3.262696	3.495395	1.516357	1.742388	3.500814	1.467587	1.775324
C	1.821707	0.487646	2.238455	1.703675	0.027217	2.394405	2.382812	1.273670	0.957504	2.395359	1.237162	0.974001
C	0.478930	1.158937	2.400035	0.358592	0.672711	2.625695	0.931658	1.666323	1.102278	0.943010	1.627688	1.113927
C	-0.198582	0.894660	1.027861	-0.257823	0.690486	1.200441	0.339941	1.250672	-0.266363	0.362155	1.235165	-0.266241
C	-1.486083	0.032001	1.082131	-1.553696	-0.143984	1.030163	-1.014896	0.509609	-0.233172	-0.992685	0.493364	-0.255750
O	-2.409441	0.692816	1.945937	-2.509804	0.351721	1.965724	-0.865895	-0.671036	0.562085	-0.849353	-0.700001	0.521362
C	-1.227444	-1.341828	1.709977	-1.335316	-1.617889	1.388475	-1.433929	0.132877	-1.665029	-1.400350	0.140041	-1.696785

C	-2.081937	-0.188747	-0.332913	-2.086713	-0.074838	-0.424935	-2.068217	1.387649	0.465509	-2.051812	1.359467	0.449051
C	-2.370007	1.020147	-1.248480	-2.324618	1.294007	-1.097517	-3.453675	0.739188	0.598283	-3.438315	0.708965	0.558962
C	-3.491384	1.973561	-0.814594	-3.456066	2.162962	-0.532099	-4.413288	1.595343	1.430229	-4.404545	1.549561	1.399034
O	-2.970411	2.799846	0.260572	-2.974770	2.756648	0.703124	-4.568000	2.838415	0.724715	-4.552772	2.806108	0.716362
C	-3.995653	2.867990	-1.932441	-3.905161	3.265054	-1.474225	-5.766753	0.944243	1.666932	-5.760242	0.894861	1.611780
C	-2.986761	3.757715	-2.616485	-2.861164	4.254660	-1.930446	-6.593997	0.610134	0.449810	-6.577054	0.583963	0.381531
C	-5.293065	2.863888	-2.256652	-5.187429	3.344333	-1.845573	-6.196132	0.719203	2.913172	-6.200477	0.646851	2.849851
O	0.801590	0.231504	0.186376	0.774154	0.185352	0.289738	1.322364	0.331496	-0.857264	1.349177	0.326645	-0.864654
C	1.911238	-0.010196	0.934701	1.848083	-0.211514	1.023549	2.497301	0.466805	-0.177672	2.518691	0.449927	-0.173296
C	3.038789	-0.681031	0.444742	2.991959	-0.790976	0.461860	3.693383	-0.136938	-0.577599	3.718175	-0.148145	-0.573832
C	3.084414	-1.234153	-0.966411	3.095735	-1.062075	-1.026557	3.756579	-1.044807	-1.790236	3.791981	-1.035590	-1.800783
C	2.366418	-2.559951	-1.089426	2.374042	-2.327457	-1.434439	3.182186	-2.415929	-1.508348	3.215313	-2.411065	-1.546093
C	1.385240	-2.903731	-1.937819	1.430550	-2.485550	-2.375439	2.217817	-3.071361	-2.172785	2.255639	-3.054932	-2.228392
C	0.813009	-4.302316	-1.923781	0.846347	-3.850845	-2.655324	1.796132	-4.457410	-1.742418	1.831648	-4.448472	-1.825307
C	0.777479	-1.990676	-2.974671	0.878736	-1.380488	-3.242812	1.487021	-2.544854	-3.383762	1.533273	-2.507690	-3.435255
C	4.094882	-0.830133	1.344889	4.009860	-1.126865	1.358408	4.805866	0.127952	0.226973	4.820543	0.103931	0.245530
O	6.239681	-1.122522	3.201539	4.930390	-1.086972	4.893556	5.900046	1.930238	3.209641	7.060017	0.641918	1.924167
H	5.885143	-0.255539	5.248565	6.682503	-1.997223	3.813152	7.734596	0.894434	2.408316	6.650382	1.953549	3.711138
H	2.835722	0.698395	4.131306	2.652221	-0.151889	4.332791	3.452361	2.133374	2.634165	3.432389	2.071075	2.673910
H	-0.117672	0.753530	3.222386	-0.275729	0.122804	3.326862	0.457946	1.095755	1.910377	0.462332	1.043748	1.908325
H	0.582743	2.234480	2.586034	0.459735	1.689528	3.022881	0.781938	2.731390	1.302578	0.791230	2.689382	1.330493
H	-0.434267	1.838701	0.529508	-0.462090	1.717058	0.884901	0.276749	2.112590	-0.942735	0.303725	2.108546	-0.928215
H	-2.624613	1.554178	1.531229	-2.696996	1.281596	1.719805	-0.142753	-1.180738	0.157423	-0.124158	-1.203843	0.112994
H	-2.163015	-1.909980	1.734778	-2.275914	-2.163871	1.262434	-2.331585	-0.490324	-1.643321	-2.297829	-0.483776	-1.692303
H	-0.487668	-1.906200	1.135268	-0.575344	-2.072349	0.746593	-1.645059	1.024958	-2.266558	-1.607236	1.041801	-2.285216
H	-0.875662	-1.244892	2.740966	-1.029335	-1.729220	2.432515	-0.637809	-0.429310	-2.162457	-0.600106	-0.413576	-2.197154
H	-1.394242	-0.843933	-0.879941	-1.379898	-0.621560	-1.059783	-1.702105	1.628256	1.471394	-1.694146	1.582667	1.462000
H	-3.013177	-0.754217	-0.194346	-3.027376	-0.641491	-0.439630	-2.161861	2.336211	-0.075221	-2.140910	2.317219	-0.076031
H	-1.459179	1.603514	-1.427326	-1.402182	1.886039	-1.120901	-3.897413	0.584253	-0.391348	-3.874124	0.572453	-0.436869
H	-2.665089	0.613666	-2.224409	-2.579970	1.090631	-2.145463	-3.354628	-0.242552	1.073782	-3.343014	-0.281443	1.016937
H	-4.333127	1.380176	-0.429534	-4.318048	1.519671	-0.303963	-3.947355	1.783944	2.411368	-3.946846	1.719008	2.387559
H	-3.678236	3.410461	0.525445	-3.688421	3.315273	1.053377	-5.153431	3.398210	1.259051	-5.144705	3.354774	1.255067
H	-2.413540	4.334152	-1.881234	-2.316159	4.668737	-1.074316	-6.645128	1.469740	-0.227999	-6.621737	1.455951	-0.280738
H	-2.260650	3.168939	-3.191607	-2.115107	3.777240	-2.578633	-6.150521	-0.216817	-0.120027	-6.129158	-0.232689	-0.199570
H	-3.473657	4.455049	-3.304334	-3.312733	5.079530	-2.489159	-7.611525	0.318136	0.726415	-7.597135	0.287721	0.643825
H	-5.688614	3.498059	-3.046282	-5.543911	4.125819	-2.512172	-7.164450	0.267130	3.114871	-7.170676	0.191639	3.034724
H	-6.007580	2.217190	-1.752197	-5.927822	2.623443	-1.505931	-5.588978	0.968769	3.780741	-5.600706	0.879784	3.727133
H	2.671003	-0.498147	-1.662327	2.719026	-0.199091	-1.583565	3.241573	-0.569725	-2.630807	3.284127	-0.546586	-2.637729
H	4.138741	-1.373410	-1.244366	4.160345	-1.161351	-1.282379	4.810226	-1.150556	-2.084796	4.848132	-1.136502	-2.087217
H	2.719617	-3.319955	-0.391064	2.687664	-3.213236	-0.880175	3.633796	-2.916266	-0.650510	3.661605	-2.926173	-0.694288
H	1.290820	-4.932282	-1.167032	1.283236	-4.622293	-2.013563	2.354037	-4.801723	-0.866050	2.383560	-4.807744	-0.951165
H	-0.267320	-4.286804	-1.721492	-0.241934	-3.855639	-2.500963	0.724685	-4.488981	-1.498833	0.758456	-4.484923	-1.590076
H	0.938206	-4.790809	-2.900677	1.012094	-4.145248	-3.701493	1.950264	-5.185908	-2.551002	1.992143	-5.162664	-2.645336
H	-0.317789	-1.984052	-2.887041	1.231593	-0.387758	-2.955734	0.400260	-2.586355	-3.227714	0.445378	-2.553034	-3.288213
H	1.123435	-0.957784	-2.897792	1.145548	-1.543974	-4.296705	1.748827	-1.514927	-3.635356	1.795912	-1.473213	-3.666536
H	1.003488	-2.349761	-3.988871	-0.219177	-1.370304	-3.200826	1.695677	-3.170206	-4.263285	1.749208	-3.117054	-4.324196
H	5.004056	-1.335367	1.031781	4.921950	-1.573645	0.975327	5.764356	-0.304497	-0.041837	5.789227	-0.316868	-0.007874

	2b-9			2b-10			2b-11			2b-12		
**Element**	**X**	**Y**	**Z**	**X**	**Y**	**Z**	**X**	**Y**	**Z**	**X**	**Y**	**Z**
O	5.606250	0.345045	2.837099	6.407820	0.766513	2.098376	4.640564	2.927428	4.151465	6.798269	0.551004	2.216823
C	4.478052	0.835451	3.420134	5.231490	1.154545	2.662939	5.113585	2.189967	3.109043	5.636552	1.016060	2.753008
C	3.364773	1.037056	2.466224	4.081686	1.080461	1.733040	4.066451	1.839806	2.122933	4.492214	0.953826	1.816056
C	2.155444	1.547427	2.972268	2.818472	1.458197	2.225321	2.726288	2.237754	2.283200	3.242461	1.403558	2.279864
C	1.102653	1.749506	2.098237	1.732713	1.409409	1.370115	1.811081	1.881559	1.306479	2.162503	1.369302	1.415912
C	-0.301453	2.264539	2.315839	0.261702	1.686791	1.575865	0.316569	2.075362	1.199547	0.700403	1.699828	1.603628
C	-0.868205	2.315146	0.870696	-0.270642	1.692588	0.122618	0.033642	1.636280	-0.258288	0.166099	1.654361	0.150982
C	-2.228595	1.638261	0.594036	-1.619972	0.987435	-0.128510	-1.189136	0.720709	-0.487048	-1.175016	0.923219	-0.078155
O	-2.498979	1.860365	-0.808672	-1.480811	-0.387430	0.278384	-0.971007	-0.489150	0.262548	-0.996499	-0.447129	0.326185
C	-3.334575	2.384016	1.343060	-1.975385	1.055985	-1.621256	-1.319817	0.392194	-1.982050	-1.551875	0.983443	-1.566281
C	-2.189124	0.125224	0.909176	-2.702559	1.616420	0.768988	-2.454376	1.389484	0.082209	-2.260989	1.530025	0.829896
C	-3.495114	-0.686742	0.778919	-4.163070	1.173385	0.556764	-3.812082	0.712817	-0.188569	-3.712982	1.051528	0.636555
C	-4.103230	-0.877101	-0.635511	-4.435796	-0.342903	0.739591	-3.950483	-0.740709	0.336290	-3.945636	-0.470773	0.825746
O	-4.742816	0.294351	-1.104336	-3.749478	-0.861526	1.876628	-3.390540	-0.878571	1.640188	-3.229705	-0.970575	1.952717
C	-3.115614	-1.481522	-1.632587	-5.921489	-0.608604	0.910391	-5.409432	-1.159609	0.390718	-5.421648	-0.772436	1.019217
C	-2.487884	-2.798813	-1.239879	-6.499039	-0.383310	2.284663	-6.204710	-0.699637	1.586089	-5.983399	-0.560037	2.402057
C	-2.860075	-0.902507	-2.810648	-6.664085	-1.010723	-0.125167	-5.944571	-1.890723	-0.591432	-6.170088	-1.193267	-0.004639
O	0.134609	1.686669	0.006599	0.744342	0.988004	-0.669130	1.215933	0.877261	-0.679803	1.181018	0.929265	-0.621861
C	1.255563	1.439353	0.744097	1.909428	0.982395	0.051550	2.228407	1.139255	0.200312	2.331033	0.888488	0.115612
C	2.440268	0.938466	0.196478	3.141421	0.594961	-0.479450	3.543929	0.705126	0.011015	3.547243	0.412511	-0.381672
C	2.567637	0.624797	-1.283827	3.282237	0.131252	-1.918143	3.934110	-0.148322	-1.179571	3.663168	-0.152481	-1.783732
C	1.983573	-0.722123	-1.647494	2.854608	-1.307193	-2.106073	3.477271	-1.582815	-1.026255	3.070023	-1.540601	-1.890076
C	2.617302	-1.796162	-2.141993	3.589022	-2.348029	-2.526155	2.733958	-2.318198	-1.867940	2.136011	-1.987694	-2.744166
C	1.855035	-3.065590	-2.442978	2.973241	-3.722630	-2.645992	2.405602	-3.757470	-1.543679	1.691515	-3.431824	-2.707608

C	4.093419	-1.873369	-2.447514	5.045996	-2.284651	-2.914823	2.184907	-1.835072	-3.188282	1.466805	-1.155626	-3.810908
C	3.489959	0.742296	1.098819	4.225275	0.653941	0.403213	4.450293	1.082055	1.004501	4.626012	0.462797	0.506821
O	4.429606	1.071356	4.613073	5.171783	1.519526	3.822066	6.285569	1.872163	3.034500	5.584726	1.433119	3.894860
H	6.252922	0.258922	3.561379	7.074144	0.859039	2.803841	5.412925	3.089067	4.723762	7.462971	0.644135	2.923726
H	2.081589	1.772096	4.031485	2.730195	1.778997	3.258243	2.429656	2.809857	3.155498	3.158480	1.765269	3.299586
H	-0.881349	1.600156	2.967470	-0.207555	0.883058	2.156298	-0.206882	1.424203	1.909991	0.211260	0.937557	2.221938
H	-0.319373	3.257063	2.777942	0.059897	2.633810	2.084647	-0.009271	3.103759	1.381694	0.524886	2.674440	2.068430
H	-0.952822	3.349723	0.521486	-0.314339	2.717414	-0.268187	-0.030302	2.508839	-0.920695	0.112361	2.664542	-0.274289
H	-1.804966	1.398663	-1.311757	-0.720637	-0.750847	-0.207080	-0.108620	-0.842909	-0.018242	-0.227904	-0.787680	-0.164676
H	-4.312708	2.032373	1.005566	-2.877282	0.473785	-1.825476	-2.113629	-0.341533	-2.142625	-2.440372	0.377005	-1.758832
H	-3.260509	2.223389	2.423584	-2.154445	2.090954	-1.935108	-1.558890	1.290924	-2.562611	-1.763843	2.013291	-1.876776
H	-3.274523	3.459042	1.142810	-1.159773	0.656039	-2.231445	-0.383592	-0.020643	-2.369712	-0.733917	0.605531	-2.187089
H	-1.413295	-0.324988	0.277528	-2.434821	1.417387	1.811985	-2.332002	1.481070	1.166340	-1.977968	1.341684	1.870615
H	-1.839072	0.003503	1.941918	-2.656869	2.705599	0.634390	-2.500089	2.411799	-0.317220	-2.242327	2.619517	0.690611
H	-3.291413	-1.679992	1.199423	-4.765135	1.731868	1.284299	-4.572436	1.338449	0.295386	-4.319505	1.596769	1.370430
H	-4.280723	-0.252018	1.407198	-4.524731	1.474223	-0.434207	-4.047962	0.719189	-1.259843	-4.094325	1.341073	-0.350400
H	-4.915519	-1.609588	-0.513638	-4.094389	-0.866234	-0.164214	-3.425985	-1.409826	-0.359745	-3.605051	-0.986945	-0.082509
H	-4.052697	0.988750	-1.154135	-2.820113	-0.944096	1.596459	-2.426275	-0.897674	1.501091	-2.302786	-1.030454	1.658225
H	-1.816567	-2.699574	-0.376876	-6.010348	-1.041073	3.011380	-5.763751	-1.095631	2.507136	-5.467583	-1.204935	3.121506
H	-3.255863	-3.531127	-0.954836	-6.332294	0.644117	2.633953	-6.198817	0.393928	1.683735	-5.836360	0.471495	2.748100
H	-1.910020	-3.223799	-2.066447	-7.575979	-0.577122	2.297759	-7.245788	-1.030234	1.519661	-7.054971	-0.780040	2.431883
H	-2.179418	-1.353414	-3.528679	-7.736013	-1.168715	-0.033845	-6.996000	-2.167915	-0.591180	-7.236346	-1.377173	0.103136
H	-3.360966	0.012309	-3.109961	-6.229463	-1.198865	-1.104597	-5.352313	-2.241838	-1.433770	-5.746050	-1.371506	-0.990552
H	2.040285	1.403778	-1.851647	2.655716	0.772395	-2.553970	3.532873	0.295133	-2.096119	3.189973	0.531388	-2.495134
H	3.621707	0.687887	-1.567765	4.316386	0.282053	-2.239619	5.028855	-0.127673	-1.271753	4.728762	-0.195635	-2.049196
H	0.910429	-0.804909	-1.472226	1.810234	-1.496310	-1.854823	3.819360	-2.059307	-0.106504	3.479624	-2.252304	-1.171882
H	0.790738	-2.969630	-2.206277	1.917422	-3.725561	-2.357461	2.825689	-4.066920	-0.581666	2.203691	-3.998617	-1.923933
H	1.944890	-3.339552	-3.503662	3.045383	-4.100483	-3.675571	1.319005	-3.920205	-1.510203	0.608974	-3.510822	-2.534149
H	2.256236	-3.912770	-1.868921	3.501107	-4.448342	-2.011425	2.794725	-4.434319	-2.317320	1.886569	-3.928639	-3.668436
H	4.634695	-0.949719	-2.230217	5.481461	-1.287379	-2.819271	1.101556	-2.009653	-3.244084	1.739497	-0.098783	-3.772743
H	4.564395	-2.680671	-1.869662	5.639917	-2.970048	-2.294528	2.362486	-0.772969	-3.370374	1.721353	-1.533042	-4.811261
H	4.256856	-2.115453	-3.506815	5.183161	-2.611228	-3.954991	2.629745	-2.398226	-4.020701	0.373531	-1.224368	-3.726084
H	4.434975	0.356784	0.730260	5.210252	0.370758	0.046752	5.493725	0.792312	0.921952	5.599159	0.117356	0.172908

	2b-13			2b-14			2b-15			2b-16		
**Element**	**X**	**Y**	**Z**	**X**	**Y**	**Z**	**X**	**Y**	**Z**	**X**	**Y**	**Z**
O	6.260674	-0.317676	2.339120	5.335353	2.487194	3.654756	6.814226	0.498090	2.281455	6.424322	0.703549	2.167020
C	5.110181	0.155298	2.892009	5.618112	1.729429	2.559331	5.653959	0.948826	2.832813	5.250939	1.083322	2.743215
C	3.954609	0.113945	1.967269	4.435775	1.481809	1.703534	4.506529	0.908739	1.898383	4.097796	1.030690	1.815941
C	2.713909	0.567561	2.452018	3.162063	1.986202	2.024985	3.257903	1.345562	2.377330	2.837196	1.401567	2.320001
C	1.622503	0.545071	1.602545	2.110871	1.721487	1.162771	2.175110	1.331646	1.516342	1.748482	1.372681	1.467635
C	0.165571	0.888980	1.808336	0.635691	2.039288	1.236302	0.713339	1.656008	1.716985	0.278850	1.649551	1.683881
C	-0.377046	0.874145	0.358908	0.146150	1.647328	-0.179620	0.174488	1.646365	0.265351	-0.258004	1.686535	0.232774
C	-1.752270	0.210795	0.134953	-1.167488	0.839356	-0.265488	-1.167056	0.920506	0.022362	-1.610270	0.990803	-0.028488
O	-1.657439	-1.157746	0.574572	-0.963426	-0.396257	0.445110	-0.986169	-0.459684	0.390703	-1.473248	-0.393051	0.347724
C	-2.116086	0.254904	-1.356748	-1.501525	0.546115	-1.736130	-1.548517	1.018074	-1.462723	-1.970253	1.092216	-1.518382
C	-2.805585	0.898837	1.023774	-2.295300	1.602204	0.454786	-2.249884	1.504632	0.949154	-2.687446	1.604474	0.886257
C	-4.282241	0.502653	0.831839	-3.728136	1.046950	0.344730	-3.702977	1.033447	0.749131	-4.150744	1.174159	0.669610
C	-4.606747	-0.998360	1.052522	-3.922873	-0.396078	0.885472	-3.932754	-0.494084	0.911702	-4.426507	-0.345776	0.830254
O	-3.930840	-1.513942	2.197093	-3.220966	-0.576749	2.105401	-3.208003	-0.998041	2.022576	-3.733103	-0.868221	1.952756
C	-6.099615	-1.207188	1.239059	-5.394595	-0.716164	1.078989	-5.409264	-0.814099	1.064757	-5.913654	-0.623364	0.961568
C	-6.658867	-0.929187	2.611257	-6.255170	-0.664814	-0.161074	-6.297501	-0.426651	-0.093652	-6.773113	-0.211912	-0.210207
C	-6.863380	-1.606953	0.218095	-5.888724	-1.037290	2.276949	-5.884678	-1.415971	2.157514	-6.422154	-1.210564	2.047383
O	0.606139	0.113122	-0.420900	1.202332	0.799869	-0.743408	1.187231	0.941204	-0.528487	0.752246	0.995001	-0.576382
C	1.772782	0.073541	0.296324	2.330549	0.964286	0.010760	2.339845	0.883364	0.204001	1.919796	0.971855	0.140176
C	2.979941	-0.397329	-0.224431	3.573371	0.426306	-0.336766	3.554806	0.421150	-0.308970	3.148967	0.592064	-0.402663
C	3.096485	-0.887243	-1.656803	3.746877	-0.438152	-1.569946	3.666695	-0.108690	-1.725018	3.283942	0.157014	-1.850844
C	3.136778	0.250334	-2.652899	3.193235	-1.832316	-1.370659	3.074931	-1.494517	-1.863781	2.853001	-1.276613	-2.066059
C	4.111270	0.562150	-3.520234	2.295892	-2.490937	-2.120814	2.137910	-1.921383	-2.724890	3.584041	-2.310150	-2.509399
C	3.965089	1.751813	-4.440439	1.889298	-3.903042	-1.767121	1.695386	-3.366577	-2.722626	2.965351	-3.681009	-2.654476
C	5.407826	-0.192956	-3.683594	1.635499	-1.943983	-3.362953	1.463072	-1.063785	-3.767600	5.039800	-2.241589	-2.901736
C	4.070372	-0.360871	0.650892	4.619556	0.712213	0.543222	4.636480	0.450501	0.576944	4.235895	0.630602	0.477367
O	5.075408	0.562947	4.037854	6.742268	1.317386	2.343486	5.605613	1.337556	3.984743	5.196069	1.425362	3.909532
H	6.934135	-0.237281	3.039272	6.180865	2.575903	4.131656	7.481214	0.574282	2.988238	7.093273	0.780336	2.871887
H	2.645527	0.920171	3.476043	3.019842	2.567344	2.929550	3.176927	1.681769	3.405983	2.753117	1.701760	3.359442
H	-0.331541	0.123014	2.415943	0.147631	1.422817	2.000818	0.226800	0.878198	2.317741	-0.190720	0.835420	2.249355
H	0.006356	1.858216	2.289717	0.420813	3.088101	1.461561	0.538450	2.618532	2.206523	0.081103	2.586456	2.212561
H	-0.387252	1.888811	-0.059468	0.077346	2.532194	-0.825003	0.118957	2.666783	-0.134503	-0.299642	2.719240	-0.136956
H	-0.917957	-1.560351	0.087936	-0.171035	-0.813118	0.062889	-0.216826	-0.785589	-0.108900	-0.713067	-0.746178	-0.145323
H	-3.039751	-0.299232	-1.540516	-2.366430	-0.118414	-1.804882	-2.436436	0.414925	-1.668132	-2.873387	0.515384	-1.732437
H	-2.260093	1.287469	-1.695599	-1.732721	1.470314	-2.278724	-1.762928	2.055177	-1.746362	-2.148712	2.133991	-1.809346
H	-1.319842	-0.189495	-1.961796	-0.655373	0.064593	-2.235503	-0.731945	0.657361	-2.095454	-1.157184	0.704361	-2.139654
H	-2.537446	0.715520	2.069530	-2.038207	1.667352	1.516664	-1.963905	1.290924	1.983780	-2.417562	1.382326	1.923643
H	-2.722638	1.982210	0.862618	-2.301376	2.630471	0.068175	-2.231003	2.597197	0.836178	-2.637352	2.696059	0.774330

H	-4.858907	1.098996	1.549838	-4.372762	1.717539	0.927241	-4.309645	1.550046	1.503848	-4.751238	1.708451	1.416899
H	-4.640565	0.792583	-0.163583	-4.079366	1.099612	-0.692921	-4.078347	1.358077	-0.228930	-4.504950	1.508802	-0.312952
H	-4.290510	-1.554535	0.159245	-3.528235	-1.098086	0.131552	-3.580478	-0.991004	-0.008087	-4.074950	-0.854279	-0.083434
H	-3.007091	-1.636132	1.912899	-2.289318	-0.694650	1.847290	-2.287037	-1.069541	1.714084	-2.810298	-0.967250	1.658423
H	-6.188050	-1.586185	3.350354	-6.334281	0.352225	-0.567659	-6.352447	0.662054	-0.226473	-6.795862	0.877843	-0.343804
H	-6.453885	0.099806	2.934680	-5.839658	-1.291830	-0.962756	-5.922773	-0.839848	-1.040944	-6.395730	-0.635744	-1.151706
H	-7.741779	-1.084815	2.636010	-7.269286	-1.017230	0.050080	-7.317406	-0.794518	0.053626	-7.804921	-0.551293	-0.078332
H	-7.939527	-1.724675	0.319967	-6.942962	-1.270846	2.406365	-6.942722	-1.646893	2.257228	-7.487682	-1.411333	2.131328
H	-6.442703	-1.833368	-0.759321	-5.252219	-1.080745	3.153056	-5.228952	-1.697191	2.973312	-5.786987	-1.509673	2.873021
H	3.981737	-1.523324	-1.742095	3.278227	0.050700	-2.429654	3.190283	0.592017	-2.417637	2.656472	0.811906	-2.471545
H	2.229199	-1.523987	-1.883420	4.820821	-0.506340	-1.792132	4.731437	-0.143895	-1.995005	4.317265	0.312338	-2.172817
H	2.250136	0.884120	-2.636198	3.601542	-2.349466	-0.501264	3.488433	-2.223433	-1.165361	1.809156	-1.468889	-1.814973
H	3.012559	2.269805	-4.291280	2.393667	-4.260706	-0.864137	2.211442	-3.952047	-1.955407	1.910476	-3.687796	-2.362567
H	4.775572	2.476845	-4.280325	0.804656	-3.976194	-1.604293	0.613656	-3.451378	-2.546816	3.033459	-4.038492	-3.691577
H	4.025440	1.447062	-5.494798	2.125468	-4.597765	-2.585442	1.887172	-3.838993	-3.696332	3.493949	-4.420096	-2.036167
H	5.505233	-1.046825	-3.009167	0.542380	-2.026600	-3.288405	1.735669	-0.008079	-3.705057	5.477414	-1.247195	-2.787775
H	5.511340	-0.565965	-4.712035	1.879316	-0.897830	-3.560113	1.712586	-1.416592	-4.778149	5.634550	-2.940289	-2.297283
H	6.265219	0.471451	-3.508039	1.928019	-2.529860	-4.245634	0.370243	-1.135007	-3.678875	5.172847	-2.547575	-3.948679
H	5.037656	-0.706566	0.301195	5.617954	0.338890	0.335009	5.608860	0.114481	0.231381	5.218965	0.352211	0.111982

3.3. Compound 3

Table S3.1. Detailed DP4+ probability of **3a** (Isomer 1) and **3b** (Isomer 2)

Functional	Solvent?		Basis Set		Type of Data	
B3LYP	PCM		6-31+G(d,p)		Shielding Tensors	
	Isomer 1	Isomer 2	Isomer 3	Isomer 4	Isomer 5	Isomer 6
sDP4+ (H data)	86.30%	13.70%	-	-	-	-
sDP4+ (C data)	99.91%	0.09%	-	-	-	-
sDP4+ (all data)	99.99%	0.01%	-	-	-	-
uDP4+ (H data)	29.30%	70.70%	-	-	-	-
uDP4+ (C data)	98.24%	1.76%	-	-	-	-
uDP4+ (all data)	95.85%	4.15%	-	-	-	-
DP4+ (H data)	72.30%	27.70%	-	-	-	-
DP4+ (C data)	100.00%	0.00%	-	-	-	-
DP4+ (all data)	100.00%	0.00%	-	-	-	-

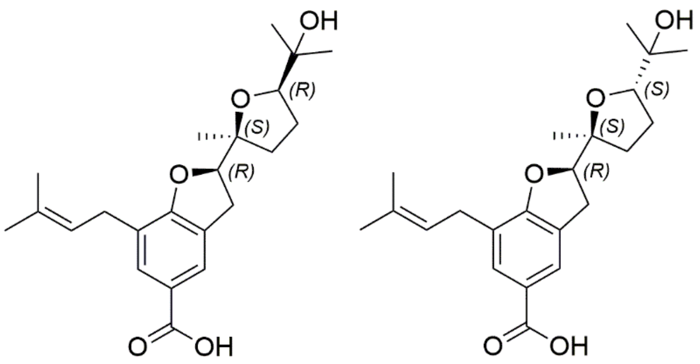

2*R*,1'*S*,4'*R*-**3** (**3a**)   2*R*,1'*R*,4'*S*-**3** (**3b**)

Table S3.2. Experimental and calculated ^{1}H-NMR chemical shifts (δ in ppm) of **3a** and **3b**

**Proton**									
**3**		**3a (2*R*,1'*S*,4'*R*)**				**3b (2*R*,1'*S*,4'*S*)**			
No.	δ_H Exp. (mult., *J* in Hz)	δ_H Calc.	|$\Delta\delta$|	δ_H Calc. CORRECTED	|$\Delta\delta$|	δ_H Calc.	|$\Delta\delta$|	δ_H Calc. CORRECTED	|$\Delta\delta$|
2	4.83 (1H, m)	4.93	0.10	4.69	0.14	4.94	0.11	4.72	0.11
3α	3.23 (1H, 9.4)	3.38	0.15	3.21	0.02	3.39	0.16	3.23	0.00
3β	3.04 (1H, dd, 16.1, 7.8)	3.25	0.21	3.09	0.05	3.35	0.31	3.20	0.16
4	7.73 (1H, s)	8.11	0.38	7.70	0.03	8.10	0.37	7.75	0.02
6	7.74 (1H, s)	8.09	0.35	7.69	0.05	8.00	0.26	7.66	0.08
2'α	2.02 (1H, dt, 12.1, 9.0)	2.36	0.34	2.24	0.22	2.19	0.17	2.07	0.05
2'β	1.74 (1H, m)	1.85	0.11	1.76	0.02	2.10	0.36	2.00	0.26
3'	1.88 (2H, m)	1.99	0.11	1.90	0.02	2.01	0.13	1.91	0.03
4'	3.91 (1H, t, 7.6)	4.17	0.26	3.96	0.05	4.15	0.24	3.97	0.06
6'	1.22 (3H, s)	1.23	0.01	1.17	0.05	1.22	0.00	1.15	0.07
7'	1.19 (3H, s)	1.06	0.13	1.01	0.18	1.05	0.14	0.98	0.21
8'	1.13 (3H, s)	1.18	0.05	1.12	0.01	1.16	0.03	1.09	0.04
1"	3.27 (2H, t, 7.5)	3.39	0.12	3.22	0.05	3.34	0.07	3.18	0.09
2"	5.26 (1H, ddt, 7.4, 5.8, 1.5)	5.67	0.41	5.39	0.13	5.53	0.27	5.29	0.03
4"	1.71 (3H, s)	1.89	0.18	1.80	0.09	1.87	0.16	1.77	0.06
5"	1.70 (3H, s)	1.73	0.03	1.64	0.06	1.73	0.03	1.64	0.06
		**MAE**	**0.18**	**CMAE**	**0.07**	**MAE**	**0.18**	**CMAE**	**0.08**

Table S3.3. Experimental and calculated ^{13}C-NMR chemical shifts (δ in ppm) of **3a** and **3b**

**Carbon**									
	**3**	**3a (2*R*,1'*S*,4'*R*)**				**3b (2*R*,1'*S*,4'*S*)**			
No.	δ_C Exp.	δ_C Calc.	$\lvert\Delta\delta\rvert$	δ_C Calc. CORRECTED	$\lvert\Delta\delta\rvert$	δ_C Calc.	$\lvert\Delta\delta\rvert$	δ_C Calc. CORRECTED	$\lvert\Delta\delta\rvert$
2	89.01	90.65	1.64	90.45	1.44	92.73	3.72	92.57	3.56
3	31.51	35.21	3.70	32.45	0.94	35.45	3.94	32.69	1.18
3a	127.10	126.26	0.84	127.70	0.60	126.35	0.75	127.72	0.62
4	125.14	123.03	2.11	124.32	0.82	123.26	1.88	124.49	0.65
5	121.80	118.84	2.96	119.93	1.87	118.68	3.12	119.70	2.10
6	131.73	130.38	1.35	132.01	0.28	129.85	1.88	131.38	0.35
7	123.38	123.42	0.04	124.73	1.35	122.88	0.50	124.09	0.71
7a	163.05	159.97	3.08	162.97	0.08	160.73	2.32	163.67	0.62
1'	84.68	88.28	3.60	87.97	3.29	87.37	2.69	86.96	2.28
2'	33.76	37.85	4.09	35.21	1.45	40.29	6.53	37.74	3.98
3'	26.61	30.49	3.88	27.51	0.90	29.83	3.22	26.81	0.20
4'	87.45	88.08	0.63	87.76	0.31	88.83	1.38	88.50	1.05
5'	71.03	73.96	2.93	72.98	1.95	72.73	1.70	71.66	0.63
6'	27.78	29.30	1.52	26.27	1.51	25.34	2.44	22.12	5.66
7'	22.82	23.40	0.58	20.10	2.72	21.65	1.17	18.25	4.57
8'	24.23	25.36	1.13	22.14	2.09	29.53	5.30	26.49	2.26
1"	28.45	33.82	5.37	30.99	2.54	34.61	6.16	31.81	3.36
2"	121.62	122.06	0.44	123.31	1.69	121.71	0.09	122.87	1.25
3"	133.32	133.28	0.04	135.04	1.72	132.96	0.36	134.63	1.31
4"	26.00	27.70	1.70	24.59	1.41	27.64	1.64	24.51	1.49
5"	18.04	19.25	1.21	15.76	2.28	19.17	1.13	15.66	2.38
COOH	172.14	163.30	8.84	166.45	5.69	163.29	8.85	166.34	5.80
		**MAE**	**2.35**	**CMAE**	**1.68**	**MAE**	**2.76**	**CMAE**	**2.09**

Table S3.4. Calculated conformational analysis of the **3a** at B3LYP/6-31g(d) level

conformer	3D conformer	G (Hartree)	ΔG (kcal/mol)	Population
3a-1	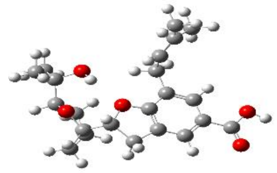	-1232.116478	0	17.552%
3a-2	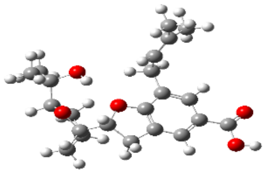	-1232.116307	0.10730421	14.644%
3a-3	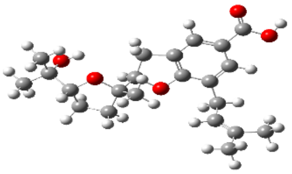	-1232.115594	0.55471884	6.881%
3a-4	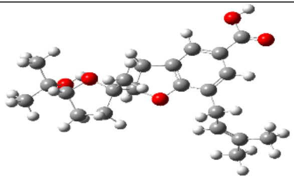	-1232.115548	0.5835843	6.554%
3a-5	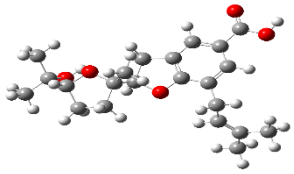	-1232.115508	0.6086847	6.282%
3a-6	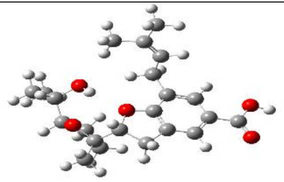	-1232.115479	0.62688249	6.092%
3a-7	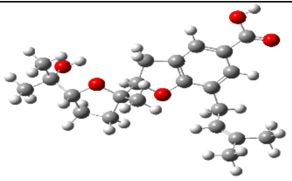	-1232.115459	0.63943269	5.964%
3a-8	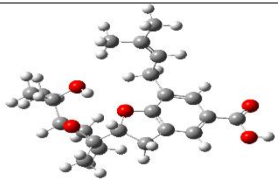	-1232.115356	0.70406622	5.348%

3a-9	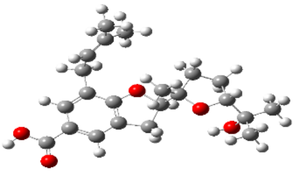	-1232.115213	0.79380015	4.596%
3a-10	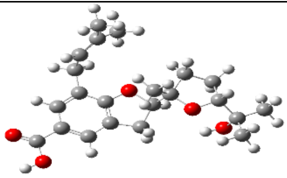	-1232.115183	0.81262545	4.452%
3a-11	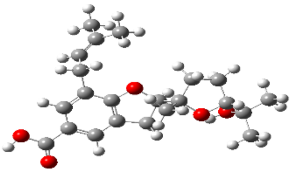	-1232.115155	0.83019573	4.322%
3a-12	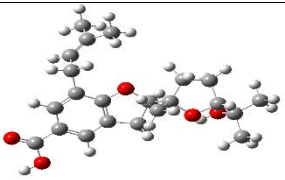	-1232.115126	0.84839352	4.192%
3a-13	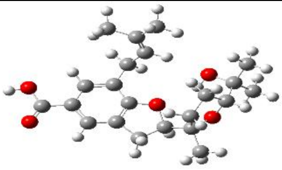	-1232.11475	1.08433728	2.815%
3a-14	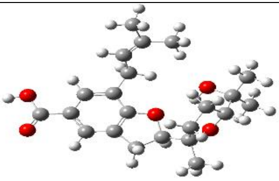	-1232.114731	1.09625997	2.758%
3a-15	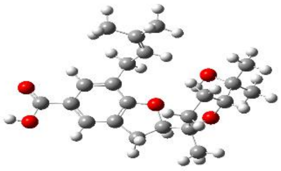	-1232.114623	1.16403105	2.460%
3a-16	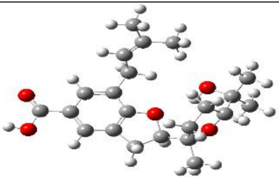	-1232.114546	1.21234932	2.268%
3a-17	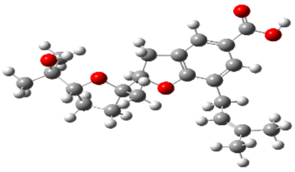	-1232.114154	1.45833324	1.497%

3a-18	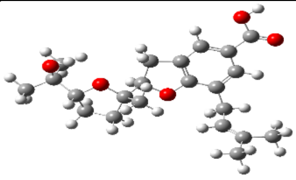	-1232.114037	1.53175191	1.323%

Table S3.5. Cartesian coordinates of low-energy conformers of **3a**

	**3a-1**			**3a-2**			**3a-3**			**3a-4**		
**Element**	**X**	**Y**	**Z**	**X**	**Y**	**Z**	**X**	**Y**	**Z**	**X**	**Y**	**Z**
O	-5.441947	-1.921242	-2.049419	-4.453479	-4.042348	-2.160944	-5.908790	-1.187472	-2.450450	-5.166718	-3.075125	-1.443489
C	-4.421246	-2.812433	-1.917604	-4.464370	-2.698646	-1.933312	-4.809230	-1.976734	-2.297272	-5.232484	-1.739022	-1.183797
C	-3.152273	-2.191277	-1.478455	-3.153680	-2.173752	-1.490954	-3.597916	-1.225696	-1.902290	-3.919393	-1.146679	-0.848126
C	-2.038541	-3.033671	-1.305978	-2.033780	-3.009241	-1.322413	-2.406105	-1.952444	-1.725853	-2.741923	-1.916263	-0.803262
C	-0.841994	-2.474739	-0.895606	-0.843350	-2.438986	-0.904107	-1.259627	-1.271126	-1.358871	-1.552421	-1.286571	-0.476726
C	0.506207	-3.089696	-0.598381	0.509332	-3.044218	-0.606500	0.155655	-1.737789	-1.111602	-0.139997	-1.807240	-0.347916
C	1.377377	-1.848938	-0.254889	1.369454	-1.797519	-0.256659	0.878845	-0.424005	-0.699294	0.648717	-0.541935	0.095183
C	2.136427	-1.894795	1.085125	2.132945	-1.845132	1.080753	1.482774	-0.415223	0.723984	1.329074	-0.632701	1.480637
C	3.190955	-3.007577	1.037927	3.197372	-2.947982	1.023106	0.432870	-0.621550	1.815432	0.330934	-0.856261	2.616248
C	1.219335	-1.987883	2.332980	1.220540	-1.954698	2.330703	2.341551	0.850813	0.951034	2.248164	0.586927	1.724393
C	1.160089	-0.554020	2.879552	1.149853	-0.525030	2.886810	3.754096	0.409635	0.546386	3.617698	0.117092	1.217376
C	2.488874	0.057895	2.418590	2.471146	0.102538	2.425300	3.770871	-1.062453	0.967133	3.599642	-1.374881	1.561071
C	2.452003	1.572007	2.104777	2.418091	1.618072	2.120747	4.707557	-1.996485	0.166103	4.454044	-2.298718	0.662147
C	2.069564	2.372447	3.352197	2.034319	2.407046	3.375075	6.146387	-1.479185	0.184112	5.910549	-1.834902	0.624443
O	1.433920	1.830993	1.133505	1.392517	1.873354	1.156575	4.295693	-1.993048	-1.205238	3.967698	-2.208820	-0.681617
C	3.813496	2.035106	1.559563	3.772295	2.097649	1.571618	4.634827	-3.426713	0.725949	4.356732	-3.752745	1.152670
O	2.819275	-0.630220	1.196829	2.804530	-0.575318	1.198477	2.418384	-1.522191	0.752965	2.220443	-1.773213	1.400508
O	0.477717	-0.697747	-0.256296	0.458804	-0.655586	-0.247795	-0.107602	0.648960	-0.803481	-0.305261	0.564255	0.107102
C	-0.762877	-1.099274	-0.659856	-0.777009	-1.065225	-0.658319	-1.307130	0.112295	-1.166837	-1.543358	0.082846	-0.201748
C	-1.842172	-0.224499	-0.815253	-1.863757	-0.198136	-0.810695	-2.466134	0.875806	-1.338993	-2.689166	0.884986	-0.233803
C	-1.709112	1.271018	-0.583328	-1.744615	1.296877	-0.568937	-2.469766	2.378893	-1.123206	-2.626884	2.373357	0.057698
C	-1.240196	1.992928	-1.827067	-1.259210	2.027954	-1.801059	-2.475606	2.757312	0.341071	-2.061723	3.163463	-1.101779
C	-1.863453	2.960177	-2.516245	-1.886209	2.980329	-2.507306	-3.418499	3.421678	1.025830	-2.657132	4.126908	-1.820405
C	-1.220569	3.562666	-3.743852	-1.225829	3.595237	-3.719369	-3.240157	3.703602	2.499680	-1.921144	4.800933	-2.955136
C	-3.210638	3.546222	-2.170289	-3.254993	3.535570	-2.197574	-4.709139	3.946833	0.445718	-4.061094	4.636039	-1.602152
C	-3.039547	-0.812771	-1.235168	-3.051735	-0.796506	-1.236945	-3.611403	0.165824	-1.709765	-3.875082	0.228892	-0.567301
O	-4.578898	-3.997356	-2.147706	-5.473401	-2.037262	-2.090491	-4.861900	-3.179442	-2.477411	-6.287653	-1.134745	-1.234391
H	-6.207580	-2.449760	-2.340240	-5.360653	-4.260994	-2.442577	-6.625899	-1.795799	-2.706888	-6.081876	-3.340960	-1.648063
H	-2.147547	-4.096334	-1.497708	-2.114702	-4.073099	-1.517432	-2.416428	-3.025921	-1.885429	-2.778692	-2.978254	-1.020618
H	0.452375	-3.794746	0.240310	0.459964	-3.752533	0.229890	0.225490	-2.500429	-0.329459	-0.045834	-2.617527	0.382161
H	0.917102	-3.639866	-1.451536	0.927291	-3.587836	-1.460521	0.604362	-2.167437	-2.014923	0.239156	-2.192931	-1.301710
H	2.112198	-1.651750	-1.043160	2.100114	-1.588341	-1.045793	1.675549	-0.176751	-1.408046	1.413082	-0.278764	-0.642702
H	3.852193	-2.882029	0.173623	3.854958	-2.810666	0.157798	-0.134492	-1.545283	1.666441	-0.284173	-1.745991	2.450178
H	3.806250	-2.966775	1.942078	3.814917	-2.907624	1.925738	0.927100	-0.685791	2.789974	0.872641	-0.994154	3.557302
H	2.731200	-4.000928	0.983152	2.746456	-3.945101	0.963019	-0.271546	0.215813	1.837508	-0.332107	0.008205	2.720109
H	1.671316	-2.664751	3.066920	1.680869	-2.632256	3.058821	2.317556	1.129048	2.011133	2.293421	0.807562	2.797254
H	0.226779	-2.383651	2.097706	0.230818	-2.357759	2.095784	1.966500	1.703697	0.378186	1.875435	1.482750	1.219615
H	0.334180	0.000976	2.428794	0.317178	0.025136	2.442636	3.900796	0.477543	-0.536791	3.706845	0.237131	0.132415
H	1.044950	-0.526070	3.967125	1.038326	-0.505282	3.974932	4.536887	0.996933	1.035136	4.448806	0.648670	1.689824
H	3.283561	-0.133252	3.157483	3.270129	-0.085103	3.160432	4.008229	-1.150958	2.039260	3.891349	-1.528432	2.612236
H	2.040523	3.439072	3.107347	1.993278	3.474756	3.136819	6.793724	-2.170968	-0.364174	6.499713	-2.519492	0.006001
H	2.798174	2.224279	4.157296	2.768602	2.261346	4.175460	6.521854	-1.396842	1.210288	6.344645	-1.820126	1.630485
H	1.078332	2.084541	3.715378	1.047903	2.107092	3.741593	6.216832	-0.498986	-0.296454	5.992198	-0.833902	0.190835
H	1.491957	1.122412	0.467636	1.454035	1.170420	0.485112	3.352808	-2.230533	-1.183058	3.018283	-2.409802	-0.616952
H	4.611728	1.892204	2.298489	4.575711	1.957553	2.305455	5.002801	-3.471340	1.758030	4.779349	-3.864690	2.158369
H	4.077597	1.471844	0.660029	4.037071	1.542870	0.667021	3.602562	-3.791681	0.721165	3.312287	-4.079284	1.188844
H	3.762300	3.098292	1.304051	3.709616	3.161936	1.323394	5.245178	-4.094798	0.110058	4.905407	-4.409294	0.469958
H	-2.670984	1.661647	-0.238877	-2.714995	1.678718	-0.239421	-1.569206	2.801013	-1.591114	-3.626642	2.722962	0.329097
H	-0.984779	1.451468	0.221787	-1.034890	1.478062	0.248937	-3.327693	2.808434	-1.647839	-1.985964	2.531509	0.936937
H	-0.266253	1.662687	-2.189977	-0.269532	1.718478	-2.138859	-1.589476	2.431391	0.886037	-1.041938	2.886980	-1.369697
H	-0.246921	3.111290	-3.958652	-0.236921	3.165867	-3.908197	-2.293273	3.307816	2.880256	-0.908852	4.403677	-3.079178
H	-1.859188	3.432892	-4.629261	-1.838604	3.449371	-4.620359	-3.263474	4.783720	2.702524	-2.456880	4.670385	-3.906061
H	-1.073673	4.645331	-3.623025	-1.106878	4.681184	-3.597265	-4.055460	3.259907	3.088521	-1.843711	5.884869	-2.789249
H	-3.669551	3.094827	-1.287590	-3.724448	3.078197	-1.323641	-4.826519	3.737176	-0.619906	-4.584053	4.139253	-0.781797
H	-3.126678	4.626484	-1.986662	-3.201597	4.618691	-2.019412	-5.571901	3.511521	0.968871	-4.053458	5.714338	-1.390084
H	-3.910395	3.428558	-3.009521	-3.931131	3.395182	-3.052400	-4.781136	5.034805	0.582818	-4.664359	4.503852	-2.511001
H	-3.910962	-0.182197	-1.378643	-3.938741	-0.187271	-1.382703	-4.540818	0.706241	-1.857161	-4.806355	0.785582	-0.612325

	**3a-5**			**3a-6**			**3a-7**			**3a-8**		
**Element**	**X**	**Y**	**Z**	**X**	**Y**	**Z**	**X**	**Y**	**Z**	**X**	**Y**	**Z**
O	-6.202298	-1.202424	-1.298084	-5.723665	-1.735518	-2.020352	-4.761720	-3.211064	-2.442480	-4.767575	-3.902878	-2.173290
C	-5.134946	-2.018908	-1.075691	-4.718810	-2.649199	-1.926346	-4.882237	-1.862547	-2.287734	-4.744407	-2.558170	-1.952830
C	-3.880151	-1.284193	-0.805150	-3.420246	-2.055850	-1.538734	-3.614991	-1.206434	-1.898948	-3.409210	-2.057365	-1.558946
C	-2.719567	-2.040532	-0.558405	-2.317836	-2.919937	-1.412170	-2.421311	-1.930880	-1.723492	-2.299080	-2.911584	-1.429068
C	-1.534600	-1.375459	-0.299631	-1.097614	-2.386271	-1.040902	-1.276258	-1.240986	-1.361794	-1.087452	-2.362475	-1.046249
C	-0.139226	-1.875251	-0.007468	0.247265	-3.035243	-0.813693	0.141203	-1.702680	-1.116888	0.262503	-2.998013	-0.810598
C	0.651005	-0.556538	0.228373	1.136607	-1.836319	-0.379879	0.860778	-0.385744	-0.708099	1.137743	-1.789628	-0.374198
C	1.262893	-0.390062	1.638534	1.788926	-1.956230	1.013167	1.461928	-0.370960	0.716383	1.793985	-1.905647	1.017176
C	0.208161	-0.387722	2.744663	2.822815	-3.089599	0.996922	0.410205	-0.575441	1.806417	2.838741	-3.028979	0.997173
C	2.190404	0.846155	1.694778	0.777788	-2.083376	2.183510	2.317859	0.897480	0.940612	0.786112	-2.044398	2.189012

C	3.574131	0.278216	1.354061	0.711109	-0.676020	2.793908	3.732215	0.457391	0.541050	0.707502	-0.638852	2.802249
C	3.513787	-1.122382	1.969843	2.086097	-0.081051	2.464872	3.750709	-1.012934	0.967713	2.075845	-0.029909	2.471174
C	4.393719	-2.205496	1.303397	2.110058	1.445594	2.221863	4.691494	-1.948617	0.173412	2.083840	1.497301	2.230481
C	5.858343	-1.769130	1.253473	1.637195	2.202961	3.464513	6.129101	-1.428001	0.192029	1.607271	2.247768	3.475920
O	3.974467	-2.364651	-0.056433	1.182080	1.760358	1.177164	4.282414	-1.953072	-1.198768	1.149467	1.804694	1.189460
C	4.247251	-3.541046	2.051081	3.520418	1.899499	1.811591	4.620821	-3.376271	0.740163	3.488350	1.965950	1.816689
O	2.137008	-1.532374	1.818488	2.488687	-0.715812	1.236394	2.399725	-1.476142	0.751120	2.482045	-0.658967	1.240838
O	-0.281899	0.541214	-0.016343	0.282755	-0.650950	-0.419667	-0.127735	0.684088	-0.817790	0.269596	-0.615069	-0.408589
C	-1.513114	0.021665	-0.288051	-0.976997	-1.013456	-0.801042	-1.327038	0.142493	-1.175118	-0.983565	-0.989974	-0.799956
C	-2.640905	0.813351	-0.526380	-2.041093	-0.114607	-0.930313	-2.489265	0.902495	-1.346504	-2.057001	-0.101775	-0.932404
C	-2.563085	2.329356	-0.518104	-1.886161	1.385290	-0.742344	-2.498468	2.406282	-1.136339	-1.920800	1.398865	-0.736482
C	-1.936325	2.880209	-1.779717	-1.767865	2.100090	-2.073136	-2.485834	2.790741	0.326433	-1.812781	2.121490	-2.063887
C	-2.478704	3.706802	-2.686209	-0.882988	3.033403	-2.454484	-3.432694	3.434226	1.025537	-0.936859	3.064067	-2.443062
C	-1.684583	4.144784	-3.894983	-0.963013	3.632291	-3.840328	-3.234619	3.727041	2.494736	-1.026602	3.669712	-3.825377
C	-3.874544	4.277350	-2.620886	0.230399	3.589005	-1.601615	-4.746936	3.922932	0.467177	0.174698	3.624522	-1.590952
C	-3.825469	0.119524	-0.788079	-3.266627	-0.681153	-1.299417	-3.629949	0.185466	-1.710993	-3.270554	-0.682708	-1.311868
O	-5.246408	-3.230689	-1.105536	-4.911372	-3.830627	-2.147638	-5.945777	-1.297153	-2.461981	-5.744951	-1.877524	-2.078881
H	-6.952874	-1.802342	-1.461773	-6.512130	-2.246949	-2.278931	-5.651088	-3.519700	-2.695078	-5.687818	-4.104953	-2.423130
H	-2.784318	-3.123765	-0.576666	-2.453325	-3.979479	-1.604360	-2.410589	-3.004852	-1.874811	-2.403321	-3.973621	-1.623081
H	-0.093517	-2.535004	0.864809	0.202519	-3.811647	-0.041116	0.216132	-2.463626	-0.333465	0.221162	-3.772934	-0.036113
H	0.275433	-2.436784	-0.852923	0.636187	-3.510986	-1.720851	0.589896	-2.131930	-2.020549	0.662002	-3.472025	-1.714123
H	1.454299	-0.444366	-0.506504	1.936392	-1.646182	-1.102767	1.658364	-0.139264	-1.416114	1.933967	-1.587999	-1.098024
H	-0.413590	-1.287844	2.718152	3.552039	-2.940318	0.193239	-0.154746	-1.501033	1.659659	3.564661	-2.871729	0.192012
H	0.702137	-0.350230	3.720693	3.365761	-3.101768	1.947106	0.902475	-0.634905	2.782260	3.383993	-3.036972	1.946078
H	-0.444006	0.486200	2.651284	2.352019	-4.069818	0.861804	-0.296233	0.260288	1.823789	2.377120	-4.013594	0.862036
H	2.188356	1.264370	2.708130	1.157523	-2.805411	2.915317	2.290791	1.179812	1.999541	1.173559	-2.764505	2.918703
H	1.857751	1.633979	1.012923	-0.203146	-2.440874	1.856826	1.942638	1.747443	0.363568	-0.192134	-2.409948	1.863069
H	3.716987	0.191320	0.271688	-0.064580	-0.077805	2.309976	3.881360	0.521309	-0.542046	-0.074982	-0.047245	2.321202
H	4.390928	0.882344	1.759543	0.513248	-0.695807	3.869659	4.512721	1.048120	1.029300	0.512341	-0.662587	3.878406
H	3.752781	-1.078083	3.044345	2.818090	-0.329572	3.249903	3.984866	-1.096608	2.040920	2.812078	-0.272381	3.254109
H	6.464387	-2.562448	0.804482	1.651098	3.279324	3.264931	6.779185	-2.120859	-0.351646	1.609542	3.324514	3.278019
H	6.243648	-1.569597	2.259705	2.291222	2.004550	4.321140	6.502228	-1.340035	1.218592	2.266012	2.054641	4.330141
H	5.978483	-0.867910	0.645133	0.613165	1.923644	3.729905	6.198349	-0.449910	-0.292955	0.586959	1.957666	3.744024
H	3.020381	-2.545995	-0.003856	1.277452	1.068897	0.496487	3.338866	-2.188006	-1.176463	1.250265	1.116444	0.506417
H	4.618898	-3.466134	3.080077	4.249807	1.709452	2.608638	4.986820	-3.415043	1.773183	4.222098	1.781731	2.611106
H	3.196718	-3.846429	2.094669	3.848810	1.366754	0.914779	3.589349	-3.743489	0.735056	3.819247	1.438196	0.917839
H	4.816523	-4.318365	1.531572	3.511828	2.973032	1.597613	5.233890	-4.046005	0.128767	3.468349	3.039722	1.604737
H	-3.566219	2.735311	-0.361022	-2.782839	1.753744	-0.221405	-1.607281	2.832120	-1.618607	-2.821920	1.752970	-0.213603
H	-1.955767	2.643912	0.342735	-1.038509	1.601553	-0.088191	-3.366554	2.827808	-1.650335	-1.075387	1.622255	-0.081841
H	-0.915547	2.538505	-1.951091	-2.525929	1.802973	-2.799687	-1.582202	2.488953	0.856356	-2.571219	1.822205	-2.789017
H	-0.680201	3.710058	-3.903737	-1.787432	3.210868	-4.424281	-2.270983	3.357465	2.859424	-1.849329	3.244346	-4.408786
H	-2.189763	3.852891	-4.826620	-1.101731	4.721758	-3.792555	-3.282159	4.807083	2.693699	-1.174705	4.757635	-3.771177
H	-1.582429	5.238835	-3.927373	-0.030125	3.463810	-4.396929	-4.027407	3.265159	3.100068	-0.094082	3.512440	-4.385947
H	-4.440359	3.949781	-1.745668	0.331107	3.113900	-0.624276	-4.877278	3.708067	-0.595814	0.280651	3.147345	-0.615177
H	-3.843760	5.375810	-2.607098	1.193818	3.492142	-2.122253	-5.588064	3.464370	1.005327	1.137691	3.535920	-2.113930
H	-4.448339	3.995560	-3.514714	0.080955	4.666058	-1.437359	-4.846534	5.008679	0.604628	0.018295	4.699996	-1.422952
H	-4.733260	0.681902	-0.980995	-4.131206	-0.033122	-1.404208	-4.570428	0.706880	-1.862108	-4.152219	-0.058119	-1.424375

	3a-9			3a-10			3a-11			3a-12		
**Element**	**X**	**Y**	**Z**	**X**	**Y**	**Z**	**X**	**Y**	**Z**	**X**	**Y**	**Z**
O	-6.343470	-1.118175	-2.410954	-5.152706	-3.124146	-2.584697	-6.615015	-0.977334	-1.442764	-5.521243	-3.027400	-1.656706
C	-5.221421	-1.889036	-2.361759	-5.299881	-1.796573	-2.314750	-5.530605	-1.801374	-1.418317	-5.596728	-1.693681	-1.387276
C	-4.014101	-1.141946	-1.948258	-4.034671	-1.139442	-1.921808	-4.277376	-1.110843	-1.044947	-4.291355	-1.098166	-1.028946
C	-2.800633	-1.849529	-1.870344	-2.817966	-1.842493	-1.847812	-3.098949	-1.877134	-0.989277	-3.110289	-1.861203	-0.976764
C	-1.656986	-1.170630	-1.490039	-1.676183	-1.152512	-1.475948	-1.915152	-1.253806	-0.637444	-1.928541	-1.229038	-0.627372
C	-0.224531	-1.620960	-1.327087	-0.240597	-1.595160	-1.318354	-0.506605	-1.778224	-0.487698	-0.517366	-1.747631	-0.480895
C	0.477814	-0.333142	-0.811454	0.457780	-0.301648	-0.811409	0.276934	-0.516656	-0.026145	0.262107	-0.482309	-0.022469
C	1.070642	-0.428768	0.614123	1.056653	-0.388194	0.612243	0.948661	-0.621982	1.362480	0.938666	-0.584121	1.364053
C	0.006203	-0.699847	1.676892	-0.002462	-0.658644	1.680452	-0.057951	-0.848445	2.490079	-0.063514	-0.813338	2.495004
C	1.935866	0.811629	0.936767	1.917885	0.857322	0.925640	1.874292	0.589433	1.622054	1.860549	0.630971	1.619709
C	3.349444	0.390025	0.515512	3.331724	0.439496	0.501469	3.244391	0.114367	1.121288	3.231068	0.160375	1.115843
C	3.352376	-1.107361	0.834916	3.342000	-1.056202	0.828437	3.213858	-1.380093	1.452816	3.206924	-1.333748	1.449283
C	4.292464	-1.991201	-0.017507	4.283061	-1.940659	-0.022265	4.068010	-2.302635	0.552349	4.062502	-2.254442	0.548280
C	5.734483	-1.487532	0.053216	5.723035	-1.430156	0.040090	5.528401	-1.849937	0.529786	5.520965	-1.795924	0.520858
O	3.897447	-1.890123	-1.390039	3.882200	-1.849553	-1.393818	3.593139	-2.197059	-0.794279	3.583256	-2.153514	-0.797166
C	4.202946	-3.455953	0.441560	4.201881	-3.403128	0.445678	3.955842	-3.760066	1.029348	3.957538	-3.711443	1.028355
O	1.999908	-1.541370	0.574547	1.990547	-1.497177	0.574191	1.833250	-1.767416	1.279238	1.827352	-1.726435	1.278870
O	-0.524842	0.729127	-0.836463	-0.549739	0.755340	-0.837255	-0.678541	0.588460	-0.009027	-0.697901	0.618202	-0.002886
C	-1.726187	0.192159	-1.187261	-1.750394	0.210776	-1.179171	-1.909037	0.113821	-0.349600	-1.927388	0.138176	-0.340118
C	-2.910545	0.934468	-1.250238	-2.939159	0.947461	-1.239086	-3.056135	0.914088	-0.392870	-3.078379	0.934101	-0.380556
C	-2.944639	2.405290	-0.882042	-2.979732	2.419614	-0.877337	-2.996716	2.401899	-0.101912	-3.025594	2.422276	-0.090677
C	-2.975277	2.624221	0.614482	-3.005767	2.644203	0.618446	-2.521983	3.200343	-1.295818	-2.559788	3.221781	-1.287406
C	-2.112497	3.315860	1.374990	-2.143011	3.342300	1.373068	-1.418887	3.954922	-1.410230	-1.460154	3.980486	-1.407664
C	-2.333634	3.442013	2.864674	-2.360043	3.474600	2.862838	-1.119760	4.685020	-2.698736	-1.170790	4.711857	-2.697692

C	-0.887799	4.033415	0.862440	-0.922780	4.062304	0.853275	-0.392414	4.161119	-0.323774	-0.428846	4.190860	-0.326567
C	-4.051559	0.228731	-1.641657	-4.075285	0.232111	-1.620757	-4.240069	0.262031	-0.748119	-4.257102	0.274567	-0.732331
O	-5.253427	-3.073996	-2.638549	-6.383130	-1.248502	-2.400883	-5.627568	-2.985196	-1.684694	-6.653843	-1.093662	-1.447900
H	-7.055549	-1.721325	-2.692401	-6.043731	-3.435759	-2.827621	-7.363099	-1.547238	-1.698953	-6.432251	-3.295586	-1.876192
H	-2.792323	-2.907124	-2.114083	-2.787330	-2.900863	-2.083197	-3.149292	-2.936352	-1.220600	-3.138311	-2.921296	-1.204576
H	-0.111738	-2.450466	-0.621990	-0.119535	-2.420969	-0.610161	-0.427858	-2.592018	0.240240	-0.432788	-2.560231	0.247861
H	0.206717	-1.951083	-2.279712	0.188173	-1.926846	-2.271669	-0.112624	-2.161154	-1.436417	-0.123598	-2.129713	-1.430149
H	1.274160	-0.018441	-1.493064	1.250064	0.013444	-1.497581	1.045259	-0.244588	-0.756821	1.027037	-0.207550	-0.755738
H	-0.566283	-1.606445	1.457429	-0.572019	-1.568664	1.467525	-0.678495	-1.732049	2.312001	-0.681424	-1.699351	2.319606
H	0.488137	-0.835852	2.650355	0.483788	-0.787954	2.652679	0.477013	-0.999059	3.433045	0.474930	-0.961185	3.436425
H	-0.690270	0.141757	1.744831	-0.702234	0.180303	1.747059	-0.715468	0.019769	2.597811	-0.723890	0.052509	2.604020
H	1.903404	1.015071	2.013605	1.888388	1.066023	2.001541	1.913438	0.802003	2.696836	1.901790	0.845024	2.694105
H	1.571517	1.704985	0.421663	1.547981	1.746593	0.407469	1.512272	1.491648	1.120751	1.493858	1.531233	1.118315
H	3.506497	0.530144	-0.559306	3.484438	0.574897	-0.574584	3.341945	0.241825	0.037915	3.325298	0.286951	0.032066
H	4.131517	0.937159	1.049938	4.113364	0.992573	1.030369	4.075672	0.636673	1.603716	4.061666	0.686350	1.595437
H	3.576858	-1.270188	1.901137	3.570270	-1.212630	1.894797	3.497161	-1.544121	2.504710	3.492926	-1.495366	2.500805
H	6.383883	-2.144860	-0.533691	6.373154	-2.087782	-0.545700	6.116870	-2.534053	-0.089823	6.110427	-2.478583	-0.099427
H	6.097752	-1.478955	1.087036	6.090257	-1.414254	1.072423	5.954896	-1.846885	1.539191	5.950346	-1.789679	1.529019
H	5.817697	-0.477158	-0.357824	5.800086	-0.421711	-0.376810	5.621307	-0.846084	0.105071	5.608598	-0.792348	0.094406
H	2.951830	-2.117696	-1.394671	2.936531	-2.077055	-1.392269	2.642274	-2.394120	-0.739804	2.632477	-2.349868	-0.738380
H	4.558237	-3.574475	1.472244	4.561672	-3.514097	1.475644	4.369423	-3.883898	2.037412	4.374484	-3.831775	2.035456
H	3.168256	-3.811465	0.399849	3.168606	-3.763409	0.410007	2.908798	-4.079166	1.054168	2.911820	-4.034603	1.056716
H	4.815782	-4.084876	-0.211956	4.815004	-4.033104	-0.206564	4.505176	-4.414729	0.345367	4.507528	-4.365181	0.344007
H	-2.090096	2.916071	-1.335527	-2.129713	2.932991	-1.336386	-4.005509	2.738225	0.174605	-4.035123	2.753088	0.189001
H	-3.847470	2.848268	-1.325363	-3.886914	2.855127	-1.318677	-2.357062	2.579144	0.767727	-2.383378	2.603449	0.776280
H	-3.822126	2.151033	1.113631	-3.849548	2.170438	1.122110	-3.175675	3.125592	-2.165971	-3.218124	3.144612	-2.153766
H	-3.234900	2.913811	3.191187	-3.258122	2.944246	3.194556	-1.891983	4.514002	-3.455247	-1.946305	4.537653	-3.450072
H	-1.479648	3.039812	3.427825	-1.502624	3.078799	3.425370	-1.041802	5.768625	-2.531072	-1.096602	5.795784	-2.530371
H	-2.431204	4.495899	3.161657	-2.461269	4.529481	3.155035	-0.155206	4.366167	-3.118568	-0.207066	4.397167	-3.122629
H	-0.692836	3.855289	-0.196985	-0.730202	3.879431	-0.205790	-0.590731	3.575818	0.576145	-0.620048	3.604573	0.574268
H	-0.981594	5.118149	1.013498	-1.020724	5.147423	0.998850	0.607232	3.884733	-0.686753	0.570129	3.918860	-0.694772
H	0.003248	3.719278	1.422988	-0.028739	3.754964	1.412885	-0.336204	5.220858	-0.037515	-0.375668	5.250770	-0.040342
H	-4.997503	0.756224	-1.712781	-5.033116	0.739001	-1.694038	-5.161971	0.833209	-0.792584	-5.191229	0.826782	-0.779518

		3a-13			3a-14			3a-15			3a-16	
**Element**	**X**	**Y**	**Z**	**X**	**Y**	**Z**	**X**	**Y**	**Z**	**X**	**Y**	**Z**
O	-4.932768	-1.868458	-3.662460	-5.014910	-1.663193	-3.852867	-3.970221	-3.983403	-3.454165	-4.351157	-3.836173	-3.437805
C	-3.959061	-2.747301	-3.296848	-3.941889	-2.491803	-3.728821	-4.004689	-2.628114	-3.315354	-4.364736	-2.497385	-3.184644
C	-2.792227	-2.098998	-2.659288	-2.827874	-1.898658	-2.956256	-2.794793	-2.079983	-2.664212	-3.122139	-2.015579	-2.541338
C	-1.730484	-2.922593	-2.246831	-1.671025	-2.678862	-2.772825	-1.733284	-2.901878	-2.246620	-2.054753	-2.881053	-2.235969
C	-0.626675	-2.332769	-1.658291	-0.613110	-2.134545	-2.070678	-0.632859	-2.304139	-1.655794	-0.921497	-2.343665	-1.652916
C	0.644684	-2.922551	-1.095209	0.729077	-2.701000	-1.668672	0.637600	-2.889508	-1.085739	0.361091	-2.981603	-1.170894
C	1.491383	-1.656149	-0.808759	1.470863	-1.433254	-1.183456	1.481053	-1.620334	-0.801788	1.253953	-1.742241	-0.926050
C	2.250591	-1.624846	0.526839	2.385179	-1.567125	0.042800	2.237278	-1.582609	0.535390	2.184506	-1.763955	0.295183
C	3.272475	-2.768840	0.559592	3.484018	-2.597266	-0.244438	3.260526	-2.725200	0.575347	3.153019	-2.947515	0.183367
C	1.337015	-1.601715	1.781049	1.634290	-1.839262	1.371406	1.320763	-1.555276	1.787358	1.448552	-1.712437	1.658589
C	1.352828	-0.137462	2.244084	1.554473	-0.466516	2.056148	1.333500	-0.088952	2.243924	1.543079	-0.241554	2.091282
C	2.710027	0.373819	1.746314	2.796429	0.261104	1.526463	2.691282	0.421867	1.747269	2.841262	0.235413	1.428198
C	2.774344	1.871430	1.369012	2.673848	1.795501	1.379428	2.754650	1.917924	1.363798	2.884868	1.724532	1.013975
C	2.425869	2.751880	2.570961	2.373616	2.449038	2.729916	2.403354	2.802780	2.561706	2.701693	2.633108	2.231382
O	1.788882	2.143009	0.367226	1.566205	2.098066	0.523076	1.770509	2.184624	0.359576	1.791085	1.998624	0.130844
C	4.169308	2.222927	0.824755	3.962061	2.374976	0.770811	4.150189	2.268581	0.820428	4.209546	2.037699	0.297803
O	2.970127	-0.374518	0.544285	3.008044	-0.274471	0.206751	2.955220	-0.331509	0.549099	2.951669	-0.542806	0.221645
O	0.568291	-0.523900	-0.877973	0.431378	-0.443965	-0.906772	0.555856	-0.490798	-0.877175	0.340235	-0.607335	-0.818829
C	-0.587043	-0.947356	-1.472680	-0.709268	-0.837332	-1.551306	-0.596749	-0.918627	-1.474231	-0.856511	-0.973748	-1.371288
C	-1.618475	-0.088867	-1.870599	-1.846608	-0.037434	-1.686531	-1.629313	-0.062990	-1.877661	-1.901759	-0.083843	-1.634025
C	-1.545758	1.418414	-1.690000	-1.949242	1.353808	-1.081906	-1.562034	1.444759	-1.700502	-1.830034	1.394578	-1.286480
C	-1.633397	1.886231	-0.248652	-3.031800	1.445205	-0.027394	-1.654378	1.914974	-0.260123	-2.871158	1.796586	-0.263596
C	-2.746756	2.195304	0.433330	-2.964656	1.995874	1.193836	-2.771311	2.215742	0.419789	-2.711491	2.532937	0.846094
C	-2.664756	2.661684	1.867910	-4.182237	2.008400	2.089606	-2.694803	2.686541	1.853247	-3.899417	2.847344	1.726467
C	-4.147864	2.115093	-0.121130	-1.745395	2.654595	1.790643	-4.170867	2.121899	-0.136272	-1.408665	3.126470	1.322671
C	-2.720207	-0.709143	-2.468801	-2.899351	-0.606351	-2.414697	-2.724669	-0.689911	-2.476402	-3.030505	-0.649359	-2.236759
O	-4.076747	-3.942969	-3.492490	-3.938809	-3.605556	-4.219783	-4.955841	-1.975155	-3.702042	-5.323275	-1.806510	-3.474618
H	-5.633118	-2.414636	-4.064077	-5.667908	-2.165171	-4.374133	-4.810609	-4.217056	-3.889186	-5.211610	-4.026484	-3.854371
H	-1.801064	-3.994023	-2.404586	-1.637677	-3.679876	-3.190684	-1.784088	-3.975266	-2.393615	-2.128942	-3.939130	-2.462316
H	0.445975	-3.495501	-0.179869	0.618925	-3.441433	-0.864437	0.437083	-3.458699	-0.168333	0.194529	-3.552240	-0.246785
H	1.155030	-3.594789	-1.792016	1.263382	-3.188761	-2.489451	1.152272	-3.563737	-1.777580	0.813045	-3.663457	-1.897610
H	2.223834	-1.495089	-1.609313	2.076357	-1.017950	-1.999527	2.215070	-1.460938	-1.601283	1.876962	-1.549000	-1.809018
H	3.926300	-2.734935	-0.318758	4.014571	-2.356454	-1.172197	3.916271	-2.694249	-0.301690	3.678934	-2.934476	-0.777609
H	3.900204	-2.672426	1.450971	4.212879	-2.590940	0.571904	3.886187	-2.624121	1.467657	3.902282	-2.882516	0.978388
H	2.782549	-3.748563	0.592335	3.074663	-3.610248	-0.329596	2.771806	-3.705444	0.611336	2.631416	-3.906211	0.282638
H	1.756637	-2.258944	2.551123	2.213076	-2.544115	1.979311	1.739552	-2.208529	2.561287	1.963244	-2.362641	2.375326
H	0.324170	-1.955821	1.568359	0.646614	-2.281244	1.210850	0.308848	-1.911596	1.573863	0.413282	-2.058826	1.588621
H	0.551796	0.425910	1.760029	0.657822	0.070612	1.738895	0.532914	0.471196	1.755420	0.702369	0.331634	1.694073

H	1.242754	-0.040063	3.328201	1.550233	-0.542799	3.147518	1.220647	0.013048	3.327324	1.564030	-0.125833	3.178974
H	3.500775	0.155990	2.482393	3.676202	0.032555	2.149333	3.480582	0.208173	2.486112	3.709024	0.019652	2.072112
H	2.466418	3.805686	2.277172	2.278820	3.531960	2.600083	2.443412	3.855427	2.263772	2.723462	3.680501	1.913538
H	3.133501	2.597118	3.393367	3.179431	2.259129	3.448018	3.109715	2.651927	3.385937	3.503723	2.479913	2.962383
H	1.414439	2.542218	2.932304	1.433641	2.076504	3.147698	1.391462	2.593652	2.922014	1.739813	2.449347	2.719116
H	1.806425	1.388576	-0.249314	1.583546	1.440776	-0.195532	1.789902	1.428431	-0.254683	1.713377	1.229778	-0.462044
H	4.946508	2.068931	1.583477	4.827419	2.199797	1.421904	4.926321	2.118274	1.580994	5.070276	1.880217	0.959473
H	4.410648	1.600476	-0.041592	4.167670	1.915757	-0.200266	4.393535	1.643042	-0.043139	4.332179	1.396221	-0.579481
H	4.187606	3.273110	0.516506	3.846342	3.454283	0.629466	4.167951	3.317567	0.508141	4.209803	3.082049	-0.030193
H	-0.595701	1.777823	-2.106125	-0.979142	1.653257	-0.680458	-0.612389	1.806458	-2.115580	-0.823057	1.644532	-0.946782
H	-2.338695	1.869303	-2.296121	-2.182301	2.062667	-1.892058	-2.355519	1.890999	-2.309078	-1.998921	1.970991	-2.209590
H	-0.674521	1.987716	0.256023	-3.987909	1.015179	-0.328769	-0.696850	2.025947	0.245152	-3.878587	1.444042	-0.488152
H	-1.630353	2.705073	2.222629	-5.048064	1.536049	1.614716	-1.661231	2.739743	2.209183	-4.825957	2.411976	1.338986
H	-3.106732	3.661450	1.984965	-3.982551	1.482664	3.034152	-3.145511	3.682764	1.967065	-3.745415	2.468741	2.746995
H	-3.228385	1.994071	2.535522	-4.461985	3.036207	2.361284	-3.253688	2.015978	2.521893	-4.046221	3.932897	1.818533
H	-4.179429	1.848818	-1.180285	-0.836813	2.544787	1.195308	-4.199523	1.850702	-1.194142	-0.533915	2.813094	0.749399
H	-4.738861	1.366353	0.425051	-1.925658	3.729695	1.936190	-4.756657	1.370831	0.412223	-1.456224	4.224763	1.291564
H	-4.671267	3.073334	0.002878	-1.542435	2.242922	2.789693	-4.702029	3.076428	-0.016811	-1.232572	2.860085	2.374797
H	-3.546145	-0.093575	-2.809595	-3.802459	-0.025864	-2.573709	-3.560364	-0.092020	-2.827231	-3.877792	-0.020157	-2.493259

	3a-17			3a-18		
**Element**	**X**	**Y**	**Z**	**X**	**Y**	**Z**
O	-5.946483	-1.185391	-2.369121	-4.800355	-3.265260	-2.328078
C	-4.852538	-1.982213	-2.214753	-4.923189	-1.917472	-2.169159
C	-3.642141	-1.242817	-1.795490	-3.658420	-1.261220	-1.772344
C	-2.456378	-1.978359	-1.613797	-2.464740	-1.985023	-1.593533
C	-1.309953	-1.308133	-1.226782	-1.321320	-1.295199	-1.226484
C	0.095724	-1.788791	-0.953353	0.092172	-1.759000	-0.963715
C	0.842610	-0.469644	-0.609813	0.826853	-0.430629	-0.628736
C	1.565655	-0.441164	0.754120	1.539498	-0.384501	0.740447
C	0.609348	-0.604792	1.935639	0.575849	-0.547618	1.916056
C	2.458777	0.819403	0.882615	2.420014	0.885150	0.865315
C	3.843247	0.338485	0.420941	3.812218	0.413512	0.417445
C	3.845145	-1.112612	0.906978	3.824090	-1.033694	0.914756
C	4.775891	-2.140966	0.231693	4.768574	-2.058644	0.253571
C	4.558758	-2.244643	-1.285679	4.563521	-2.174855	-1.264592
O	4.504020	-3.413986	0.835409	4.502781	-3.329835	0.864099
C	6.237336	-1.830434	0.559359	6.224855	-1.733428	0.589716
O	2.485974	-1.558611	0.721450	2.470341	-1.493750	0.723783
O	-0.153176	0.599573	-0.639016	-0.176534	0.630261	-0.675316
C	-1.352230	0.073561	-1.020146	-1.374402	0.088006	-1.037371
C	-2.505414	0.845128	-1.194197	-2.536933	0.847063	-1.209948
C	-2.503399	2.345131	-0.958937	-2.548353	2.350045	-0.995168
C	-2.566209	2.702133	0.509282	-2.577088	2.727505	0.469085
C	-3.518121	3.387104	1.160400	-3.531960	3.389430	1.139431
C	-3.400341	3.640391	2.645483	-3.377394	3.669682	2.616278
C	-4.761849	3.964407	0.529549	-4.814447	3.912340	0.539686
C	-3.650795	0.145989	-1.585122	-3.675873	0.129909	-1.579934
O	-4.908911	-3.181947	-2.412549	-5.986552	-1.352660	-2.346278
H	-6.663664	-1.786535	-2.641808	-5.688347	-3.574245	-2.585067
H	-2.471287	-3.050009	-1.784955	-2.452794	-3.058787	-1.746306
H	0.141990	-2.507976	-0.128872	0.155587	-2.476106	-0.138575
H	0.545382	-2.278610	-1.823591	0.539988	-2.244478	-1.837583
H	1.573691	-0.227796	-1.388793	1.561977	-0.190531	-1.404256
H	0.011181	-1.517398	1.852213	-0.012713	-1.466744	1.835726
H	1.183507	-0.664562	2.865669	1.143410	-0.593970	2.850897
H	-0.072245	0.248931	2.001323	-0.114695	0.299715	1.969535
H	2.499365	1.140367	1.929673	2.449984	1.214778	1.910025
H	2.066604	1.655826	0.297179	2.024456	1.713227	0.270397
H	3.929239	0.384019	-0.670274	3.905821	0.451571	-0.673419
H	4.660724	0.927178	0.847517	4.621049	1.013028	0.845404
H	4.073181	-1.141143	1.984378	4.045151	-1.051848	1.993830
H	5.157055	-3.074504	-1.673711	5.171399	-3.002559	-1.642215
H	4.857817	-1.332248	-1.813958	4.859085	-1.263852	-1.797202
H	3.506491	-2.444720	-1.512045	3.514578	-2.385063	-1.497164
H	3.538292	-3.521993	0.805417	3.537939	-3.444361	0.830303
H	6.559632	-0.890240	0.099212	6.542499	-0.793611	0.125578
H	6.384739	-1.763708	1.642511	6.363690	-1.658140	1.673430
H	6.874754	-2.636304	0.182004	6.871970	-2.536260	0.222504
H	-1.580062	2.763563	-1.383684	-1.642320	2.774701	-1.450028
H	-3.334928	2.790964	-1.511775	-3.400280	2.776237	-1.531869
H	-1.718198	2.338168	1.089908	-1.699583	2.401555	1.028045
H	-2.486407	3.206607	3.063317	-2.435990	3.275046	3.011451
H	-3.397059	4.717478	2.865116	-3.406005	4.749487	2.820126

H	-4.257005	3.215974	3.187947	-4.201241	3.223722	3.191283
H	-4.837831	3.771442	-0.542977	-4.915067	3.703464	-0.527728
H	-5.662296	3.554402	1.007655	-5.684127	3.474135	1.048574
H	-4.801648	5.052657	0.677290	-4.891022	4.999976	0.677064
H	-4.575561	0.693370	-1.736046	-4.616191	0.650939	-1.733389

Table S3.6. Calculated conformational analysis of the **3b** at B3LYP/6-31g(d) level

conformer	3D conformer	G (Hartree)	ΔG (kcal/mol)	Population
3b-1	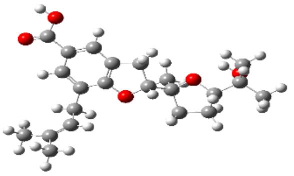	-1232.116207	0	12.004%
3b-2	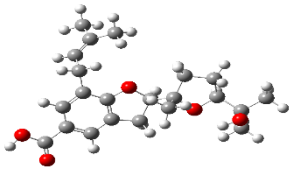	-1232.116138	0.04329819	11.158%
3b-3	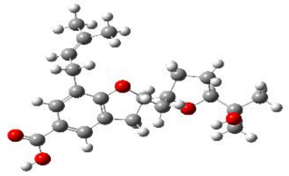	-1232.116119	0.05522088	10.936%
3b-4	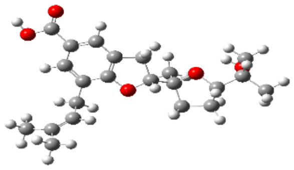	-1232.116111	0.06024096	10.844%
3b-5	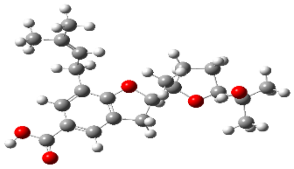	-1232.116006	0.12612951	9.702%
3b-6	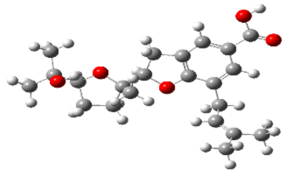	-1232.11599	0.13616967	9.539%
3b-7	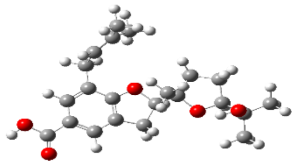	-1232.115613	0.37274094	6.399%
3b-8	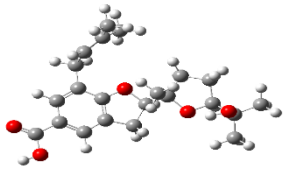	-1232.115562	0.40474395	6.062%

3b-9	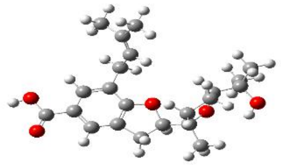	-1232.114958	0.78375999	3.197%
3b-10	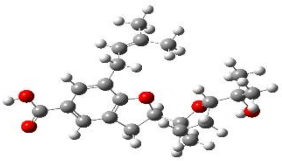	-1232.114851	0.85090356	2.855%
3b-11	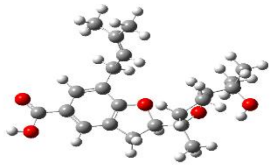	-1232.114834	0.86157123	2.804%
3b-12	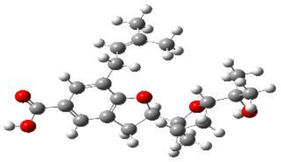	-1232.114768	0.90298689	2.614%
3b-13	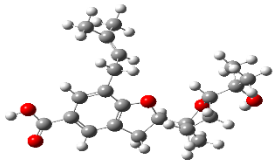	-1232.114695	0.94879512	2.420%
3b-14	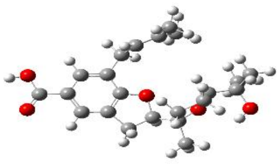	-1232.114439	1.10943768	1.845%
3b-15	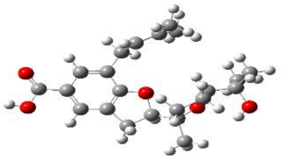	-1232.114429	1.11571278	1.826%
3b-16	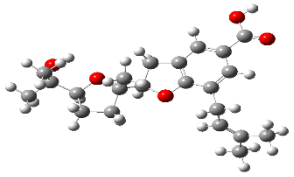	-1232.113956	1.41252501	1.106%

Table S3.7. Cartesian coordinates of low-energy conformers of **3b**

	**3b-1**			**3b-2**			**3b-3**			**3b-4**		
**Element**	**X**	**Y**	**Z**	**X**	**Y**	**Z**	**X**	**Y**	**Z**	**X**	**Y**	**Z**
O	-5.323643	-3.249525	1.028200	-5.609844	-0.812871	4.049896	-4.816241	-2.943820	3.516552	-6.272301	-1.575088	1.185615
C	-5.349761	-1.887634	1.070600	-4.771858	-1.695090	3.437357	-4.817587	-1.581793	3.470761	-5.148225	-2.336212	1.072774
C	-4.040211	-1.271171	0.766357	-3.732038	-1.041460	2.613746	-3.739131	-1.027318	2.624295	-3.938374	-1.551424	0.744631
C	-2.900133	-2.043429	0.475459	-2.812506	-1.868056	1.942642	-2.817136	-1.851143	1.952503	-2.722596	-2.247591	0.613481
C	-1.709932	-1.389926	0.202391	-1.828601	-1.279542	1.168385	-1.833965	-1.253749	1.181194	-1.576911	-1.535255	0.307538
C	-0.329754	-1.907778	-0.128177	-0.701722	-1.867930	0.352672	-0.703589	-1.836615	0.366097	-0.142160	-1.965707	0.112342
C	0.480520	-0.598330	-0.338925	-0.039551	-0.605533	-0.265730	-0.047755	-0.570689	-0.251953	0.567444	-0.627752	-0.236589
C	1.742567	-0.424106	0.527655	1.469712	-0.427916	-0.011885	1.462102	-0.389361	-0.004678	1.756824	-0.227105	0.657304
C	1.449163	-0.475072	2.029262	1.823089	-0.370176	1.476576	1.822033	-0.333235	1.482237	1.380081	-0.106507	2.136185
C	2.540107	0.843956	0.138913	2.050816	0.777667	-0.789322	2.036256	0.819276	-0.782567	2.467352	1.045827	0.137719
C	3.997913	0.364890	0.159628	3.381060	0.239903	-1.333757	3.364953	0.285924	-1.335103	3.956222	0.708121	0.293510
C	3.878925	-1.081703	-0.327011	3.054752	-1.228866	-1.616299	3.040744	-1.183029	-1.618973	3.987846	-0.794795	0.003820
C	4.953946	-2.068258	0.186610	4.234233	-2.224687	-1.515041	4.223173	-2.176223	-1.526867	5.108776	-1.603099	0.698763
C	6.360843	-1.574181	-0.152368	5.382151	-1.813300	-2.437812	5.365060	-1.759652	-2.454774	6.487235	-1.032351	0.363312
O	4.877316	-2.123761	1.614108	4.752085	-2.183959	-0.182051	4.748058	-2.137929	-0.196574	4.951865	-1.475988	2.115084
C	4.711629	-3.468648	-0.401206	3.754514	-3.648554	-1.842831	3.745100	-3.600396	-1.855871	5.019927	-3.085034	0.297279
O	2.605924	-1.536768	0.184018	2.100517	-1.595565	-0.594981	2.093551	-1.554325	-0.592714	2.728895	-1.292249	0.510021
O	-0.429291	0.510965	-0.062880	-0.743605	0.552082	0.280871	-0.752635	0.582996	0.300587	-0.446510	0.421842	-0.158491
C	-1.664197	0.006196	0.220622	-1.768079	0.113076	1.064453	-1.777100	0.138808	1.081528	-1.648889	-0.150111	0.136355
C	-2.770843	0.812118	0.509057	-2.659612	0.972996	1.715119	-2.672050	0.994860	1.733944	-2.832878	0.583306	0.261918
C	-2.674411	2.327067	0.505748	-2.573665	2.478541	1.547811	-2.591067	2.501174	1.572016	-2.861313	2.087018	0.055305
C	-2.639581	2.899275	-0.893982	-3.234063	2.951546	0.271622	-3.254625	2.975941	0.298103	-2.802496	2.469082	-1.406982
C	-3.512908	3.737643	-1.471572	-2.671173	3.557956	-0.784188	-2.694855	3.586848	-0.756768	-3.704536	3.154569	-2.125030
C	-3.305008	4.197768	-2.895893	-3.511445	3.963501	-1.972654	-3.538147	3.994669	-1.942324	-3.463451	3.435844	-3.590082
C	-4.748088	4.300585	-0.811710	-1.206388	3.897312	-0.910224	-1.231032	3.929957	-0.884150	-5.005209	3.705813	-1.593447
C	-3.958984	0.131082	0.778254	-3.642567	0.355586	2.493412	-3.651771	0.369857	2.507054	-3.977100	-0.158168	0.568398
O	-6.370986	-1.281550	1.336920	-4.901565	-2.896935	3.580862	-5.642782	-0.928109	4.081501	-5.180371	-3.542268	1.234611
H	-6.232228	-3.529064	1.243532	-6.237201	-1.363771	4.552873	-5.562303	-3.180425	4.097454	-6.985673	-2.203766	1.399921
H	-2.964807	-3.126129	0.470027	-2.896303	-2.944603	2.052338	-2.883160	-2.929616	2.047629	-2.714968	-3.323390	0.756825
H	0.091380	-2.523951	0.673172	0.002666	-2.441858	0.963909	0.003753	-2.406424	0.977999	0.282282	-2.432028	1.007520
H	-0.318460	-2.522094	-1.034684	-1.059297	-2.543582	-0.431203	-1.056285	-2.514234	-0.418367	-0.029562	-2.689735	-0.701423
H	0.780700	-0.498788	-1.387629	-0.200410	-0.581866	-1.349040	-0.213702	-0.545316	-1.334523	0.920062	-0.640362	-1.273510
H	0.722114	0.293649	2.307977	1.284625	0.444111	1.970803	1.282913	0.478195	1.980391	0.576452	0.623412	2.272782
H	1.053312	-1.451468	2.326292	1.574134	-1.308192	1.982903	1.578673	-1.273106	1.987891	1.049764	-1.067088	2.544103
H	2.370481	-0.301669	2.595278	2.897913	-0.199423	1.598546	2.896791	-0.158965	1.599704	2.248618	0.222243	2.716567
H	2.346809	1.675979	0.821347	2.167690	1.659026	-0.152875	2.154323	1.699524	-0.144832	2.164761	1.939572	0.690021
H	2.260870	1.175190	-0.868294	1.384115	1.055485	-1.614266	1.364589	1.097341	-1.603368	2.222096	1.214524	-0.917544
H	4.651202	0.971066	-0.474605	3.720711	0.775512	-2.224991	3.698569	0.824230	-2.226980	4.593333	1.282249	-0.385438
H	4.403202	0.369952	1.176761	4.170680	0.294063	-0.577186	4.158436	0.340468	-0.582599	4.298148	0.883928	1.318705
H	3.854267	-1.117317	-1.427775	2.577961	-1.334602	-2.603750	2.558352	-1.287756	-2.603807	4.032469	-0.977350	-1.081645
H	7.100883	-2.299255	0.201051	6.193675	-2.544279	-2.364422	6.178787	-2.488786	-2.387632	7.262767	-1.634189	0.847701
H	6.486239	-1.454728	-1.234457	5.051995	-1.769948	-3.481856	5.029184	-1.714461	-3.496915	6.666697	-1.045945	-0.717669
H	6.568242	-0.616742	0.334419	5.782724	-0.835748	-2.153819	5.764719	-0.781839	-2.170437	6.584477	-0.004163	0.723957
H	3.940917	-2.305166	1.807869	3.977234	-2.315925	0.391954	3.976032	-2.270349	0.381144	4.024702	-1.716856	2.287208
H	4.821245	-3.469796	-1.492491	3.404460	-3.724883	-2.879422	3.389668	-3.674853	-2.890771	5.194386	-3.220418	-0.777032
H	5.433688	-4.174539	0.021268	4.578021	-4.355589	-1.701219	4.571056	-4.305806	-1.720539	5.771860	-3.660340	0.846598
H	3.701743	-3.817747	-0.162942	2.928569	-3.938428	-1.185378	2.923419	-3.893929	-1.194687	4.030334	-3.489983	0.532249
H	-3.508117	2.735704	1.083128	-3.075797	2.948800	2.404474	-3.094288	2.966193	2.430650	-3.753767	2.494414	0.538339
H	-1.752812	2.620560	1.028543	-1.526448	2.792258	1.586806	-1.544819	2.818148	1.610970	-1.994605	2.527368	0.568805
H	-1.788858	2.564044	-1.487303	-4.305538	2.751196	0.227468	-4.325743	2.773653	0.255652	-1.903336	2.124089	-1.917449
H	-2.400186	3.767579	-3.336630	-4.566633	3.707585	-1.834899	-4.592459	3.735790	-1.803546	-2.511555	3.019996	-3.934890
H	-4.158441	3.918363	-3.529865	-3.444900	5.045718	-2.153772	-3.474552	5.077715	-2.119540	-4.265048	3.011512	-4.211179
H	-3.223236	5.292625	-2.951102	-3.159197	3.473464	-2.891454	-3.186356	3.508907	-2.863607	-3.455296	4.516694	-3.790447
H	-4.890679	3.956615	0.215215	-0.602389	3.522117	-0.082140	-0.624779	3.552794	-0.058592	-5.855963	3.295604	-2.155155
H	-4.711994	5.398858	-0.794940	-0.795783	3.473948	-1.837259	-0.821080	3.511329	-1.813666	-5.171038	3.491156	-0.535224
H	-5.647158	4.029309	-1.382216	-1.062913	4.985132	-0.975955	-1.090042	5.018351	-0.945700	-5.045854	4.796300	-1.723653
H	-4.861435	0.689256	1.008978	-4.359007	0.974564	3.024189	-4.379260	0.970305	3.045574	-4.925694	0.357830	0.675606

	**3b-5**			**3b-6**			**3b-7**			**3b-8**		
**Element**	**X**	**Y**	**Z**	**X**	**Y**	**Z**	**X**	**Y**	**Z**	**X**	**Y**	**Z**
O	-6.598447	-1.094899	-0.070436	-5.028518	-3.279440	2.311177	-5.998971	-1.206365	3.366692	-5.838347	-3.220164	0.358246
C	-5.539587	-1.946242	-0.167931	-5.100531	-1.920360	2.241797	-5.151136	-2.028028	2.687080	-5.878638	-1.858463	0.321723
C	-4.241711	-1.253866	-0.322059	-3.961417	-1.313848	1.519029	-4.153027	-1.299233	1.875154	-4.576755	-1.247232	-0.022221
C	-3.085535	-2.048428	-0.433626	-2.927047	-2.092316	0.966397	-3.231734	-2.059433	1.131780	-3.431917	-2.022446	-0.284082
C	-1.861129	-1.423712	-0.587006	-1.900491	-1.448399	0.296237	-2.291996	-1.399910	0.360057	-2.251218	-1.373519	-0.605021
C	-0.456368	-1.966855	-0.709226	-0.652836	-1.969766	-0.378471	-1.194911	-1.907890	-0.544684	-0.876752	-1.897998	-0.946192
C	0.366400	-0.684497	-1.006043	-0.076028	-0.687605	-1.036491	-0.532051	-0.593763	-1.043139	-0.058915	-0.592955	-1.153625
C	1.652831	-0.470752	-0.188502	1.424725	-0.410397	-0.836896	0.953371	-0.406316	-0.673359	1.129704	-0.383666	-0.193528
C	1.406552	-0.435041	1.321936	1.826304	-0.306802	0.636627	1.195635	-0.417724	0.838303	0.706765	-0.350180	1.277496
C	2.445048	0.767683	-0.672680	1.897610	0.818244	-1.650854	1.567762	0.844422	-1.346417	1.978165	0.852093	-0.577855

C	3.900187	0.280208	-0.667468	3.240548	0.359355	-2.234809	2.941904	0.353505	-1.821613	3.421501	0.358549	-0.409647
C	3.759066	-1.190984	-1.066879	3.001026	-1.131589	-2.486701	2.661228	-1.103246	-2.199184	3.320525	-1.110415	-0.828435
C	4.843050	-2.154396	-0.528616	4.245541	-2.047584	-2.415811	3.847945	-2.086515	-2.065344	4.332874	-2.080474	-0.174884
C	6.242242	-1.690516	-0.935431	5.330987	-1.576834	-3.384626	5.049636	-1.613147	-2.884102	5.771001	-1.621083	-0.418230
O	4.807611	-2.127562	0.901345	4.806339	-1.954133	-1.103009	4.271644	-2.106399	-0.699059	4.135193	-2.059199	1.241832
C	4.575875	-3.584181	-1.028040	3.848128	-3.504220	-2.708198	3.416968	-3.498499	-2.496231	4.118912	-3.507174	-0.707767
O	2.498955	-1.607127	-0.494502	2.109447	-1.543902	-1.426527	1.645559	-1.534196	-1.266634	2.002693	-1.522695	-0.403647
O	-0.526449	0.448549	-0.773693	-0.833413	0.437497	-0.494279	-1.298776	0.509880	-0.469502	-0.991411	0.519658	-0.991652
C	-1.795853	-0.027530	-0.621668	-1.907299	-0.055005	0.188188	-2.270129	-0.002352	0.336519	-2.214909	0.022417	-0.657374
C	-2.917117	0.800776	-0.515284	-2.914033	0.756414	0.721645	-3.164967	0.792822	1.060913	-3.328743	0.830979	-0.402127
C	-2.795323	2.313555	-0.553588	-2.876396	2.267773	0.584034	-3.083335	2.306836	1.031241	-3.236174	2.344148	-0.437246
C	-2.322895	2.889191	0.762728	-1.913492	2.913076	1.555552	-2.012080	2.844487	1.954209	-2.654222	2.914805	0.837207
C	-2.960464	3.745896	1.574382	-2.182896	3.780770	2.542347	-0.960229	3.620037	1.649226	-1.568574	3.689667	0.985020
C	-2.316375	4.205865	2.861666	-1.072633	4.314903	3.417303	-0.010058	4.083483	2.729107	-1.163949	4.190474	2.352240
C	-4.328345	4.331636	1.321379	-3.553974	4.308285	2.888521	-0.625053	4.118721	0.264726	-0.675737	4.152644	-0.140320
C	-4.142915	0.146762	-0.361687	-3.938235	0.084582	1.391366	-4.108267	0.104267	1.828587	-4.508003	0.154347	-0.086243
O	-5.690817	-3.153386	-0.126971	-6.027574	-1.308780	2.739484	-5.240849	-3.239280	2.768737	-6.905368	-1.248406	0.556150
H	-7.379799	-1.669499	0.026449	-5.823689	-3.552397	2.804377	-6.596571	-1.802683	3.853857	-6.743306	-3.496157	0.592579
H	-3.184765	-3.128656	-0.399380	-2.946732	-3.172038	1.067553	-3.284404	-3.142469	1.179552	-3.486686	-3.104691	-0.236327
H	-0.129484	-2.462415	0.211810	0.044967	-2.413200	0.340826	-0.477101	-2.552512	-0.027834	-0.447427	-2.522826	-0.156656
H	-0.345886	-2.696695	-1.516818	-0.858460	-2.735702	-1.132353	-1.590080	-2.488913	-1.385317	-0.881468	-2.504357	-1.858848
H	0.637624	-0.653974	-2.068201	-0.271409	-0.703316	-2.115530	-0.621241	-0.500302	-2.130189	0.322496	-0.530778	-2.177841
H	0.677703	0.340022	1.577782	1.240200	0.464884	1.144913	0.621768	0.375178	1.327446	-0.016286	0.452010	1.453349
H	1.032856	-1.396612	1.687743	1.675281	-1.256946	1.158697	0.910989	-1.377517	1.281404	0.255676	-1.299071	1.585314
H	2.342707	-0.218941	1.847422	2.887074	-0.046859	0.716781	2.258885	-0.255873	1.045553	1.582076	-0.174568	1.912032
H	2.276507	1.638530	-0.033394	1.977437	1.715342	-1.030950	1.624545	1.692947	-0.659405	1.741884	1.720268	0.043106
H	2.137459	1.042793	-1.688427	1.185981	1.038515	-2.455268	0.956419	1.156128	-2.201674	1.789801	1.134514	-1.620495
H	4.537320	0.844512	-1.354568	3.513439	0.902280	-3.144332	3.334152	0.938362	-2.658686	4.135213	0.922453	-1.017282
H	4.335969	0.340610	0.335176	4.050743	0.475788	-1.507559	3.675385	0.379279	-1.009088	3.740322	0.413110	0.636410
H	3.702182	-1.289012	-2.162626	2.497321	-1.282750	-3.454654	2.256050	-1.165019	-3.221792	3.387869	-1.204180	-1.924057
H	6.988213	-2.399580	-0.562528	6.190229	-2.253016	-3.331795	5.866533	-2.335835	-2.790775	6.467111	-2.334618	0.034115
H	6.336865	-1.633745	-2.025723	4.961950	-1.569982	-4.416456	4.791668	-1.521164	-3.945195	5.989134	-1.560759	-1.490474
H	6.469001	-0.708403	-0.510296	5.677220	-0.571534	-3.127387	5.412942	-0.645989	-2.524475	5.951745	-0.641516	0.033956
H	3.876728	-2.293472	1.132049	4.061522	-2.123919	-0.499898	3.460744	-2.274414	-0.187593	3.182469	-2.216509	1.364211
H	4.653564	-3.648397	-2.120165	3.467427	-3.618437	-3.730417	3.140911	-3.527054	-3.557320	4.320074	-3.567425	-1.784260
H	5.306007	-4.270022	-0.586839	4.720392	-4.154411	-2.587596	4.240913	-4.200246	-2.332544	4.791509	-4.197850	-0.189388
H	3.571455	-3.911782	-0.741146	3.066564	-3.836948	-2.017573	2.552708	-3.832908	-1.913437	3.087162	-3.831621	-0.538408
H	-2.072690	2.585248	-1.335944	-2.563170	2.517114	-0.439610	-2.921049	2.644865	0.003621	-2.652715	2.655418	-1.308639
H	-3.757740	2.739245	-0.850851	-3.889509	2.659450	0.709175	-4.057102	2.708664	1.344735	-4.249553	2.745742	-0.576258
H	-1.336231	2.540768	1.069032	-0.875276	2.610423	1.416724	-2.139930	2.553281	2.997793	-3.214309	2.651993	1.735724
H	-1.327570	3.759495	3.006662	-0.096646	3.908506	3.133767	-0.298503	3.709924	3.716590	-1.845327	3.841591	3.134443
H	-2.202158	5.299027	2.879535	-1.014635	5.411103	3.359424	1.016876	3.749119	2.524592	-0.148394	3.859538	2.611894
H	-2.937929	3.944871	3.729919	-1.249497	4.069203	4.474002	0.026353	5.181046	2.778608	-1.147859	5.289252	2.381390
H	-4.785133	3.988941	0.390100	-4.348038	3.914403	2.250162	-1.242168	3.672261	-0.517421	-0.906266	3.681342	-1.097747
H	-5.013535	4.079265	2.142580	-3.809647	4.062938	3.928624	-0.736985	5.210774	0.206997	-0.746404	5.241995	-0.269769
H	-4.279303	5.428922	1.285850	-3.577285	5.404492	2.814397	0.424736	3.903504	0.022945	0.376478	3.937654	0.091077
H	-5.048425	0.738811	-0.277304	-4.757591	0.647096	1.828922	-4.831528	0.671404	2.405993	-5.414748	0.716324	0.118157

	3b-9			3b-10			3b-11			3b-12		
**Element**	**X**	**Y**	**Z**	**X**	**Y**	**Z**	**X**	**Y**	**Z**	**X**	**Y**	**Z**
O	-5.919926	-2.016199	2.035936	-5.735872	-1.731423	3.423627	-5.356268	-3.977447	-0.942651	-5.545103	-4.187898	0.266957
C	-4.976125	-2.905284	1.619529	-4.857478	-2.638241	2.912736	-5.299469	-2.618022	-0.865706	-5.554082	-2.826879	0.336874
C	-3.801471	-2.263099	0.990447	-3.731326	-2.017233	2.182554	-3.917426	-2.091488	-0.895786	-4.207968	-2.229164	0.203070
C	-2.770490	-3.100197	0.524009	-2.764183	-2.871187	1.621980	-2.797291	-2.938302	-0.997041	-3.053903	-3.015752	0.032160
C	-1.667637	-2.521937	-0.076559	-1.702667	-2.311261	0.934827	-1.538150	-2.364299	-1.030179	-1.831230	-2.376626	-0.084887
C	-0.393376	-3.108423	-0.638880	-0.492497	-2.925531	0.272036	-0.152564	-2.964958	-1.090845	-0.424940	-2.910697	-0.221690
C	0.244904	-1.879804	-1.340323	0.143293	-1.702605	-0.444126	0.731397	-1.712051	-1.331448	0.391284	-1.616007	-0.487004
C	1.759685	-1.698687	-1.159762	1.668455	-1.569668	-0.316444	2.043455	-1.636601	-0.535847	1.711226	-1.485192	0.288223
C	2.506123	-2.878811	-1.791526	2.353523	-2.768446	-0.984740	2.967323	-2.790630	-0.940434	2.673803	-2.604200	-0.129193
C	2.178946	-1.431177	0.310145	2.150095	-1.332510	1.136419	1.830768	-1.537442	0.998100	1.521507	-1.393733	1.822850
C	3.133231	-0.231622	0.206465	3.217842	-0.239866	0.987836	2.759567	-0.389911	1.423341	2.492623	-0.280636	2.240150
C	2.584810	0.527625	-1.004348	2.692801	0.581282	-0.192961	2.761704	0.513317	0.187452	2.473645	0.651396	1.025606
C	3.599641	1.386911	-1.792644	3.761016	1.310251	-1.041703	4.020293	1.385925	-0.022473	3.760075	1.474796	0.781015
C	4.283080	2.406527	-0.880710	4.619020	2.231886	-0.174134	4.285302	2.271636	1.195569	4.119761	2.312223	2.008849
O	4.633760	0.531767	-2.291195	4.648564	0.332993	-1.595576	5.157117	0.525717	-0.153197	4.849389	0.568872	0.575073
C	2.896933	2.089551	-2.966478	3.089398	2.100142	-2.177251	3.865865	2.238010	-1.293467	3.590967	2.369201	-0.458288
O	2.138833	-0.513773	-1.895693	2.061361	-0.388296	-1.053099	2.704806	-0.409732	-0.918460	2.318491	-0.232156	-0.103162
O	-0.449529	-0.707989	-0.813066	-0.502613	-0.520090	0.117981	-0.102907	-0.560275	-1.000761	-0.490076	-0.502704	-0.149345
C	-1.593048	-1.130953	-0.203006	-1.606820	-0.921995	0.807393	-1.401111	-0.974778	-0.957185	-1.760467	-0.981462	-0.034064
C	-2.591983	-0.262679	0.247205	-2.544932	-0.036822	1.350174	-2.486272	-0.098392	-0.853687	-2.883533	-0.163083	0.136006
C	-2.464471	1.242309	0.096774	-2.434349	1.462993	1.148839	-2.291167	1.404315	-0.767616	-2.775073	1.350229	0.136895
C	-1.615209	1.858433	1.186018	-3.008004	1.895920	-0.182500	-1.941645	1.858702	0.631944	-2.760451	1.913526	-1.267208
C	-1.959008	2.795288	2.082408	-2.374571	2.466062	-1.218275	-2.613374	2.706740	1.425039	-1.782280	2.593823	-1.883240
C	-0.957750	3.284024	3.103247	-3.129560	2.816334	-2.479286	-2.097873	3.035085	2.807049	-1.958091	3.067578	-3.307159

C	-3.315279	3.450241	2.181156	-0.905443	2.805751	-1.256056	-3.899802	3.406794	1.060405	-0.451754	2.948611	-1.267552
C	-3.697611	-0.869394	0.850707	-3.607306	-0.624685	2.042775	-3.746146	-0.699562	-0.822567	-4.104645	-0.829164	0.254940
O	-5.123324	-4.104215	1.770040	-5.020135	-3.834966	3.065387	-6.309854	-1.945138	-0.781348	-6.589404	-2.207092	0.494716
H	-6.628739	-2.559692	2.426179	-6.418988	-2.262047	3.872922	-6.305802	-4.195594	-0.912932	-6.477154	-4.454892	0.367246
H	-2.869560	-4.174140	0.645329	-2.873872	-3.943795	1.746244	-2.931871	-4.013245	-1.049748	-3.133123	-4.096993	-0.001316
H	0.249632	-3.496435	0.162689	0.188495	-3.358640	1.016699	0.102840	-3.465097	-0.146717	-0.100896	-3.412209	0.700025
H	-0.564329	-3.928265	-1.343410	-0.742543	-3.720062	-0.438077	-0.030189	-3.701063	-1.891547	-0.310335	-3.630250	-1.038631
H	0.032783	-1.897672	-2.416292	-0.108718	-1.704203	-1.511269	0.975824	-1.612493	-2.396079	0.613804	-1.510417	-1.555499
H	2.236273	-3.830332	-1.320802	2.071301	-3.716359	-0.514177	2.516976	-3.766388	-0.728443	2.267488	-3.597360	0.090419
H	2.283448	-2.945744	-2.862337	2.089172	-2.816929	-2.047136	3.199423	-2.740366	-2.010161	2.885587	-2.546182	-1.202833
H	3.585585	-2.735794	-1.677422	3.440578	-2.661272	-0.910082	3.908362	-2.721377	-0.385147	3.620748	-2.496614	0.409739
H	2.649287	-2.307368	0.766820	2.538993	-2.246484	1.595554	2.063171	-2.479241	1.504603	1.728342	-2.346051	2.320609
H	1.304415	-1.166988	0.912457	1.322212	-0.969446	1.753206	0.790166	-1.283369	1.221591	0.492715	-1.104692	2.058762
H	3.148220	0.373382	1.117745	3.339948	0.355595	1.897300	2.409577	0.121900	2.324569	2.187013	0.219970	3.163504
H	4.156543	-0.555332	-0.011040	4.190036	-0.668029	0.722013	3.778557	-0.748812	1.602570	3.507165	-0.669325	2.377203
H	1.723228	1.147553	-0.715611	1.935926	1.304568	0.145466	1.865035	1.150210	0.170972	1.607190	1.327874	1.071003
H	4.980293	3.014826	-1.465837	5.351644	2.750998	-0.800411	5.167921	2.893269	1.013766	5.019725	2.900546	1.803004
H	3.548505	3.074928	-0.417587	4.002238	2.984775	0.329486	3.434109	2.932550	1.394252	3.308603	3.002953	2.265336
H	4.852610	1.906945	-0.091446	5.166397	1.661577	0.582084	4.477516	1.666213	2.086302	4.325257	1.673627	2.872976
H	4.161367	-0.199574	-2.726687	4.063869	-0.320712	-2.017849	4.894429	-0.131406	-0.821654	4.534221	-0.044366	-0.112023
H	2.135620	2.795159	-2.612679	2.445231	2.896265	-1.785189	3.035340	2.948107	-1.200706	2.799952	3.114022	-0.310043
H	3.633918	2.640267	-3.559727	3.856391	2.553881	-2.813104	4.787464	2.800877	-1.472939	4.529558	2.893838	-0.663868
H	2.403068	1.357703	-3.613521	2.471524	1.438663	-2.793056	3.669515	1.601770	-2.162275	3.327893	1.767472	-1.334217
H	-2.001708	1.456133	-0.876648	-2.987461	1.960563	1.957416	-1.474982	1.688418	-1.446282	-3.640534	1.756539	0.678001
H	-3.464732	1.683849	0.076234	-1.389251	1.769910	1.245395	-3.194625	1.900893	-1.131912	-1.883282	1.653735	0.692184
H	-0.598254	1.466952	1.228949	-4.073292	1.688308	-0.293178	-1.024226	1.418611	1.024400	-3.669884	1.709828	-1.834210
H	0.010942	2.785454	2.997680	-4.188538	2.548244	-2.410808	-1.165456	2.507778	3.032262	-2.933397	2.784872	-3.715579
H	-0.794647	4.367238	3.011799	-3.063668	3.892235	-2.694549	-1.913795	4.113332	2.915810	-1.864071	4.160629	-3.375412
H	-1.319762	3.110361	4.126502	-2.702916	2.299436	-3.350522	-2.834726	2.767180	3.577425	-1.180258	2.648348	-3.961036
H	-4.033154	3.082059	1.444653	-0.380974	2.560465	-0.330588	-4.266504	3.156053	0.062492	-0.348931	2.609376	-0.235025
H	-3.748077	3.292368	3.178787	-0.406488	2.261892	-2.069318	-4.691762	3.156900	1.779903	0.369379	2.500577	-1.843076
H	-3.230202	4.537972	2.049684	-0.761023	3.876292	-1.458421	-3.773129	4.497472	1.105909	-0.293556	4.036018	-1.288293
H	-4.506332	-0.244515	1.215586	-4.363838	0.015499	2.485557	-4.636909	-0.082859	-0.748422	-5.019001	-0.258680	0.390464

	**3b-13**			**3b-14**			**3b-15**			**3b-16**		
**Element**	**X**	**Y**	**Z**	**X**	**Y**	**Z**	**X**	**Y**	**Z**	**X**	**Y**	**Z**
O	-5.350936	-2.053679	3.120760	-6.215492	-1.895366	2.391475	-5.378582	-4.052087	2.035423	-5.109400	-3.654529	0.997653
C	-4.329104	-2.901553	2.817998	-5.316078	-2.802164	1.918648	-5.336759	-2.690586	1.993198	-5.258808	-2.308922	0.842737
C	-3.219155	-2.247397	2.091414	-4.143513	-2.180269	1.266520	-4.133521	-2.167253	1.310960	-3.983365	-1.609227	0.575568
C	-2.110193	-3.039645	1.741572	-3.154782	-3.034104	0.744516	-3.149830	-3.016835	0.771917	-2.755869	-2.294015	0.504490
C	-1.062682	-2.449260	1.058436	-2.055172	-2.473361	0.121520	-2.059187	-2.445323	0.139338	-1.605271	-1.565656	0.251844
C	0.269450	-2.983773	0.585745	-0.823161	-3.088659	-0.499157	-0.832657	-3.053298	-0.499488	-0.157665	-1.980402	0.129410
C	0.805200	-1.807365	-0.274162	-0.146937	-1.861079	-1.167507	-0.168794	-1.819746	-1.169295	0.551464	-0.636879	-0.198308
C	2.287324	-1.446512	-0.089819	1.371758	-1.734716	-0.970257	1.352133	-1.690319	-0.991983	1.703811	-0.221017	0.737067
C	3.169943	-2.618935	-0.531742	2.085335	-2.921840	-1.627053	2.059989	-2.871885	-1.664840	1.273965	-0.109007	2.201409
C	2.624625	-0.934397	1.335630	1.784708	-1.519731	0.509724	1.784506	-1.482200	0.483448	2.407625	1.068662	0.239953
C	3.458784	0.330580	1.082516	2.793614	-0.363592	0.442864	2.789073	-0.322728	0.409219	3.901859	0.742347	0.391184
C	2.879966	0.853250	-0.234621	2.284830	0.451468	-0.748681	2.261637	0.497316	-0.770717	3.931058	-0.753837	0.072308
C	3.830019	1.695902	-1.115945	3.341467	1.284356	-1.510010	3.305213	1.337763	-1.541674	5.084566	-1.623137	0.615670
C	4.375450	2.898022	-0.344118	4.065990	2.248063	-0.569369	4.039573	2.298412	-0.605553	5.231645	-1.538066	2.142456
O	4.961539	0.892428	-1.467148	4.339061	0.396744	-2.026150	4.298411	0.456127	-2.076359	4.797873	-2.979386	0.243756
C	3.101437	2.151265	-2.391578	2.679292	2.047007	-2.669883	2.624904	2.104851	-2.688023	6.395528	-1.268907	-0.087693
O	2.571901	-0.342633	-0.978489	1.797431	-0.544355	-1.670757	1.764896	-0.495149	-1.691663	2.699939	-1.265504	0.624394
O	-0.019869	-0.649827	0.062487	-0.807123	-0.681068	-0.615023	-0.824590	-0.645522	-0.600649	-0.474732	0.402581	-0.162327
C	-1.122007	-1.090917	0.732850	-1.937742	-1.082492	0.027218	-1.946460	-1.054087	0.052617	-1.683971	-0.182216	0.074544
C	-2.199040	-0.264588	1.068622	-2.895242	-0.196988	0.535181	-2.900303	-0.173871	0.578138	-2.881059	0.538758	0.141350
C	-2.225585	1.203749	0.683584	-2.734182	1.305719	0.427471	-2.745582	1.330027	0.479077	-2.917797	2.041476	-0.070534
C	-2.513493	1.409834	-0.787052	-1.781179	1.871157	1.460070	-1.782965	1.890896	1.505130	-2.788769	2.420714	-1.529110
C	-3.549036	2.050550	-1.349472	-0.881992	2.854861	1.304557	-0.885968	2.876028	1.346110	-3.667959	3.080587	-2.297722
C	-3.662940	2.148662	-2.853143	-0.035432	3.313911	2.468819	-0.029783	3.330619	2.505087	-3.355254	3.362934	-3.748968
C	-4.672397	2.721459	-0.597044	-0.639715	3.607072	0.018861	-0.655853	3.634753	0.061993	-5.009366	3.599120	-1.839781
C	-3.247240	-0.883373	1.755584	-4.000060	-0.787116	1.156416	-3.993139	-0.773531	1.207325	-4.026821	-0.217009	0.394353
O	-4.364753	-4.076640	3.133705	-5.496983	-3.999580	2.042392	-6.222204	-2.013559	2.481575	-6.351341	-1.779022	0.924739
H	-6.006972	-2.601048	3.589903	-6.928560	-2.426473	2.790902	-6.205966	-4.267917	2.503319	-6.007629	-3.993582	1.165783
H	-2.105457	-4.089272	2.017519	-3.282120	-4.107625	0.841106	-3.256902	-4.093084	0.853943	-2.724683	-3.368673	0.647934
H	0.931896	-3.199758	1.434742	-0.178099	-3.539258	0.266892	-0.176348	-3.505718	0.256011	0.227736	-2.431935	1.049763
H	0.185783	-3.903368	-0.001848	-1.049725	-3.869879	-1.231560	-1.064863	-3.831216	-1.233785	0.003382	-2.710610	-0.670646
H	0.634251	-1.998906	-1.340263	-0.350770	-1.843972	-2.244798	-0.387310	-1.796925	-2.243631	0.941639	-0.650865	-1.221820
H	2.978897	-3.519201	0.062231	1.779424	-3.876849	-1.186506	1.762731	-3.830143	-1.225348	0.436963	0.588287	2.304868
H	2.994209	-2.855644	-1.587109	1.872120	-2.951284	-2.701517	1.832310	-2.896128	-2.736469	0.972755	-1.080607	2.604524
H	4.225000	-2.350758	-0.415003	3.167705	-2.818364	-1.498489	3.143706	-2.766026	-1.550415	2.107625	0.257421	2.809252
H	3.161877	-1.684988	1.923355	2.208086	-2.426481	0.952547	2.216501	-2.390095	0.915538	2.096860	1.949990	0.807277
H	1.706987	-0.679008	1.874187	0.916435	-1.225027	1.106675	0.923574	-1.193180	1.093657	2.164351	1.254802	-0.812396
H	3.381865	1.054700	1.898823	2.829293	0.215689	1.370084	2.835694	0.251534	1.339053	4.534081	1.329004	-0.281801

H	4.516066	0.086748	0.934183	3.802631	-0.727339	0.221691	3.796008	-0.682248	0.172217	4.236596	0.927680	1.417384
H	1.950824	1.413419	-0.053751	1.448716	1.097438	-0.444064	1.427619	1.138736	-0.451260	3.910890	-0.902085	-1.019169
H	5.027664	3.488720	-0.995419	4.792684	2.839697	-1.135428	4.756625	2.895482	-1.178156	5.966056	-2.280342	2.469450
H	3.561842	3.543840	0.004907	3.360380	2.936993	-0.090902	3.338493	2.982416	-0.113590	5.573139	-0.551197	2.474651
H	4.964758	2.575142	0.519197	4.608165	1.702824	0.208785	4.594034	1.750510	0.162007	4.277862	-1.755353	2.633898
H	4.580454	0.062054	-1.803189	3.835996	-0.302576	-2.479638	3.791329	-0.241724	-2.527598	3.886456	-3.146771	0.537785
H	2.261137	2.815767	-2.157110	1.946699	2.776605	-2.303988	1.895194	2.830053	-2.307969	6.724430	-0.255481	0.165996
H	3.799489	2.687945	-3.042018	3.444137	2.579151	-3.244557	3.380190	2.642612	-3.270057	7.176011	-1.972404	0.219027
H	2.706479	1.288919	-2.937973	2.158200	1.353994	-3.337923	2.096812	1.414022	-3.352832	6.283391	-1.343240	-1.174610
H	-2.957766	1.720503	1.310228	-2.405024	1.563334	-0.584328	-2.428856	1.595891	-0.534548	-3.839331	2.437551	0.364588
H	-1.245294	1.644699	0.914867	-3.725966	1.762095	0.560826	-3.737277	1.781439	0.627555	-2.084472	2.494238	0.485658
H	-1.770521	0.965043	-1.448712	-1.877538	1.425406	2.450815	-1.870590	1.440891	2.494733	-1.855249	2.096444	-1.989050
H	-2.829413	1.651919	-3.359345	-0.252919	2.749263	3.380845	-0.238458	2.761182	3.416179	-2.375370	2.971490	-4.039829
H	-4.597894	1.693163	-3.209210	1.035866	3.203998	2.247273	1.039737	3.223628	2.273597	-4.110592	2.915040	-4.410154
H	-3.684355	3.197492	-3.181611	-0.198746	4.379810	2.682011	-0.192992	4.395157	2.725035	-3.365082	4.442888	-3.954022
H	-5.641049	2.290398	-0.885930	-1.201407	3.209386	-0.829376	-1.224454	3.240501	-0.783243	-5.817320	3.163324	-2.443659
H	-4.582394	2.638634	0.488551	-0.913919	4.665280	0.134383	-0.930416	4.692011	0.185046	-5.226488	3.384400	-0.790976
H	-4.722759	3.790242	-0.847872	0.426505	3.592251	-0.246372	0.408066	3.622698	-0.212610	-5.071935	4.687514	-1.978610
H	-4.112795	-0.292540	2.037216	-4.778556	-0.147404	1.559881	-4.777997	-0.153149	1.630232	-4.993956	0.272636	0.458462

3.4. Compound 5

Table S4.1 Detailed DP4+ probability of **5a** (Isomer 1) and **5b** (Isomer 2)

Functional	Solvent?	Basis Set	Type of Data
B3LYP	PCM	6-31+G(d,p)	Shielding Tensors

	Isomer 1	Isomer 2	Isomer 3	Isomer 4	Isomer 5	Isomer 6
sDP4+ (H data)	29.56%	70.44%	-	-	-	-
sDP4+ (C data)	99.02%	0.98%	-	-	-	-
sDP4+ (all data)	97.70%	2.30%	-	-	-	-
uDP4+ (H data)	83.12%	16.88%	-	-	-	-
uDP4+ (C data)	99.83%	0.17%	-	-	-	-
uDP4+ (all data)	99.96%	0.04%	-	-	-	-
DP4+ (H data)	67.38%	32.62%	-	-	-	-
DP4+ (C data)	100.00%	0.00%	-	-	-	-
DP4+ (all data)	100.00%	0.00%	-	-	-	-

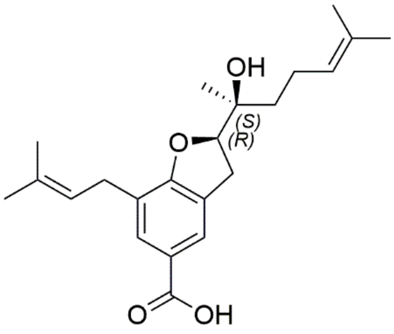

2*R*,1'*S*-**5** (**5a**)

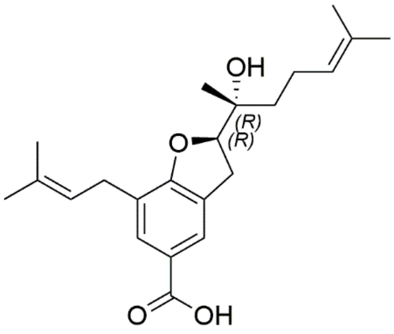

2*R*,1'*R*-**5** (**5b**)

Table S4.2. Experimental and calculated ^{1}H-NMR chemical shifts (δ in ppm) of **5a** and **5b**

Proton									
**5**		**5a (2*R*,1'*S*)**				**5b (2*R*,1'*R*)**			
No.	δ_H Exp. (mult., *J* in Hz)	δ_H Calc.	$\|\Delta\delta\|$	δ_H Calc. CORRECTED	$\|\Delta\delta\|$	δ_H Calc.	$\|\Delta\delta\|$	δ_H Calc. CORRECTED	$\|\Delta\delta\|$
2	4.72 (1H, t, 8.7)	4.86	0.14	4.64	0.08	5.03	0.31	4.78	0.06
3	3.21 (2H, m)*	3.28	0.07	3.12	0.09	3.32	0.11	3.15	0.06
4	7.73 (1H, s)**	8.11	0.38	7.73	0.00	8.11	0.38	7.71	0.02
6	7.73 (1H, s)**	8.04	0.31	7.67	0.06	8.05	0.32	7.66	0.07
2'	1.51 (2H, m)	1.34	0.17	1.28	0.23	1.67	0.16	1.58	0.07
3'	2.11 (2H, m)	2.34	0.23	2.23	0.12	2.26	0.15	2.15	0.04
4'	5.10 (1H, t, 7.0)	5.47	0.37	5.22	0.12	5.51	0.41	5.24	0.14
6'	1.67 (3H, s)	1.76	0.09	1.67	0.00	1.77	0.10	1.68	0.01
7'	1.28 (3H, s)	1.43	0.15	1.36	0.08	1.13	0.15	1.08	0.20
8'	1.61 (3H, s)	1.74	0.13	1.65	0.04	1.76	0.15	1.67	0.06
1"	3.21 (2H, m)*	3.40	0.19	3.25	0.04	3.40	0.19	3.23	0.02
2"	5.26 (1H, t, 7.4)	5.53	0.27	5.28	0.02	5.47	0.21	5.20	0.06
4"	1.72 (3H, s)	1.89	0.17	1.80	0.08	1.89	0.17	1.79	0.07
5"	1.71 (3H, s)	1.74	0.03	1.66	0.05	1.74	0.03	1.65	0.06
		**MAE**	**0.19**	**CMAE**	**0.07**	**MAE**	**0.20**	**CMAE**	**0.07**

Table S4.3. Experimental and calculated ^13^C-NMR chemical shifts (δ in ppm) of **5a** and **5b**

Carbon									
	**5**	**5a (2*R*,1'*S*)**				**5b (2*R*,1'*R*)**			
No.	δ_C Exp.	δ_C Calc.	$\|\Delta\delta\|$	δ_C Calc. CORRECTED	$\|\Delta\delta\|$	δ_C Calc.	$\|\Delta\delta\|$	δ_C Calc. CORRECTED	$\|\Delta\delta\|$
2	89.78	93.82	4.04	93.62	3.84	91.07	1.29	90.70	0.92
3	30.10	33.77	3.67	30.92	0.82	33.82	3.72	30.81	0.71
3a	127.38	123.12	2.10	124.21	1.01	123.24	1.98	124.36	0.86
4	125.22	119.06	2.87	119.97	1.96	118.96	2.97	119.89	2.04
5	121.93	130.08	1.54	131.48	0.14	130.03	1.59	131.46	0.16
6	131.62	123.32	0.02	124.42	1.08	123.19	0.15	124.31	0.97
7	123.34	76.20	2.23	75.22	1.25	75.98	2.01	74.91	0.94
7a	162.60	40.12	3.02	37.56	0.46	44.52	7.42	42.00	4.90
1'	73.97	26.76	4.60	23.61	1.45	28.24	6.08	24.97	2.81
2'	37.10	123.54	0.69	124.65	0.42	123.44	0.79	124.57	0.34
3'	22.16	133.33	0.90	134.87	2.44	133.26	0.83	134.84	2.41
4'	124.23	27.75	1.85	24.64	1.26	27.76	1.86	24.47	1.43
5'	132.43	24.71	1.87	21.46	1.38	22.03	0.81	18.47	4.37
6'	25.90	18.64	0.76	15.12	2.76	18.78	0.90	15.07	2.81
7'	22.84	34.67	6.18	31.86	3.37	34.90	6.41	31.93	3.44
8'	17.88	121.45	0.10	122.47	0.92	121.66	0.11	122.70	1.15
1"	28.49	133.13	0.27	134.67	1.27	133.37	0.03	134.96	1.56
2"	121.55	27.68	1.70	24.56	1.42	27.68	1.70	24.38	1.60
3"	133.40	19.26	1.20	15.77	2.29	19.37	1.31	15.69	2.37
4"	25.98	127.12	0.26	128.39	1.01	127.27	0.11	128.57	1.19
5"	18.06	159.55	3.05	162.25	0.35	159.80	2.80	162.61	0.01
COOH	172.05	163.42	8.63	166.30	5.75	163.38	8.67	166.35	5.70
		**MAE**	**2.34**	**CMAE**	**1.67**	**MAE**	**2.43**	**CMAE**	**1.94**

Table S4.4. Calculated conformational analysis of the **5a** at B3LYP/6-31g(d) level

conformer	3D conformer	G (Hartree)	ΔG (kcal/mol)	Population
5a-1	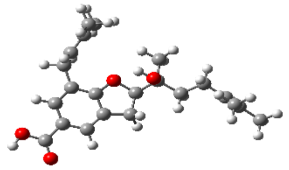	-1156.886182	0	5.248%
5a-2	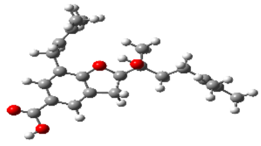	-1156.886172	0.0062751	5.192%
5a-3	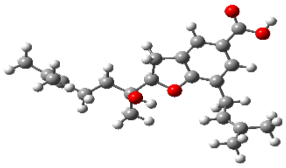	-1156.886107	0.04706325	4.847%
5a-4	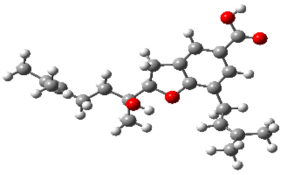	-1156.886078	0.06526104	4.700%
5a-5	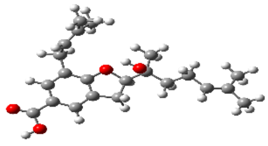	-1156.886042	0.0878514	4.524%
5a-6	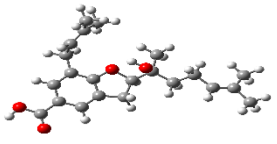	-1156.886026	0.09789156	4.448%
5a-7	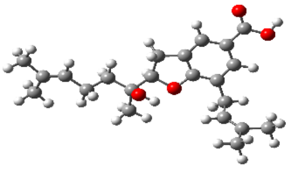	-1156.885927	0.16001505	4.005%
5a-8	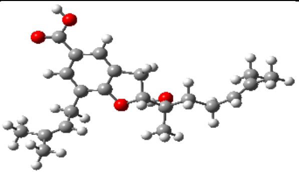	-1156.88586	0.20205822	3.731%

5a-9	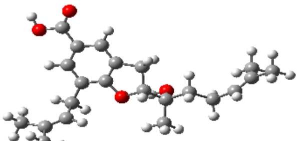	-1156.885839	0.21523593	3.649%
5a-10	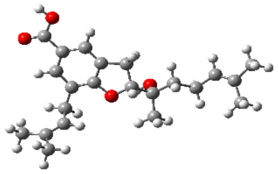	-1156.885732	0.2823795	3.258%
5a-11	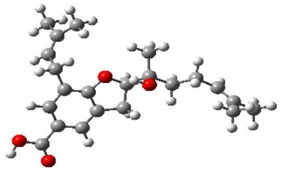	-1156.885656	0.33007026	3.006%
5a-12	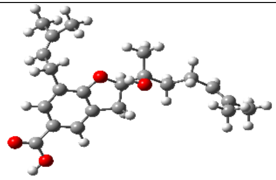	-1156.88564	0.34011042	2.956%
5a-13	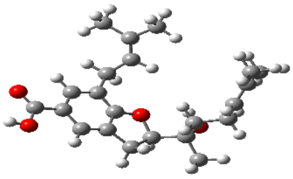	-1156.885535	0.40599897	2.644%
5a-14	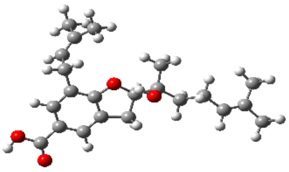	-1156.885462	0.4518072	2.448%
1a-15	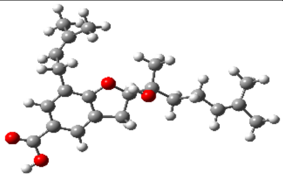	-1156.885456	0.45557226	2.432%
5a-16	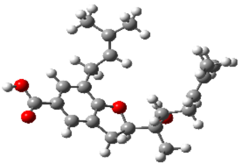	-1156.885426	0.47439756	2.356%
5a-17	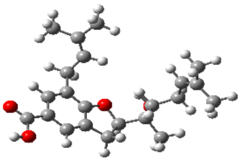	-1156.88532	0.54091362	2.106%

5a-18	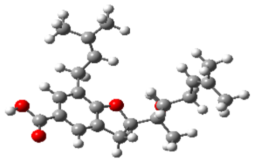	-1156.885284	0.56350398	2.027%
5a-19	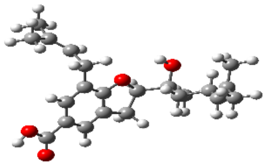	-1156.885188	0.62374494	1.831%
5a-20	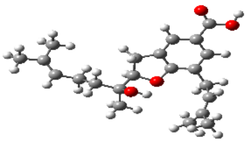	-1156.885167	0.63692265	1.791%
5a-21	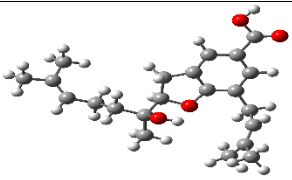	-1156.885124	0.66390558	1.711%
5a-22	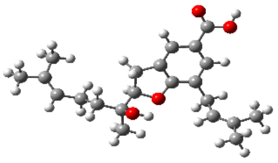	-1156.885089	0.68586843	1.649%
5a-23	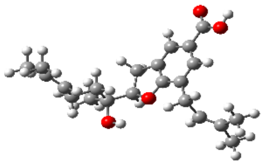	-1156.885067	0.69967365	1.611%
5a-24	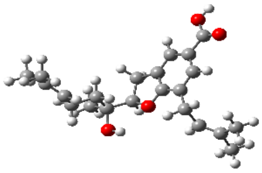	-1156.885012	0.7341867	1.520%
5a-25	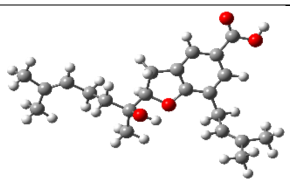	-1156.884864	0.82705818	1.299%
5a-26	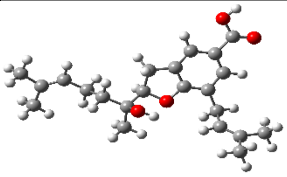	-1156.884863	0.82768569	1.298%

5a-27	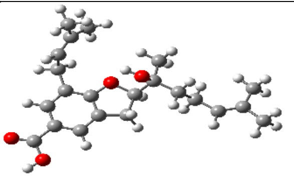	-1156.884857	0.83145075	1.290%
5a-28	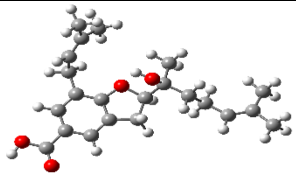	-1156.884836	0.84462846	1.261%
5a-29	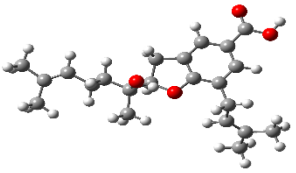	-1156.884748	0.89984934	1.149%

Table S4.5. Cartesian coordinates of low-energy conformers of **5a**

	5a-1			5a-2			5a-3			5a-4		
**Element**	**X**	**Y**	**Z**	**X**	**Y**	**Z**	**X**	**Y**	**Z**	**X**	**Y**	**Z**
C	1.557467	4.876473	-0.582193	2.574895	4.458911	-0.628997	2.246960	5.311478	0.293500	2.234394	5.334318	0.301690
C	0.708123	4.320976	0.537699	1.503395	4.167952	0.396377	1.648615	4.735858	1.556006	1.652820	4.755500	1.570557
C	-0.773926	4.551848	0.370143	0.128486	4.663711	0.019885	2.014882	5.485184	2.813930	2.054598	5.487451	2.827781
C	1.278843	3.699918	1.581578	1.808995	3.535785	1.540088	0.863752	3.649406	1.503591	0.852235	3.680323	1.523630
C	0.613130	3.100590	2.801015	0.895667	3.176003	2.691503	0.179608	2.926910	2.642488	0.181210	2.957096	2.669926
C	1.001198	1.650043	3.010768	0.956401	1.699481	3.030003	0.641599	1.487110	2.777318	0.643721	1.516913	2.797869
C	1.943930	1.249972	3.962842	1.678573	1.200988	4.116355	1.580254	1.082670	3.732069	1.583101	1.107022	3.746906
C	2.319696	-0.094136	4.122561	1.757121	-0.172336	4.399898	2.001870	-0.251995	3.844577	2.007760	-0.226763	3.855268
C	3.310369	-0.518101	5.136105	2.543442	-0.586982	5.582466	2.985827	-0.681554	4.862780	2.998511	-0.549815	4.905877
O	3.806048	0.515075	5.872327	2.553692	-1.936052	5.772424	3.426729	0.337080	5.651987	3.342828	-1.867711	4.929778
O	3.674368	-1.664008	5.325198	3.136009	0.164032	6.334663	3.387884	-1.820408	5.013133	3.482412	0.246503	5.688527
C	1.753686	-1.089094	3.304098	1.104423	-1.104399	3.571440	1.487806	-1.233972	2.976821	1.493933	-1.207807	2.985850
C	0.813731	-0.717476	2.360001	0.375976	-0.632617	2.492443	0.557201	-0.857413	2.025853	0.559685	-0.825246	2.038466
C	0.452397	0.626250	2.232604	0.309129	0.740247	2.244141	0.156333	0.478750	1.940959	0.157380	0.510230	1.958999
O	-0.493481	0.836117	1.271888	-0.454811	1.049823	1.156682	-0.766012	0.696112	0.955481	-0.767429	0.730063	0.976187
C	-0.970243	-0.471472	0.801394	-1.124622	-0.174941	0.700367	-1.217455	-0.611103	0.459782	-1.217972	-0.575213	0.476246
C	-1.154259	-0.364668	-0.728739	-1.100013	-0.158532	-0.844243	-1.423554	-0.485535	-1.065096	-1.425874	-0.444306	-1.047984
O	0.108261	-0.031098	-1.313654	0.264281	-0.123112	-1.274120	-0.169211	-0.157335	-1.671957	-0.172392	-0.112862	-1.654879
C	-2.190357	0.726188	-1.050937	-1.849212	1.081961	-1.361011	-2.456971	0.614797	-1.361584	-2.460589	0.656153	-1.339274
C	-1.559762	-1.736322	-1.300144	-1.702622	-1.467846	-1.387314	-1.844633	-1.851606	-1.639270	-1.846327	-1.808598	-1.626812
C	-1.707771	-1.781115	-2.835481	-1.673530	-1.606706	-2.923857	-2.018452	-1.885735	-3.172046	-2.022387	-1.836932	-3.159438
C	-2.049224	-3.165411	-3.320601	-2.226411	-2.933814	-3.372486	-2.371216	-3.266047	-3.660493	-2.373578	-3.215828	-3.652939
C	-1.326495	-3.979892	-4.105322	-1.595488	-3.931114	-4.011889	-1.663583	-4.076505	-4.462921	-1.667162	-4.020788	-4.461951
C	-1.844371	-5.348909	-4.480190	-2.330061	-5.200415	-4.375525	-2.190542	-5.441917	-4.838302	-2.192524	-5.385509	-4.842087
C	0.025443	-3.644521	-4.687001	-0.145798	-3.908223	-4.431941	-0.321086	-3.739769	-5.065317	-0.327836	-3.677979	-5.067979
C	0.075376	-1.498815	1.299237	-0.373032	-1.340585	1.388312	-0.143673	-1.622726	0.928060	-0.142386	-1.587530	0.939149
H	1.279651	4.433166	-1.548939	2.332254	4.000800	-1.598345	1.933060	6.354496	0.145926	1.932314	6.382901	0.169696
H	1.412345	5.961002	-0.687048	2.663318	5.539192	-0.811845	3.344815	5.322400	0.346014	3.333079	5.329626	0.332978
H	2.623695	4.692266	-0.417760	3.555095	4.088069	-0.313606	1.956159	4.741497	-0.594518	1.918415	4.776648	-0.585598
H	-1.116591	4.172870	-0.602522	-0.163677	4.280634	-0.967581	1.688186	6.532423	2.749530	1.745157	6.540711	2.778008
H	-1.378046	4.075790	1.145416	-0.647246	4.375667	0.732583	1.580897	5.056614	3.720181	1.630361	5.058337	3.738275
H	-1.001094	5.627247	0.378600	0.121498	5.759864	-0.061120	3.105699	5.508940	2.944209	3.147765	5.490787	2.938366
H	2.365723	3.605824	1.580537	2.847249	3.236364	1.689897	0.683085	3.204604	0.524392	0.646946	3.246506	0.544328
H	0.916751	3.668896	3.691488	1.199588	3.744053	3.581792	0.341876	3.442146	3.593226	0.355922	3.470461	3.619282
H	-0.475955	3.182341	2.732513	-0.138657	3.460080	2.474761	-0.904420	2.929266	2.461046	-0.904937	2.960786	2.501553
H	2.395002	2.000507	4.604100	2.198228	1.886298	4.779632	1.988774	1.821054	4.414288	2.002548	1.830495	4.439594
H	4.437720	0.107121	6.492531	3.098059	-2.078193	6.568376	4.060061	-0.073325	6.268879	3.992161	-1.949482	5.652110
H	2.067256	-2.119724	3.435558	1.177163	-2.165350	3.784553	1.832208	-2.258117	3.078012	1.828662	-2.236114	3.068173
H	-1.942122	-0.637898	1.283504	-2.160499	-0.112716	1.057873	-2.179830	-0.810551	0.948902	-2.179533	-0.777917	0.965678
H	0.395928	0.793274	-0.884389	0.658627	0.663341	-0.858631	0.128829	0.667466	-1.252480	0.124971	0.711247	-1.233498
H	-2.247406	0.885760	-2.130642	-1.744062	1.161946	-2.445919	-2.536173	0.778931	-2.439140	-2.540837	0.824473	-2.416110
H	-3.187381	0.450428	-0.686899	-2.917428	1.033855	-1.117656	-3.447857	0.345990	-0.976464	-3.450883	0.384854	-0.954388
H	-1.909725	1.672441	-0.578554	-1.441949	1.991301	-0.908812	-2.159426	1.557382	-0.891888	-2.163604	1.597146	-0.866087
H	-0.802960	-2.474446	-1.012165	-1.149459	-2.312334	-0.961351	-1.085425	-2.593858	-1.368068	-1.085883	-2.551084	-1.359616
H	-2.501922	-2.047865	-0.827608	-2.737463	-1.552973	-1.026921	-2.779840	-2.162690	-1.153043	-2.780499	-2.122583	-1.140445
H	-2.507983	-1.094089	-3.144676	-2.276886	-0.805660	-3.373549	-2.822187	-1.194831	-3.462913	-2.827742	-1.146221	-3.446226
H	-0.784637	-1.407544	-3.285394	-0.647474	-1.455074	-3.267905	-1.102259	-1.511327	-3.635192	-1.107556	-1.459050	-3.622452
H	-3.012058	-3.541592	-2.967086	-3.279447	-3.089121	-3.126417	-3.328695	-3.642673	-3.293240	-3.328884	-3.596380	-3.284052
H	-2.825829	-5.550515	-4.039258	-3.376243	-5.174649	-4.054327	-3.164917	-5.644592	-4.382420	-3.164471	-5.592818	-4.383110
H	-1.933839	-5.453725	-5.570845	-2.312151	-5.371355	-5.461327	-2.298410	-5.539107	-5.927991	-2.304878	-5.477339	-5.931783
H	-1.154000	-6.137942	-4.149614	-1.854491	-6.078921	-3.916654	-1.496361	-6.234522	-4.524681	-1.495159	-6.178130	-4.535600
H	0.391398	-2.658413	-4.393149	0.373795	-2.990921	-4.146891	0.052529	-2.756943	-4.770098	0.044637	-2.695727	-4.769430
H	0.773966	-4.386768	-4.376372	0.397899	-4.753969	-3.988263	0.430735	-4.486365	-4.773688	0.426904	-4.424267	-4.783109
H	-0.005853	-3.679256	-5.785125	-0.056337	-4.021826	-5.521501	-0.371573	-3.765660	-6.162967	-0.382639	-3.698540	-6.165527
H	0.754013	-1.776864	0.483745	0.327444	-1.808515	0.685896	0.548102	-1.848167	0.107224	0.547780	-1.809505	0.115939
H	-0.398246	-2.410697	1.674881	-1.061716	-2.111136	1.747693	-0.590893	-2.562165	1.266005	-0.588723	-2.528529	1.274255

	5a-5			5a-6			5a-7			5a-8		
**Element**	**X**	**Y**	**Z**	**X**	**Y**	**Z**	**X**	**Y**	**Z**	**X**	**Y**	**Z**
C	3.100233	4.233853	-0.618181	2.058595	4.802163	-0.509661	2.038458	5.545797	-0.126312	2.059896	5.523727	-0.122595
C	2.005749	3.980682	0.392860	1.186954	4.236206	0.587653	1.711567	4.924738	1.211861	1.705014	4.903976	1.209035
C	0.649303	4.504186	-0.011925	-0.289597	4.489155	0.404392	2.197165	5.718833	2.399879	2.144611	5.711393	2.406052
C	2.277795	3.355149	1.548617	1.735414	3.587756	1.626780	1.043026	3.763799	1.278245	1.051957	3.733616	1.262908
C	1.337826	3.030311	2.688964	1.045669	2.972757	2.824900	0.628472	2.986609	2.507661	0.613973	2.955643	2.483598
C	1.356630	1.556428	3.043575	1.411652	1.513071	3.009560	1.266823	1.610605	2.566408	1.251587	1.579750	2.553631
C	2.051061	1.051814	4.145108	2.338234	1.081269	3.963650	2.373508	1.325340	3.369537	2.352943	1.299739	3.369000
C	2.090528	-0.319792	4.444558	2.693703	-0.270793	4.100871	2.960125	0.050610	3.413950	2.936345	0.023602	3.421886
C	2.849531	-0.741074	5.642537	3.666898	-0.728178	5.116824	4.121702	-0.141032	4.310463	4.093624	-0.275296	4.294116
O	2.822112	-2.087703	5.847504	4.168343	0.283516	5.878544	4.615340	-1.410495	4.282903	4.518875	0.799084	5.015031
O	3.450995	0.002926	6.394629	4.012913	-1.882509	5.287487	4.617755	0.717255	5.016216	4.640551	-1.358561	4.387144

C	1.425493	-1.244162	3.617265	2.123208	-1.241591	3.256864	2.443530	-0.995916	2.626363	2.423938	-1.023960	2.633453
C	0.724174	-0.766032	2.523222	1.198979	-0.838423	2.310212	1.345227	-0.737182	1.824227	1.334218	-0.770653	1.820789
C	0.695808	0.605295	2.259184	0.857525	0.512511	2.205455	0.784264	0.542149	1.805368	0.774534	0.509693	1.792623
O	-0.045467	0.921841	1.158100	-0.075013	0.754022	1.239100	-0.291484	0.639499	0.967106	-0.294025	0.604957	0.945184
C	-0.742956	-0.289931	0.709106	-0.567190	-0.537650	0.741842	-0.655731	-0.716501	0.536748	-0.656518	-0.753023	0.517770
C	-0.705269	-0.290790	-0.835038	-0.741728	-0.399794	-0.786636	-1.097097	-0.634622	-0.940193	-1.087464	-0.676625	-0.962483
O	0.662493	-0.294204	-1.254181	0.527514	-0.072023	-1.359376	0.006947	-0.171649	-1.724497	0.022047	-0.216786	-1.740893
C	-1.419774	0.961474	-1.372143	-1.762692	0.709368	-1.094360	-2.284577	0.333773	-1.076136	-2.273710	0.291570	-1.110443
C	-1.335735	-1.590100	-1.369979	-1.161887	-1.754659	-1.386324	-1.436532	-2.046795	-1.453368	-1.423670	-2.090686	-1.472573
C	-1.341400	-1.722634	-2.907502	-1.350487	-1.755026	-2.918008	-1.876993	-2.112015	-2.930878	-1.853856	-2.161444	-2.952852
C	-1.752549	-3.103937	-3.343905	-1.528260	-3.150661	-3.454737	-1.961997	-3.532926	-3.422142	-1.936141	-3.584265	-3.439038
C	-2.829126	-3.477400	-4.053062	-2.593287	-3.686365	-4.071271	-3.037248	-4.213157	-3.849336	-3.008682	-4.265552	-3.871318
C	-3.065157	-4.931604	-4.388702	-2.572858	-5.123713	-4.536759	-2.911152	-5.648486	-4.304273	-2.880204	-5.702831	-4.319373
C	-3.885043	-2.538260	-4.584316	-3.880454	-2.955641	-4.368622	-4.438119	-3.656446	-3.931693	-4.408561	-3.708319	-3.966190
C	-0.027808	-1.466651	1.416304	0.461275	-1.588136	1.226286	0.585479	-1.591290	0.836384	0.581551	-1.627616	0.830206
H	2.862114	3.770459	-1.586137	1.788141	4.381649	-1.488603	1.601073	6.550354	-0.214414	1.609951	6.521030	-0.228135
H	3.217742	5.309493	-0.811714	1.929176	5.890387	-0.594974	3.123733	5.668070	-0.250166	3.146245	5.661974	-0.216901
H	4.065982	3.843187	-0.282792	3.119983	4.600454	-0.334817	1.668423	4.940982	-0.960211	1.722601	4.909353	-0.963303
H	0.362967	4.114130	-0.998389	-0.624196	4.129943	-0.578556	1.762525	6.728119	2.394393	1.691899	6.712492	2.384851
H	-0.144060	4.245430	0.692552	-0.910163	4.009206	1.164129	1.957944	5.258467	3.361105	1.887704	5.250571	3.362580
H	0.670444	5.598895	-0.108457	-0.502799	5.567203	0.426749	3.287030	5.850819	2.355027	3.232707	5.863632	2.389402
H	3.306077	3.033004	1.718664	2.820931	3.479741	1.638446	0.754561	3.293188	0.337658	0.796251	3.254840	0.317004
H	1.641845	3.600145	3.578080	1.345195	3.518487	3.730748	0.875987	3.530904	3.422985	0.842799	3.500153	3.403841
H	0.314309	3.337362	2.453048	-0.041269	3.070557	2.744229	-0.463742	2.864118	2.495868	-0.477701	2.832409	2.450583
H	2.578957	1.731057	4.808121	2.792349	1.812678	4.624552	2.801788	2.102584	3.995351	2.764101	2.090441	3.988082
H	3.351664	-2.234773	6.652506	4.787287	-0.145150	6.497611	5.366545	-1.406491	4.903999	5.273872	0.473076	5.538291
H	1.468059	-2.304155	3.842937	2.421063	-2.278880	3.371439	2.902935	-1.977842	2.658088	2.894313	-2.000558	2.685698
H	-1.779864	-0.197395	1.057034	-1.543825	-0.699853	1.215793	-1.505808	-1.020643	1.161331	-1.511350	-1.053433	1.137620
H	1.072646	0.488769	-0.847511	0.822329	0.742669	-0.916683	0.260863	0.691560	-1.356455	0.274213	0.647199	-1.373536
H	-1.300681	1.026555	-2.456712	-1.808845	0.890025	-2.171369	-2.536331	0.475697	-2.130151	-2.518135	0.429392	-2.166726
H	-2.491412	0.940279	-1.140143	-2.765578	0.437652	-0.743550	-3.170884	-0.046091	-0.554011	-3.163730	-0.085991	-0.592979
H	-0.996582	1.866162	-0.925274	-1.475287	1.643065	-0.601511	-2.035873	1.309658	-0.647931	-2.027645	1.269091	-0.684403
H	-0.780380	-2.438663	-0.952845	-0.393968	-2.495395	-1.133318	-0.548527	-2.678397	-1.329483	-0.536713	-2.721949	-1.340047
H	-2.364105	-1.669314	-0.992735	-2.091283	-2.085558	-0.903781	-2.222082	-2.477811	-0.818497	-2.213714	-2.519084	-0.841525
H	-1.992829	-0.961891	-3.347470	-2.196696	-1.118304	-3.192269	-2.831635	-1.594001	-3.061196	-2.807308	-1.643484	-3.091909
H	-0.325482	-1.514021	-3.267757	-0.456997	-1.302711	-3.367990	-1.136944	-1.563698	-3.528428	-1.109338	-1.615885	-3.547374
H	-1.072046	-3.894793	-3.020786	-0.663696	-3.800495	-3.302611	-1.008587	-4.065720	-3.413216	-0.983181	-4.117587	-3.420898
H	-2.278113	-5.577210	-3.986358	-1.623692	-5.616021	-4.302633	-1.881490	-6.012274	-4.229664	-1.851340	-6.066894	-4.235837
H	-4.028485	-5.279710	-3.989364	-3.381572	-5.703631	-4.069509	-3.551129	-6.312182	-3.705469	-3.524833	-6.363643	-3.722377
H	-3.107623	-5.085697	-5.476355	-2.731547	-5.191222	-5.622490	-3.237102	-5.763717	-5.347825	-3.198753	-5.822229	-5.364748
H	-3.709240	-1.492746	-4.320758	-3.886050	-1.922706	-4.012942	-4.516767	-2.624005	-3.583376	-4.489104	-2.674426	-3.622620
H	-3.945155	-2.601816	-5.679893	-4.076026	-2.938457	-5.450016	-4.808806	-3.690077	-4.965808	-4.771695	-3.745900	-5.002855
H	-4.877713	-2.818081	-4.204536	-4.734044	-3.473686	-3.909338	-5.131905	-4.265835	-3.335713	-5.107079	-4.314882	-3.372859
H	0.670380	-1.956770	0.726790	1.144287	-1.856081	0.411003	1.171377	-1.743608	-0.078428	1.174899	-1.783545	-0.079112
H	-0.738862	-2.217577	1.773656	-0.026612	-2.502192	1.577598	0.304419	-2.572608	1.230160	0.297289	-2.607266	1.225534

		5a-9			5a-10			5a-11			5a-12	
**Element**	**X**	**Y**	**Z**	**X**	**Y**	**Z**	**X**	**Y**	**Z**	**X**	**Y**	**Z**
C	-2.431279	3.563754	4.642479	-2.440278	3.524268	4.632995	-1.726638	4.246170	4.075377	-2.611277	3.984363	3.588869
C	-1.021606	3.678928	4.110366	-1.034440	3.648499	4.092846	-0.385127	4.306617	3.382361	-1.452158	3.847998	2.629261
C	-0.121793	4.575040	4.925957	-0.141561	4.565987	4.892257	0.546265	5.363288	3.924407	-1.791454	4.164557	1.193708
C	-0.657976	3.017606	3.001513	-0.668060	2.977301	2.990862	-0.102304	3.466881	2.375389	-0.242046	3.476019	3.072516
C	0.692608	3.000404	2.320699	0.679112	2.966514	2.303281	1.171919	3.368799	1.566291	1.033920	3.287551	2.282394
C	1.302144	1.610469	2.280937	1.297115	1.580194	2.264237	1.842100	2.013622	1.705500	1.576154	1.875148	2.398294
C	2.326226	1.210525	3.142245	2.325820	1.189001	3.126968	2.964038	1.805515	2.510808	2.580613	1.529605	3.308091
C	2.870703	-0.083394	3.111350	2.875638	-0.102902	3.094545	3.565428	0.544221	2.648944	3.049791	0.212268	3.438557
C	3.951686	-0.397674	4.071741	3.962347	-0.517016	4.009388	4.749059	0.439634	3.530827	4.108216	-0.153108	4.405500
O	4.403990	-1.678584	3.968173	4.363832	0.472624	4.854553	5.250715	-0.824807	3.605667	4.567328	0.905292	5.129423
O	4.418996	0.376114	4.886405	4.474318	-1.620833	4.036123	5.255555	1.360329	4.144484	4.552558	-1.274327	4.568956
C	2.393290	-1.031994	2.187194	2.400949	-1.052832	2.170828	3.046926	-0.568222	1.959376	2.517473	-0.811071	2.632663
C	1.374643	-0.659524	1.326466	1.380611	-0.689658	1.310755	1.931959	-0.386774	1.158703	1.523682	-0.492613	1.725115
C	0.851121	0.633886	1.388378	0.850024	0.601433	1.373122	1.354085	0.880105	1.049193	1.070563	0.825861	1.625072
O	-0.145494	0.849672	0.477351	-0.149545	0.811297	0.464166	0.263271	0.898976	0.224997	0.095154	0.982975	0.681919
C	-0.498975	-0.442210	-0.125494	-0.496832	-0.483298	-0.137796	-0.083670	-0.490017	-0.102834	-0.329026	-0.352028	0.238961
C	-0.793301	-0.192927	-1.620532	-0.795483	-0.235912	-1.632260	-0.536878	-0.520960	-1.578300	-0.603379	-0.275543	-1.278582
O	0.392628	0.313263	-2.242379	0.385957	0.277854	-2.256415	0.554305	-0.097835	-2.402105	0.609470	0.091030	-1.945066
C	-1.938452	0.824417	-1.762301	-1.947347	0.774156	-1.771975	-1.740177	0.417374	-1.773207	-1.695716	0.770538	-1.559146
C	-1.113940	-1.532289	-2.310655	-1.108997	-1.577339	-2.321662	-0.857788	-1.970999	-1.987004	-0.987649	-1.672639	-1.801853
C	-1.363190	-1.436187	-3.830354	-1.361029	-1.483050	-3.841031	-1.306329	-2.147892	-3.452892	-1.222849	-1.753414	-3.324751
C	-1.612032	-2.791052	-4.439158	-1.603120	-2.839522	-4.449005	-1.373522	-3.601297	-3.840947	-1.535540	-3.159470	-3.764605
C	-0.876660	-3.447967	-5.349754	-0.864003	-3.493551	-5.358635	-2.440797	-4.324884	-4.213401	-0.826520	-3.960461	-4.575274
C	-1.294620	-4.815740	-5.837040	-1.274790	-4.863881	-5.844820	-2.296349	-5.787200	-4.565322	-1.309323	-5.355964	-4.895280
C	0.394496	-2.925786	-5.974268	0.404987	-2.965686	-5.982757	-3.849624	-3.794741	-4.328646	0.475372	-3.584040	-5.240048
C	0.685539	-1.385681	0.194926	0.693461	-1.419946	0.180882	1.171992	-1.323683	0.249160	0.798959	-1.313201	0.684818

H	-2.435138	3.167598	5.667822	-2.435198	3.141455	5.663409	-1.607662	4.042667	5.149018	-3.018700	5.005197	3.575450
H	-2.919390	4.547612	4.688404	-2.939513	4.502968	4.668280	-2.256143	5.206422	3.998647	-3.438118	3.317123	3.306902
H	-3.049979	2.906965	4.022948	-3.054060	2.852398	4.024923	-2.370926	3.469042	3.652355	-2.321207	3.748821	4.617571
H	-0.055536	4.214360	5.961751	-0.068530	4.221821	5.933281	0.733713	5.199114	4.994572	-2.141065	5.202038	1.097292
H	0.895360	4.647328	4.534266	0.873434	4.643357	4.495732	1.514222	5.394348	3.419113	-0.951911	4.027672	0.508982
H	-0.533414	5.592642	4.978414	-0.563528	5.579961	4.930849	0.092050	6.360353	3.839236	-2.614566	3.526216	0.844243
H	-1.408602	2.398547	2.510186	-1.413942	2.344018	2.510631	-0.866707	2.745993	2.085366	-0.135950	3.268729	4.138271
H	0.571732	3.361865	1.289327	0.550646	3.324113	1.271495	0.929960	3.535013	0.506675	0.883480	3.538703	1.228399
H	1.394976	3.680090	2.810653	1.379416	3.652789	2.787336	1.885666	4.146728	1.850449	1.797511	3.979431	2.664508
H	2.724849	1.911185	3.869806	2.709385	1.904387	3.847120	3.397431	2.636668	3.059007	3.009842	2.303776	3.936213
H	5.103993	-1.757990	4.641805	5.071763	0.075957	5.394544	6.016206	-0.761258	4.205729	5.249983	0.535251	5.718618
H	2.823343	-2.027219	2.158544	2.847630	-2.041768	2.156310	3.520378	-1.538817	2.059532	2.902032	-1.820130	2.740894
H	-1.412655	-0.778758	0.381380	-1.407708	-0.824486	0.370965	-0.924710	-0.759459	0.549124	-1.261872	-0.571183	0.774311
H	0.629048	1.122661	-1.758379	0.618858	1.088028	-1.772048	0.792678	0.795442	-2.101434	0.889641	0.935509	-1.553137
H	-2.086913	1.082763	-2.813742	-2.099493	1.031304	-2.823181	-2.001859	0.479727	-2.832516	-1.824795	0.902949	-2.636270
H	-2.879258	0.424335	-1.365764	-2.884831	0.368199	-1.373535	-2.616709	0.062826	-1.217849	-2.657502	0.468403	-1.127596
H	-1.706481	1.742087	-1.213027	-1.720126	1.693436	-1.223370	-1.503198	1.425039	-1.418123	-1.422456	1.737042	-1.124881
H	-0.275735	-2.219194	-2.147858	-0.266438	-2.259104	-2.159967	0.040415	-2.579121	-1.824963	-0.186471	-2.374885	-1.545613
H	-1.989548	-1.978755	-1.818893	-1.981143	-2.029024	-1.828524	-1.632703	-2.367224	-1.317342	-1.887607	-2.010738	-1.269301
H	-2.241321	-0.801859	-4.015906	-2.242952	-0.853695	-4.025581	-2.269239	-1.654091	-3.612985	-2.066972	-1.104784	-3.597981
H	-0.510846	-0.931877	-4.292189	-0.512149	-0.974193	-4.304263	-0.578070	-1.633126	-4.093242	-0.343533	-1.352755	-3.835050
H	-2.509552	-3.292579	-4.069776	-2.498234	-3.345223	-4.079499	-0.412748	-4.119063	-3.798731	-2.460661	-3.565949	-3.349371
H	-2.219989	-5.155218	-5.360843	-2.198917	-5.207389	-5.369105	-1.261411	-6.130592	-4.469917	-2.255044	-5.587172	-4.394810
H	-1.451876	-4.817685	-6.925042	-1.430961	-4.867800	-6.932977	-2.924624	-6.415140	-3.917561	-1.456619	-5.485903	-5.976908
H	-0.514181	-5.563505	-5.636384	-0.490874	-5.607533	-5.642524	-2.624663	-5.981156	-5.596391	-0.569876	-6.110474	-4.591330
H	0.690277	-1.943663	-5.599240	0.695491	-1.981619	-5.608693	-3.941440	-2.741174	-4.054758	0.817466	-2.578585	-4.985605
H	1.227094	-3.619602	-5.792242	1.240912	-3.655021	-5.799066	-4.223701	-3.907407	-5.355923	1.269719	-4.289922	-4.959980
H	0.287906	-2.851887	-7.065689	0.298955	-2.893741	-7.074370	-4.532526	-4.369359	-3.687284	0.382869	-3.642163	-6.333713
H	1.348760	-1.470672	-0.674639	1.355997	-1.501553	-0.689449	1.750277	-1.539807	-0.657574	1.459986	-1.541359	-0.160242
H	0.342834	-2.389675	0.462531	0.357437	-2.425728	0.449808	0.907635	-2.274808	0.720947	0.400133	-2.256046	1.070585

	5a-13			5a-14			5a-15			5a-16		
**Element**	**X**	**Y**	**Z**	**X**	**Y**	**Z**	**X**	**Y**	**Z**	**X**	**Y**	**Z**
C	-2.599638	4.016126	3.618157	1.251268	3.999965	0.270420	-2.216033	3.875636	3.569128	-2.182147	3.932498	3.584558
C	-1.440644	3.877296	2.658726	0.784939	3.381495	1.567941	-1.156899	3.811498	2.493424	-1.126506	3.857263	2.506125
C	-1.777829	4.199142	1.223830	1.235020	4.119987	2.804821	-1.677767	4.021380	1.093154	-1.649623	4.067032	1.106653
C	-0.232281	3.499404	3.101735	0.034766	2.269564	1.559803	0.126014	3.587234	2.814419	0.155634	3.624805	2.824354
C	1.043576	3.308703	2.311964	-0.521421	1.502759	2.738926	1.324258	3.491196	1.896342	1.350809	3.518493	1.903400
C	1.578348	1.893106	2.422195	-0.017188	0.071907	2.787551	2.026026	2.150660	2.009238	2.041368	2.172226	2.015933
C	2.576867	1.535792	3.331218	1.020777	-0.334404	3.627942	3.156046	1.960847	2.811163	3.168532	1.970594	2.815844
C	3.041262	0.216706	3.458157	1.486930	-1.658770	3.659985	3.776905	0.707988	2.943095	3.781327	0.714192	2.948882
C	4.098977	-0.047638	4.458345	2.586742	-1.979410	4.596231	4.968810	0.507212	3.796207	4.967571	0.616336	3.827668
O	4.474104	-1.356439	4.507548	2.965620	-3.287335	4.548997	5.387530	1.645493	4.415896	5.482611	-0.643230	3.893382
O	4.609469	0.786289	5.182805	3.126565	-1.189305	5.347861	5.547312	-0.551685	3.955464	5.465443	1.538401	4.446370
C	2.507786	-0.802098	2.646749	0.910143	-2.632550	2.823599	3.273204	-0.407466	2.248774	3.270208	-0.398963	2.255643
C	1.516921	-0.471421	1.737903	-0.121553	-2.253002	1.981396	2.156828	-0.243351	1.449131	2.153320	-0.223050	1.456412
C	1.070252	0.849013	1.642441	-0.561567	-0.927056	1.973731	1.555199	1.014038	1.345205	1.561573	1.038396	1.352185
O	0.098665	1.014941	0.696661	-1.582377	-0.705271	1.097130	0.470778	1.017852	0.514881	0.477187	1.050405	0.521686
C	-0.330105	-0.315464	0.246098	-1.981041	-1.986627	0.503740	0.147163	-0.376048	0.182786	0.142730	-0.340454	0.189888
C	-0.598478	-0.230892	-1.272114	-2.183429	-1.762976	-1.011107	-0.301366	-0.412857	-1.293957	-0.305641	-0.374328	-1.287009
O	0.618855	0.132071	-1.932404	-3.175076	-0.735470	-1.149910	0.782326	0.037260	-2.113495	0.782286	0.065811	-2.106376
C	-1.684224	0.822155	-1.552002	-2.803458	-3.019857	-1.626743	-1.525353	0.497699	-1.491209	-1.521640	0.546614	-1.485170
C	-0.988091	-1.623607	-1.803038	-0.873600	-1.334448	-1.713365	-0.588924	-1.868607	-1.707512	-0.605545	-1.827782	-1.699865
C	-1.218878	-1.696159	-3.327002	-1.045209	-0.938236	-3.194462	-1.028542	-2.051778	-3.175389	-1.046571	-2.007980	-3.167678
C	-1.536451	-3.098673	-3.774563	0.245585	-0.448385	-3.795716	-1.062496	-3.505513	-3.566593	-1.092831	-3.461530	-3.558290
C	-0.830068	-3.897913	-4.589260	0.532157	0.765763	-4.290950	-2.112704	-4.252225	-3.941867	-2.149351	-4.199530	-3.933096
C	-1.318179	-5.289680	-4.917408	1.903941	1.067911	-4.847567	-1.934979	-5.710313	-4.296057	-1.983947	-5.659160	-4.286913
C	0.473535	-3.522777	-5.251420	-0.434124	1.923805	-4.354388	-3.532927	-3.753615	-4.058064	-3.565348	-3.689039	-4.049217
C	0.791898	-1.284031	0.691353	-0.926756	-3.028989	0.964239	1.415237	-1.189983	0.535407	1.404446	-1.164264	0.542775
H	-3.002456	5.038823	3.607401	0.889809	5.033339	0.171473	-2.729350	4.847615	3.562347	-2.688037	4.908357	3.576380
H	-3.429397	3.353385	3.334027	2.348519	4.049794	0.229108	-2.991960	3.114387	3.405349	-2.964369	3.176721	3.425215
H	-2.310979	3.776581	4.646314	0.904938	3.434172	-0.600298	-1.795354	3.721599	4.567698	-1.759754	3.778308	4.582362
H	-2.122590	5.238481	1.129935	0.870365	5.156537	2.790591	-2.144384	5.011815	0.997220	-2.108079	5.061089	1.008770
H	-0.938511	4.060233	0.539215	0.895905	3.660956	3.736221	-0.905410	3.938054	0.325705	-0.880106	3.974694	0.337373
H	-2.603743	3.565559	0.872221	2.331653	4.178117	2.840902	-2.460315	3.286110	0.860481	-2.439089	3.337716	0.878508
H	-0.127247	3.289285	4.166984	-0.214300	1.836490	0.590494	0.365539	3.444608	3.869094	0.397325	3.483909	3.878712
H	0.895604	3.565344	1.258930	-0.277834	1.996165	3.683657	1.036804	3.669934	0.856111	1.062535	3.698785	0.863653
H	1.810156	3.994400	2.698646	-1.618032	1.484015	2.664894	2.044178	4.277570	2.163015	2.077990	4.298442	2.168422
H	3.018763	2.292826	3.972622	1.491326	0.384815	4.291629	3.565176	2.808397	3.351887	3.597537	2.804501	3.363856
H	5.166310	-1.398621	5.192502	3.685741	-3.368798	5.200825	6.167069	1.380659	4.937702	6.248552	-0.575541	4.492417
H	2.875869	-1.818242	2.737742	1.272703	-3.654440	2.849430	3.774526	-1.364095	2.355247	3.751724	-1.366187	2.350167
H	-1.265861	-0.533064	0.777031	-2.964854	-2.213240	0.929456	-0.690892	-0.660033	0.832283	-0.697584	-0.618071	0.839258
H	0.901201	0.974193	-1.536933	-2.857511	0.023749	-0.632061	1.008288	0.929233	-1.799615	1.014580	0.956829	-1.794364
H	-1.808443	0.960244	-2.628985	-3.105937	-2.821886	-2.658214	-1.784119	0.556751	-2.551453	-1.779414	0.607501	-2.545551

H	-2.649228	0.523066	-1.125554	-2.096339	-3.856250	-1.627445	-2.395476	0.120724	-0.940426	-2.395204	0.177334	-0.934634
H	-1.407661	1.785163	-1.112144	-3.699014	-3.315484	-1.069918	-1.313999	1.509579	-1.132314	-1.301818	1.556741	-1.126484
H	-0.191224	-2.331055	-1.547519	-0.455094	-0.479022	-1.168575	0.321668	-2.457394	-1.544009	0.300010	-2.424254	-1.535996
H	-1.891432	-1.959620	-1.274902	-0.130083	-2.139117	-1.638612	-1.357246	-2.283372	-1.041473	-1.377442	-2.235652	-1.033693
H	-2.059281	-1.042493	-3.599670	-1.389669	-1.812744	-3.765271	-2.001485	-1.578821	-3.337917	-2.015452	-1.526880	-3.330459
H	-0.336252	-1.296920	-3.832675	-1.840152	-0.191721	-3.266711	-0.309467	-1.519950	-3.812178	-0.323018	-1.482497	-3.804659
H	-2.463492	-3.503814	-3.362293	1.045890	-1.191731	-3.811161	-0.090431	-4.001644	-3.523708	-0.124947	-3.965819	-3.515500
H	-2.264953	-5.520081	-4.418556	2.572075	0.203392	-4.781357	-0.892766	-6.030560	-4.199539	-0.944429	-5.988133	-4.190522
H	-1.465692	-5.412814	-5.999797	1.845543	1.372563	-5.902227	-2.549989	-6.353311	-3.650342	-2.604255	-6.296807	-3.640957
H	-0.581760	-6.048845	-4.617671	2.374716	1.903356	-4.310059	-2.257248	-5.909798	-5.327988	-2.308052	-5.856196	-5.318732
H	0.819676	-2.520315	-4.990736	-1.421298	1.687133	-3.951378	-3.648359	-2.702391	-3.784032	-3.671921	-2.636845	-3.775355
H	1.264931	-4.233676	-4.975654	-0.040786	2.786933	-3.798778	-3.903686	-3.874379	-5.085628	-3.937209	-3.806855	-5.076719
H	0.381030	-3.573721	-6.345437	-0.567525	2.260862	-5.391898	-4.203324	-4.343437	-3.417292	-4.240608	-4.273105	-3.408284
H	1.454276	-1.511547	-0.152901	-0.299666	-3.376379	0.134496	2.000096	-1.393752	-0.369916	1.987277	-1.372948	-0.362808
H	0.386736	-2.226704	1.071186	-1.410631	-3.913473	1.391637	1.166287	-2.146642	1.004118	1.147361	-2.118891	1.011478

	**5a-17**			**5a-18**			**5a-19**			**5a-20**		
**Element**	**X**	**Y**	**Z**	**X**	**Y**	**Z**	**X**	**Y**	**Z**	**X**	**Y**	**Z**
C	1.251274	3.978154	0.265442	1.371473	4.349662	0.403309	1.342360	4.342787	0.400235	-2.779723	2.825182	4.449945
C	0.771935	3.360811	1.558737	0.870001	3.747676	1.695395	0.833008	3.736672	1.687300	-1.429904	2.806427	3.770874
C	1.190449	4.113236	2.798382	1.341195	4.463573	2.937681	1.262216	4.473499	2.932655	-0.437413	3.793333	4.335714
C	0.036985	2.238805	1.545208	0.074424	2.667842	1.678791	0.065453	2.636855	1.664363	-1.191900	1.963271	2.755174
C	-0.529292	1.471447	2.719120	-0.521510	1.921874	2.851793	-0.533590	1.883841	2.831212	0.081362	1.792585	1.956594
C	-0.022224	0.041725	2.773832	-0.068808	0.474045	2.906288	-0.066484	0.440653	2.888724	0.644021	0.386362	2.058163
C	1.016290	-0.357764	3.619804	0.953940	0.034968	3.749058	0.963962	0.015667	3.732367	1.739132	0.068857	2.866231
C	1.482788	-1.682154	3.655022	1.372570	-1.304878	3.787742	1.393382	-1.320975	3.771391	2.241466	-1.239558	2.962517
C	2.576569	-2.107157	4.556007	2.459771	-1.659948	4.726341	2.478050	-1.772891	4.670341	3.402165	-1.575658	3.816037
O	3.065302	-1.096349	5.326535	2.791383	-2.980850	4.686084	2.998201	-0.772994	5.434476	3.916183	-0.501345	4.476299
O	3.024174	-3.236506	4.631367	3.027292	-0.885823	5.474179	2.893656	-2.914160	4.749114	3.880781	-2.687551	3.942885
C	0.907891	-2.657499	2.819641	0.761882	-2.261522	2.955565	0.788478	-2.283239	2.941942	1.643347	-2.282660	2.232377
C	-0.121456	-2.285422	1.974021	-0.255449	-1.849502	2.111295	-0.233366	-1.885967	2.098847	0.556004	-1.992665	1.427817
C	-0.563257	-0.959508	1.961734	-0.648057	-0.508603	2.096861	-0.638209	-0.548137	2.082614	0.077436	-0.681976	1.356134
O	-1.582083	-0.741478	1.082074	-1.659890	-0.255502	1.217898	-1.653891	-0.306025	1.205193	-0.997708	-0.546475	0.528402
C	-1.977963	-2.025580	0.491577	-2.116093	-1.528890	0.649020	-2.100407	-1.585331	0.640709	-1.366046	-1.869000	0.008468
C	-2.177134	-1.806797	-1.024352	-2.353449	-1.314686	-0.861510	-2.344567	-1.376977	-0.869492	-1.580843	-1.734562	-1.515765
O	-3.169712	-0.780789	-1.168693	-3.322042	-0.263988	-0.985731	-3.323331	-0.335518	-0.993258	-2.600766	-0.743408	-1.705365
C	-2.794233	-3.066389	-1.637369	-3.020853	-2.561360	-1.447790	-3.002235	-2.631341	-1.450378	-2.169145	-3.039558	-2.057937
C	-0.866341	-1.378765	-1.725061	-1.053087	-0.927616	-1.604179	-1.050430	-0.979530	-1.617441	-0.286529	-1.307525	-2.246553
C	-1.034895	-0.989970	-3.208477	-1.251316	-0.571380	-3.091826	-1.256538	-0.631385	-3.105947	-0.469525	-1.022792	-3.751612
C	0.255605	-0.497019	-3.807770	-0.008619	0.030084	-3.692214	-0.022622	-0.017345	-3.711700	0.799733	-0.516232	-4.384007
C	0.536995	0.714777	-4.311685	0.747865	-0.429105	-4.701273	0.739180	-0.473357	-4.718198	1.022399	0.659446	-4.992022
C	1.909287	1.020594	-4.865061	1.971371	0.331864	-5.156232	1.952193	0.300580	-5.179314	2.382480	0.987355	-5.562652
C	-0.436049	1.866306	-4.389111	0.482335	-1.697673	-5.475399	0.490162	-1.750801	-5.483140	-0.008966	1.745819	-5.178534
C	-0.923706	-3.065311	0.957748	-1.085045	-2.599782	1.094427	-1.058467	-2.645706	1.085564	-0.281611	-2.858503	0.515206
H	0.877170	5.005757	0.154150	1.054918	5.397685	0.305129	1.000440	5.381454	0.288589	-2.685641	2.601464	5.522040
H	2.348281	4.043075	0.241796	2.470109	4.352968	0.369811	2.440986	4.374635	0.386461	-3.244831	3.818714	4.380152
H	0.927333	3.402483	-0.607349	1.007527	3.802251	-0.471887	1.008913	3.780056	-0.477331	-3.468572	2.096825	4.010872
H	0.807057	5.142814	2.773921	1.020633	5.514578	2.923487	0.910242	5.514191	2.907001	-0.274324	3.605965	5.406130
H	0.845593	3.652515	3.726929	0.975997	4.016930	3.865213	0.894925	4.021443	3.856830	0.536333	3.766447	3.841202
H	2.285227	4.192134	2.850082	2.438990	4.475311	2.982191	2.358338	4.519278	2.994770	-0.823952	4.819019	4.256908
H	-0.190469	1.796973	0.574570	-0.185174	2.247403	0.706595	-0.166187	2.203793	0.690666	-1.999306	1.298539	2.447992
H	-0.297293	1.966805	3.665950	-0.270663	2.406984	3.798944	-0.297315	2.371572	3.780959	-0.132216	2.009218	0.899832
H	-1.625009	1.449795	2.633858	-1.617138	1.942110	2.765922	-1.628505	1.892438	2.735149	0.848633	2.504609	2.272670
H	1.473126	0.376690	4.275095	1.449319	0.740065	4.409831	1.443344	0.739664	4.383191	2.217735	0.857552	3.437552
H	3.769374	-1.502222	5.864738	3.507947	-3.084783	5.338637	3.692507	-1.196289	5.971946	4.662897	-0.852887	4.994903
H	1.284461	-3.674485	2.862362	1.087933	-3.295506	2.986170	1.136799	-3.310122	2.987185	2.050558	-3.285042	2.317821
H	-2.962541	-2.251652	0.915758	-3.094481	-1.719969	1.104284	-3.075585	-1.783810	1.099655	-2.342038	-2.094723	0.451081
H	-2.853975	-0.019661	-0.652562	-2.972729	0.493049	-0.485521	-2.979159	0.425971	-0.496288	-2.298777	0.052629	-1.235643
H	-3.095320	-2.871811	-2.669897	-3.350082	-2.362707	-2.470977	-3.336883	-2.438332	-2.472892	-2.486084	-2.905111	-3.095254
H	-2.085880	-3.901705	-1.634457	-2.332970	-3.413437	-1.463735	-2.306453	-3.476951	-1.466558	-1.438532	-3.854603	-2.021817
H	-3.690240	-3.361798	-1.081162	-3.905532	-2.832762	-0.862005	-3.882251	-2.909592	-0.860809	-3.050992	-3.330663	-1.477306
H	-0.451421	-0.519724	-1.183164	-0.613229	-0.064702	-1.086863	-0.618264	-0.109899	-1.104840	0.095871	-0.400382	-1.762275
H	-0.121165	-2.181278	-1.644395	-0.317050	-1.737663	-1.526246	-0.305682	-1.781408	-1.538329	0.487402	-2.075544	-2.115635
H	-1.373432	-1.868706	-3.776309	-1.567482	-1.457989	-3.650054	-1.563253	-1.523924	-3.659953	-0.770696	-1.950048	-4.260511
H	-1.832973	-0.247397	-3.286685	-2.078121	0.148299	-3.159067	-2.092082	0.078053	-3.173940	-1.297535	-0.320339	-3.876343
H	1.060398	-1.235620	-3.813804	0.307794	0.963272	-3.220648	0.281766	0.923133	-3.246708	1.641731	-1.209375	-4.321078
H	2.582533	0.160946	-4.788198	2.127035	1.243706	-4.571052	2.096048	1.218530	-4.600668	3.098781	0.174535	-5.406377
H	1.853593	1.315356	-5.922682	2.876908	-0.285657	-5.071070	2.865872	-0.304265	-5.090369	2.322393	1.184705	-6.642465
H	2.372502	1.863834	-4.333141	1.890148	0.617322	-6.214768	1.866605	0.577460	-6.239786	2.795513	1.897400	-5.104643
H	-1.423147	1.627352	-3.987263	-0.394660	-2.245940	-5.123635	-0.379603	-2.307872	-5.127323	-0.984303	1.494562	-4.755992
H	-0.049895	2.737006	-3.840302	0.333428	-1.477391	-6.541824	0.338489	-1.540192	-6.551128	0.328431	2.683629	-4.715208
H	-0.567976	2.192859	-5.430172	1.346966	-2.373730	-5.418149	1.363472	-2.415138	-5.420942	-0.152536	1.963993	-6.246103
H	-0.294102	-3.414815	0.130936	-0.470194	-2.954245	0.258404	-0.441962	-2.996010	0.249129	0.313584	-3.274805	-0.305852
H	-1.407061	-3.948879	1.387371	-1.587432	-3.475931	1.517309	-1.551082	-3.525702	1.511673	-0.737406	-3.704959	1.039738

	5a-21			5a-22			5a-23			5a-24		
**Element**	**X**	**Y**	**Z**	**X**	**Y**	**Z**	**X**	**Y**	**Z**	**X**	**Y**	**Z**
C	1.376267	5.454308	-0.619258	2.180440	5.062719	-1.417336	2.915860	5.437739	-0.582186	2.891923	5.470902	-0.570546
C	0.658517	4.643643	0.435127	1.323628	4.547852	-0.283809	2.287799	4.919048	0.690514	2.286524	4.943837	0.709671
C	-0.847189	4.712456	0.357407	-0.153440	4.810450	-0.449065	2.669743	5.690589	1.930223	2.706422	5.694385	1.949900
C	1.352648	3.953913	1.353586	1.884637	3.933393	0.769194	1.466078	3.859299	0.661419	1.451748	3.894197	0.686008
C	0.827080	3.108918	2.493390	1.210736	3.374260	2.003133	0.748171	3.193231	1.813880	0.752810	3.222490	1.846904
C	1.385186	1.699517	2.466863	1.557395	1.916593	2.234067	1.158011	1.742352	1.991618	1.166677	1.771352	2.012056
C	2.421305	1.280812	3.307285	2.497596	1.502583	3.179970	2.080004	1.332550	2.960241	2.093798	1.354967	2.970290
C	2.951643	-0.018648	3.248307	2.836184	0.151651	3.361429	2.453978	-0.012345	3.112095	2.473996	0.011024	3.112682
C	4.041538	-0.464101	4.143883	3.839321	-0.173170	4.399116	3.419734	-0.447105	4.145362	3.455414	-0.317698	4.170229
O	4.464358	0.504246	5.003347	4.093782	-1.507976	4.498614	3.895335	0.577587	4.906051	3.755419	-1.645377	4.228085
O	4.539336	-1.574778	4.144785	4.404150	0.635073	5.112315	3.780230	-1.594826	4.329965	3.967267	0.481933	4.931420
C	2.450570	-0.945650	2.315691	2.230894	-0.842073	2.569472	1.907511	-0.999967	2.270926	1.926147	-0.974403	2.269347
C	1.420781	-0.555789	1.478766	1.289800	-0.455815	1.630028	0.992564	-0.618318	1.306943	1.003166	-0.585584	1.313548
C	0.906988	0.740762	1.568522	0.968190	0.895464	1.481159	0.638681	0.728359	1.182998	0.645055	0.760295	1.200063
O	-0.111425	0.974054	0.691008	0.018157	1.116984	0.527068	-0.273937	0.949938	0.189442	-0.273960	0.985684	0.213121
C	-0.470598	-0.301886	0.056035	-0.505584	-0.184512	0.090612	-0.772311	-0.356065	-0.264898	-0.770578	-0.318067	-0.247732
C	-0.756207	-0.001145	-1.430735	-0.702664	-0.102008	-1.438104	-0.975606	-0.267065	-1.791127	-0.979931	-0.219794	-1.772645
O	0.421372	0.574497	-2.006632	0.562397	0.196664	-2.038223	0.287377	0.020707	-2.402554	0.280369	0.073516	-2.386988
C	-1.926485	0.987528	-1.561839	-1.710489	1.004873	-1.789099	-1.976678	0.847724	-2.135658	-1.983895	0.895651	-2.106327
C	-1.000050	-1.289188	-2.240339	-1.110948	-1.460972	-2.038520	-1.394981	-1.624248	-2.388623	-1.399553	-1.573770	-2.377213
C	-2.202682	-2.157252	-1.815644	-2.440405	-2.066323	-1.542277	-2.729668	-2.218581	-1.893308	-2.732890	-2.171828	-1.882820
C	-2.388503	-3.331633	-2.741253	-2.770964	-3.342509	-2.272000	-3.071056	-3.490589	-2.625439	-3.074183	-3.440519	-2.620681
C	-2.327048	-4.642461	-2.460931	-2.915568	-4.581726	-1.777990	-3.223748	-4.729722	-2.133641	-3.229237	-4.681525	-2.134374
C	-2.537083	-5.672323	-3.546383	-3.244043	-5.740571	-2.690196	-3.562373	-5.884195	-3.047668	-3.567956	-5.831738	-3.053715
C	-2.057895	-5.226763	-1.095309	-2.779921	-4.957459	-0.322224	-3.087812	-5.109536	-0.678969	-3.096465	-5.067506	-0.681019
C	0.710394	-1.258826	0.346356	0.514714	-1.234105	0.593105	0.268055	-1.389279	0.228435	0.274624	-1.351939	0.234282
H	1.101615	5.122645	-1.630592	1.888868	4.607371	-2.374386	2.639299	6.486281	-0.761846	2.624301	6.524893	-0.730820
H	1.099513	6.516283	-0.556886	2.059089	6.148053	-1.542241	4.012968	5.412545	-0.519988	3.989739	5.431800	-0.533437
H	2.463940	5.381931	-0.521680	3.242951	4.857971	-1.253274	2.612830	4.853024	-1.456412	2.562064	4.900995	-1.444893
H	-1.194567	4.443735	-0.649692	-0.507914	4.428165	-1.416230	2.379924	6.746181	1.833674	2.431395	6.755446	1.871778
H	-1.349477	4.054900	1.070106	-0.765416	4.357039	0.333580	2.214028	5.302945	2.844218	2.264676	5.302621	2.868877
H	-1.197466	5.738233	0.539874	-0.356291	5.890752	-0.452595	3.759663	5.680611	2.069469	3.798648	5.665360	2.065869
H	2.441462	3.994556	1.299147	2.968933	3.813707	0.765550	1.278055	3.393547	-0.306588	1.236791	3.442370	-0.283021
H	1.114713	3.573785	3.446941	1.538343	3.947438	2.881656	0.921612	3.728559	2.751463	0.941351	3.753201	2.784003
H	-0.266424	3.070016	2.480081	0.124022	3.486822	1.941092	-0.333697	3.228670	1.623350	-0.331990	3.258070	1.673795
H	2.824293	1.979403	4.033612	2.988824	2.237725	3.810756	2.513054	2.075291	3.622303	2.538544	2.081706	3.643509
H	5.172938	0.088726	5.528083	4.761996	-1.589035	5.203593	4.512358	0.162886	5.536448	4.403405	-1.729929	4.951307
H	2.882906	-1.940390	2.278439	2.503327	-1.883434	2.702397	2.215710	-2.032223	2.402026	2.226894	-2.010783	2.377567
H	-1.383081	-0.644692	0.558365	-1.471930	-0.307579	0.594141	-1.735413	-0.504126	0.238497	-1.731299	-0.472335	0.258376
H	0.637260	1.350209	-1.460031	0.874129	1.016504	-1.616517	0.606836	0.841928	-1.991263	0.599797	0.893272	-1.972704
H	-2.099620	1.211062	-2.619397	-1.819987	1.068403	-2.876458	-2.097087	0.909006	-3.221923	-2.109035	0.963249	-3.191686
H	-2.853060	0.589990	-1.133212	-2.696824	0.820022	-1.349483	-2.959609	0.673314	-1.684569	-2.964651	0.717331	-1.652056
H	-1.696465	1.922944	-1.042366	-1.359379	1.972836	-1.418318	-1.613699	1.814104	-1.771681	-1.620625	1.860291	-1.738127
H	-1.121753	-0.983248	-3.287172	-1.156059	-1.322293	-3.126172	-1.437750	-1.486990	-3.476530	-1.444625	-1.430070	-3.464189
H	-0.088374	-1.896452	-2.210522	-0.300305	-2.176907	-1.861954	-0.589661	-2.345716	-2.209443	-0.593386	-2.295802	-2.204021
H	-2.081468	-2.492665	-0.779872	-2.400259	-2.234868	-0.460599	-2.691358	-2.389762	-0.812046	-2.692975	-2.348112	-0.802426
H	-3.116976	-1.546852	-1.835364	-3.253427	-1.345461	-1.710301	-3.536262	-1.490252	-2.060192	-3.540347	-1.443424	-2.045048
H	-2.587171	-3.052555	-3.778043	-2.893403	-3.218437	-3.349912	-3.194574	-3.363233	-3.702841	-3.195985	-3.308538	-3.697720
H	-2.730363	-5.208416	-4.518667	-3.335126	-5.426081	-3.734553	-3.653187	-5.566844	-4.091187	-3.656286	-5.509991	-4.096090
H	-1.657861	-6.323962	-3.648968	-2.469951	-6.519040	-2.635488	-2.793804	-6.668298	-2.996027	-2.800779	-6.617342	-3.003867
H	-3.384662	-6.330819	-3.309083	-4.187909	-6.221124	-2.396084	-4.509094	-6.358520	-2.752650	-4.516022	-6.305713	-2.762475
H	-1.894370	-4.472057	-0.322344	-2.528149	-4.113514	0.324452	-2.828816	-4.268909	-0.030853	-2.837017	-4.229933	-0.029127
H	-2.896487	-5.859234	-0.772179	-3.714006	-5.398356	0.052972	-4.024193	-5.544759	-0.302903	-4.034344	-5.502369	-0.308274
H	-1.172874	-5.877530	-1.121457	-2.003071	-5.723667	-0.192051	-2.316054	-5.881362	-0.552043	-2.326488	-5.841457	-0.555935
H	1.364261	-1.335036	-0.530676	1.173164	-1.548170	-0.225709	0.952422	-1.657546	-0.585532	0.954898	-1.614119	-0.585150
H	0.364160	-2.265079	0.602077	0.017766	-2.124177	0.991696	-0.209218	-2.305424	0.589693	-0.199429	-2.271110	0.592410

	5a-25			5a-26			5a-27			5a-28		
**Element**	**X**	**Y**	**Z**	**X**	**Y**	**Z**	**X**	**Y**	**Z**	**X**	**Y**	**Z**
C	-3.354701	3.221965	4.183869	-3.744186	2.275649	4.497646	2.977968	5.335072	-0.068958	2.915165	5.382716	-0.029629
C	-1.947809	3.224134	3.632409	-2.331218	2.488083	4.005844	2.377722	4.737846	1.182542	2.341250	4.770052	1.226756
C	-1.035365	4.247066	4.264185	-1.531063	3.472700	4.823259	2.756474	5.454725	2.455600	2.757232	5.463064	2.501318
C	-1.597078	2.367377	2.661771	-1.881323	1.830008	2.927081	1.580965	3.661324	1.108782	1.534973	3.700476	1.155442
C	-0.251864	2.213955	1.987256	-0.512271	1.903689	2.287958	0.893799	2.920385	2.233962	0.870890	2.946814	2.286108
C	0.332603	0.824893	2.169790	0.199640	0.563059	2.287728	1.344546	1.474310	2.335677	1.332135	1.502968	2.369676
C	1.357838	0.551598	3.079358	1.226819	0.253284	3.180502	2.293071	1.044276	3.269315	2.286934	1.067706	3.291704
C	1.880441	-0.741347	3.248856	1.870241	-0.995268	3.181739	2.705588	-0.295440	3.350377	2.711794	-0.268710	3.358142
C	2.966899	-1.030750	4.210430	2.947894	-1.213678	4.171624	3.699705	-0.752149	4.346643	3.719544	-0.619860	4.383238
O	3.393825	0.069248	4.890150	3.501781	-2.456129	4.094463	4.159657	0.247726	5.148859	4.063002	-1.938118	4.366619
O	3.458054	-2.127378	4.404376	3.332234	-0.397285	4.987959	4.094346	-1.896846	4.469891	4.217098	0.155235	5.178519
C	1.375882	-1.813570	2.490766	1.489126	-1.988724	2.261075	2.172176	-1.256450	2.470860	2.182414	-1.226301	2.472025
C	0.358587	-1.567566	1.586162	0.470000	-1.705232	1.366994	1.231461	-0.854571	1.540528	1.232512	-0.818593	1.551162

C	-0.143006	-0.271326	1.443710	-0.152070	-0.455093	1.395213	0.839289	0.486176	1.487151	0.829862	0.518980	1.513474
O	-1.142501	-0.180031	0.520441	-1.136240	-0.322893	0.460328	-0.095657	0.729436	0.519689	-0.111976	0.764909	0.553218
C	-1.440079	-1.524232	0.010596	-1.290463	-1.598704	-0.248448	-0.567015	-0.567268	0.012257	-0.575480	-0.529484	0.034324
C	-1.551101	-1.432482	-1.527376	-1.375553	-1.292267	-1.760166	-0.802082	-0.410087	-1.504023	-0.817961	-0.358553	-1.479339
O	-2.583101	-0.478223	-1.813824	-2.488247	-0.405811	-1.942872	0.439140	-0.049484	-2.121022	0.418299	0.016622	-2.097602
C	-2.061270	-2.767297	-2.076170	-1.741953	-2.573048	-2.514183	-1.846612	0.685507	-1.773671	-1.871005	0.732533	-1.733534
C	-0.221608	-0.984871	-2.178566	-0.081289	-0.632849	-2.291192	-1.189368	-1.747154	-2.164995	-1.198895	-1.691424	-2.152391
C	-0.302106	-0.763182	-3.702987	-0.149073	-0.196159	-3.769108	-2.498613	-2.405668	-1.683382	-2.501540	-2.363853	-1.672130
C	0.935321	-0.091270	-4.235825	1.030264	0.656784	-4.154138	-2.730443	-3.732441	-2.359245	-2.727405	-3.685032	-2.360838
C	1.831347	-0.554588	-5.121165	1.983751	0.414384	-5.067083	-3.738094	-4.098701	-3.166210	-3.736603	-4.050052	-3.166435
C	3.020598	0.284839	-5.526213	3.096347	1.409066	-5.303195	-3.784825	-5.488638	-3.756713	-3.776365	-5.433944	-3.771478
C	1.765251	-1.903643	-5.795505	2.058720	-0.818479	-5.935141	-4.895868	-3.215671	-3.564263	-4.902774	-3.171307	-3.549125
C	-0.370333	-2.471299	0.618563	-0.147262	-2.521412	0.254535	0.510241	-1.592790	0.437110	0.511512	-1.551172	0.443654
H	-3.354967	3.022067	5.264758	-3.750513	1.907186	5.533228	2.674994	6.384486	-0.192021	2.617642	6.436251	-0.128365
H	-3.834510	4.201787	4.049631	-4.308702	3.218836	4.500695	4.076044	5.332858	-0.020153	4.014032	5.371428	-0.007837
H	-3.982712	2.467783	3.699498	-4.289190	1.556343	3.878404	2.678013	4.788252	-0.968398	2.588824	4.852775	-0.930039
H	-0.967605	4.085145	5.348934	-1.472166	3.145515	5.870541	2.441458	6.506754	2.415723	2.453251	6.518956	2.484658
H	-0.019687	4.235288	3.862055	-0.509948	3.614145	4.461898	2.320586	5.010901	3.353568	2.338027	5.009939	3.402475
H	-1.437047	5.260787	4.127566	-2.021602	4.456026	4.833486	3.847906	5.463309	2.582580	3.851260	5.457377	2.602908
H	-2.356656	1.676253	2.296396	-2.565407	1.141563	2.430839	1.392389	3.240400	0.120538	1.319564	3.296202	0.165710
H	-0.372392	2.404865	0.910968	-0.625687	2.240697	1.247311	1.063484	3.413629	3.195023	1.053956	3.433148	3.248048
H	0.465898	2.951364	2.356923	0.122183	2.640301	2.788078	-0.190646	2.935611	2.056123	-0.216421	2.956947	2.126198
H	1.763849	1.363345	3.674110	1.552835	0.992254	3.906301	2.716808	1.766146	3.959876	2.718841	1.772545	3.995860
H	4.098704	-0.252759	5.481397	4.187738	-2.473890	4.786735	4.797976	-0.179385	5.749049	4.724291	-2.039190	5.075539
H	1.797901	-2.803173	2.633824	1.991293	-2.950075	2.260411	2.510169	-2.284869	2.547281	2.517625	-2.256552	2.521502
H	-2.438614	-1.765907	0.390475	-2.268903	-1.985662	0.056059	-1.515194	-0.766237	0.526000	-1.519910	-0.741422	0.549821
H	-2.339857	0.337875	-1.344550	-2.335840	0.351184	-1.351959	0.740595	0.758176	-1.671064	0.715716	0.822354	-1.641485
H	-2.308914	-2.665115	-3.135877	-1.974393	-2.339331	-3.556337	-1.988970	0.797967	-2.853146	-2.018630	0.854982	-2.811214
H	-1.310632	-3.557668	-1.969935	-0.921071	-3.297828	-2.495994	-2.814285	0.452296	-1.315891	-2.835137	0.488082	-1.274155
H	-2.972403	-3.073228	-1.551074	-2.630903	-3.035628	-2.072274	-1.511051	1.644257	-1.365879	-1.540315	1.689339	-1.317342
H	0.088647	-0.047277	-1.698658	0.126701	0.247823	-1.669297	-1.254656	-1.561409	-3.243610	-1.270013	-1.495128	-3.228761
H	0.569957	-1.714180	-1.965135	0.771867	-1.309907	-2.157769	-0.356346	-2.448220	-2.031864	-0.360530	-2.388114	-2.029980
H	-0.480892	-1.716046	-4.210652	-0.225404	-1.075572	-4.415998	-2.448163	-2.573001	-0.596412	-2.445286	-2.542065	-0.587161
H	-1.178880	-0.133882	-3.906363	-1.077012	0.374116	-3.909928	-3.342188	-1.728750	-1.848587	-3.350348	-1.690993	-1.826642
H	1.114197	0.904812	-3.824656	1.105934	1.591937	-3.594713	-1.955501	-4.475812	-2.161221	-1.946083	-4.424856	-2.174811
H	3.029997	1.253656	-5.016855	3.004295	2.287054	-4.656075	-2.930576	-6.096162	-3.441932	-2.915993	-6.038488	-3.467782
H	3.965113	-0.229035	-5.297285	4.080431	0.954026	-5.121222	-4.703696	-6.014130	-3.460331	-4.689759	-5.969307	-3.475844
H	3.024411	0.470731	-6.609735	3.104000	1.755453	-6.346529	-3.789802	-5.451667	-4.855246	-3.787561	-5.385330	-4.869504
H	0.910879	-2.506418	-5.478996	1.259298	-1.536964	-5.739709	-4.840203	-2.208290	-3.145393	-4.852532	-2.168244	-3.119371
H	1.707979	-1.789379	-6.887131	2.011900	-0.547266	-6.999205	-4.950454	-3.121419	-4.657676	-4.963356	-3.065376	-4.641146
H	2.677831	-2.481826	-5.593574	3.017931	-1.335176	-5.790748	-5.849126	-3.659461	-3.244751	-5.851154	-3.625689	-3.230010
H	0.306106	-2.874149	-0.144327	0.580649	-2.744018	-0.534553	1.188167	-1.801081	-0.399566	1.186501	-1.745942	-0.398710
H	-0.840436	-3.327735	1.113229	-0.543290	-3.480845	0.603903	0.064701	-2.538573	0.760617	0.074024	-2.503451	0.759364

5a-29

Element	X	Y	Z
C	2.246586	4.960265	-0.927681
C	1.414655	4.381120	0.193301
C	-0.069087	4.622066	0.057767
C	2.000978	3.733110	1.211847
C	1.354275	3.108086	2.428591
C	1.734313	1.649706	2.593227
C	2.689887	1.215713	3.514458
C	3.059131	-0.134021	3.634626
C	4.076008	-0.481459	4.651406
O	4.360283	-1.813351	4.691024
O	4.627705	0.307268	5.396047
C	2.470502	-1.105379	2.803311
C	1.514908	-0.699374	1.887124
C	1.162369	0.649619	1.799893
O	0.200707	0.891289	0.862539
C	-0.297964	-0.401608	0.373775
C	-0.511683	-0.254773	-1.148082
O	0.739307	0.105763	-1.743309
C	-1.552886	0.838991	-1.438134
C	-0.890573	-1.593281	-1.810370
C	-2.204714	-2.250630	-1.340337
C	-2.426508	-3.582371	-2.009652
C	-3.424537	-3.956580	-2.824888
C	-3.461361	-5.350470	-3.406723
C	-4.580244	-3.079202	-3.240987
C	0.750225	-1.448333	0.821164
H	1.950677	4.540839	-1.899701
H	2.103632	6.047458	-1.004211

H	3.314781	4.768829	-0.785981
H	-0.429746	4.272074	-0.919300
H	-0.661914	4.126509	0.829516
H	-0.291610	5.697648	0.100269
H	3.087218	3.634982	1.189669
H	1.680698	3.650343	3.326959
H	0.264711	3.199935	2.384037
H	3.169254	1.933310	4.173917
H	5.034579	-1.910303	5.388166
H	2.766578	-2.145251	2.888925
H	-1.255818	-0.568770	0.880487
H	1.033611	0.911998	-1.284404
H	-1.675920	0.949039	-2.520308
H	-2.528648	0.605601	-0.997833
H	-1.225060	1.798493	-1.026291
H	-0.944486	-1.410686	-2.890166
H	-0.059310	-2.294259	-1.667023
H	-2.167039	-2.409987	-0.251574
H	-3.047133	-1.576141	-1.520367
H	-1.651887	-4.322524	-1.798589
H	-2.608233	-5.953213	-3.079886
H	-4.381350	-5.876993	-3.115671
H	-3.455966	-5.320719	-4.505470
H	-4.532410	-2.069523	-2.826701
H	-4.622444	-2.990748	-4.335433
H	-5.535854	-3.524156	-2.930222
H	1.410000	-1.709229	-0.015179
H	0.276802	-2.366720	1.182427

Table S4.6. Calculated conformational analysis of the **5b** at B3LYP/6-31g(d) level

conformer	3D conformer	G (Hartree)	ΔG (kcal/mol)	Population
5b-1	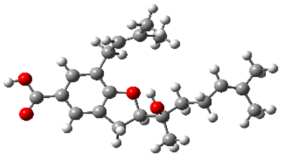	-1156.886338	0	9.876%
5b-2	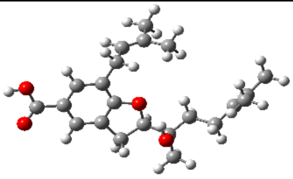	-1156.886069	0.16880019	7.427%
5b-3	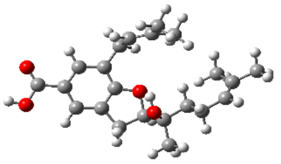	-1156.885984	0.22213854	6.788%
5b-4	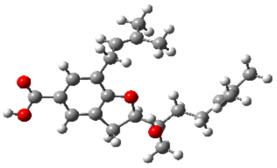	-1156.885977	0.22653111	6.738%
5b-5	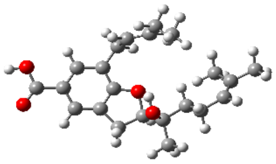	-1156.885925	0.25916163	6.376%
5b-6	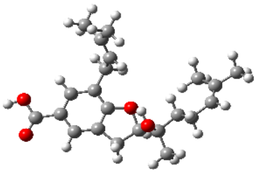	-1156.885615	0.45368973	4.592%
5b-7	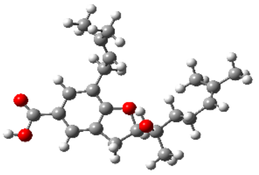	-1156.885578	0.4769076	4.415%
5b-8	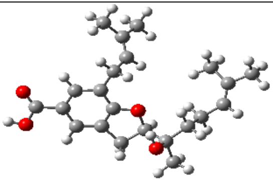	-1156.885572	0.48067266	4.387%

5b-9	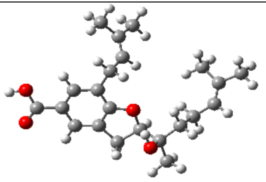	-1156.885522	0.51204816	4.161%
5b-10	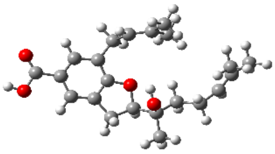	-1156.885463	0.54907125	3.909%
5b-11	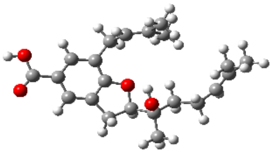	-1156.885453	0.55534635	3.868%
5b-12	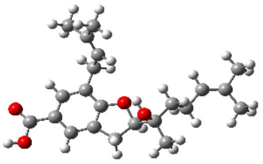	-1156.88541	0.58232928	3.695%
5b-13	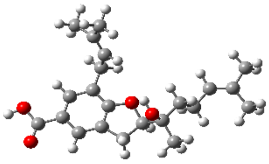	-1156.885364	0.61119474	3.520%
5b-14	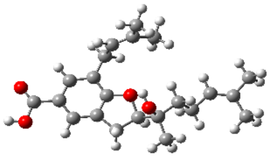	-1156.885252	0.68147586	3.126%
5b-15	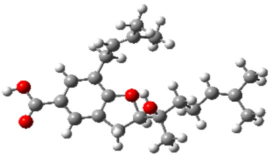	-1156.885246	0.68524092	3.106%
5b-16	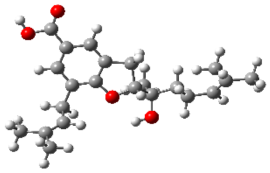	-1156.885201	0.71347887	2.962%
5b-17	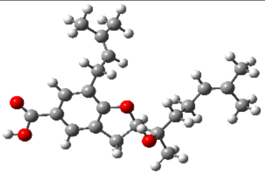	-1156.885154	0.74297184	2.818%

5b-18	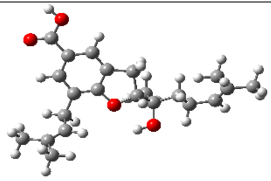	-1156.88514	0.75175698	2.776%
5b-19	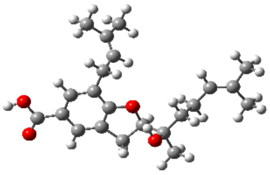	-1156.885136	0.75426702	2.765%
5b-20	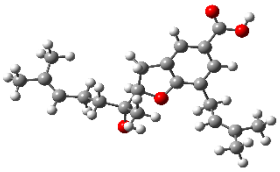	-1156.884958	0.8659638	2.290%
5b-21	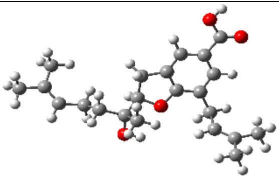	-1156.88486	0.92745978	2.064%
5b-22	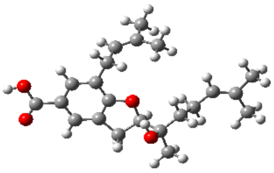	-1156.884804	0.96260034	1.945%
5b-23	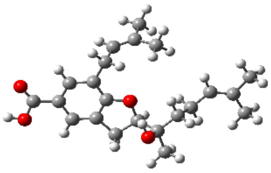	-1156.884768	0.9851907	1.872%

Table S4.7. Cartesian coordinates of low-energy conformers of **5b**

	**5b-1**			**5b-2**			**5b-3**			**5b-4**		
**Element**	**X**	**Y**	**Z**	**X**	**Y**	**Z**	**X**	**Y**	**Z**	**X**	**Y**	**Z**
C	-0.057538	-4.148207	-1.062366	0.646840	-1.091713	4.401907	-0.361209	-3.757703	-2.076006	0.606207	-1.109557	4.444934
C	-0.190907	-3.314360	0.190973	0.209081	-1.538097	3.026356	-0.361990	-3.101185	-0.714675	0.184470	-1.554631	3.063949
C	1.122678	-2.897056	0.805624	1.342051	-1.776620	2.059151	1.003599	-2.967757	-0.085908	1.328313	-1.786998	2.108097
C	-1.402521	-3.015935	0.684905	-1.092970	-1.696585	2.745383	-1.513721	-2.707301	-0.149242	-1.113970	-1.717709	2.769257
C	-1.744270	-2.222615	1.927213	-1.728710	-2.132840	1.444122	-1.724907	-2.057694	1.200893	-1.734551	-2.154792	1.460957
C	-2.707201	-1.088354	1.636329	-2.712213	-1.107144	0.912532	-2.539378	-0.782411	1.107900	-2.711061	-1.128493	0.918002
C	-4.073755	-1.166437	1.922757	-4.094318	-1.231700	1.086838	-3.876175	-0.707955	1.504372	-4.093322	-1.245797	1.081328
C	-4.961756	-0.121699	1.617767	-4.996868	-0.260202	0.623859	-4.629822	0.471722	1.389167	-4.991784	-0.275382	0.609037
C	-6.405782	-0.201260	1.929664	-6.458276	-0.390646	0.814288	-6.035551	0.438084	1.848570	-6.435169	-0.501436	0.843105
O	-6.766257	-1.372501	2.523913	-6.818487	-1.531365	1.465278	-6.676853	1.630735	1.697874	-7.219857	0.498782	0.353349
O	-7.223221	0.669281	1.694684	-7.289060	0.416998	0.441751	-6.601686	-0.531412	2.318283	-6.915733	-1.465471	1.409209
C	-4.490265	1.048144	0.993960	-4.523803	0.880737	-0.050129	-4.047093	1.633472	0.849169	-4.513096	0.862971	-0.066034
C	-3.141063	1.150462	0.707462	-3.160703	1.025547	-0.234058	-2.720511	1.585580	0.454756	-3.146292	1.001249	-0.239401
C	-2.280870	0.098254	1.032417	-2.286891	0.046155	0.246551	-1.996082	0.399001	0.591204	-2.278418	0.021940	0.250430
O	-0.980611	0.350549	0.701624	-0.976351	0.327391	-0.017755	-0.703652	0.509469	0.170137	-0.965341	0.298728	-0.006523
C	-0.877729	1.754256	0.282355	-0.899654	1.707864	-0.511641	-0.438239	1.917045	-0.146457	-0.881464	1.676157	-0.506242
C	0.098254	1.805780	-0.913688	0.185266	1.754598	-1.608906	0.384342	1.946481	-1.450805	0.208947	1.714478	-1.598453
O	-0.419083	0.970889	-1.954001	-0.193922	0.862973	-2.663124	-0.407380	1.377944	-2.500272	-0.168983	0.820538	-2.651191
C	0.154739	3.224099	-1.486884	0.238698	3.152859	-2.229739	0.662037	3.390323	-1.874875	0.270901	3.110171	-2.224300
C	1.495000	1.311010	-0.461707	1.548065	1.333583	-1.005270	1.688089	1.121052	-1.326953	1.567230	1.290357	-0.986976
C	2.545121	1.227110	-1.587547	2.704695	1.237232	-2.020071	2.710863	1.565240	-0.260108	2.728268	1.186446	-1.996018
C	3.788054	0.503536	-1.142028	3.969039	0.727100	-1.380215	3.977568	0.752749	-0.337662	3.987591	0.673324	-1.348688
C	5.045084	0.964643	-1.049571	4.664310	-0.385166	-1.664913	4.501715	-0.076732	0.578135	4.679852	-0.442436	-1.626938
C	6.162806	0.067558	-0.571019	5.919080	-0.728609	-0.896356	5.792866	-0.811505	0.302114	5.929436	-0.788679	-0.851234
C	5.481283	2.366811	-1.399912	4.301953	-1.379918	-2.740939	3.902187	-0.361787	1.933932	4.318798	-1.438861	-2.701849
C	-2.330038	2.211004	0.001735	-2.338278	2.067666	-0.954334	-1.825033	2.605969	-0.207741	-2.316604	2.038648	-0.958419
H	0.493381	-3.605636	-1.843628	1.310479	-1.834322	4.867071	0.260156	-3.195545	-2.787478	1.267606	-1.850811	4.915428
H	0.511336	-5.067994	-0.865869	1.219415	-0.154836	4.349082	0.065537	-4.769464	-2.026316	1.175927	-0.170483	4.400198
H	-1.032210	-4.433867	-1.470062	-0.204482	-0.931661	5.070893	-1.369883	-3.836360	-2.493513	-0.252670	-0.954227	5.105297
H	1.751688	-2.386345	0.063382	2.020007	-2.552504	2.441588	1.687682	-2.421882	-0.749916	2.004258	-2.562388	2.495085
H	1.009476	-2.229257	1.662218	1.008475	-2.083594	1.065739	0.990367	-2.449131	0.875104	1.006013	-2.091408	1.110189
H	1.691231	-3.777367	1.137202	1.946961	-0.867319	1.940471	1.451754	-3.958838	0.072739	1.932066	-0.875708	1.998913
H	-2.273999	-3.388272	0.144511	-1.811193	-1.484478	3.538620	-2.434987	-2.873897	-0.709302	-1.841322	-1.510174	3.555285
H	-2.214757	-2.891074	2.662091	-0.966915	-2.338958	0.686350	-2.261237	-2.756867	1.857742	-0.964296	-2.361766	0.712029
H	-0.840605	-1.824168	2.398427	-2.272554	-3.073958	1.606777	-0.766538	-1.844608	1.684857	-2.281319	-3.094879	1.618289
H	-4.460080	-2.059829	2.402927	-4.482535	-2.106054	1.599519	-4.364130	-1.582418	1.924848	-4.504592	-2.111340	1.592607
H	-7.726545	-1.298422	2.673796	-7.790762	-1.495927	1.524670	-7.580700	1.478708	2.029552	-8.133685	0.234555	0.565939
H	-5.195105	1.838360	0.756140	-5.238655	1.613685	-0.410084	-4.634311	2.539933	0.749883	-5.209124	1.605676	-0.440516
H	-0.454547	2.302293	1.133933	-0.591250	2.324614	0.342458	0.154200	2.315206	0.685743	-0.575478	2.296131	0.346406
H	-0.559200	0.095653	-1.551320	-0.333651	-0.003748	-2.243809	-0.616666	0.469801	-2.220371	-0.311851	-0.044687	-2.229823
H	0.797469	3.245734	-2.370531	0.964624	3.174405	-3.046315	1.267846	3.394977	-2.786498	1.000483	3.125619	-3.037738
H	0.555449	3.928691	-0.749519	0.532547	3.901104	-1.485179	1.200287	3.943076	-1.098231	0.564610	3.859995	-1.481271
H	-0.838060	3.564410	-1.794173	-0.732864	3.434237	-2.645661	-0.272005	3.916848	-2.091412	-0.697643	3.394053	-2.645602
H	1.866842	1.963800	0.339910	1.813660	2.038501	-0.204344	1.413339	0.078271	-1.128686	1.831653	1.996869	-0.187068
H	1.372140	0.317696	-0.012001	1.421926	0.357785	-0.521623	2.161153	1.137628	-2.317021	1.435191	0.316656	-0.500717
H	2.091099	0.690543	-2.431986	2.386115	0.606673	-2.854592	2.964154	2.624190	-0.415802	2.411205	0.554531	-2.830093
H	2.786562	2.229760	-1.953067	2.902768	2.232812	-2.442605	2.264710	1.496743	0.737179	2.932167	2.179966	-2.420658
H	3.619244	-0.535435	-0.849049	4.350204	1.357458	-0.573509	4.518530	0.859929	-1.280183	4.367266	1.304434	-0.541878
H	5.805040	-0.938181	-0.328701	6.145414	0.017224	-0.127730	6.193931	-0.576332	-0.688784	6.155120	-0.041437	-0.083790
H	6.949872	-0.026460	-1.332771	5.827420	-1.707412	-0.404316	6.559695	-0.558386	1.047968	5.831241	-1.765549	-0.356646
H	6.647509	0.481196	0.324853	6.787487	-0.799948	-1.566647	5.650247	-1.899762	0.362273	6.800777	-0.865715	-1.517066
H	4.659750	3.008657	-1.726244	3.390017	-1.119244	-3.282597	2.959722	0.161044	2.111993	4.175453	-2.439369	-2.270112
H	5.962151	2.848901	-0.537218	5.114331	-1.468242	-3.475871	3.715924	-1.438079	2.054275	3.410688	-1.176093	-3.248906
H	6.232288	2.350546	-2.202213	4.164923	-2.382301	-2.311475	4.600220	-0.078201	2.734085	5.134518	-1.532716	-3.432378
H	-2.532254	2.196748	-1.076297	-2.440066	1.960536	-2.041406	-2.125266	2.766241	-1.249998	-2.411966	1.928463	-2.045818
H	-2.515348	3.224199	0.370700	-2.600422	3.095375	-0.686042	-1.817718	3.575764	0.299492	-2.576667	3.068255	-0.694974
	**5b-5**			**5b-6**			**5b-7**			**5b-8**		
**Element**	**X**	**Y**	**Z**	**X**	**Y**	**Z**	**X**	**Y**	**Z**	**X**	**Y**	**Z**
C	-0.153214	-3.724488	-2.109959	-0.870374	-4.697024	-1.187415	-0.868649	-4.709504	-1.190653	0.363498	-2.342584	3.780471
C	-0.115447	-3.082307	-0.742346	-1.244553	-3.919371	0.052957	-1.248551	-3.939852	0.052967	-0.517835	-2.752504	2.623391
C	1.274078	-2.819656	-0.214936	-1.945095	-4.737340	1.110413	-1.972854	-4.759266	1.093054	-1.336552	-3.994819	2.876971
C	-1.254184	-2.808200	-0.086984	-0.952734	-2.613916	0.151893	-0.942019	-2.639133	0.168455	-0.530200	-2.034177	1.490697
C	-1.425302	-2.192280	1.284454	-1.243424	-1.669055	1.296446	-1.236084	-1.702796	1.319209	-1.337849	-2.277124	0.235184
C	-2.366515	-1.003737	1.271970	-2.155646	-0.526455	0.887783	-2.146214	-0.556626	0.916229	-2.296886	-1.139234	-0.066119
C	-3.671066	-1.068140	1.770761	-3.524572	-0.515142	1.174129	-3.512667	-0.540003	1.205265	-3.667037	-1.219180	0.192275
C	-4.541759	0.033597	1.729588	-4.358613	0.545891	0.786449	-4.347565	0.522271	0.823764	-4.544412	-0.155619	-0.073428
C	-5.918890	-0.032681	2.265438	-5.804406	0.563460	1.100488	-5.777881	0.439901	1.193825	-5.976880	-0.350606	0.241971
O	-6.239146	-1.251162	2.783216	-6.222544	-0.537871	1.784021	-6.500632	1.518343	0.780711	-6.741089	0.740690	-0.042888
O	-6.717952	0.885452	2.263021	-6.577564	1.452897	0.796496	-6.297754	-0.476357	1.802985	-6.465571	-1.362981	0.707952

C	-4.116974	1.250033	1.164103	-3.829928	1.641500	0.078124	-3.820799	1.619817	0.116411	-4.057529	1.044217	-0.625837
C	-2.828419	1.339233	0.669272	-2.478506	1.652937	-0.214687	-2.468264	1.626645	-0.179144	-2.701524	1.148511	-0.885839
C	-1.982683	0.228589	0.733361	-1.674345	0.582565	0.187562	-1.665066	0.554102	0.217265	-1.855202	0.073691	-0.602552
O	-0.743258	0.469338	0.218127	-0.364339	0.735787	-0.171937	-0.355877	0.705784	-0.146228	-0.548146	0.327380	-0.913238
C	-0.642008	1.899195	-0.095959	-0.187487	2.107764	-0.663489	-0.177846	2.077945	-0.635272	-0.428003	1.752291	-1.243973
C	0.073144	2.019850	-1.457111	0.797070	2.059200	-1.847831	0.801470	2.030035	-1.824045	0.564148	1.878914	-2.416518
O	-0.734944	1.382485	-2.453174	0.205913	1.303103	-2.911477	0.202961	1.279791	-2.887768	0.015618	1.209258	-3.558097
C	0.173923	3.487177	-1.879471	1.010466	3.465286	-2.413640	1.017393	3.437309	-2.385905	0.709228	3.345214	-2.830660
C	1.457777	1.327677	-1.442354	2.134587	1.384845	-1.456492	2.138198	1.349637	-1.440785	1.931743	1.233646	-2.084705
C	2.509725	1.865243	-0.449411	2.938410	2.025935	-0.305324	2.948740	1.983201	-0.290132	2.714698	1.813045	-0.887415
C	3.840394	1.181959	-0.631344	4.282039	1.365833	-0.132343	4.290027	1.316576	-0.123972	4.079522	1.186513	-0.763242
C	4.512028	0.405409	0.233144	4.725980	0.625571	0.895206	4.732475	0.567685	0.897960	4.565656	0.422285	0.226967
C	5.844243	-0.197307	-0.147756	6.117159	0.036526	0.875822	6.121084	-0.027137	0.872359	5.972277	-0.124976	0.157526
C	4.048905	0.058326	1.627399	3.932255	0.305515	2.138736	3.939341	0.242664	2.140578	3.808756	0.033423	1.473849
C	-2.090292	2.447796	-0.043332	-1.612002	2.623140	-0.983244	-1.602512	2.597352	-0.948207	-1.871417	2.240818	-1.518976
H	0.354382	-3.097737	-2.856797	-0.205458	-5.537419	-0.942780	-0.217609	-5.560802	-0.946394	-0.234067	-2.145243	4.681745
H	0.373347	-4.689356	-2.107954	-1.759831	-5.133578	-1.663305	-1.757593	-5.129814	-1.681832	1.068226	-3.143611	4.045198
H	-1.177952	-3.899139	-2.452524	-0.364190	-4.069139	-1.927360	-0.345258	-4.080699	-1.917798	0.943329	-1.442144	3.554229
H	1.848929	-2.202956	-0.919222	-1.313826	-5.577799	1.430920	-1.357749	-5.612738	1.410600	-1.984530	-3.856543	3.753525
H	1.282856	-2.313121	0.752541	-2.212652	-4.163853	2.000844	-2.242517	-4.192135	1.986809	-1.972967	-4.278642	2.035772
H	1.828105	-3.762766	-0.106095	-2.867032	-5.179145	0.707559	-2.896747	-5.182141	0.674876	-0.683901	-4.848741	3.106256
H	-2.194354	-3.059888	-0.579897	-0.445025	-2.144970	-0.691606	-0.418798	-2.167811	-0.664207	0.108141	-1.151945	1.441639
H	-1.837706	-2.946411	1.969720	-1.692796	-2.196872	2.142155	-1.689543	-2.236347	2.158905	-0.645792	-2.388233	-0.611840
H	-0.458551	-1.888384	1.698123	-0.293940	-1.246231	1.653846	-0.287514	-1.283562	1.683255	-1.905604	-3.209203	0.300619
H	-4.020809	-1.996878	2.210147	-3.954843	-1.347464	1.721522	-3.959573	-1.364981	1.751960	-4.084931	-2.127407	0.616141
H	-7.157854	-1.162206	3.096486	-7.177738	-0.403906	1.924571	-7.412232	1.344860	1.078983	-7.649643	0.488227	0.203839
H	-4.809299	2.085190	1.131842	-4.494192	2.447532	-0.216501	-4.469715	2.435922	-0.182135	-4.739456	1.859810	-0.840403
H	-0.030355	2.347262	0.695971	0.247898	2.678732	0.165506	0.262262	2.646321	0.193032	-0.022473	2.242909	-0.350972
H	-0.833297	0.456579	-2.170578	0.031960	0.416485	-2.552169	0.029341	0.391841	-2.531546	-0.110951	0.281809	-3.294565
H	0.706271	3.558805	-2.833189	1.723883	3.422101	-3.242538	1.726691	3.394448	-3.218337	1.436937	3.426548	-3.644192
H	0.711457	4.085358	-1.136726	1.400740	4.151469	-1.655300	1.413877	4.119258	-1.626964	1.048836	3.969282	-1.997939
H	-0.821259	3.919285	-2.018676	0.071720	3.872524	-2.800561	0.078353	3.849475	-2.766942	-0.244412	3.739404	-3.193808
H	1.303310	0.261530	-1.238164	1.928190	0.341010	-1.190585	1.929221	0.305511	-1.178144	1.772493	0.163158	-1.906799
H	1.850321	1.397239	-2.464785	2.749296	1.359207	-2.365118	2.749259	1.325461	-2.351909	2.541225	1.311785	-2.993867
H	2.645572	2.944727	-0.610634	3.098289	3.092590	-0.520034	3.112578	3.049931	-0.501518	2.838276	2.897484	-1.022721
H	2.149061	1.748366	0.577530	2.359679	1.979594	0.622599	2.372969	1.935958	0.639579	2.139560	1.679004	0.034225
H	4.294655	1.345130	-1.610885	4.961880	1.508484	-0.974795	4.968935	1.461760	-0.966736	4.738707	1.381918	-1.611569
H	6.143152	0.079260	-1.163729	6.658080	0.294274	-0.040162	6.661694	0.234524	-0.042705	6.485621	0.180882	-0.759518
H	6.637334	0.128230	0.540311	6.709305	0.390998	1.731483	6.716030	0.318958	1.729500	6.572610	0.215560	1.013111
H	5.814441	-1.294522	-0.088346	6.084355	-1.059350	0.954980	6.083667	-1.123360	0.944133	5.971939	-1.223466	0.197296
H	3.978804	-1.031478	1.750059	2.919311	0.714409	2.126028	2.927925	0.655432	2.132075	2.789573	0.425942	1.504228
H	4.774985	0.406783	2.375267	3.851429	-0.781980	2.273828	3.854475	-0.845351	2.268653	3.748241	-1.060063	1.562850
H	3.076196	0.485282	1.882040	4.440248	0.690871	3.033956	4.450167	0.620143	3.037536	4.332817	0.388556	2.372259
H	-2.483482	2.584685	-1.057565	-1.807974	2.555101	-2.060114	-1.802063	2.533080	-2.024706	-2.055276	2.295611	-2.598753
H	-2.140420	3.409749	0.476024	-1.744520	3.664751	-0.675509	-1.731011	3.638525	-0.636922	-2.051595	3.231225	-1.089839

	5b-9			5b-10			5b-11			5b-12		
**Element**	**X**	**Y**	**Z**	**X**	**Y**	**Z**	**X**	**Y**	**Z**	**X**	**Y**	**Z**
C	0.837789	-2.753341	3.374913	0.147250	-3.600428	-0.852173	-0.053932	-3.396748	-1.387399	-0.843190	-5.004784	-1.050388
C	-0.204279	-3.017056	2.312772	-0.107984	-2.821312	0.417605	-0.110007	-2.815982	0.006888	-1.289944	-4.275502	0.195344
C	-0.993019	-4.287175	2.519730	1.133280	-2.509866	1.217883	1.238433	-2.628015	0.658410	-2.003803	-5.145107	1.201337
C	-0.369153	-2.159913	1.294324	-1.358580	-2.480699	0.766912	-1.290883	-2.530297	0.577808	-1.044960	-2.964844	0.341384
C	-1.349570	-2.244684	0.145810	-1.820957	-1.732940	1.998537	-1.557938	-1.973231	1.959194	-1.410685	-2.063480	1.499445
C	-2.317158	-1.074709	0.122098	-2.683995	-0.533941	1.657578	-2.435730	-0.737368	1.925717	-2.350636	-0.944489	1.088710
C	-3.639269	-1.181502	0.564932	-4.067534	-0.516233	1.844842	-3.779196	-0.750894	2.312403	-3.725880	-0.987055	1.329497
C	-4.519910	-0.087580	0.554762	-4.858575	0.591630	1.497960	-4.586009	0.397656	2.248840	-4.588185	0.050661	0.941508
C	-5.917804	-0.200380	1.026327	-6.313874	0.503411	1.748619	-6.005048	0.385016	2.666529	-6.026059	-0.095099	1.259027
O	-6.246262	-1.452774	1.448928	-6.989485	1.628510	1.382431	-6.428992	-0.833691	3.102656	-6.775956	0.963863	0.843814
O	-6.725677	0.709591	1.050328	-6.890472	-0.454349	2.229437	-6.753442	1.344469	2.638529	-6.530241	-1.044315	1.829671
C	-4.088092	1.166045	0.082657	-4.266004	1.734425	0.928894	-4.054533	1.610817	1.773591	-4.080867	1.183423	0.276704
C	-2.783042	1.296590	-0.356122	-2.894135	1.742398	0.739433	-2.725853	1.649616	1.390733	-2.720287	1.248895	0.029285
C	-1.929069	0.190682	-0.325481	-2.135533	0.628801	1.105862	-1.946660	0.493443	1.477133	-1.889734	0.199395	0.430898
O	-0.673822	0.472941	-0.787773	-0.802029	0.788473	0.869751	-0.657296	0.686188	1.077654	-0.575703	0.409858	0.117195
C	-0.570287	1.927065	-0.962276	-0.566688	2.171268	0.440549	-0.455901	2.117919	0.827190	-0.435080	1.799428	-0.333798
C	0.249355	2.187622	-2.241141	0.443862	2.119160	-0.727454	0.378335	2.239713	-0.467894	0.587603	1.816665	-1.486714
O	-0.468067	1.659794	-3.363273	-0.130982	1.343359	-1.784185	-0.355543	1.628378	-1.534026	0.063138	1.049509	-2.577098
C	0.365100	3.692020	-2.497961	0.669534	3.522315	-1.294932	0.550447	3.711513	-0.849899	0.752756	3.239488	-2.025903
C	1.636342	1.502075	-2.184686	1.762408	1.471128	-0.237957	1.739165	1.527045	-0.271684	1.943257	1.199389	-1.064826
C	2.592820	1.934294	-1.053081	2.820787	1.254351	-1.337815	2.626960	1.475763	-1.531040	2.722261	1.908794	0.063019
C	3.947528	1.290410	-1.197354	3.990429	0.448612	-0.836561	3.838628	0.605124	-1.325297	3.990687	1.171494	0.406580
C	4.552336	0.414511	-0.380110	4.477880	-0.708725	-1.312268	4.230171	-0.468646	-2.030324	5.261752	1.585325	0.288273
C	5.922766	-0.128455	-0.711784	5.672450	-1.367835	-0.662421	5.490575	-1.216250	-1.661247	6.405831	0.680500	0.682391
C	3.971432	-0.111487	0.910130	3.930953	-1.451711	-2.507462	3.505056	-1.026286	-3.231530	5.689130	2.939404	-0.224176
C	-2.026889	2.452185	-0.968859	-1.964637	2.751010	0.106248	-1.874439	2.740607	0.785242	-1.867270	2.268782	-0.688627

H	0.377433	-2.677549	4.370246	0.829133	-3.056494	-1.521239	0.538150	-2.759145	-2.059186	-0.161420	-5.830754	-0.803034
H	1.563778	-3.576839	3.431173	0.631746	-4.562671	-0.633695	0.435569	-4.381005	-1.385214	-1.699162	-5.454951	-1.572671
H	1.389782	-1.827339	3.185408	-0.777385	-3.804848	-1.401072	-1.051092	-3.516349	-1.822617	-0.329447	-4.340282	-1.752245
H	-1.506700	-4.267719	3.490953	1.874633	-1.984369	0.600323	1.895737	-2.015514	0.026053	-1.358140	-5.973936	1.523484
H	-1.746431	-4.464653	1.748847	0.938394	-1.894590	2.098908	1.183564	-2.152060	1.639835	-2.324992	-4.606231	2.095542
H	-0.324551	-5.159177	2.541052	1.614861	-3.438142	1.556688	1.743289	-3.596540	0.782830	-2.894465	-5.603809	0.750156
H	0.266897	-1.275130	1.267226	-2.168914	-2.778647	0.099962	-2.193599	-2.724445	-0.003330	-0.521272	-2.455996	-0.468654
H	-0.785340	-2.254139	-0.797739	-2.412738	-2.410174	2.630142	-2.067163	-2.737269	2.563473	-1.869830	-2.630528	2.313698
H	-1.921494	-3.176147	0.177961	-0.966522	-1.408572	2.600894	-0.619900	-1.741299	2.473383	-0.491116	-1.616115	1.902314
H	-3.996004	-2.138586	0.931336	-4.565055	-1.378810	2.278419	-4.211766	-1.676148	2.679398	-4.158562	-1.840804	1.842398
H	-7.177981	-1.391862	1.728724	-7.923474	1.445862	1.593085	-7.365119	-0.707677	3.343230	-7.689756	0.748611	1.105952
H	-4.788746	1.994746	0.073587	-4.880902	2.583036	0.649441	-4.699062	2.482365	1.720486	-4.750641	1.980481	-0.027195
H	-0.032115	2.305001	-0.084715	-0.120437	2.690823	1.297946	0.117977	2.504150	1.679051	-0.049789	2.364522	0.523458
H	-0.577496	0.708663	-3.193028	-0.348284	0.477967	-1.394445	-0.535013	0.715348	-1.247416	-0.085687	0.152504	-2.232397
H	0.972726	3.866105	-3.391555	1.341047	3.475916	-2.155989	1.092699	3.793469	-1.795292	1.505555	3.245958	-2.820467
H	0.831225	4.211934	-1.654954	1.116770	4.181306	-0.542312	1.112737	4.252155	-0.080320	1.070309	3.933989	-1.241476
H	-0.621773	4.130076	-2.673478	-0.270903	3.965669	-1.634493	-0.419206	4.199591	-0.983241	-0.187287	3.602164	-2.451877
H	1.481495	0.418798	-2.110332	2.184152	2.084761	0.570979	2.283486	2.014501	0.549830	1.763227	0.158603	-0.766322
H	2.112543	1.683398	-3.156536	1.516382	0.502197	0.211087	1.539032	0.502215	0.061064	2.566907	1.162783	-1.965880
H	2.719517	3.026434	-1.079956	2.338437	0.779084	-2.196285	2.018463	1.132620	-2.372168	2.934832	2.944044	-0.221701
H	2.150780	1.697294	-0.080138	3.187368	2.230291	-1.688423	2.960578	2.492958	-1.783543	2.093907	1.956775	0.964673
H	4.484076	1.578914	-2.103583	4.493359	0.877961	0.032956	4.467876	0.901030	-0.482781	3.828768	0.161618	0.788872
H	6.307446	0.278209	-1.652359	6.035567	-0.798556	0.199118	5.983067	-0.780999	-0.785921	6.053356	-0.288612	1.049211
H	6.645546	0.108264	0.081916	5.428387	-2.383648	-0.320630	5.276319	-2.271729	-1.441353	7.077818	0.498487	-0.168294
H	5.904396	-1.224224	-0.796923	6.503014	-1.472717	-1.374925	6.210170	-1.213638	-2.492316	7.020624	1.139047	1.469902
H	3.900778	-1.207658	0.882521	3.636448	-2.473236	-2.228338	2.594522	-0.477131	-3.481289	4.852792	3.578102	-0.517565
H	4.624851	0.135274	1.758695	3.065669	-0.964079	-2.961908	4.156523	-1.012971	-4.116601	6.269913	3.476859	0.538496
H	2.975471	0.280111	1.130220	4.701918	-1.555456	-3.283856	3.232021	-2.078180	-3.066705	6.349856	2.831248	-1.095578
H	-2.360840	2.635934	-1.997120	-2.113836	2.783750	-0.979720	-2.175962	2.933270	-0.251341	-2.025880	2.218503	-1.772671
H	-2.123371	3.385102	-0.405257	-2.086201	3.765571	0.498069	-1.918313	3.685957	1.334591	-2.048390	3.297545	-0.362625

		**5b-13**			**5b-14**			**5b-15**			**5b-16**	
**Element**	**X**	**Y**	**Z**	**X**	**Y**	**Z**	**X**	**Y**	**Z**	**X**	**Y**	**Z**
C	-1.483033	-4.912455	-0.628603	0.012398	-4.306275	-1.511332	0.011236	-4.259830	-1.554142	1.047096	-2.918770	5.061309
C	-1.689741	-4.069479	0.608303	-0.162775	-3.502353	-0.243613	-0.158576	-3.466598	-0.278995	0.369537	-3.581797	3.884539
C	-2.335823	-4.803021	1.758325	1.129489	-3.115735	0.433507	1.136648	-3.081782	0.393438	-0.270138	-4.910805	4.204902
C	-1.309929	-2.783182	0.626874	-1.390265	-3.202830	0.208738	-1.384146	-3.173640	0.182779	0.370287	-2.992543	2.679555
C	-1.432031	-1.782219	1.753957	-1.774249	-2.436535	1.455682	-1.762769	-2.417149	1.437335	-0.249471	-3.489396	1.392252
C	-2.295702	-0.590977	1.380332	-2.711334	-1.283620	1.154022	-2.706060	-1.265452	1.150209	-1.296917	-2.534855	0.848058
C	-3.631461	-0.473809	1.778047	-4.085818	-1.343952	1.393540	-4.079903	-1.334364	1.401307	-2.668832	-2.769180	0.974235
C	-4.421088	0.631451	1.422332	-4.950777	-0.283150	1.078849	-4.948421	-0.272388	1.099481	-3.628414	-1.870166	0.479651
C	-5.830446	0.761146	1.853807	-6.388204	-0.453099	1.384504	-6.400338	-0.342268	1.373912	-5.080838	-2.118727	0.611279
O	-6.263157	-0.286433	2.608975	-7.139513	0.631912	1.045911	-6.788562	-1.522789	1.931500	-5.373469	-3.288221	1.244454
O	-6.564150	1.693559	1.582573	-6.891257	-1.441532	1.885413	-7.202234	0.542901	1.139738	-5.958426	-1.378224	0.207588
C	-3.880986	1.664696	0.633456	-4.445086	0.891220	0.490516	-4.448485	0.905946	0.514918	-3.223606	-0.689391	-0.169077
C	-2.561730	1.571121	0.229792	-3.084066	0.976756	0.250264	-3.091646	0.999381	0.263548	-1.870514	-0.434700	-0.303247
C	-1.801210	0.459099	0.602801	-2.249254	-0.092450	0.584314	-2.251345	-0.070130	0.584882	-0.940351	-1.346625	0.202544
O	-0.519004	0.508158	0.130967	-0.936141	0.145371	0.299692	-0.941059	0.173099	0.292474	0.352518	-0.964893	0.000257
C	-0.291139	1.849458	-0.420877	-0.800510	1.549254	-0.106232	-0.812325	1.580540	-0.105097	0.360332	0.360931	-0.635432
C	0.585674	1.695535	-1.678937	0.191327	1.590131	-1.286568	0.171831	1.631948	-1.291390	1.362489	0.304028	-1.807358
O	-0.137821	0.933117	-2.652485	-0.352365	0.811917	-2.358618	-0.375602	0.857842	-2.364507	2.635066	-0.071249	-1.264302
C	0.829560	3.061917	-2.323888	0.321798	3.015126	-1.828857	0.293618	3.060551	-1.826141	0.947435	-0.721997	-2.874551
C	1.912018	0.958559	-1.371335	1.568210	0.995233	-0.901552	1.553327	1.039935	-0.918530	1.564050	1.689437	-2.450941
C	2.885939	1.638397	-0.385731	2.381869	1.737372	0.179977	2.371587	1.779410	0.161347	2.065658	2.804634	-1.509489
C	4.105149	0.788069	-0.138776	3.659412	1.008830	0.508201	3.653783	1.053735	0.477517	2.478482	4.032649	-2.276914
C	5.388585	1.055784	-0.425106	4.926808	1.414063	0.333740	4.918592	1.464235	0.296679	1.960340	5.269865	-2.231590
C	6.474080	0.055227	-0.103307	6.081498	0.520588	0.722787	6.078925	0.572912	0.673699	2.524188	6.373988	-3.095334
C	5.886845	2.326886	-1.069166	5.339610	2.747557	-0.240813	5.322952	2.801959	-0.274017	0.810190	5.696641	-1.351699
C	-1.699558	2.452864	-0.643104	-2.237928	2.038995	-0.411130	-2.253122	2.067951	-0.397036	-1.119122	0.701324	-0.958605
H	-0.851042	-5.785391	-0.412285	0.601374	-3.751394	-2.255327	0.594131	-3.697206	-2.297062	0.333390	-2.737993	5.877541
H	-2.438685	-5.306375	-1.002403	0.561747	-5.237982	-1.315359	0.564459	-5.191463	-1.369021	1.837283	-3.560128	5.476701
H	-1.011366	-4.344939	-1.436970	-0.948035	-4.569195	-1.965471	-0.951124	-4.521760	-2.004786	1.498004	-1.960080	4.786742
H	-1.727784	-5.668818	2.055472	1.791581	-2.589580	-0.267921	1.792447	-2.547129	-0.307429	-1.020160	-4.795786	4.999716
H	-2.485280	-4.181880	2.644429	0.987254	-2.472816	1.304825	0.997925	-2.447121	1.271338	-0.762029	-5.377627	3.348413
H	-3.315476	-5.201070	1.459559	1.676648	-4.010986	0.761110	1.688822	-3.978117	0.709456	0.478720	-5.618646	4.586747
H	-0.856124	-2.378127	-0.278261	-2.242951	-3.552433	-0.375016	-2.239242	-3.520505	-0.399158	0.868383	-2.027410	2.587164
H	-1.840150	-2.247862	2.655253	-2.282908	-3.116737	2.153110	-2.263714	-3.104219	2.133922	0.544053	-3.605616	0.639811
H	-0.427933	-1.417136	2.011870	-0.886284	-2.061288	1.973662	-0.872647	-2.042353	1.951913	-0.702984	-4.475708	1.522991
H	-4.069386	-1.256424	2.388998	-4.516722	-2.232907	1.844879	-4.487955	-2.234318	1.850247	-3.004687	-3.674184	1.469918
H	-7.191598	-0.080803	2.822999	-8.053086	0.397044	1.291473	-7.751364	-1.440664	2.059383	-6.347028	-3.330923	1.266255
H	-4.511315	2.506531	0.365623	-5.116544	1.704259	0.236656	-5.138465	1.709594	0.278485	-3.981584	-0.011278	-0.547937
H	0.247858	2.412576	0.350495	-0.388579	2.082907	0.758447	-0.396229	2.109377	0.760530	0.762337	1.037818	0.123145
H	-0.336442	0.077396	-2.235556	-0.488905	-0.082738	-2.000813	-0.508459	-0.038797	-2.010354	2.489799	-0.891352	-0.763549
H	1.481162	2.945412	-3.195628	1.060306	3.034643	-2.636622	1.027158	3.087573	-2.638215	1.740460	-0.793677	-3.624761

H	1.305049	3.758567	-1.625970	0.641389	3.715321	-1.050231	0.615220	3.757463	-1.045399	0.018930	-0.444462	-3.385446
H	-0.112373	3.500716	-2.665669	-0.631250	3.361984	-2.238343	-0.663210	3.406071	-2.227785	0.807613	-1.711969	-2.428246
H	1.663922	-0.039495	-0.987751	1.409596	-0.040505	-0.575482	1.400541	0.001937	-0.596810	2.296742	1.554311	-3.256793
H	2.418507	0.807262	-2.331932	2.158240	0.946246	-1.824422	2.137364	0.997825	-1.845542	0.630319	2.004532	-2.933838
H	3.166826	2.628279	-0.758813	2.586779	2.762758	-0.143209	2.570808	2.807073	-0.158078	1.298869	3.050335	-0.766958
H	2.376162	1.801310	0.575366	1.781941	1.816030	1.098862	1.777474	1.851522	1.084540	2.927790	2.414333	-0.953475
H	3.888501	-0.176852	0.324086	3.508247	0.013666	0.931476	3.508927	0.055949	0.896858	3.309794	3.866896	-2.965502
H	6.071466	-0.849432	0.363046	5.739513	-0.433740	1.135517	5.742989	-0.384590	1.084067	3.355247	6.023825	-3.715619
H	7.019109	-0.243035	-1.010142	6.725338	0.306285	-0.142026	6.717738	0.365027	-0.196396	1.754091	6.789488	-3.760851
H	7.220682	0.486073	0.578878	6.722270	1.005266	1.473053	6.723025	1.056182	1.422026	2.887322	7.211004	-2.482098
H	6.426164	2.102120	-1.999869	4.495172	3.377184	-0.530496	4.474437	3.429867	-0.555354	-0.009241	6.107182	-1.958185
H	5.090389	3.035281	-1.308521	5.945160	3.310833	0.482970	5.931009	3.363973	0.448644	0.402142	4.884890	-0.744818
H	6.604488	2.838585	-0.412663	5.972032	2.606151	-1.128291	5.950272	2.667107	-1.166124	1.120936	6.500061	-0.669186
H	-1.989693	2.363316	-1.696908	-2.414000	2.055245	-1.493363	-2.437258	2.090059	-1.477762	-1.306990	0.739970	-2.038045
H	-1.735480	3.510736	-0.365958	-2.413829	3.045967	-0.020332	-2.429798	3.071792	0.001092	-1.395089	1.680235	-0.551921

	**5b-17**			**5b-18**			**5b-19**			**5b-20**		
**Element**	**X**	**Y**	**Z**	**X**	**Y**	**Z**	**X**	**Y**	**Z**	**X**	**Y**	**Z**
C	0.200839	-2.691805	3.977239	1.051531	-2.953422	5.068406	0.370035	-2.369452	4.133938	0.314665	-6.085730	0.239071
C	-0.626404	-3.120480	2.787510	0.362417	-3.612690	3.896233	-0.655512	-2.761389	3.095580	-0.225853	-5.220593	1.353848
C	-1.378136	-4.413777	2.988020	-0.302679	-4.926710	4.226200	-1.525911	-3.928698	3.492978	-0.932551	-5.985826	2.446105
C	-0.651626	-2.374308	1.673183	0.374632	-3.032311	2.687008	-0.738273	-2.092773	1.935743	-0.060029	-3.889842	1.323832
C	-1.413421	-2.628824	0.391442	-0.254369	-3.527598	1.403557	-1.689744	-2.326909	0.783472	-0.525086	-2.861467	2.330607
C	-2.416184	-1.530151	0.087392	-1.297072	-2.566621	0.861811	-2.570453	-1.121394	0.506962	-1.470462	-1.841625	1.721836
C	-3.787581	-1.678194	0.306030	-2.668773	-2.790312	0.989705	-3.908751	-1.057815	0.907452	-2.858727	-1.923196	1.863419
C	-4.705406	-0.649337	0.040195	-3.626437	-1.887728	0.498604	-4.707364	0.071336	0.663361	-3.724547	-0.977532	1.290046
C	-6.135374	-0.916352	0.311082	-5.053875	-2.227535	0.687721	-6.122568	0.139373	1.089729	-5.193403	-1.058856	1.446732
O	-6.940794	0.146115	0.030677	-5.899629	-1.286367	0.182388	-6.552715	-0.989945	1.717873	-5.599236	-2.125609	2.189473
O	-6.589587	-1.960877	0.739443	-5.474127	-3.231622	1.231623	-6.862803	1.089645	0.915590	-5.993529	-0.272321	0.974915
C	-4.259534	0.584362	-0.470719	-3.219103	-0.708185	-0.151461	-4.172761	1.186193	-0.009135	-3.205535	0.093798	0.539925
C	-2.903330	0.756024	-0.690934	-1.862719	-0.463227	-0.287835	-2.849843	1.147750	-0.410555	-1.834776	0.195286	0.386500
C	-2.016716	-0.286306	-0.409748	-0.936162	-1.379613	0.215078	-2.079719	0.011836	-0.146475	-0.998796	-0.762247	0.968536
O	-0.714881	0.033151	-0.679495	0.358214	-1.004729	0.008717	-0.795036	0.120734	-0.601472	0.324455	-0.533004	0.733922
C	-0.651018	1.470160	-0.971181	0.371043	0.322236	-0.623332	-0.580711	1.509235	-1.027927	0.460039	0.757465	0.042899
C	0.365023	1.671503	-2.112604	1.373838	0.265136	-1.794696	0.291994	1.479212	-2.297994	1.509581	0.577001	-1.072411
O	-0.122069	1.004221	-3.283125	2.644827	-0.115150	-1.251698	-0.430063	0.803797	-3.334830	2.725927	0.142096	-0.450360
C	0.452546	3.152469	-2.488292	0.956473	-0.757187	-2.864544	0.523205	2.900968	-2.814918	1.075506	-0.465969	-2.115343
C	1.753034	1.083344	-1.759679	1.579963	1.651460	-2.434912	1.624858	0.726840	-2.064426	1.837090	1.914510	-1.764116
C	2.497786	1.702234	-0.557832	2.085536	2.762544	-1.490735	2.598159	1.320022	-1.023796	2.372334	3.035213	-0.848114
C	3.803058	0.997217	-0.293495	2.501820	3.991362	-2.254974	3.822736	0.457831	-0.857442	2.903301	4.196315	-1.646444
C	5.051175	1.484869	-0.366023	1.989196	5.230662	-2.204348	5.103470	0.756760	-1.124330	2.471819	5.466789	-1.677279
C	6.241029	0.602439	-0.068514	2.556072	6.335347	-3.065398	6.195680	-0.261845	-0.895228	3.149006	6.489800	-2.559292
C	5.407519	2.905353	-0.732112	0.842882	5.659424	-1.320408	5.592011	2.082003	-1.657081	1.317185	6.010320	-0.870695
C	-2.107345	1.899923	-1.274920	-1.107117	0.668426	-0.946283	-1.994521	2.119662	-1.189333	-0.974021	1.186788	-0.362632
H	-0.428926	-2.556968	4.868023	0.340624	-2.753016	5.882471	-0.113081	-2.094828	5.082359	1.030284	-6.826204	0.623487
H	0.946647	-3.456177	4.237737	1.829047	-3.606444	5.489494	1.044771	-3.207052	4.360887	-0.491307	-6.655384	-0.244767
H	0.729728	-1.751917	3.790510	1.520766	-2.005546	4.786924	0.980182	-1.521857	3.806536	0.820053	-5.493760	-0.530395
H	-2.054694	-4.336217	3.850347	-1.053046	-4.790465	5.017225	-2.064048	-3.708297	4.425366	-0.254149	-6.717147	2.907097
H	-1.975772	-4.711099	2.123355	-0.801075	-5.391773	3.372621	-2.267109	-4.198790	2.737284	-1.325498	-5.349390	3.242278
H	-0.683075	-5.234767	3.212888	0.431763	-5.644788	4.616832	-0.912775	-4.818521	3.692902	-1.772221	-6.561139	2.032108
H	-0.063744	-1.456488	1.663115	0.889856	-2.076844	2.587910	-0.048471	-1.263875	1.777227	0.469671	-3.466780	0.469601
H	-0.695220	-2.689906	-0.438848	0.534972	-3.651668	0.647974	-1.101962	-2.547990	-0.119053	-1.017179	-3.336368	3.183845
H	-1.938356	-3.587369	0.423069	-0.715424	-4.509640	1.538663	-2.326895	-3.197260	0.963022	0.352197	-2.330756	2.726735
H	-4.174415	-2.614129	0.697891	-3.025770	-3.689127	1.483515	-4.343753	-1.905695	1.426484	-3.281562	-2.739015	2.440659
H	-7.843106	-0.153061	0.246097	-6.795874	-1.622231	0.366883	-7.485100	-0.817377	1.943650	-6.571719	-2.064521	2.215206
H	-4.972193	1.373349	-0.684872	-3.960458	-0.015289	-0.534450	-4.810995	2.043776	-0.196064	-3.892553	0.812442	0.104669
H	-0.292971	1.954630	-0.054953	0.774482	0.996200	0.137165	-0.042678	2.002615	-0.209733	0.872309	1.440531	0.791240
H	-0.215529	0.066609	-3.042679	2.497086	-0.936136	-0.753089	-0.614942	-0.090111	-2.999596	2.500744	-0.640223	0.080256
H	1.200823	3.288069	-3.275555	1.750370	-0.830394	-3.613684	1.171522	2.870448	-3.696312	1.899653	-0.629913	-2.815884
H	0.735983	3.770159	-1.630028	0.029841	-0.475020	-3.376351	0.997016	3.533443	-2.057312	0.198917	-0.146176	-2.689291
H	-0.506958	3.510570	-2.872714	0.812116	-1.747444	-2.420383	-0.423266	3.362703	-3.110876	0.837532	-1.421082	-1.635788
H	1.628983	0.007637	-1.580050	2.312041	1.515975	-3.241256	1.385083	-0.304078	-1.773623	2.594378	1.693084	-2.527007
H	2.374375	1.180835	-2.657770	0.647175	1.970885	-2.916855	2.128408	0.668414	-3.036616	0.950315	2.269407	-2.304545
H	2.656393	2.772206	-0.725362	1.320001	3.008550	-0.746995	2.872500	2.341298	-1.306209	1.592132	3.365668	-0.154018
H	1.870172	1.618066	0.341780	2.946721	2.367946	-0.936315	2.090498	1.392405	-0.050479	3.179525	2.613416	-0.235518
H	3.693678	-0.054404	-0.020867	3.331070	3.824432	-2.945757	3.613059	-0.545121	-0.480134	3.750217	3.943366	-2.287905
H	5.939370	-0.416550	0.193463	3.384415	5.983764	-3.688494	5.799925	-1.206061	-0.508154	3.979808	6.056139	-3.124750
H	6.917798	0.545501	-0.932852	1.786499	6.756452	-3.728015	6.738582	-0.476630	-1.826607	2.439835	6.926393	-3.276974
H	6.835198	1.005787	0.763771	2.924017	7.168680	-2.450025	6.942728	0.112137	-0.180746	3.542768	7.326927	-1.965414
H	4.538786	3.528008	-0.958146	0.024267	6.076556	-1.923533	6.128700	1.942149	-2.605753	0.555966	6.448300	-1.531420
H	5.963138	3.388365	0.083866	0.432177	4.847118	-0.716039	4.790574	2.803713	-1.831141	0.826940	5.256458	-0.250135
H	6.069642	2.923161	-1.608954	1.158926	6.458440	-0.635164	6.309488	2.538961	-0.961201	1.653556	6.820428	-0.208547
H	-2.264293	1.972812	-2.357854	-1.295107	0.705429	-2.025878	-2.284295	2.134310	-2.246871	-1.134614	1.118860	-1.445308
H	-2.344078	2.870323	-0.827937	-1.377857	1.649806	-0.541821	-2.039247	3.144004	-0.807239	-1.170353	2.224875	-0.074494

	5b-21			5b-22			5b-23		
**Element**	**X**	**Y**	**Z**	**X**	**Y**	**Z**	**X**	**Y**	**Z**
C	0.334606	-6.104603	0.264088	0.576746	-2.321577	3.823623	0.570462	-2.419023	3.806405
C	-0.221619	-5.243398	1.374176	0.180117	-2.486383	2.374985	0.173283	-2.556042	2.355013
C	-0.956471	-6.010762	2.446065	1.339372	-2.503290	1.409535	1.332434	-2.558448	1.389256
C	-0.045434	-3.913757	1.356738	-1.110865	-2.610020	2.032701	-1.118042	-2.670149	2.010754
C	-0.523799	-2.889533	2.361568	-1.707956	-2.792545	0.654831	-1.715608	-2.826806	0.629909
C	-1.467295	-1.871887	1.746438	-2.701775	-1.698149	0.313133	-2.703053	-1.721201	0.306675
C	-2.854450	-1.951177	1.882124	-4.074814	-1.837597	0.540436	-4.075140	-1.852746	0.533439
C	-3.721188	-1.009459	1.304185	-4.986727	-0.806149	0.263878	-4.983256	-0.813770	0.274548
C	-5.174011	-1.186629	1.520562	-6.438043	-0.952533	0.510657	-6.413899	-1.062785	0.557659
O	-5.926116	-0.222814	0.919020	-6.780027	-2.170368	1.015634	-7.208909	0.008036	0.279337
O	-5.687729	-2.084562	2.161210	-7.275898	-0.095824	0.297672	-6.876824	-2.100151	0.994084
C	-3.202936	0.060949	0.551693	-4.532886	0.413859	-0.270557	-4.525422	0.412608	-0.242487
C	-1.829735	0.160781	0.403795	-3.179490	0.573869	-0.505431	-3.169440	0.565771	-0.476486
C	-0.994652	-0.793517	0.991067	-2.294562	-0.467667	-0.210731	-2.290042	-0.484581	-0.199615
O	0.329191	-0.563246	0.759948	-0.995877	-0.162504	-0.505073	-0.990224	-0.182380	-0.492535
C	0.465412	0.725031	0.066155	-0.927144	1.267399	-0.831377	-0.913852	1.251288	-0.797757
C	1.519790	0.543446	-1.044456	0.067108	1.437473	-1.997117	0.077900	1.432934	-1.963929
O	2.734429	0.112937	-0.416252	-0.439702	0.733551	-3.137423	-0.437323	0.750190	-3.113327
C	1.091929	-0.503474	-2.086003	0.141066	2.907084	-2.419154	0.160246	2.908376	-2.363456
C	1.847512	1.879536	-1.738782	1.465531	0.867729	-1.656433	1.473522	0.848817	-1.636517
C	2.378049	3.003489	-0.824055	2.229607	1.523674	-0.487065	2.245748	1.481971	-0.459941
C	2.909503	4.163462	-1.623672	3.563349	0.860978	-0.258085	3.575149	0.806126	-0.244454
C	2.477172	5.433560	-1.657796	4.792190	1.395339	-0.331820	4.807785	1.331964	-0.316119
C	3.155418	6.455482	-2.540256	6.016612	0.548640	-0.072946	6.026585	0.472749	-0.072444
C	1.320621	5.977763	-0.854396	5.093236	2.837176	-0.662231	5.118714	2.775494	-0.629590
C	-0.967814	1.150672	-0.346414	-2.385124	1.701685	-1.119821	-2.370083	1.698363	-1.075789
H	1.036835	-6.853212	0.657345	1.251421	-3.128234	4.143696	1.242717	-3.233468	4.111505
H	-0.465887	-6.664670	-0.239578	1.123328	-1.380631	3.978994	1.119863	-1.482711	3.979108
H	0.859657	-5.511097	-0.490953	-0.292381	-2.323068	4.488792	-0.298673	-2.430358	4.471440
H	-0.294716	-6.754010	2.912148	1.999066	-3.359337	1.609929	1.989821	-3.419813	1.573846
H	-1.357471	-5.377596	3.240712	1.031721	-2.552965	0.362909	1.024473	-2.588200	0.341950
H	-1.794878	-6.572256	2.011257	1.955187	-1.602008	1.535040	1.950775	-1.661279	1.531068
H	0.503708	-3.488569	0.515915	-1.851151	-2.575175	2.833245	-1.858342	-2.648420	2.811666
H	-1.022931	-3.368050	3.208459	-0.927177	-2.833450	-0.109803	-0.935007	-2.859477	-0.135325
H	0.347798	-2.356716	2.767343	-2.234608	-3.756661	0.618729	-2.248244	-3.786698	0.578302
H	-3.295401	-2.759356	2.457973	-4.447985	-2.772483	0.946549	-4.470112	-2.785142	0.926493
H	-6.849961	-0.448824	1.132383	-7.747436	-2.137202	1.130134	-8.112954	-0.279502	0.503185
H	-3.874394	0.786311	0.105173	-5.255018	1.194191	-0.488314	-5.229383	1.210285	-0.453377
H	0.873255	1.411301	0.814041	-0.548583	1.769451	0.066871	-0.529900	1.738080	0.106605
H	2.508959	-0.668460	0.115631	-0.537787	-0.193930	-2.862063	-0.538764	-0.181220	-2.852784
H	1.919484	-0.668453	-2.782267	0.878773	3.022757	-3.219467	0.895945	3.031542	-3.164492
H	0.217539	-0.186688	-2.665012	0.430955	3.552996	-1.584166	0.457340	3.539301	-1.519597
H	0.853028	-1.457347	-1.604508	-0.825166	3.247526	-2.802290	-0.804993	3.261168	-2.737913
H	2.607661	1.657238	-2.498564	1.353534	-0.203793	-1.446150	1.354950	-0.224936	-1.441924
H	0.961969	2.231574	-2.283148	2.067248	0.945752	-2.569696	2.072847	0.936522	-2.550474
H	1.595262	3.334318	-0.133035	2.351980	2.595305	-0.672579	2.375595	2.555275	-0.630162
H	3.184078	2.584548	-0.207996	1.632077	1.433220	0.432259	1.650199	1.382450	0.459714
H	3.757954	3.910002	-2.262890	3.496492	-0.201155	-0.013375	3.500913	-0.258419	-0.012821
H	3.987476	6.021322	-3.103464	5.755205	-0.487619	0.163429	5.758064	-0.564354	0.151896
H	2.447373	6.890569	-3.259979	6.684052	0.540379	-0.946310	6.690778	0.470367	-0.948313
H	3.547704	7.293770	-1.947042	6.606404	0.949855	0.763461	6.622434	0.858905	0.766749
H	0.560459	6.414281	-1.517346	4.200437	3.433846	-0.862909	4.229800	3.381466	-0.819399
H	0.829700	5.224588	-0.233516	5.638902	3.317657	0.161922	5.671149	3.241418	0.198403
H	1.655223	6.789135	-0.192901	5.745760	2.902891	-1.543953	5.768423	2.847249	-1.512918
H	-1.123842	1.078143	-1.429561	-2.559841	1.757265	-2.200961	-2.546664	1.771703	-2.155629
H	-1.166012	2.189910	-0.063380	-2.609472	2.680754	-0.685725	-2.587037	2.672207	-0.626187
